# Synthesis of Large Macrocycles with Chiral Sulfur Centers via Enantiospecific SuFEx and SuPhenEx Click Reactions

**DOI:** 10.1021/acs.joc.3c01656

**Published:** 2023-10-30

**Authors:** Yang Chao, Muthusamy Subramaniam, Kayambu Namitharan, Yumei Zhu, Victor Koolma, Zitong Hao, Shikang Li, Yaxin Wang, Ilyos Hudoynazarov, Fedor M. Miloserdov, Han Zuilhof

**Affiliations:** ‡ School of Pharmaceutical Science and Technology, Tianjin University, 92 Weijin Road, Tianjin 300072, China; † Laboratory of Organic Chemistry, Wageningen University, Stippeneng 4, 6708WE Wageningen, The Netherlands; § Division of Organic Synthesis and Applied Chemistry, National University of Uzbekistan, Tashkent 100174, Uzbekistan

## Abstract

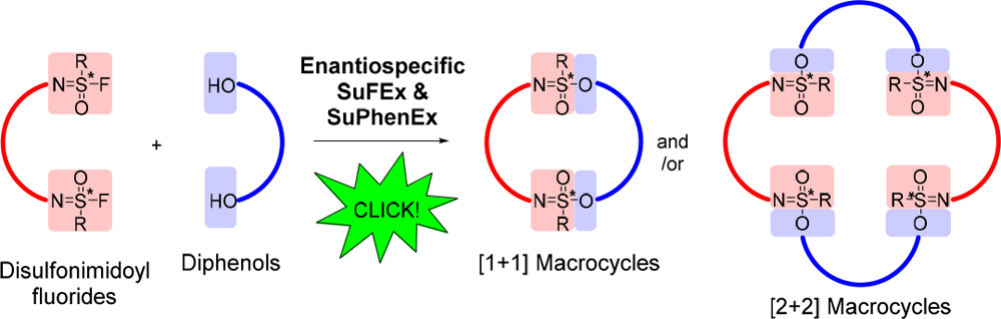

Here we report the first asymmetric synthesis of large chiral macrocycles with chiral sulfur atoms. Building on stereospecific SuFEx and SuPhenEx click chemistries, this approach utilizes disulfonimidoyl fluorides and disulfonimidoyl *p*-nitrophenolates—which are efficient building blocks with two chiral sulfur centers, and diphenols to efficiently form novel S–O bonds. Characteristic results include the enantiospecific one-step synthesis of rings consisting of 21–58 members and characterization of both enantiomers (***R,R*** and ***S,S***) by e.g. X-ray crystallography.

## Introduction

Macrocycles, typically having 12 or more ring atoms, are noteworthy due to their distinct molecular structure and properties^1^ resulting in a vast range of applications, especially in the areas of supramolecular chemistry^2^ and drug design.^3^ Their ability to bind specifically to target molecules makes them useful as highly selective and specific host molecules and therapeutic agents.^4^ Since 2013 more than 25 macrocyclic drugs have been approved for clinical use, for a wide range of illnesses, stressing the need for additional methods to construct such macrocycles.^5^ However, the synthesis of large macrocycles is often highly challenging, typically involves complex multistep syntheses, and often requires precise control over the reaction conditions.^1,3c,6^ In some cases this synthetic challenge can be overcome by a kinetically or thermodynamically preferred cyclization product, such as in the case of pillararenes.^7^ While, of course, these products can be varied in structure as well,^8^ such variation often comes at the price of more complicated syntheses. In combination with the formation of byproducts and solubility issues, the formation of large macrocycles is typically difficult to scale up.

These factors only increase when the formation of chiral macrocycles is investigated.^9^ Consequently, there are not many reports in the literature on the synthesis of large macrocycles with multiple chiral centers,^10^ especially on macrocycles with stereogenic heteroatoms,^11^ and in fact enantiopure macrocycles with chiral-at-sulfur linkages have not been reported at all. On the other hand, the synthesis of sulfur-centered chiral molecules has gained significant attention in recent years,^12^ specifically in the area of drug discovery^13^ and as catalysts/ligands in asymmetric catalysis.^14^ Although significant progress has been made toward synthesizing S-centered chiral molecules,^14c,15^ the challenge of accessing macrocycles that contain chiral sulfur has yet remained an unmet goal in synthetic chemistry.

**Figure 1:**
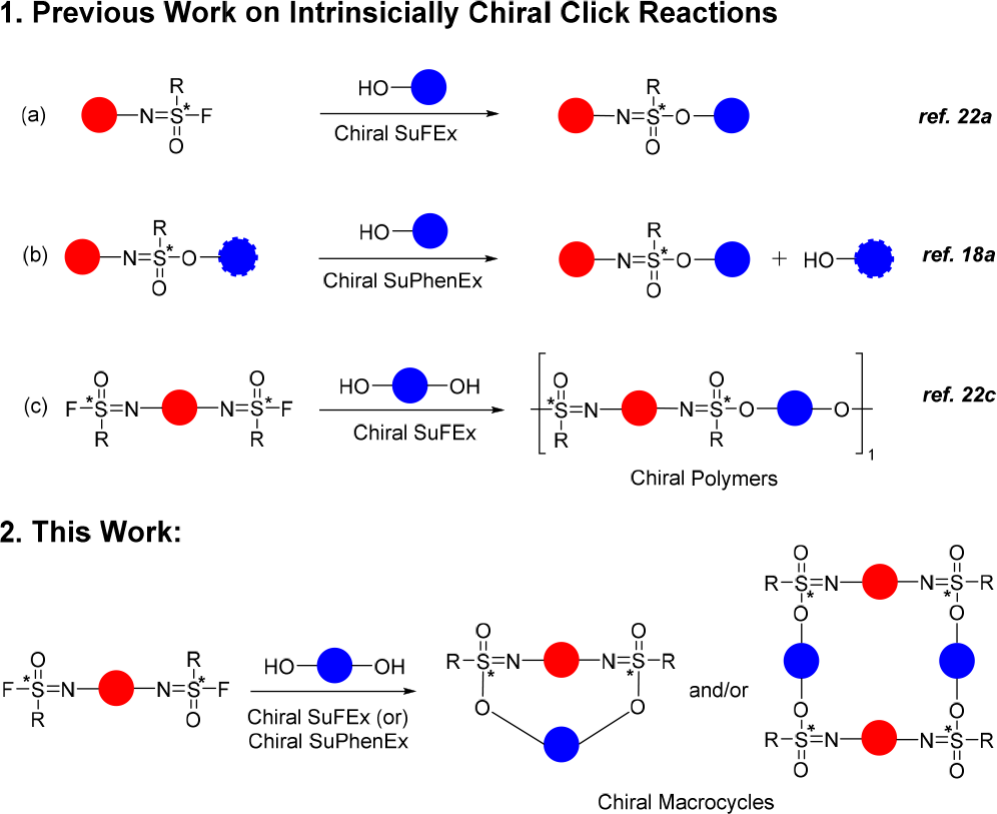
Enantiospecific synthesis of sulfonimidate linkages via SuFEx reactions.

In this, we noted the potential of enantiospecific S(VI) exchange reactions.^16^ Such reactions, like the sulfur–fluorine exchange (SuFEx)^17^ and sulfur–phenolate exchange (SuPhenEx)^18^ click reactions, have become widely popular tools in synthetic chemistry for the synthesis of both small molecules,^15f,19^ biologically relevant molecules,^13c,20^ and new functional organic materials.^21^ Importantly, these reactions are highly efficient, resulting in high yields of the desired product, and they are often performed under mild conditions. Our research group has shown a keen interest in the area of sulfur(VI) exchange reactions, specifically as a tool to introduce intrinsically chiral click chemistries.^18,22^ Using the enantiospecificity observed for small-molecule SuFEx and SuPhenEx reactions of chiral sulfonimidoyl fluorides and phenolates,^18a,22a^ our group synthesized, for example, configurationally chiral polymers using disulfonimidoyl fluorides (further: di-SFs) and diphenols^22c^ (Figure 1). Interestingly, in nearly all polymerization reactions byproducts were observed in low yields (typically <5%) with a distinct mass that could be directly related to the structure of the di-SFs and diphenolates used. We hypothesized that these might be macrocycles, and that by careful adjustment of the reaction conditions, it should be possible to regulate the polymerization and steer the SuFEx and SuPhenEx coupling reactions to macrocycle formation. Trace amounts of similar SuFEx-derived achiral sulfonyl macrocyclic compounds have recently been observed in the polymerization of diamine-derived sulfonyl fluorides.^23^ We therefore imagined that the development of such a novel class of macrocyclic compounds with sulfonimidate linkages would display a series of potential advantages: (a) the sulfonimidate linkage, which can be decorated at will with a wide variety of functionalities,^18a,22a^ is rapidly formed using the SuFEx or SuPhenEx click reactions; (b) a wide range of macrocycles can be formed given the easy variation of the structure of the di-S(VI) compounds, and especially the diphenolate moieties; (c) such an approach would be easily be extendable to the enantiospecific synthesis of macrocycles with chiral sulfur centers, and thus open up a new region in chemical space as these are not yet reported.

In this report, we thus describe the development of a generic synthesis route for this novel class of SuFEx- and SuPhenEx-based macrocycles with multiple chiral sulfur centers and subsequently describe the synthesis of a wide range of macrocycles, in both racemic and enantiopure form (21- to 58-membered rings; in many cases both enantiomers), study their structure by DOSY NMR, circular dichroism, and X-ray crystallography, and outline the potential of this class of materials for a range of applications.

## Results and Discussion

We initiated our investigation by examining *N*′,*N*″-terephthaloyl-*bis*-sulfonimidoyl fluoride (**1a**) and 4,4′-disulfanediyldiphenol (**2a**) as model substrates using DBU as base and DMF as solvent (as shown in Table 1; all yields are isolated yields). When the reaction temperature was decreased from 80 to 50 °C, a significant improvement in the yield of the macrocyclic product was observed. Our findings also revealed that as the reaction concentration decreased, the yields of the macrocycles increased. Moreover, when we switched the solvent to acetonitrile, a maximum macrocycle yield of 96% was achieved. Thus, the optimal conditions were found to be the stirring of **1a** (1 equiv) with **2a** (1 equiv), and DBU (2.1 equiv), in ACN at 50 °C for 3 h (Table 1, entry 6). The structure of product **3a** was determined by NMR spectroscopic analyses and X-ray crystallography to confirm the formation of the macrocycles (see Supporting Information, Figures S21–22 and S188–189).

**Table 1:**
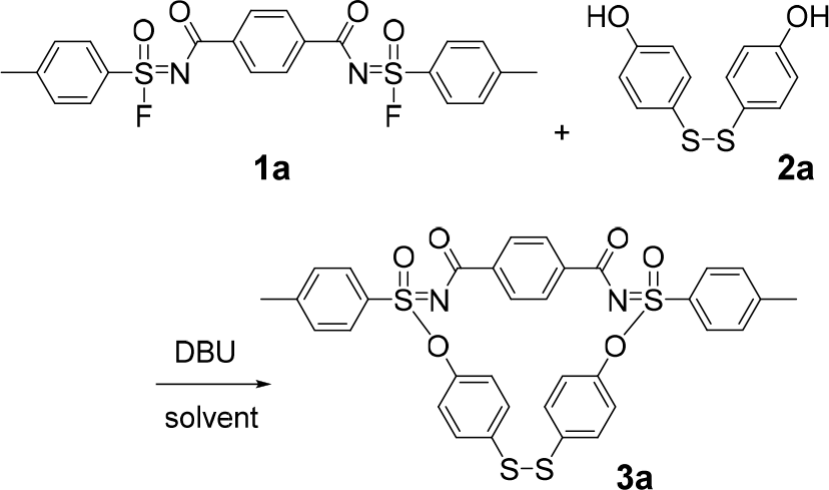
Optimization of SuFEx Macrocyclization Conditions^a^

Entry	Solvent	Conc. of **1a** and **2a**	Temp. (°C)	Yield of **3a**	Yield of oligomer + polymer
1	DMF (1 mL)	0.1 M	80	8	59
2	DMF (2 mL)	0.05 M	80	15	45
3	DMF (2 mL)	0.05 M	50	41	26
4	DMF (5 mL)	0.02 M	50	72	6
5	DMF (10 mL)	0.01 M	50	79	7
6	ACN (10 mL)	0.01 M	50	96	0

a Conditions: **1a** (0.1 mmol), **2a** (0.1 mmol), DBU (0.21 mmol), solvent, Ar atmosphere, 3 h.

**Figure 2:**
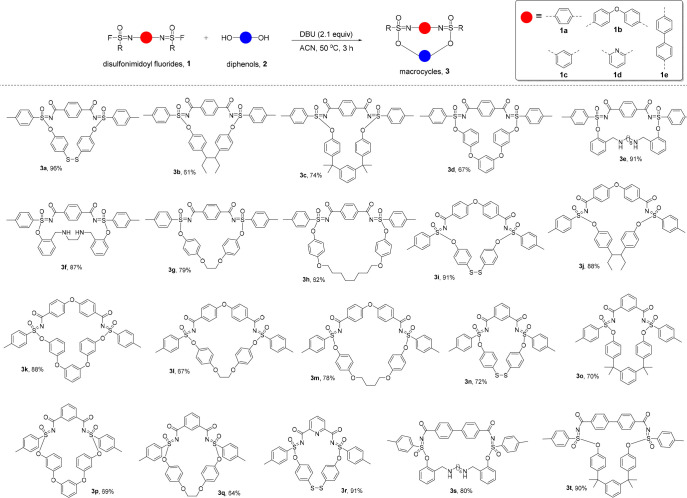
SuFEx synthesis of macrocycles. Typical reaction conditions: 0.2 mmol of **1**, 0.2 mmol of **2** in 10 mL of acetonitrile, 50 °C, 3–5 h.

With the optimized conditions in hand, the scope of the reaction was explored with **1a** and a variety of diphenols. The corresponding macrocyclic products **3a**–**h** were isolated in good to excellent yields (67–96%; Figure 2). While the compatibility with disulfide (**3a**) or ether (**3d**) moieties was to be expected based on past experience, it is worth noting that diphenols with secondary amine groups also reacted well in these macrocyclic SuFEx reactions. Even though secondary amines have been reported to be able to react in high yields with sulfonimidoyl fluorides,^15f^ our cyclizations yielded only phenol-based products **3e** and **3f**, and in high yields. Furthermore, increased flexibility of the diphenol precursors and the ensuing increased ring sizes are well tolerated, and even macrocycle **3h**, which contains a flexible 30-atom ring, could be synthesized with an isolated yield of 62%.

Apart from variation of the diphenol moiety, changes in the structure of the di-SF unit are also nicely tolerated. Various diphenols were reacted with the diphenyl ether-based di-SF **1b**, resulting in the production of macrocycles **3i**–**m**, with isolated yields ranging from 67% to 91%. Moreover, di-SFs based on isophthaloyl, pyridyl, and biphenyl moieties also worked well and yielded the corresponding macrocycles **3n**–**t** in good to excellent isolated yields (ranging from 64% to 91%). In short: these SuFEx-based macrocycles can be readily synthesized in a single step under mild reaction conditions.

**Figure 3:**
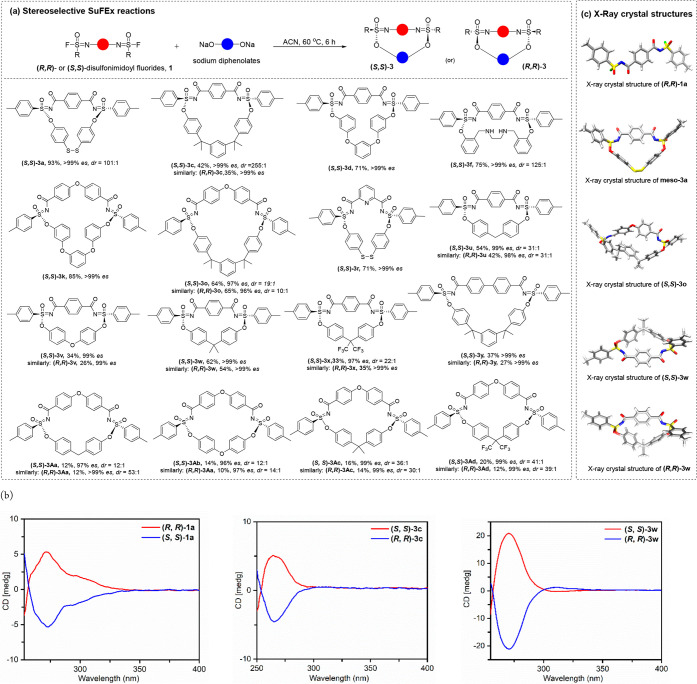
Asymmetric SuFEx synthesis of chiral macrocycles. (a) Synthesis, yield and enantioselectivity. (b) Circular dichroism spectra for both enantiomers of di-SF reactants **1a** and macrocyclic product **3c** and **3w**. (c) X-ray structures of various reactants and macrocyclic products. Notes: Typical reaction conditions: 0.5 mmol of **1**, 0.5 mmol of **2** in 40 mL acetonitrile, 60 °C, 6 h. *es* = enantioselectivity, given by % of **(*****S*****)**-stereocenters in **3**/ % of **(*****R*****)**-stereocenters in **1**. . For a few macrocycles, *dr* could not be determined, as the HPLC peak for the meso compound was too small or partly overlapped. As pointed out by a reviewer of a previous version of this manuscript, in the CIP rules for sulfonates and related compounds alkoxy (RO−) > oxo (O= ).^24^

The consistently high yields opened up the possibility for stereospecific macrocyclization, which we regard as highly relevant for, e.g., medicinal chemistry or highly functionalized rotaxane-based materials. Four different enantiopure chiral di SFs, including a pyridine-derived di-SF **1d**, were synthesized from the corresponding chiral sulfinamides and utilized in these reactions (disulfonimidoyl fluorides **1a**, **1b**, and **1e** were synthesized in both **(*****R*****,*****R*****)** and **(*****S*****,*****S*****)** stereoisomeric forms); % *ee* for all starting materials **1**: 96–99+%. We were pleased to observe that the SuFEx macrocyclization using premade sodium diphenolates proceeded with excellent stereoselectivity (*es* = 97–99+% in all cases, and correspondingly high diastereoisomeric ratios *dr*). As can been seen in Figure 3, the isolated yields of the various chiral macrocycles varied quite a bit, with several >70%, but also a few <20%. One factor that could possibly play a role is the ring strain in the macrocycle that was formed. To test this hypothesis, we calculated a measure of the ring strain for the five highest-yielding macrocycles and for the four lowest-yielding macrocycles (**3Aa**, **3Ab**, **3Ac**, **3Ad**). As a measure of the ring strain, we compared by performing MMFF94 molecular mechanics geometry optimizations, the total steric energy as calculated for MMFF94-optimized structures for both a macrocycle and its corresponding ring-opened structure that resulted from hydrolysis of one of the sulfonimidate moieties. This yielded two data sets that differed only to a statistically insignificant degree; i.e., there was via this approach no clear difference in the strain energy noticeable. [Several other factors do play a clearer role, and are discussed below.] As expected, the corresponding macrocycles were formed with inverted stereochemistry as confirmed by circular dichroism for macrocycles **3c** and **3w** resulting from phenyl-derved chiral di-SF **1a** (Figure 3b), and by X-ray crystallography for **3c** and **3w** (Figure 3c; see also, Supporting Information, Figures S190–191).

With the chiral macrocycles in hand, we tried, by various means, also to isolate meso macrocycles from a mixture of diastereomers. Typically, this turned out to be very difficult due to highly similar *R*_*f*_ values on TLC. While unsuccessful in using column chromatography, we found that **meso-3u** can be isolated from diastereomeric **3u** by preparative TLC. In ^1^H NMR analyses **meso-3u** showed slight chemical shift differences with **racemic-3u** (see Supporting Information, Figures S80–81), with their diastereoisomeric relation. Both chiral HPLC results revealed the ratio of (R,R), (S,S), and **meso-3u** to be 1:1:2, and also the isolated ratio of **racemic-3u** and **meso-3u** was 1:1 (see Supporting Information, Figure S152). Thus, we suppose that there is, at least in this case, no preference for the formation of racemic or meso compounds. In addition, the structure of **meso-3u** was also unambiguously determined by X-ray crystallography, which clearly displayed its higher symmetry, and which is also in line with the observation that it showed a significantly poorer solubility than **racemic-3u** (see Supporting Information, Figure S193).

**Figure 4:**
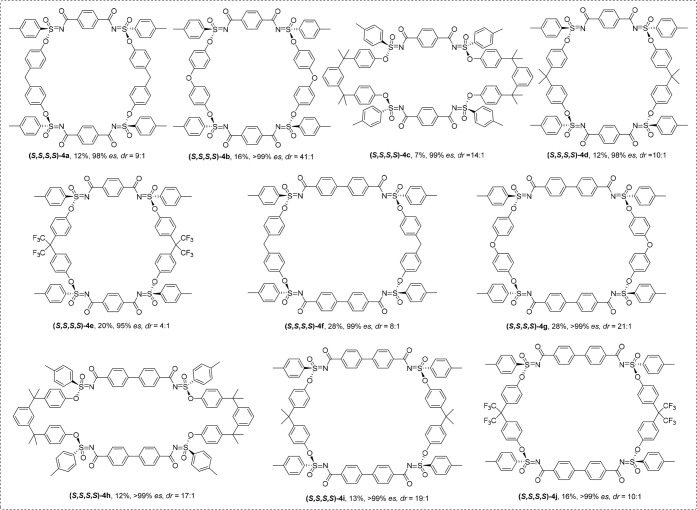
Isolated larger [2 + 2] chiral macrocycles, *es* = enantioselectivity, given by % of **(*****S*****)**-stereocenters in **4**/% of **(*****R*****)**-stereocenters in **1**. Note: the [2 + 2] chiral macrocycles were obtained from the same reaction mixture as reported in Figure 3 for the [1 + 1] chiral macrocycles (**4f**, **4g**, **4i**, and **4j** were not observed as the corresponding [1 + 1] macrocycles.

The number of atoms forming the ring of the isolated enantiopure macrocycles ranged from 24 to 32. However, we were surprised to discover that in a few enantiospecific SuFEx reactions minor products consisting of large macrocycles were also isolated (Figure 4). We hypothesized that these structures resulted from the reaction of two di-SFs and two diphenolates, and this was indeed shown to be the case by both NMR and HRMS studies. These experiments showed several things: (1) Large macrocylces, with up to 58 ring atoms (**4h**) could be readily obtained in a 7–28% isolated yield for the whole series of 10 compounds that we tried. We thus believe this is a general one-pot route for making such large macrocycles. (2) DOSY NMR studies indicated significant differences in the observed diffusion coefficient (*D*) of these larger [2 + 2] macrocycles with those of the previously mentioned [1 + 1] macrocycles, providing further support for the conclusion that these larger species are indeed [2 + 2] macrocycles: all [1 + 1] macrocycles displayed diffusion coefficients *D* in the range of (4.4–5.9) × 10^–10^ m^2^ s^–1^, whereas for all investigated [2 + 2] macrocycles *D* fell in the range of (2.6–3.8) × 10^–10^ m^2^ s^–1^ (see Supporting Information for 21 examples). (3) Also for these larger macrocycles, which would potentially suffer more from epimerization events and accompanying reduced enantioselectivity due to slower kinetics and more reaction sites, the coupling reaction of the four involved sulfur atoms is basically enantiospecific. (4) For several of the [2 + 2] macrocycles (**4f**, **4g**, **4i**, **4j**), such as the one obtained from the biphenyl-desired di-SF, geometric strain basically prevents the formation of [1 + 1] macrocycles, and such larger rings gave in fact rise to the highest isolated yields (up to 28%) of [2 + 2] product without significant optimization. This suggests a specific entry point to obtaining such large macrocycles, as the SuFEx reaction is apparently sufficiently facile and efficient that it even tolerates the formation of four chiral-at-sulfur linkages readily and enantiospecifically. (5) All structures that gave significant yields of the [2 + 2] macrocycle were derived from rigid di-SF compounds (phenyl-bridged, biphenyl-bridged). Diphenylether-linked di-SF compounds showed no significant yield of the [2 + 2] macrocycles. We would argue that this displays the competition between the formation of larger macrocycles and polymerization. If the di-SF has only limited conformation freedom, relatively little entropy is lost upon the formation of a macrocycle; this is the case for the phenyl- or biphenyl-bridged di-SF compounds. However, if the di-SF displays significant rotational freedom, then ring formation, and especially the formation of [2 + 2] macrocycles, would be accompanied by a significant loss of disorder. As a result, if a di-SF compound has reacted once with a diphenol but rather than reacting with that same diphenol again to form the [1 + 1] macrocycle reacts with another diphenol, then such species does not readily form [2 + 2] products, if the di-SF displays significant conformational freedom. Such a di-SF compound would not easily participate in the formation of such large macrocycles and rather forms polymers. We note that in our previous report on the formation of SuFEx-based polymers with chiral backbones,^22c^ indeed these diphenyl ether-bridged di-SF compounds were among the better SuFEx agents for the formation of such polymers.

Finally, we were interested in determining whether these macrocycles could also be synthesized via enantiospecific sulfur phenolate exchange (SuPhenEx) reactions. In this manner, no independent synthesis of both enantiomers of a di-SF would be needed, but only one, the stereochemistry of which could then be inverted via a SuFEx reaction with *p*-nitrophenolate and subsequently reacted with the corresponding diphenolate.

**Figure 5:**
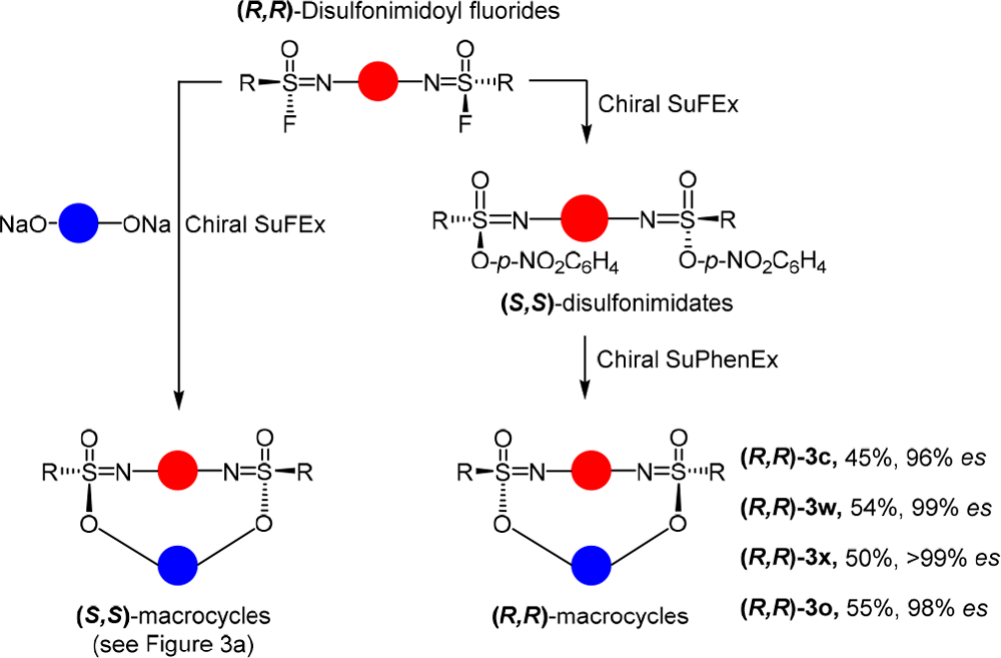
Synthesis of **(*****R*****,*****R*****)**-macrocycles via subsequent enantiospecific SuFEx and SuPhenEx reactions.

To investigate this, *p*-nitrophenol-derived disulfonimidates were synthesized and reacted with the disodium salts of diphenols in acetonitrile at 50 or 60 °C for 0.5–5 h. We were pleased to find that the desired macrocycles were again obtained in good yields in high enantiomeric excess, thus facilitating the smooth synthesis of all macrocyclic enantiomers. This further highlights the potential of enantiospecific SuPhenEx reactions in the synthesis of sulfur-containing chiral compounds (Figure 5 and Supporting Information Table S2).

## Conclusions

We have demonstrated the facile asymmetric synthesis of a number of large chiral macrocycles using robust and enantiospecific SuFEx and SuPhenEx click reactions. This first asymmetric synthesis of macrocycles with chiral S atoms opens up the field for the synthesis of a wide range of chiral macrocycles. Since the latter have been shown to be of high relevance for both supramolecular and medicinal chemistry, we envisage a wide applicability of this easily formed rings, and our laboratories are currently further investigating several such options.

## Data Availability

The data underlying this study are available in the published article and its Supporting Information.

## Supporting Information Available

Synthetic procedures, HPLC analyses, CD and NMR spectra (PDF)
