# Supplementary material for: Synthesis of Large Macrocycles with Chiral Sulfur Centers via Enantiospecific SuFEx and SuPhenEx Click Reactions
Source: J Org Chem. 2023 Oct 30;88(22):15658–65. doi: 10.1021/acs.joc.3c01656 (PMC10660663; doi:10.1021/acs.joc.3c01656)
Supplement: Supplementary file 1 — jo3c01656_si_001.pdf [file jo3c01656_si_001.pdf]

Supporting Information to:

Synthesis of Large Macrocycles with Chiral Sulfur Centers via Enantiospecific SuFEx and SuPhenEx Click Reactions

*Yang Chao,[+] Muthusamy Subramaniam,[+] Kayambu Namitharan,[+] Yumei Zhu, Victor Koolma, Zitong Hao, Shikang Li, Yaxin Wang, Ilyos Hudoynazarov, Fedor M. Miloserdov, Han Zuilhof**

[a] Y. Chao, Y. Zhu, Z. Hao, S. Li, Y. Wang, H. Zuilhof, School of Pharmaceutical Science and Technology, Tianjin University, 92 Weijin Road, Tianjin 300072 (China)

[b] M. Subramaniam, K. Namitharan, F. M. Miloserdov, H. Zuilhof, Laboratory of Organic Chemistry, Wageningen University, Stippeneng 4, 6708WE Wageningen (The Netherlands)

[c] Dr. Ilyos Hudoynazarov. Division of Organic Synthesis and Applied Chemistry, National University of Uzbekistan, Tashkent 100174 (Uzbekistan)

*E-mail: Han.Zuilhof@wur.nl

Table of contents

1. **Materials and general methods** 3

2. **Synthesis of starting materials** 4

   2.1 Synthesis of disulfonimidoyl fluorides 4

   2.2 General procedure for the synthesis of diastereomeric macrocycles 7

   2.3 General procedure for the synthesis of enantiomeric chiral macrocycles 17

   2.4 Comparison of the syntheses of macrocycles using diphenol or diphenolate 18

   2.5 Synthesis of diastereomeric macrocycle on gram scale 32

3. **NMR spectra** 32

4. **HPLC data** 102

5. **ECD spectra** 129

6. **Chiral macrocycles based on SuPhenEx reaction** 131

   6.1 Synthesis of enantiomer di-*p*-nitrophenolate sulfonimidate 131

   6.2 Synthesis of chiral macrocycles by SuPhenEx reaction 135

      6.2.1 NMR spectra 136

      6.2.2 ECD spectra 138

      6.2.3 HPLC spectra 139

7. **X-Ray crystallography** 140

8. **References** 158

1. Materials and general methods

Starting materials, reagents, and solvents were purchased from commercial vendors and used as received, unless otherwise noted. All reactions were performed under an argon atmosphere and in dry solvents, unless otherwise stated. All the heating reactions were performed using an oil bath. Analytical thin-layer chromatography (TLC) was performed on aluminum sheets, precoated with silica gel GF254. Preparative thin-layer chromatography (PTLC) separations were carried out on 200 × 200 mm, 1.0 mm XINNUO silica gel plates (GF-254). Flash column chromatography was performed over silica gel (200−300 mesh or 300−400 mesh). ^{1}H, ^{13}C and ^{19}F NMR spectra were recorded on Bruker Avance 400 MHz spectrometers at 298 K, unless otherwise noted. The chemical shifts are listed in ppm on the δ scale and coupling constants were recorded in Hertz (Hz). Chemical shifts are calibrated relative to the signals corresponding of the non-deuterated solvents ($CHCl_3$: δ 7.26 ppm for ^{1}H and 77.16 ppm for ^{13}C, CH_2Cl_2: δ 5.32 ppm for ^{1}H and 53.84 ppm for ^{13}C, $(CH_3)_2SO$: δ 2.50 ppm for ^{1}H and 39.52 ppm for ^{13}C). The following abbreviations were used for multiplicities: s, singlet; d, doublet; t, triplet; m, multiplet or overlapping peaks; b, broad peaks. High-resolution mass spectra (HRMS) were measured on a Q Exactive HF spectrometer using electrospray ionization (ESI) in positive mode.

Enantioselectivity was monitored using an HPLC Chiralpak IA column or Chiralpak IE column. A reference, racemic product was synthesized to determine the column conditions for baseline separation. High-performance liquid chromatography (HPLC) analyses were operated using an Agilent 1260 liquid chromatography system (Agilent Technologies, USA). HPLC Grade isopropanol, methanol, dichloromethane and *n*-hexane were purchased from Concord Technology, P. R. The electronic circular dichroism (ECD) spectra were recorded on a MOS-500 spectrophotometer. All chiral compounds were dissolved in CH_2Cl_2 at 10 mg/L. The ECD spectra were recorded in the range 400 to 200 nm, a step size of 0.5 nm, a bandwidth of 2 nm, a step of 0.5 s, and an acquisition period of 0.5 s.

2. Synthesis of starting materials

2.1 Synthesis of disulfonimidoyl fluorides

Disulfonimidoyl fluorides **1a-e,** both racemic and chiral were synthesized according to our previously published method.[1]

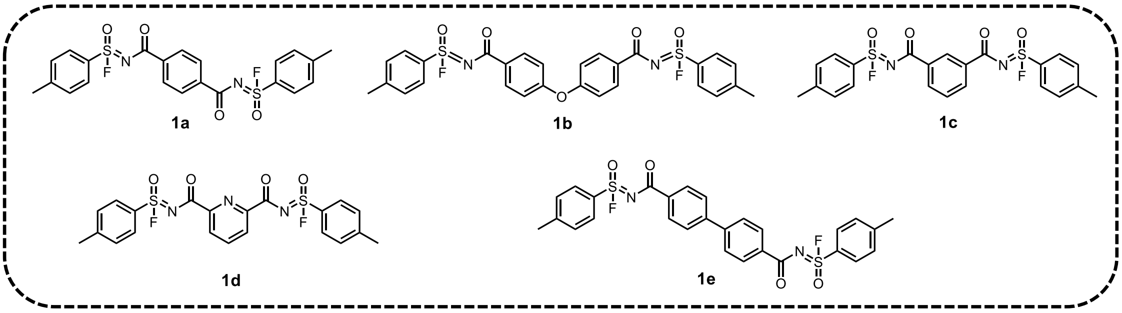

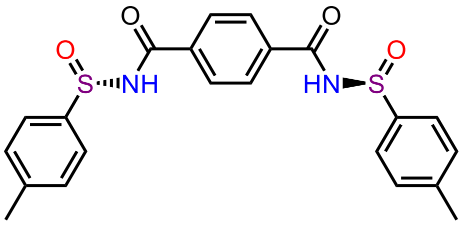

(*S*, *S*)-1a-NH

(*S*, *S*)-1a-NH: The title compound was obtained as a white solid (7.39 g, 16.8 mmol, 84%) ^{1}H NMR (400 MHz, DMSO-d_6) δ 11.73 (s, 2H), 8.00 (s, 4H), 7.69 (d, *J* = 8.1 Hz, 4H), 7.44 (d, *J* = 8.0 Hz, 4H), 2.41 (s, 6H). ^{13}C{1H} NMR (101 MHz, DMSO-d_6) δ 167.4 (s), 141.7 (s), 140.3 (s), 135.5 (s), 129.7 (s), 128.5 (s), 125.2 (s), 21.0 (s). HRMS (ESI) *m/z* [*M* + H]$^+$ Calcd for $C_{22}H_{21}N_2O_4S_2$ 441.0937, found 441.0944.

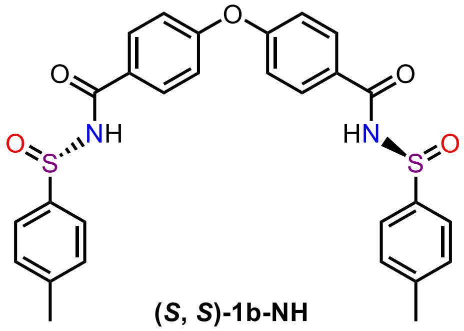

(*S*, *S*)-1b-NH

(*S*, *S*)-1b-NH: The title compound was obtained as a white solid (9.4 g, 17.7 mmol, 88%) ^{1}H NMR (400 MHz, DMSO-d_6) δ 11.52 (s, 2H), 8.02 – 7.95 (m, 4H), 7.66 (d, *J* = 8.2 Hz, 4H), 7.43 (d, *J* = 8.0 Hz, 4H), 7.19 – 7.13 (m, 4H), 2.41 (s, 6H). ^{13}C{1H} NMR (101 MHz, DMSO-d_6) δ 167.1 (s), 159.4 (s), 141.4 (s), 140.5 (s), 131.0 (s), 129.6 (s), 127.4 (s), 125.2 (s), 118.6 (s), 20.9 (s). HRMS (ESI) *m/z* [*M* + H]$^+$ Calcd for $C_{28}H_{25}N_2O_5S_2$ 533.1199, found 533.1205.

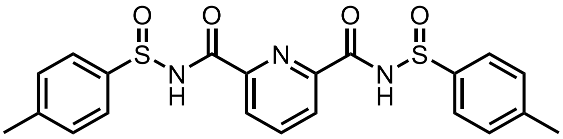

1d-NH

Diastereomeric 1d-NH : The title compound was obtained as a white solid (4.65 g, 10.5 mmol, 75%). ^{1}H NMR (400 MHz, $CDCl_3$) δ 10.9 (s, 1H), 8.3 (d, *J* = 7.8 Hz, 1H), 8.1 (t, *J* = 7.8 Hz, 1H), 7.3 (d, *J* = 7.9 Hz, 2H), 7.2 (d, *J* = 7.9 Hz, 2H), 2.3 (s, 3H). ^{13}C{1H} NMR (101 MHz, DMSO-d_6) δ 167.4 (s), 141.7 (s), 140.3 (s), 135.5 (s), 129.7 (s), 128.5 (s), 125.2 (s), 21.0 (s). HRMS (ESI) *m/z* [*M* + H]$^+$ Calcd for $C_{22}H_{21}N_2O_4S_2$ 441.0937, found 441.0944.

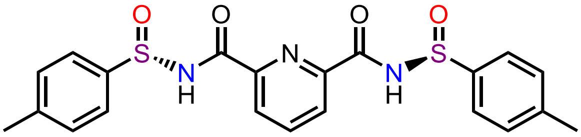

(*S*, *S*)-1d-NH

(*S*, *S*)-1d-NH: The title compound was obtained as a white solid (2.24 g, 5.1 mmol, 68%). ^{1}H NMR (400 MHz, $CDCl_3$) δ 10.8 (s, 1H), 8.5 – 8.2 (m, 1H), 8.2 – 8.0 (m, 1H), 7.4 (d, *J* = 7.9 Hz, 2H), 7.3 – 7.3 (m, 3H), 7.2 – 7.1 (m, 2H), 2.4 (s, 3H). ^{13}C{1H} NMR (101 MHz, DMSO-d_6) δ 167.4 (s), 141.7 (s), 140.3 (s), 135.5 (s), 129.7 (s), 128.5 (s), 125.2 (s), 21.0 (s). HRMS (ESI) *m/z* [*M* + H]$^+$ Calcd for $C_{22}H_{21}N_2O_4S_2$ 441.0937, found 441.0944.

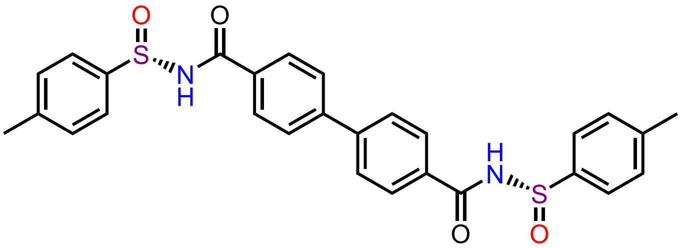

(*S*, *S*)-1e-NH

(*S*, *S*)-1e-NH: The title compound was obtained as a white solid (8.2 g, 15.9 mmol, 80%) ^{1}H NMR (400 MHz, DMSO-d_6) δ 11.66 (s, 2H), 8.05 – 7.99 (m, 4H), 7.92 – 7.86 (m, 4H), 7.71 – 7.65 (m, 4H), 7.45 (d, *J* = 8.0 Hz, 4H), 2.41 (s, 6H). ^{13}C{1H} NMR (101 MHz, DMSO-d_6) δ 167.7 (s), 142.9 (s), 141.5 (s), 140.5 (s), 131.5 (s), 129.7 (s), 129.2 (s), 127.1 (s), 125.3 (s), 21.0 (s). HRMS (ESI) *m/z* [*M* + H]$^+$ Calcd for $C_{28}H_{25}N_2O_4S_2$ 517.1250, found 517.1256.

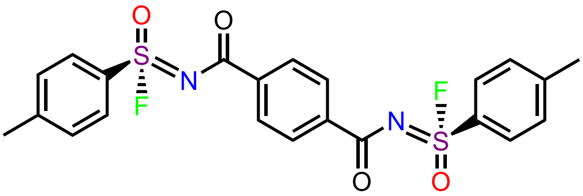

(*R, R*)-1a

Disulfonimidoyl fluoride **(*R, R*)-1a:**

The title compound was purified by silica gel column chromatography (EtOAc/*n*-hexane = 1:4 to CH_2Cl_2/ EtOAc = 1:1) to afford **(*R, R*)-1a** as white solid (7.7 g, 16.2 mmol, 96%, >99 % *ee, dr* = 98:1), R_f = 0.33 (EtOAc/*n*-hexane = 1:4).^{1}H NMR (400 MHz, $CDCl_3$) δ 8.20 (s, 4H), 8.07 (d, *J* = 8.5 Hz, 4H), 7.47 (d, *J* = 8.4 Hz, 4H), 2.52 (s, 6H). ^{13}C{1H} NMR (101 MHz, $CDCl_3$) δ 169.6 (s), 147.6 (s), 138.0 (s), 131.4 (d, *J* = 20 Hz), 130.5 (s), 130.0 (s), 128.2 (s), 22.0 (s). ^{19}F NMR (376 MHz, $CDCl_3$) δ 64.81 (s). HRMS (ESI) *m/z* $[M + H]^+$ Calcd for $C_{22}H_{19}F_2N_2O_4S_2$ 477.0749, found 477.0746.

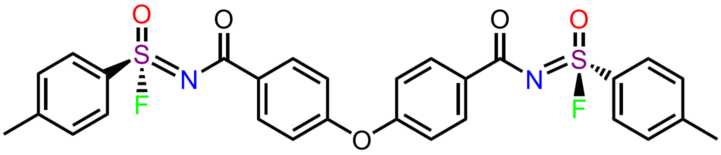

(*R, R*)-1b

Disulfonimidoyl fluoride **(*R, R*)-1b:**

The title compound was purified by silica gel column chromatography (EtOAc/*n*-hexane = 1:4 to CH_2Cl_2/ EtOAc = 1:1) to afford **(*R, R*)-1c** as white solid (4.81g, 8.5 mmol, 85%, >99% *ee, dr* = 82:1), R_f = 0.17 (EtOAc/*n*-hexane = 1:4).^{1}H NMR (400 MHz, $CDCl_3$) δ 8.19 – 8.13 (m, 4H), 8.06 (d, *J* = 8.4 Hz, 4H), 7.45 (d, *J* = 8.3 Hz, 4H), 7.10 – 7.03 (m, 4H), 2.51 (s, 6H). ^{13}C{1H} NMR (101 MHz, $CDCl_3$) δ 169.3 (s), 160.6 (s), 147.4 (s), 132.5 (s), 131.7 (d, *J* = 20 Hz), 130.4 (s), 130.0 (s), 128.1 (s), 118.7 (s), 22.0 (s).^{19}F NMR (376 MHz, $CDCl_3$) δ 65.31 (s). HRMS (ESI) *m/z* $[M + H]^+$ Calcd for $C_{28}H_{23}F_2N_2O_5S_2$ 569.1011, found 569.1020.

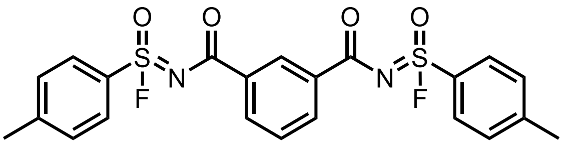

1c

Disulfonimidoyl fluoride **diastereomeric 1c:**

The title compound was obtained as a white solid (2.5 g, 5.6 mmol, 65%).^{1}H NMR (400 MHz, $CDCl_3$) δ 8.71 (t, *J* = 1.8 Hz, 1H), 8.39 (dd, *J* = 7.7, 1.8 Hz, 2H), 8.25 – 8.19 (m, 4H), 7.75 (t, *J* = 7.8 Hz, 1H), 7.63 (dd, *J* = 8.4, 2.9 Hz, 4H). ^{13}C{1H} NMR (101 MHz, $CDCl_3$) δ 169.42, 147.38, 134.51, 134.32, 131.52, 131.51, 131.22, 131.02, 130.37, 128.57, 128.06, 21.90. HRMS (ESI) *m/z* $[M + H]^+$ Calcd for $C_{22}H_{18}F_2N_2O_4S_2$ 477.0749, found 477.0785.

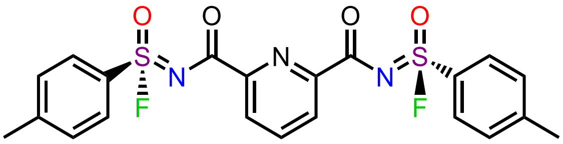

(*R*, *R*)-1d

Disulfonimidoyl fluoride **(*R*, *R*)-1d:**

The title compound was obtained as a white solid (1.3 g, 2.9 mmol, 67%, 99% *ee*).^{1}H NMR (400 MHz, $CDCl_3$) δ 8.3 (d, *J* = 7.7 Hz, 2H), 8.1 – 8.0 (m, 4H), 7.9 (t, *J* = 7.8 Hz, 1H), 7.3 (d, *J* = 8.2 Hz, 4H), 2.4 (s, 6H). ^{13}C{1H} NMR (101 MHz, $CDCl_3$) δ 144.6, 142.0, 140.5, 136.5, 130.2, 129.4, 127.6, 124.6, 21.7, 21.5. HRMS (ESI) *m/z* $[M + H]^+$ Calcd for $C_{21}H_{17}F_2N_3O_4S_2$ 478.0701, found 478.0756.

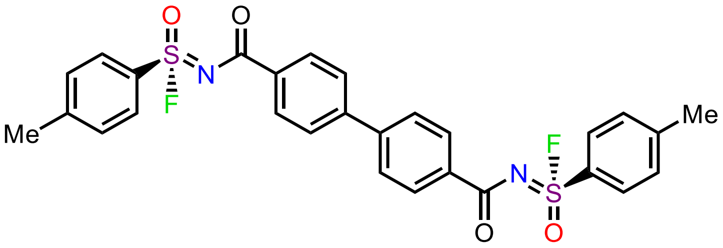

(*R*, *R*)-1e

Disulfonimidoyl fluoride **(*R*, *R*)-1e:**

The title compound was purified by silica gel column chromatography (CH_2Cl_2/*n*-hexane = 3:2 to CH_2Cl_2/ EtOAc = 1:1) to afford **(*R*, *R*)-1b** as white solid (3.54 g, 6.41 mmol, 73%, 96% *ee*), R_f = 0.21 (CH_2Cl_2/*n*-hexane = 3:2).^{1}H NMR (400 MHz, $CDCl_3$) δ 8.26 – 8.21 (m, 4H), 8.08 (d, *J* = 8.5 Hz, 4H), 7.72 – 7.67 (m, 4H), 7.47 (d, *J* = 8.3 Hz, 4H), 2.52 (s, 6H). ^{13}C{1H} NMR (101 MHz, $CDCl_3$) δ 169.9 (s), 147.4 (s), 144.8 (s), 133.8 (s), 131.6 (d, *J* = 20 Hz), 130.7 (s), 130.5 (s), 128.2 (s), 127.4 (s), 22.0 (s). ^{19}F NMR (376 MHz, $CDCl_3$) δ 65.23 (s). HRMS (ESI) *m/z* $[M + H]^+$ Calcd for $C_{28}H_{23}F_2N_2O_4S_2$ 553.1062, found 553.1071.

2.2 General procedure for the synthesis of diastereomeric macrocycles

To a seal tube (50 mL) with a magnetic stirring bar, was added di-sulfinimidoyl fluride compounds **1** (0.2 mmol, 1.0 equiv.) and 1.0 equiv of diphenols **2**. Next 10 mL of anhydrous acetonitrile was added into the seal tube. Finally, 2.1 equiv of DBU was added. The reaction mixture was allowed to stir for 3 h at 50 ºC. Then the reaction was quenched with 20 mL water. The solution was extracted with CH_2Cl_2 (3 × 200 mL), dried with anhydrous Na_2SO_4 and concentrated by reduced pressure. The resulting residual was purified by silica gel column chromatography (*n*-hexane/EtOAc = 2:1 to 1:1) to afford the product **3**.

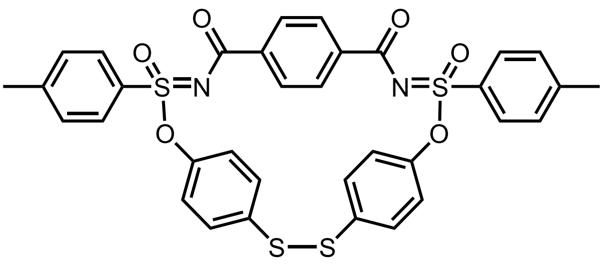

3a

3a was obtained as a white solid (132 mg, 0.19 mmol, 96%) ^{1}H NMR (400 MHz, $CDCl_3$) δ 7.92 (d, *J* = 8.0 Hz, 2H), 7.83 (d, *J* = 8.0 Hz, 2H), 7.66 (s, 2H), 7.58 – 7.45 (m, 4H), 7.40 – 7.21 (m, 8H), 6.93 (d, *J* = 8.3 Hz, 2H), 2.43 (s, 6H). ^{13}C{1H} NMR (101 MHz, $CDCl_3$) δ 146.8, 145.7, 135.3, 133.5, 133.3, 132.5, 132.1, 129.9, 129.8, 129.7, 128.4, 128.1, 128.0, 127.8, 126.9, 126.5, 121.5, 120.7, 21.8. HRMS (ESI) *m/z* $[M + H]^+$ Calcd for $C_{34}H_{27}N_2O_6S_4$ 687.0746, found 687.0714.

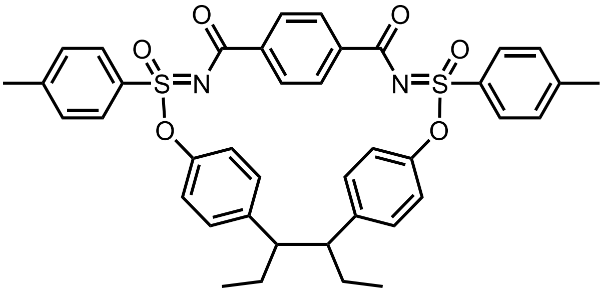

3b

3b was obtained as a white solid (86 mg, 0.12 mmol, 61%) ^{1}H NMR (400 MHz, $CDCl_3$) δ 8.0 (ttd, *J* = 5.2, 3.9, 3.1, 1.5 Hz, 4H), 7.9 (dddd, *J* = 11.9, 8.3, 6.9, 3.6 Hz, 4H), 7.3 – 7.2 (m, 4H), 7.0 (dddt, *J* = 10.6, 7.2, 4.0, 1.6 Hz, 4H), 6.9 – 6.8 (m, 4H), 2.5 – 2.4 (m, 8H), 1.3 – 1.1 (m, 4H), 0.4 (tdd, *J* = 7.5, 4.7, 2.1 Hz, 6H). ^{13}C{1H} NMR (101 MHz, $CDCl_3$) δ 171.1, 147.5, 145.8, 143.3, 138.5, 133.2, 129.9, 129.4, 129.3, 128.2, 122.5, 53.5, 21.8, 12.1. HRMS (ESI) *m/z* $[M + H]^+$ Calcd for $C_{40}H_{39}N_2O_6S_2$ 707.2244, found 707.2261.

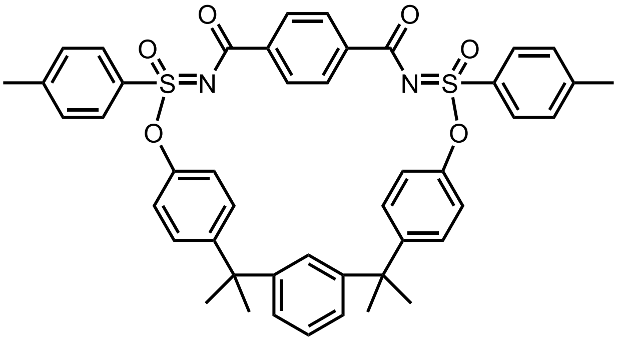

3c

3c was obtained as a white solid (116 mg, 0.15 mmol, 74%) ^{1}H NMR (400 MHz, $CDCl_3$) δ 7.96 (t, *J* = 7.3 Hz, 4H), 7.79 (d, *J* = 2.9 Hz, 4H), 7.40 (d, *J* = 8.0 Hz, 4H), 7.22 (t, *J* = 8.0 Hz, 1H), 7.08 (d, *J* = 8.4 Hz, 6H), 6.96 (t, *J* = 9.0 Hz, 4H), 6.85 (dd, *J* = 33.7, 1.9 Hz, 1H), 2.50 (s, 6H), 1.56 (t, *J* = 3.5 Hz, 12H). ^{13}C{1H} NMR (101 MHz, $CDCl_3$) δ

170.6, 170.5, 150.8, 150.7, 149.4, 149.4, 147.1, 147.0, 145.8, 138.5, 138.4, 133.4, 133.4, 130.0, 129.9, 129.1, 129.1, 128.3, 128.3, 128.0, 128.0, 125.2, 125.2, 124.7, 124.7, 122.6, 122.5, 60.4, 43.2, 43.2, 31.1, 31.0, 31.0, 21.8. HRMS (ESI) *m/z* [*M* + H]$^+$ Calcd for $C_{46}H_{43}N_2O_6S_2$ 783.2557, found 783.2534.

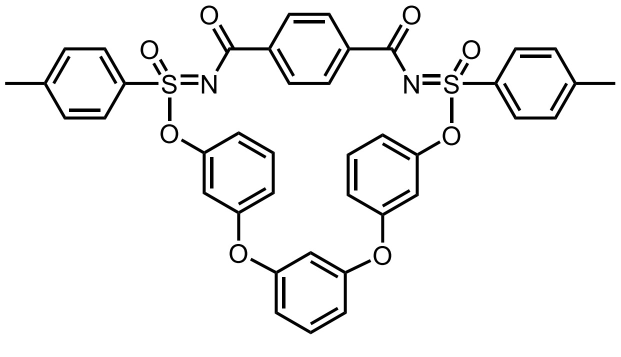

3d

3d was obtained as a white solid (98 mg, 0.13 mmol, 67%) ^{1}H NMR (400 MHz, $CDCl_3$) δ 8.0 (dd, *J* = 8.3, 6.6 Hz, 4H), 7.8 (d, *J* = 7.5 Hz, 4H), 7.4 (dd, *J* = 8.4, 3.3 Hz, 5H), 7.2 (d, *J* = 9.0 Hz, 2H), 7.1 – 7.0 (m, 3H), 7.0 – 7.0 (m, 2H), 6.9 – 6.8 (m, 4H), 6.6 (s, 1H), 2.5 (s, 6H). ^{13}C{1H} NMR (101 MHz, $CDCl_3$) δ 170.8, 157.0, 157.0, 157.0, 156.9, 146.0, 146.0, 144.4, 144.0, 138.4, 138.4, 133.5, 133.4, 131.5, 130.1, 130.1, 129.0, 129.0, 128.3, 128.3, 124.3, 124.2, 117.7, 117.7, 117.7, 117.6, 112.1, 112.0, 21.8. HRMS (ESI) *m/z* [*M* + H]$^+$ Calcd for $C_{40}H_{31}N_2O_8S_2$ 731.1516, found 731.1574.

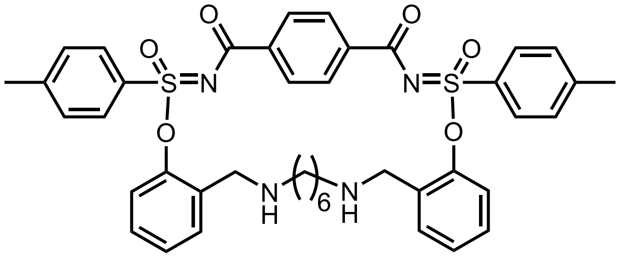

3e

3e was obtained as a white solid (140 mg, 0.18 mmol, 91%) ^{1}H NMR (400 MHz, $CDCl_3$) δ 8.02 (dd, *J* = 8.6, 1.8 Hz, 8H), 7.48 (dt, *J* = 7.5, 1.5 Hz, 2H), 7.42 (dd, *J* = 8.5, 2.7 Hz, 4H), 7.28 (d, *J* = 7.3 Hz, 2H), 7.21 (td, *J* = 7.7, 1.9 Hz, 2H), 7.12 (d, *J* = 8.0 Hz, 2H), 6.97 – 6.92 (m, 4H), 4.03 – 3.84 (m, 4H), 2.73 – 2.58 (m, 4H), 2.49 (d, *J* = 2.0 Hz, 6H), 1.46 (t, *J* = 7.5 Hz, 4H), 1.35 – 1.21 (m, 8H).^{13}C{1H} NMR (101 MHz, $CDCl_3$) δ 171.0, 171.0, 160.4, 147.7, 147.7, 145.9, 145.9, 134.2, 134.1, 134.0, 131.8, 130.7, 130.7, 130.6, 130.2, 130.2, 128.3, 128.2, 128.1, 128.1, 127.6, 127.5, 122.2, 122.1, 118.5, 50.2, 50.1, 48.8, 48.7, 30.5, 30.5, 27.8, 27.8, 21.8. HRMS (ESI) *m/z* [*M* + H]$^+$ Calcd for $C_{42}H_{45}N_4O_6S_2$ 765.2775, found 765.2725.

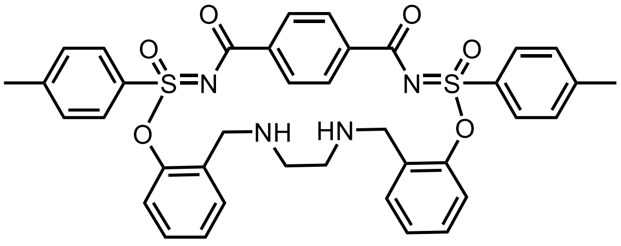

3f

3f was obtained as a white solid (123 mg, 0.17 mmol, 87%) ^{1}H NMR (400 MHz, $CDCl_3$) δ 8.04 (d, *J* = 8.0 Hz, 4H), 7.89 (d, *J* = 11.3 Hz, 4H), 7.71 (d, *J* = 7.5 Hz, 2H), 7.55 – 7.16 (m, 10H), 4.30 (d, *J* = 14.4 Hz, 2H), 3.87 (d, *J* = 14.5 Hz, 2H), 3.00 – 2.67 (m, 4H), 2.52 (s, 6H). ^{13}C{1H} NMR (101 MHz, $CDCl_3$) δ 171.0, 146.6, 146.1, 138.7, 134.0, 133.5, 130.3, 129.9, 129.8, 129.1, 128.3, 128.1, 128.1, 128.1, 127.9, 127.9, 123.1, 123.1, 50.1, 48.1, 21.8. HRMS (ESI) *m/z* [*M* + H]$^+$ Calcd for $C_{38}H_{37}N_4O_6S_2$ 709.2149, found 709.2134.

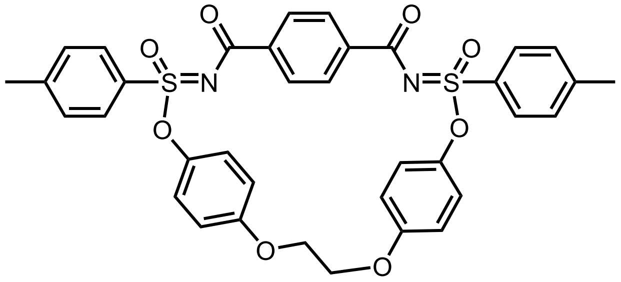

3g

3g was obtained as a white solid (108 mg, 0.16 mmol, 79%) ^{1}H NMR (400 MHz, $CDCl_3$) δ 7.99 (s, 4H), 7.83 (t, *J* = 8.3 Hz, 4H), 7.31 (d, *J* = 8.0 Hz, 4H), 7.18 – 7.00 (m, 4H), 6.81 (td, *J* = 8.9, 8.3, 2.0 Hz, 2H), 6.46 – 6.23 (m, 2H), 4.15 (dt, *J* = 12.7, 8.2 Hz, 4H), 2.41 (d, *J* = 3.6 Hz, 6H). ^{13}C{1H} NMR (101 MHz, $CDCl_3$) δ 171.0, 170.9, 159.4, 150.1, 149.7, 146.1, 146.0, 138.5, 132.9, 132.6, 130.0, 130.0, 129.9, 129.4, 128.5, 128.4, 115.5, 115.2, 112.4, 112.3, 112.2, 112.1, 66.5, 21.8. HRMS (ESI) *m/z* [*M* + H]$^+$ Calcd for $C_{36}H_{31}N_2O_8S_2$ 683.1516, found 683.1528.

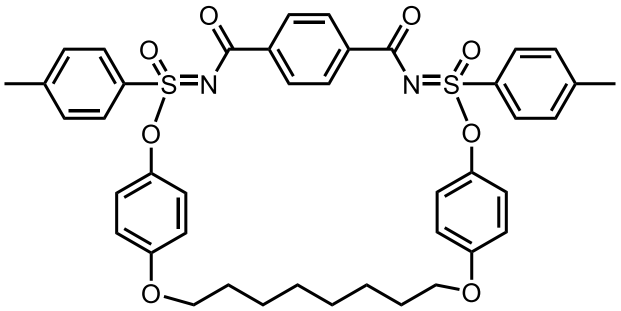

3h

3h was obtained as a white solid (95 mg, 0.12 mmol, 62%) ^{1}H NMR (400 MHz, $CDCl_3$) δ 8.02 (s, 2H), 7.99 (d, *J* = 3.5 Hz, 2H), 7.98 – 7.92 (m, 2H), 7.88 (dd, *J* = 8.4, 2.0 Hz, 2H), 7.32 (td, *J* = 8.8, 2.2 Hz, 4H), 6.99 – 6.92 (m, 4H), 6.69 (td, *J* = 9.0, 2.2 Hz, 4H), 3.82 (dt, *J* = 10.5, 6.0 Hz, 6H), 2.41 (d, *J* = 6.4 Hz, 6H), 1.70 – 1.62 (m, 6H), 1.33 (m, 4H). ^{13}C{1H} NMR (101 MHz, $CDCl_3$) δ 170.2, 170.0, 157.1, 157.1, 157.1, 152.1,

144.7, 141.2, 141.2, 141.0, 137.5, 137.4, 132.2, 132.1, 132.1, 128.9, 128.9, 128.9, 128.9, 128.3, 128.3, 128.3, 127.3, 127.3, 127.3, 122.7, 122.6, 122.6, 114.5, 114.1, 114.1, 114.0, 67.4, 67.2, 67.0, 67.0, 27.9, 27.9, 27.8, 24.6, 24.6, 24.6, 20.8, 20.7. HRMS (ESI) *m/z* $[M + H]^+$ Calcd for $C_{42}H_{43}N_2O_8S_2$ 767.2455, found 767.2483.

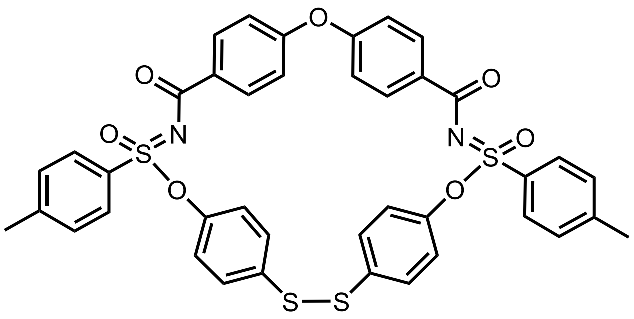

3i

3i was obtained as a white solid (136 mg, 0.17 mmol, 91%) ^{1}H NMR (400 MHz, $CDCl_3$) δ 8.0 (dq, J = 9.0, 2.4 Hz, 4H), 7.9 (ddd, J = 8.5, 3.7, 1.9 Hz, 4H), 7.4 – 7.2 (m, 8H), 7.0 (ddd, J = 8.7, 3.4, 1.6 Hz, 4H), 6.9 (dd, J = 8.6, 3.9 Hz, 4H), 2.4 (d, J = 3.0 Hz, 6H). ^{13}C{1H} NMR (101 MHz, $CDCl_3$) δ 170.7, 160.1, 148.6, 146.0, 145.9, 135.8, 133.2, 131.9, 130.6, 130.1, 130.0, 129.2, 128.2, 123.7, 118.4, 21.8. HRMS (ESI) *m/z* $[M + H]^+$ Calcd for $C_{40}H_{31}N_2O_7S_4$ 779.0696, found 779.0627.

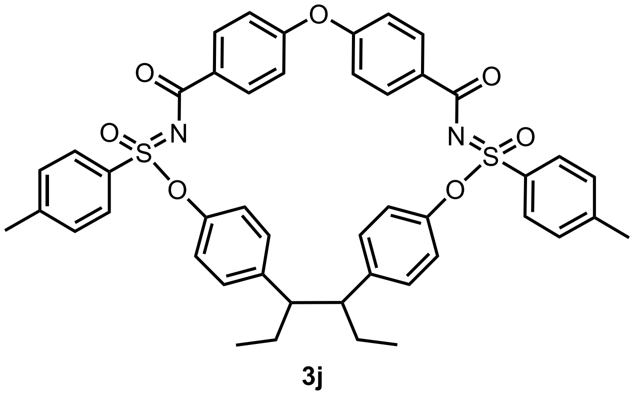

3j

3j was obtained as a white solid (135 mg, 0.17 mmol, 88%) ^{1}H NMR (400 MHz, $CDCl_3$) δ 8.10 (d, J = 8.4 Hz, 2H), 7.96 (d, J = 8.4 Hz, 2H), 7.78 (dd, J = 13.7, 8.7 Hz, 4H), 7.51 – 7.42 (m, 4H), 7.33 (d, J = 8.5 Hz, 2H), 7.23 (d, J = 8.6 Hz, 2H), 7.12 (d, J = 8.6 Hz, 2H), 6.97 (d, J = 8.5 Hz, 2H), 6.82 (d, J = 8.7 Hz, 2H), 6.75 (d, J = 8.8 Hz, 2H), 2.53 (d, J = 4.4 Hz, 6H), 1.38 – 0.99 (m, 4H), 0.98 – 0.51 (m, 2H), 0.45 (t, J = 7.3 Hz, 3H), 0.28 (t, J = 7.3 Hz, 3H). ^{13}C{1H} NMR (101 MHz, $CDCl_3$) δ 169.9, 169.9, 160.6, 160.2, 148.7, 147.0, 145.8, 145.7, 144.4, 144.1, 134.4, 134.2, 131.7, 131.7, 131.0, 130.7, 130.1, 129.3, 128.2, 128.1, 128.1, 123.5, 122.7, 119.1, 118.0, 53.7, 53.3, 28.9, 28.5, 21.8, 12.6, 12.3. HRMS (ESI) *m/z* $[M + H]^+$ Calcd for $C_{46}H_{43}N_2O_7S_2$ 799.2193, found 799.2135.

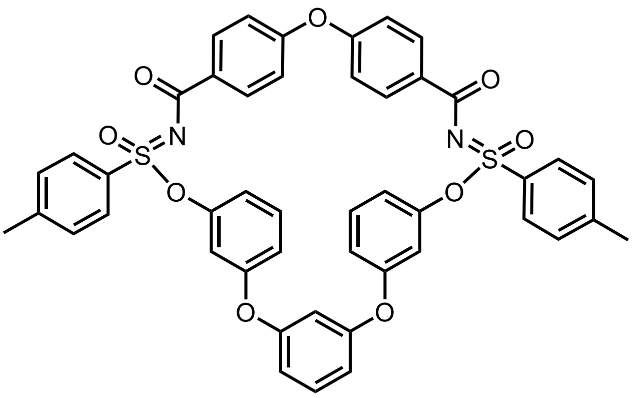

3k

3k was obtained as a white solid (146 mg, 0.18 mmol, 88%) ^{1}H NMR (400 MHz, $CDCl_3$) δ 8.0 – 7.8 (m, 8H), 7.3 (ddd, *J* = 7.7, 3.7, 2.6 Hz, 4H), 7.2 – 7.1 (m, 5H), 6.9 (dd, *J* = 9.2, 2.9 Hz, 4H), 6.8 (dd, *J* = 8.8, 2.7 Hz, 4H), 6.7 (d, *J* = 2.1 Hz, 1H), 6.6 – 6.5 (m, 2H), 2.4 (s, 6H). ^{13}C{1H} NMR (101 MHz, $CDCl_3$) δ 170.6, 160.4, 158.1, 155.8, 145.9, 144.7, 133.6, 131.8, 130.8, 130.6, 130.1, 130.1, 128.3, 124.4, 124.3, 119.7, 118.6, 114.0, 113.9, 110.1, 21.8. HRMS (ESI) *m/z* [*M* + H]$^+$ Calcd for $C_{46}H_{35}N_2O_9S_2$ 823.1465, found 823.1448.

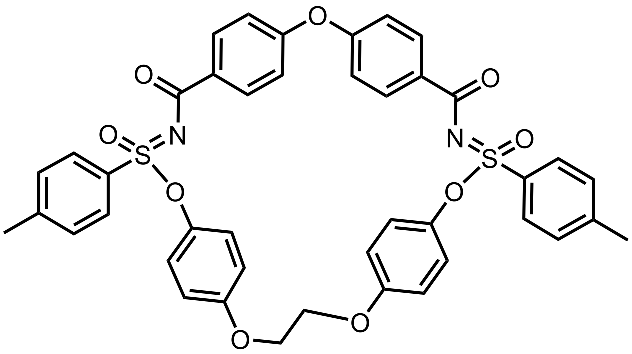

3l

3l was obtained as a white solid (103 mg, 0.13 mmol, 67%) ^{1}H NMR (400 MHz, $CDCl_3$) δ 8.1 – 8.0 (m, 4H), 7.9 – 7.8 (m, 4H), 7.3 (ddt, *J* = 7.1, 1.8, 0.8 Hz, 4H), 7.1 (td, *J* = 8.3, 6.5 Hz, 2H), 7.0 (td, *J* = 2.3, 1.2 Hz, 2H), 6.9 – 6.8 (m, 6H), 6.4 (dddd, *J* = 25.7, 8.1, 2.1, 0.9 Hz, 2H), 4.2 – 4.0 (m, 4H), 2.4 (s, 6H). ^{13}C{1H} NMR (101 MHz, $CDCl_3$) δ 170.6, 160.9, 159.4, 150.0, 149.8, 146.0, 145.9, 133.0, 132.9, 131.8, 131.0, 130.0, 130.0, 128.4, 128.3, 118.8, 115.7, 115.6, 115.4, 115.4, 109.3, 109.3, 66.7, 66.7, 21.8. HRMS (ESI) *m/z* [*M* + H]$^+$ Calcd for $C_{42}H_{35}N_2O_9S_2$ 775.1465, found 775.1427.

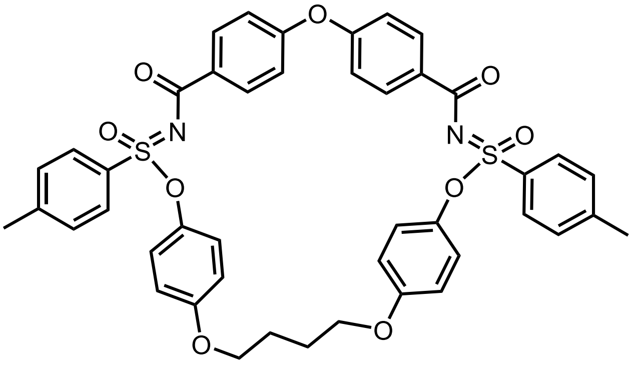

3m

3m was obtained as a white solid (120 mg, 0.15 mmol, 78%) ^{1}H NMR (400 MHz, $CDCl_3$) δ 8.0 – 7.8 (m, 8H), 7.3 (dd, *J* = 8.2, 5.4 Hz, 4H), 7.0 (dd, *J* = 22.0, 8.9 Hz, 4H), 6.9 (dd, *J* = 8.6, 4.3 Hz, 4H), 6.8 – 6.7 (m, 4H), 3.9 (s, 4H), 2.4 (s, 6H), 1.9 (d, *J* = 3.0 Hz, 4H). ^{13}C{1H} NMR (101 MHz, $CDCl_3$) δ 170.9, 160.4, 157.9, 157.8, 145.8, 145.7, 142.6, 142.4, 133.4, 133.3, 131.7, 130.9, 130.9, 130.0, 130.0, 128.4, 128.3, 124.0, 123.9, 118.6, 115.0, 114.9, 67.0, 25.6, 25.1, 21.8. HRMS (ESI) *m/z* [*M* + H]$^+$ Calcd for $C_{44}H_{39}N_2O_9S_2$ 803.1778, found 803.1729.

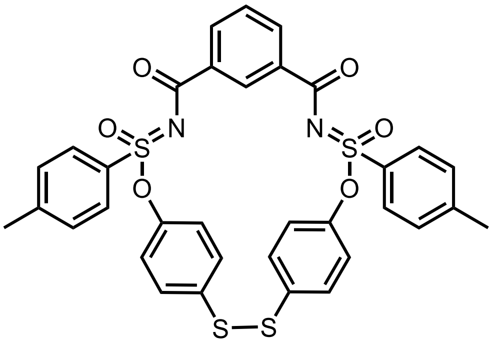

3n

3n was obtained as a white solid (95 mg, 0.14 mmol, 72%) ^{1}H NMR (400 MHz, $CDCl_3$) δ 8.3 (dt, *J* = 3.3, 1.8 Hz, 1H), 8.2 (dd, *J* = 7.8, 1.7 Hz, 1H), 8.1 (dd, *J* = 7.7, 1.8 Hz, 1H), 8.0 – 8.0 (m, 2H), 7.9 – 7.8 (m, 2H), 7.4 – 7.3 (m, 6H), 7.3 – 7.2 (m, 3H), 7.1 – 7.1 (m, 2H), 7.0 – 6.9 (m, 2H), 2.4 (d, *J* = 5.6 Hz, 6H). ^{13}C{1H} NMR (101 MHz, $CDCl_3$) δ 170.6, 170.2, 148.7, 148.0, 146.1, 145.9, 135.2, 135.2, 135.1, 133.5, 133.4, 133.1, 130.9, 130.3, 130.1, 130.0, 128.8, 128.4, 128.3, 128.2, 128.1, 128.1, 123.9, 123.6, 21.8. HRMS (ESI) *m/z* [*M* + H]$^+$ Calcd for $C_{34}H_{27}N_2O_6S_4$ 687.0433, found 687.0457.

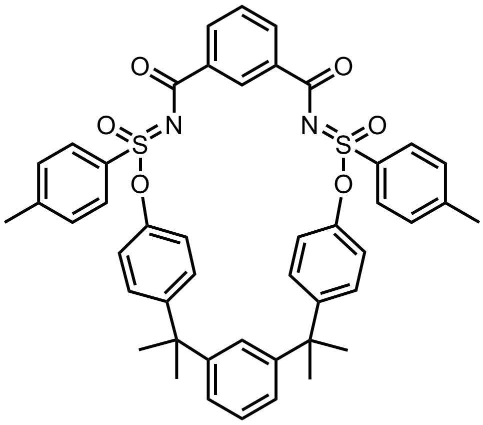

3o

3o was obtained as a white solid (105 mg, 0.13 mmol, 70%) ^{1}H NMR (400 MHz, $CDCl_3$) δ 8.82 (dt, *J* = 3.9, 1.9 Hz, 1H), 8.23 – 8.13 (m, 2H), 7.86 (dtd, *J* = 8.7, 4.2, 1.8 Hz, 4H), 7.39 – 7.29 (m, 1H), 7.23 (tt, *J* = 6.3, 2.8 Hz, 4H), 7.04 (dtd, *J* = 7.7, 5.1, 2.9 Hz, 1H), 6.95 (td, *J* = 5.3, 4.8, 2.3 Hz, 4H), 6.89 (tt, *J* = 5.6, 2.8 Hz, 6H), 6.74 – 6.64 (m, 1H), 2.38 – 2.29 (m, 6H), 1.42 (t, *J* = 2.2 Hz, 12H).^{13}C{1H} NMR (101 MHz, $CDCl_3$) δ 170.80, 150.44, 148.87, 147.06, 145.56, 135.14, 133.45, 133.16, 130.90, 129.93, 129.87, 128.23, 128.18, 128.13, 127.73, 125.88, 123.97, 122.01, 121.87, 42.56, 30.48, 29.95, 21.73. HRMS (ESI) *m/z* [*M* + H]$^+$ Calcd for $C_{46}H_{43}N_2O_6S_2$ 783.2557, found 783.2563.

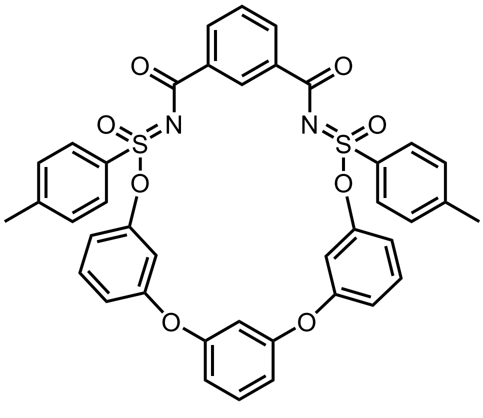

3p

3p was obtained as a white solid (100 mg, 0.14 mmol, 69%) ^{1}H NMR (400 MHz, $CDCl_3$) δ 8.8 (p, *J* = 1.8 Hz, 1H), 8.2 (dt, *J* = 8.4, 2.3 Hz, 2H), 8.0 – 7.8 (m, 4H), 7.4 (td, *J* = 7.7, 3.3 Hz, 1H), 7.3 (dd, *J* = 10.4, 8.3 Hz, 4H), 7.2 – 7.1 (m, 1H), 7.1 – 7.0 (m, 4H), 6.9 – 6.8 (m, 4H), 6.6 (dtd, *J* = 8.0, 5.1, 2.3 Hz, 2H), 6.5 (tdd, *J* = 6.1, 4.0, 2.4 Hz, 1H), 2.4 (d, *J* = 5.2 Hz, 6H). ^{13}C{1H} NMR (101 MHz, $CDCl_3$) δ 171.1, 171.1, 158.0, 157.9, 155.7, 145.9, 145.9, 144.5, 144.4, 135.3, 133.5, 133.0, 131.1, 130.8, 130.0, 130.0, 128.3, 128.3, 128.1, 124.3, 119.7, 119.6, 119.6, 114.2, 114.2, 114.1, 110.0, 109.9, 21.8, 21.8. HRMS (ESI) *m/z* [*M* + H]$^+$ Calcd for $C_{40}H_{31}N_2O_8S_2$ 731.1516, found 731.1542.

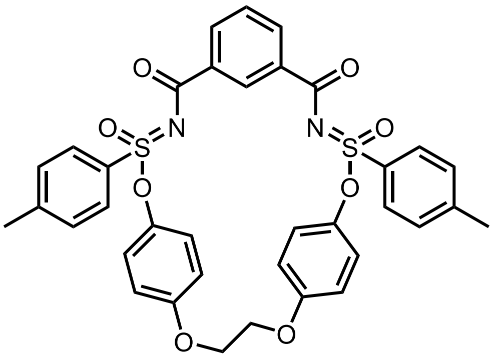

3q

3q was obtained as a white solid (87 mg, 0.13 mmol, 64%) ^{1}H NMR (400 MHz, $CDCl_3$) δ 8.8 (dt, *J* = 8.4, 1.8 Hz, 1H), 8.1 (dt, *J* = 7.7, 1.8 Hz, 2H), 8.0 – 7.9 (m, 4H), 7.4 – 7.3 (m, 5H), 7.1 – 7.0 (m, 4H), 6.8 (dddd, *J* = 8.3, 7.1, 2.5, 0.9 Hz, 2H), 6.5 (ddd, *J* = 8.2, 2.2, 0.9 Hz, 1H), 6.3 (ddd, *J* = 8.2, 2.2, 0.9 Hz, 1H), 4.2 – 4.0 (m, 4H), 2.4 (d, *J* = 8.1 Hz, 6H). ^{13}C{1H} NMR (101 MHz, $CDCl_3$) δ 171.0, 171.0, 159.3, 150.2, 149.9, 146.0, 145.8, 135.3, 135.3, 133.7, 133.6, 133.5, 133.2, 130.4, 130.4, 130.0, 130.0, 130.0, 129.9, 128.3, 128.2, 115.6, 115.6, 115.1, 114.9, 109.2, 109.0, 66.5, 66.4, 21.8, 21.8. HRMS (ESI) *m/z* $[M + H]^+$ Calcd for $C_{36}H_{31}N_2O_8S_2$ 683.1516, found 683.1524.

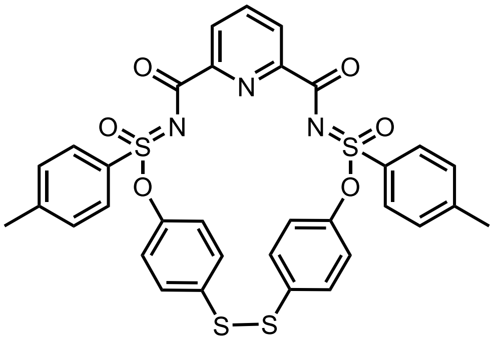

3r

3r was obtained as a white solid (105 mg, 0.15 mmol, 91%) ^{1}H NMR (400 MHz, $CDCl_3$) δ 8.2 (d, *J* = 7.7 Hz, 2H), 8.1 – 8.0 (m, 4H), 7.8 (t, *J* = 7.8 Hz, 1H), 7.4 (d, *J* = 8.3 Hz, 4H), 7.3 – 7.3 (m, 4H), 7.1 – 7.0 (m, 4H), 2.4 (s, 6H). ^{13}C{1H} NMR (101 MHz, $CDCl_3$) δ 169.9, 169.2, 151.9, 151.6, 149.2, 148.2, 146.0, 145.9, 137.5, 135.4, 135.1, 132.9, 132.7, 130.5, 130.0, 130.0, 129.8, 128.6, 128.5, 127.1, 126.5, 123.7, 123.5, 21.9, 21.8. HRMS (ESI) *m/z* $[M + H]^+$ Calcd for $C_{33}H_{26}N_3O_6S_4$ 688.0386, found 688.0365.

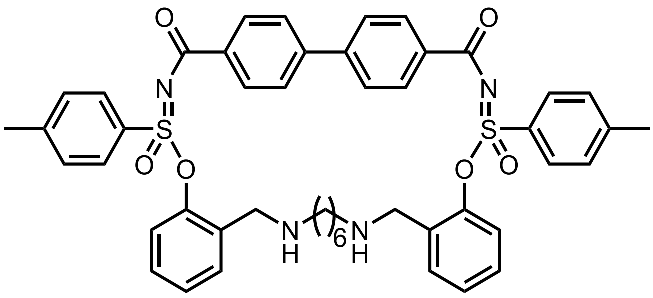

3s

3s was obtained as a white solid (115 mg, 0.14 mmol, 80%) ^{1}H NMR (400 MHz, $CDCl_3$) δ 7.98 (s, 4H), 7.88 (s, 4H), 7.48 (s, 6H), 7.36 (s, 4H), 7.22 (s, 6H), 4.06 (s, 2H), 3.71 (s, 2H), 2.53 – 2.43 (m, 2H), 2.41 (s, 6H), 1.51 (s, 2H), 1.16 (s, 4H), 0.99 (s, 4H). ^{13}C{1H} NMR (101 MHz, $CDCl_3$) δ 171.2, 147.3, 147.2, 146.0, 145.9, 143.7, 143.7, 134.9, 134.9, 134.1, 134.0, 134.0, 130.3, 130.3, 130.3, 130.2, 130.1, 130.1, 128.2, 128.1, 128.0, 127.6, 127.6, 126.8, 126.7, 122.9, 50.7, 50.6, 48.4, 31.5, 31.4, 28.5, 21.8. HRMS (ESI) *m/z* [*M* + H]$^+$ Calcd for $C_{48}H_{49}N_4O_6S_2$ 841.3088, found 841.3017.

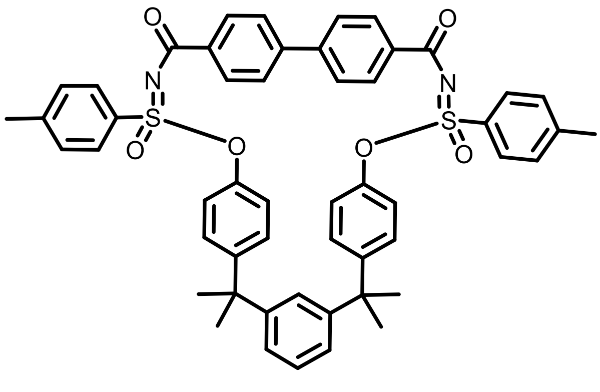

3t

3t was obtained as a white solid (105 mg, 0.12 mmol, 90%) ^{1}H NMR (400 MHz, $CDCl_3$) δ 8.16 – 8.00 (m, 4H), 8.00 – 7.86 (m, 4H), 7.71 (t, *J* = 2.0 Hz, 1H), 7.47 (t, *J* = 8.4 Hz, 8H), 7.41 – 7.32 (m, 4H), 7.24 – 7.14 (m, 4H), 6.39 (t, *J* = 7.8 Hz, 1H), 6.12 (dd, *J* = 7.8, 1.9 Hz, 2H), 2.54 (s, 6H), 1.72 (d, *J* = 2.1 Hz, 12H). ^{13}C{1H} NMR (101 MHz, $CDCl_3$) δ 170.2, 150.7, 149.0, 147.7, 145.7, 143.6, 134.7, 134.4, 130.1, 130.1, 128.6, 128.2, 127.9, 126.9, 125.0, 122.6, 121.7, 43.2, 31.4, 31.2, 21.8. HRMS (ESI) *m/z* [*M* + H]$^+$ Calcd for $C_{52}H_{47}N_2O_6S_2$ 859.2870, found 859.2855.

2.3 General procedure for the synthesis of enantiomeric chiral macrocycles

Procedure 1: To a seal tube (50 mL) with a magnetic stirring bar, was added di-sulfinimidoyl fluride compounds **(*R*, *R*)-1** (0.2 mmol, 1.0 equiv.) and 1.0 equiv of sodium salt of diphenols **2**. Next 10 mL of anhydrous acetonitrile was added into the seal tube. The reaction mixture was allowed to stir for 6 h at 60 ºC. Then the reaction was quenched with 20 mL water. The solution was extracted with CH_2Cl_2 (3 × 200 mL), dried with anhydrous Na_2SO_4 and concentrated by reduced pressure. The resulting residual was purified by silica gel column chromatography (n-hexane/EtOAc = 4:1 to 2:1) to afford the product **(*S*, *S*)-3**.

Procedure **2**: To a seal tube (50 mL) with a magnetic stirring bar, was added di-sulfinimidoyl fluoride compounds **(*R*, *R*)-1** (0.5 mmol, 1.0 equiv) and 1.0 equiv of diphenolates **2** (prepared according to a published procedure).[2] Next 40 mL of anhydrous acetonitrile was added into the seal tube. The reaction mixture was allowed to stir for 6 h at 60 ºC. Then the reaction was quenched with 50 mL water. The solution was extracted with CH_2Cl_2 (3 × 200 mL), dried with anhydrous Na_2SO_4 and concentrated by reduced pressure. The resulting residual was purified by silica gel column chromatography (*n*-hexane/EtOAc = 4:1 to 2:1) to afford **(*S*, *S*)-3** as white solid and **(*S*, *S*, *S*, *S*)-4** (*n*-hexane/EtOAc = 2:1 to 1:1) as crude product.

The corresponding macrocycles **(*R*, *R*)**-3 were prepared following the same procedures, but starting from the corresponding chiral di-sulfinimidoyl fluoride **(*S*, *S*)-1**. These macrocycles **(*R*, *R*)-3** give the same NMR spectra.

Note: All optical purities were measured by HPLC with chiral columns (see section 4). Because the diastereomers of several **(*S*, *S*)-3** in the mixture could not be separated by column chromatography, the mixture containing the meso- and enantiomers was used as the sample to be tested. The *dr* (diastereomeric ratio) was obtained here from the integrated area of the HPLC chromatogram.

The %*ee* (enantiomeric excess) of the chiral compounds with two chiral centers was calculated by

$$ee = \frac{\boldsymbol{RR-SS}}{\boldsymbol{RR+SS}} \times 100\% \qquad (1)$$

Where ***RR*** and ***SS*** refer to the integral area% of the respective peaks of the **(*R*, *R*)** and **(*S*, *S*)** enantiomers, respectively.

The *dr* (diastereomeric ratio) was calculated by

$$dr = \frac{(\boldsymbol{R,R})+(\boldsymbol{S,S})}{\boldsymbol{meso}} \qquad (2)$$

The %*es* (enantioselectivity) of the reaction for macrocycles **(*S*, *S*)-3** was calculated by:

es = % of **(*S*)**-stereocenters in **3**/ % of **(*R*)**-stereocenters in **1**

$$= \left. \frac{2*\boldsymbol{SS}+\boldsymbol{meso}}{200}\ in\ \mathbf{3} \middle/ \frac{2*\boldsymbol{RR}+\boldsymbol{meso}}{200}\ in\ \mathbf{1} \right. \times 100\% \qquad (3),$$

where ***RR*** and ***meso*** refer to the integral area% of the respective peaks of **(*R*, *R*)** and

meso configurations, respectively.

For macrocycles **(*S*, *S*, *S*, *S*)-4** we calculate it analogously: since the SuFEx reaction is enantiospecific, the expected *ee* per step is likely > 98%. As a result, isomers with 2 or more *S* centers are highly unlikely, and only **(*S*, *S*, *S*, *S*)-4** and **(*S*3*R*)** isomers, containing 3 **(*S*)**-stereocenters, are visible in chiral HPLC.

Therefore *es* =% of **(*S*)**-stereocenters in **4**/ % of **(*R*)**-stereocenters in **1**

$$= \frac{4*SSSS+3*(S3R)}{400}\ in\ \mathbf{4} \Bigg/ \frac{2*RR+meso}{200}\ in\ \mathbf{1} \times 100\% \qquad (4),$$

where ***SSSS*** and (***S3R***) refer to the integral area% of the respective peaks of **(*S*,*S*,*S*,*S*)** and (***S3R***) configurations, respectively.

2.4 Comparison of the syntheses of macrocycles using diphenol or diphenolate

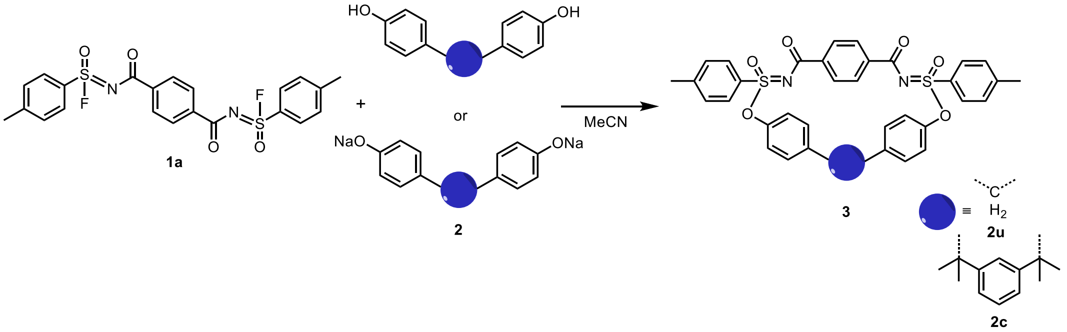

1.1 To a seal tube (50 mL) with a magnetic stirring bar, was added di-sulfinimidoyl fluride compounds **1a** (0.1 mmol, 48 mg, 1.0 equiv.) and 1.0 equiv of 4,4'-methylenediphenol **2u** in 10 mL of anhydrous acetonitrile, then added 2.1 equiv of DBU to the solution. The reaction mixture was allowed to stir for 6 h at 50 ºC. Then the reaction was quenched with 20 mL water. The solution was extracted with CH_2Cl_2 (3 × 100 mL), dried with anhydrous Na_2SO_4 and concentrated by reduced pressure. The resulting residual was purified by silica gel column chromatography (*n*-hexane/EtOAc = 2:1) to afford the product **3u** (60 mg, 94%). The **3u** was performed by prepared TLC (*n*-hxane:CH_2Cl_2: MeOH = 80:120:6) to obtain **racemic-3u** (29 mg, 46%) and **meso-3u** (30 mg, 47%) as white solid.

1.2. To a seal tube (50 mL) with a magnetic stirring bar, was added di-sulfinimidoyl fluride compounds **1a** (0.1 mmol, 48 mg, 1.0 equiv.) and 1.0 equiv of sodium 4,4'-methylenediphenolate **2u** and 2 equiv of 15-crown-5 ether in 10 mL of anhydrous acetonitrile. The reaction mixture was allowed to stir for 6 h at 50 ºC. Then the reaction was quenched with 20 mL water. The solution was extracted with CH_2Cl_2 (3 × 100 mL), dried with anhydrous Na_2SO_4 and concentrated by reduced pressure. The resulting residual was purified by silica gel column chromatography (*n*-hexane/EtOAc = 2:1) to afford the product **3u** (41 mg, 64%). The **3u** was performed by prepared TLC (*n*-hxane:CH_2Cl_2: MeOH = 80:120:6) to obtain **racemic-3u** (20 mg, 31%) and **meso-3u**

(20 mg, 31%) as white solid.

Racemic-3u: ^{1}H NMR (400 MHz, CD_2Cl_2-d_2) δ 7.97 – 7.92 (m, 4H), 7.50 – 7.46 (m, 4H), 7.38 (s, 4H), 7.29 – 7.25 (m, 4H), 7.06 – 7.02 (m, 4H), 3.88 (s, 2H), 2.52 (s, 6H).

meso-3u: ^{1}H NMR (400 MHz, CD_2Cl_2-d_2) δ 8.02 – 7.97 (m, 4H), 7.50 – 7.46 (m, 4H), 7.36 (s, 4H), 7.27 – 7.24 (m, 4H), 7.03 – 6.99 (m, 4H), 3.86 (d, J = 3.3 Hz, 2H), 2.52 (s, 6H).

2.1. To a seal tube (50 mL) with a magnetic stirring bar, was added di-sulfinimidoyl fluride compounds **1a** (0.5 mmol, 238 mg, 1.0 equiv.) and 1.0 equiv of 4,4'-(1,3-phenylenebis(propane-2,2-diyl))diphenol **2c** in 40 mL of anhydrous acetonitrile, then added 2.1 equiv of DBU to the solution. The reaction mixture was allowed to stir for 6 h at 50 ºC. Then the reaction was quenched with 50 mL water. The solution was extracted with CH_2Cl_2 (3 × 100 mL), dried with anhydrous Na_2SO_4 and concentrated by reduced pressure. The resulting residual was purified by silica gel column chromatography (*n*-hexane/EtOAc = 2:1) to afford the product **3c** (343 mg, 0.44 mmol,88%).

2.2. To a seal tube (50 mL) with a magnetic stirring bar, was added di-sulfinimidoyl fluride compounds **1a** (0.5 mmol, 238 mg, 1.0 equiv.) and 1.0 equiv. of sodium 4,4'-(1,3-phenylenebis(propane-2,2-diyl))diphenolate **2c** and 2 equiv. of 15-crown-5 ether in 40 mL of anhydrous acetonitrile. The reaction mixture was allowed to stir for 6 h at 50 ºC. Then the reaction was quenched with 50 mL water. The solution was extracted with CH_2Cl_2 (3 × 100 mL), dried with anhydrous Na_2SO_4 and concentrated by reduced pressure. The resulting residual was purified by silica gel column chromatography (*n*-hexane/EtOAc = 2:1) to afford the product **3c** (224 mg, 0.285 mmol, 57%).

Note: The TLC plots showed the reaction is not complete with diphenolates, resulting in the lower yields. We tried to isolate meso-**3c** from a mixture of diastereomer **3c**, but it neither worked by flash column nor preparative TLC. Thus, we confirmed the ratio of (R,R), (S,S) and meso-**3c** is 1:1:2 by chiral HPLC data (see Figure S145).

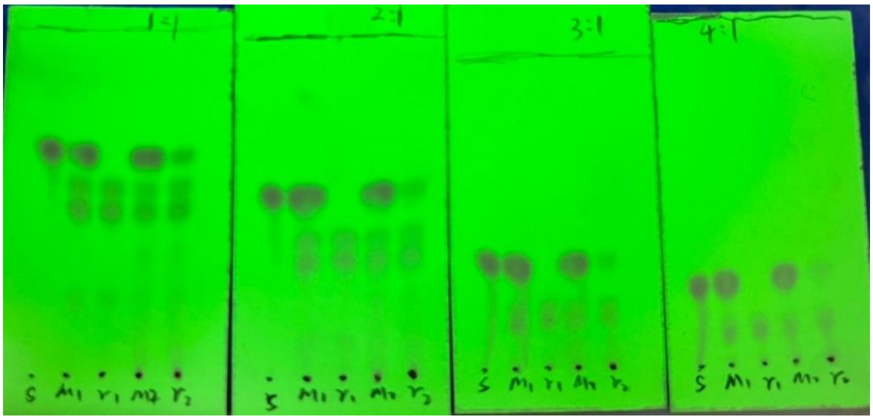

Figure S1. TLC plots of reactions for synthesizing **3c**. S = starting material, r1 = reaction was reacted with DBU and diphenol, r2 = reaction was reacted with sodium diphenolate, M = mixture of starting material and reaction solution. The eluents (from left to right) were *n*-hexane : EtOAc = 1 : 1, 2 : 1, 3 : 1, and 4 : 1, respectively.

Table S1. the synthesis of enantiomeric chiral macrocycles.

compound	Yield (%)	*ee* (%)	*dr*	*es* (%)
(*S*, *S*)-3a[a]	0.18 mmol, 93%	>99	101:1	>99
(*S*, *S*)-3c	0.21 mmol, 42%	>99	255:1	>99
(*R*, *R*)-3c	0.18 mmol, 35%	99	–	>99
(*S*, *S*)-3d[a]	0.14 mmol, 71%	>99	–	>99
(*S*, *S*)-3f[a]	0.15 mmol, 87%	98	125:1	>99
(*S*, *S*)-3k[a]	0.17 mmol, 85%	>99	–	>99
(*S*, *S*)-3o	0.32 mmol, 64%	98	19:1	97
(*R*, *R*)-3o	0.325 mmol, 65%	>99	10:1	96
(*S*, *S*)-3r[a]	0.142 mmol, 71%	>99	–	>99
(*S*, *S*)-3u[b]	0.27 mmol, 54%	99	31:1	99
(*R*, *R*)-3u[b]	0.21 mmol, 42%	98	31:1	98
(*S*, *S*)-3v[b]	0.17 mmol, 34%	97	–	99
(*R*, *R*)-3v[b]	0.13 mmol, 26%	97	–	99
(*S*, *S*)-3w	0.31 mmol, 62%	>99	–	>99
(*R*, *R*)-3w	0.27 mmol, 54%	>99	–	>99
(*S*, *S*)-3x	0.165 mmol, 33%	97	22:1	97
(*R*, *R*)-3x	0.175 mmol, 35%	99	–	>99
(*S*, *S*)-3y	0.19 mmol, 37%	>99	–	>99
(*R*, *R*)-3y	0.14 mmol, 27%	>99	–	>99
(*S*, *S*)-3Aa[b]	0.06 mmol, 12%	>99	12:1	97
(*R*, *R*)-3Aa[b]	0.06 mmol, 12%	>99	53:1	>99
(*S*, *S*)-3Ab[b]	0.07 mmol, 14%	99	12:1	96
(*R*, *R*)-3Ab[b]	0.05 mmol, 10%	>99	14:1	97
(*S*, *S*)-3Ac	0.08 mmol, 16%	>99	36:1	99
(*R*, *R*)-3Ac	0.07 mmol, 14%	>99	30:1	99
(*S*, *S*)-3Ad	0.10 mmol, 20%	99	41:1	99
(*R*, *R*)-3Ad	0.06 mmol, 12%	98	39:1	99
(*S*, *S*, *S*, *S*)-4a	0.03 mmol, 12%	>99	9:1	98
(*S*, *S*, *S*, *S*)-4b	0.04 mmol, 16%	>99	41:1	>99
(*S*, *S*, *S*, *S*)-4c	0.018 mmol, 7%	>99	14:1	99
(*S*, *S*, *S*, *S*)-4d	0.03 mmol, 12%	>99	10:1	98
(*S*, *S*, *S*, *S*)-4e	0.05 mmol, 20%	>99	4:1	95
(*S*, *S*, *S*, *S*)-4f[b]	0.07 mmol, 28%	>99	8:1	99
(*S*, *S*, *S*, *S*)-4g[b]	0.07 mmol, 28%	>99	21:1	>99
(*S*, *S*, *S*, *S*)-4h	0.03 mmol, 12%	>99	17:1	>99
(*S*, *S*, *S*, *S*)-4i	0.032 mmol, 13%	>99	19:1	>99
(*S*, *S*, *S*, *S*)-4j	0.04 mmol, 16%	>99	10:1	>99

Note: Most reactions were performed by procedure 2. But [a] The reaction was performed according to procedure 1. [b] Adding 2.0 equiv. 15-crown-5 ether to the reaction.

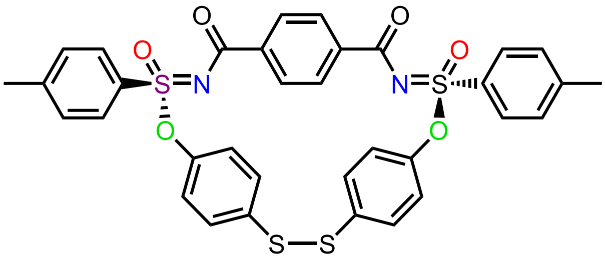

(*S*, *S*)-3a

(*S, S*)-3a was obtained as a white solid (128 mg, 0.18 mmol, 93%, >99% *es*). ^{1}H NMR (400 MHz, $CDCl_3$) δ 7.96 (dd, *J* = 36.9, 8.0 Hz, 4H), 7.74 (s, 2H), 7.66 – 7.52 (m, 3H), 7.43 (t, *J* = 7.4 Hz, 6H), 7.27 (s, 3H), 7.01 (d, *J* = 8.4 Hz, 2H), 2.52 (s, 6H). ^{13}C NMR (101 MHz, Chloroform-*d*) δ 169.9, 152.9, 146.5, 140.4, 134.8, 133.3, 133.2, 130.3, 130.3, 129.6, 128.2, 128.0, 124.2, 21.9. HRMS (ESI) *m/z* $[M + H]^+$ Calcd for $C_{34}H_{27}N_2O_6S_4$ 687.0746, found 687.0714.

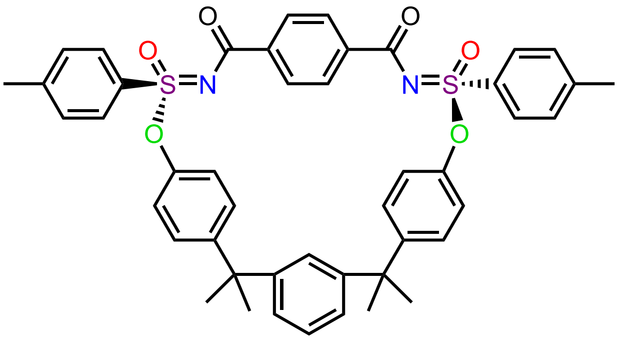

(*S*, *S*)-3c

(*S, S*)-3c was obtained as a white solid (164 mg, 0.21 mmol, 42%, >99% *es*). R_f = 0.19 (EtOAc/*n*-hexane = 1:3).^{1}H NMR (400 MHz, $CDCl_3$)δ 7.97 – 7.92 (m, 4H), 7.79 (s, 4H), 7.40 (d, *J* = 8.2 Hz, 4H), 7.25 – 7.19 (m, 1H), 7.11 – 7.06 (m, 6H), 6.99 – 6.93 (m, 4H), 6.89 (t, *J* = 1.9 Hz, 1H), 2.49 (s, 6H), 1.56 (s, 12H).^{13}C{1H} NMR (101 MHz, $CDCl_3$) δ 170.7, 150.9, 149.5, 147.1, 146.0, 138.6, 133.4, 130.1, 129.2, 128.4, 128.1, 128.0, 125.3, 124.8, 122.7, 43.3, 31.1, 31.0, 21.9. HRMS (ESI) *m/z* $[M + H]^+$ Calcd for $C_{46}H_{43}N_2O_6S_2$ 783.2557, found 783.2566.

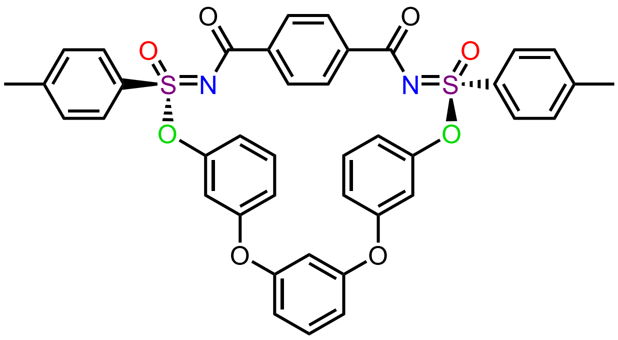

(*S*, *S*)-3d

(*S*, *S*)-3d was obtained as a white solid (103 mg, 0.14 mmol, 71%, >99% *es*). ^{1}H NMR (400 MHz, Chloroform-*d*) δ 8.06 – 7.90 (m, 4H), 7.79 (s, 4H), 7.43 (dd, *J* = 8.1, 4.8 Hz, 5H), 7.21 – 7.07 (m, 4H), 6.99 (dd, *J* = 8.2, 2.3 Hz, 2H), 6.92 – 6.81 (m, 4H), 6.56 (t, *J* = 2.3 Hz, 1H), 2.49 (s, 6H). ^{13}C NMR (101 MHz, Chloroform-*d*) δ 170.8, 157.1, 156.9, 146.0, 144.0, 138.4, 133.5, 131.5, 130.1, 129.0, 128.3, 124.3, 117.7, 117.7, 112.1, 21.8. HRMS (ESI) *m/z* [*M* + H]$^+$ Calcd for $C_{40}H_{31}N_2O_8S_2$ 731.1516, found 731.1574.

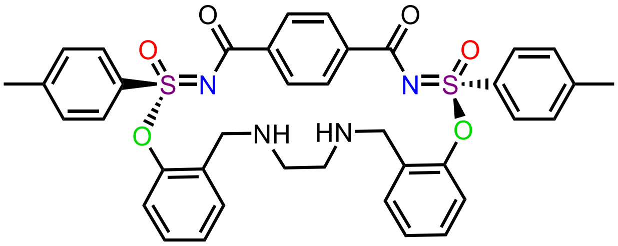

(*S*, *S*)-3f

(*S*, *S*)-3f was obtained as a white solid (106 mg, 0.15 mmol, 87%, >99% *es*) ^{1}H NMR (400 MHz, $CDCl_3$) δ 8.01 (dd, *J* = 8.5, 2.1 Hz, 4H), 7.87 (d, *J* = 11.6 Hz, 4H), 7.77 – 7.62 (m, 2H), 7.52 – 7.24 (m, 15H), 4.27 (d, *J* = 14.5 Hz, 2H), 3.88 (dd, *J* = 32.6, 14.5 Hz, 2H), 2.92 – 2.61 (m, 4H), 2.50 (s, 7H). ^{13}C NMR (101 MHz, $CDCl_3$) δ 171.0, 146.7, 146.2, 138.7, 133.5, 130.3, 129.9, 129.1, 128.3, 128.2, 128.1, 127.9, 123.2, 50.1, 48.1, 21.8. HRMS (ESI) *m/z* [*M* + H]$^+$ Calcd for $C_{38}H_{37}N_4O_6S_2$ 709.2149, found 709.2134.

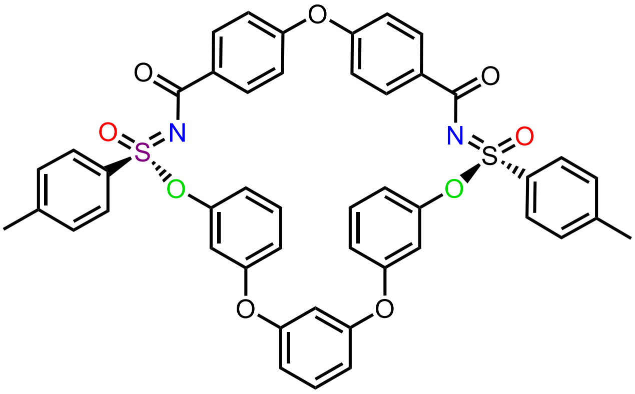

(*S*, *S*)-3k

(*S*, *S*)-3k was obtained as a white solid (139 mg, 0.17 mmol, 85%, >99% *es*) ^{1}H NMR (400 MHz, $CDCl_3$) δ 8.06 – 7.91 (m, 4H), 7.79 (s, 4H), 7.43 (dd, *J* = 8.1, 4.8 Hz, 5H), 7.20 – 7.06 (m, 4H), 6.99 (dd, *J* = 8.1, 2.2 Hz, 2H), 6.92 – 6.83 (m, 4H), 6.56 (s, 1H), 2.49 (s, 6H). ^{13}C NMR (101 MHz, $CDCl_3$) δ 170.8, 157.1, 156.9, 146.0, 143.9, 133.5, 131.5, 129.0, 128.3, 124.3, 117.7, 117.7, 112.1, 21.8. HRMS (ESI) *m/z* [*M* + H]$^+$ Calcd for $C_{46}H_{35}N_2O_9S_2$ 823.1465, found 823.1448.

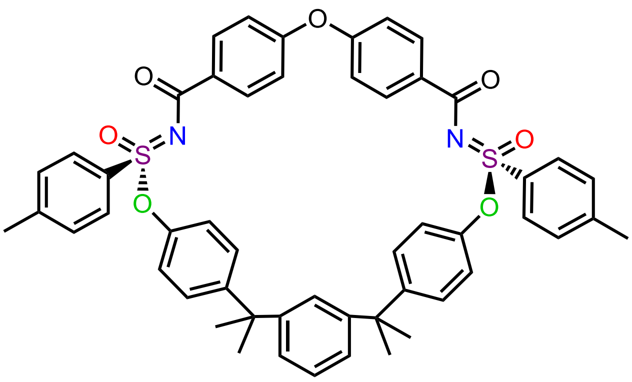

(*S*, *S*)-3o

(*S, S*)-3o was obtained as a white solid (280 mg, 0.32 mmol, 64%, 97% *es*). R_f = 0.28 (EtOAc/*n*-hexane = 1:2).^{1}H NMR (400 MHz, $CDCl_3$) δ 8.06 – 8.01 (m, 4H), 7.90 – 7.83 (m, 4H), 7.42 (d, *J* = 8.1 Hz, 4H), 7.35 (t, *J* = 1.9 Hz, 1H), 7.27 (d, *J* = 2.1 Hz, 2H), 7.25 (d, *J* = 2.3 Hz, 2H), 7.17 – 7.11 (m, 4H), 6.93 – 6.87 (m, 4H), 6.74 (t, *J* = 7.7 Hz, 1H), 6.50 (dd, *J* = 7.6, 1.8 Hz, 2H), 2.49 (s, 6H), 1.61 (d, *J* = 9.6 Hz, 12H). ^{13}C{1H} NMR (101 MHz, $CDCl_3$) δ 170.4, 160.2, 150.5, 149.7, 147.6, 145.8, 134.3, 131.7, 131.0, 130.1, 128.5, 128.3, 127.5, 125.0, 123.0, 122.2, 118.4, 43.2, 31.3, 31.0, 21.9. HRMS (ESI) *m/z* $[M + H]^+$ Calcd for $C_{52}H_{47}N_2O_7S_2$ 875.2819, found 875.2831.

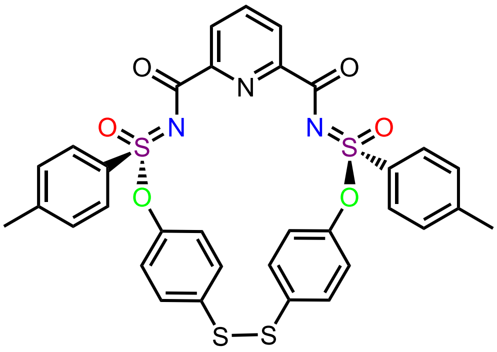

(*S*, *S*)-3r

(*S, S*)-3r was obtained as a white solid (98 mg, 0.142 mmol, 71%, >99% *es*). ^{1}H NMR (400 MHz, $CDCl_3$) δ 8.16 (d, *J* = 7.7 Hz, 2H), 8.06 – 7.99 (m, 4H), 7.81 (t, *J* = 7.8 Hz, 1H), 7.36 (d, *J* = 8.3 Hz, 4H), 7.30 – 7.25 (m, 4H), 7.13 – 7.05 (m, 4H), 2.43 (s, 6H). ^{13}C NMR (101 MHz, $CDCl_3$) δ 169.2, 151.6, 148.2, 146.0, 137.5, 135.1, 132.9, 130.0, 129.8, 128.5, 126.6, 123.7, 123.5, 21.9. HRMS (ESI) *m/z* $[M + H]^+$ Calcd for $C_{33}H_{26}N_3O_6S_4$ 688.0386, found 688.0365.

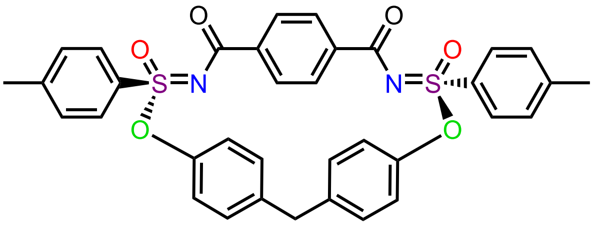

(*S*, *S*)-3u

(*S, S*)-3u was obtained as a white solid (170 mg, 0.27 mmol, 54%, 99% *es*). R_f = 0.16 (EtOAc/*n*-hexane = 1:3).^{1}H NMR (400 MHz, $CDCl_3$) δ 8.02 – 7.95 (m, 4H), 7.44 (d, *J*

= 8.2 Hz, 4H), 7.38 (s, 4H), 7.26 (s, 2H), 7.25 (s, 2H), 7.12 – 7.05 (m, 4H), 3.86 (s, 2H), 2.50 (s, 6H). ^{13}C{1H} NMR (101 MHz, $CDCl_3$) δ 169.6 (s), 148.7 (s), 145.9 (s), 140.9 (s), 138.4 (s), 134.7 (s), 130.3 (s), 129.9 (s), 129.2 (s), 128.1 (s), 123.34 (s), 41.7 (s), 21.9 (s). HRMS (ESI) *m/z* $[M + H]^+$ Calcd for $C_{35}H_{29}N_2O_6S_2$ 637.1462, found 637.1469.

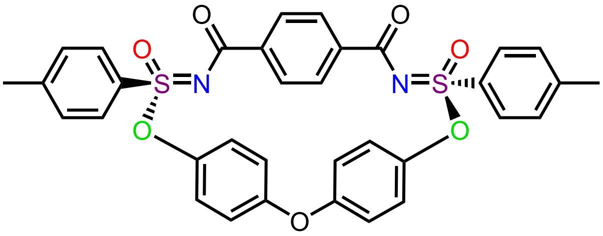

(S, S)-3v

(*S*, *S*)-3v was obtained as a white solid (110 mg, 0.17 mmol, 34%, 99% *es*). R_f = 0.17 (EtOAc/*n*-hexane = 1:3). ^{1}H NMR (400 MHz, $CDCl_3$) δ 7.99 (d, *J* = 8.2 Hz, 4H), 7.70 (s, 4H), 7.47 (d, *J* = 8.1 Hz, 4H), 6.94 – 6.87 (m, 4H), 6.85 – 6.78 (m, 4H), 2.53 (s, 6H). ^{13}C{1H} NMR (101 MHz, $CDCl_3$) δ 170.4, 157.8, 146.2, 146.0, 138.7, 133.9, 130.3, 129.1, 128.3, 124.6, 121.9, 22.0. HRMS (ESI) *m/z* $[M + H]^+$ Calcd for $C_{34}H_{27}N_2O_7S_2$ 639.1254, found 639.1256.

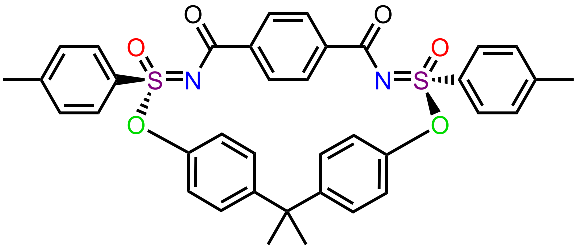

(S, S)-3w

(*S*, *S*)-3w was obtained as a white solid (207 mg, 0.31 mmol, 62%, >99% *es*). R_f = 0.28 (EtOAc/*n*-hexane = 1:2). ^{1}H NMR (400 MHz, $CDCl_3$) δ 7.98 (d, *J* = 8.1 Hz, 4H), 7.67 (s, 4H), 7.45 (d, *J* = 8.0 Hz, 4H), 7.13 (d, *J* = 8.0 Hz, 4H), 6.91 (d, *J* = 8.0 Hz, 4H), 2.52 (s, 6H), 1.60 (s, 6H). ^{13}C{1H} NMR (101 MHz, $CDCl_3$) δ 170.0, 149.3, 147.8, 146.0, 138.5, 134.2, 130.3, 129.3, 128.3, 127.7, 122.9, 42.2, 30.3, 21.9. HRMS (ESI) *m/z* $[M + H]^+$ Calcd for $C_{37}H_{33}N_2O_6S_2$ 665.1775, found 665.1775.

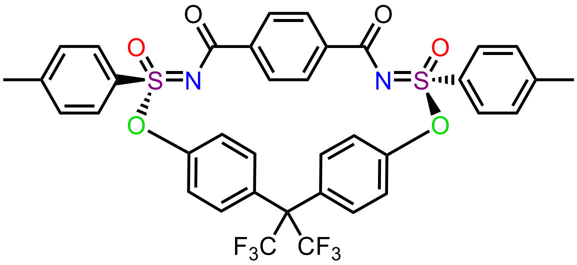

(S, S)-3x

(*S*, *S*)-3x was obtained as a white solid (127 mg, 0.165 mmol, 33%, 97% *es*). R_f = 0.23 (EtOAc/*n*-hexane = 1:3). ^{1}H NMR (400 MHz, $CDCl_3$) δ 7.99 (s, 4H), 7.98 – 7.93 (m, 4H), 7.48 (d, *J* = 7.9 Hz, 4H), 7.02 (d, *J* = 8.5 Hz, 4H), 6.86 – 6.80 (m, 4H), 2.55 (s, 6H). ^{19}F NMR (376 MHz, $CDCl_3$) δ -63.80. ^{13}C{1H} NMR (101 MHz, $CDCl_3$) δ 170.9,

149.7, 146.6, 138.9, 133.0, 132.9, 131.1, 130.4, 129.4, 128.6, 123.5, 22.0. HRMS (ESI) *m/z* $[M + H]^+$ Calcd for $C_{37}H_{27}F_6N_2O_6S_2$ 773.1209, found 773.1213.

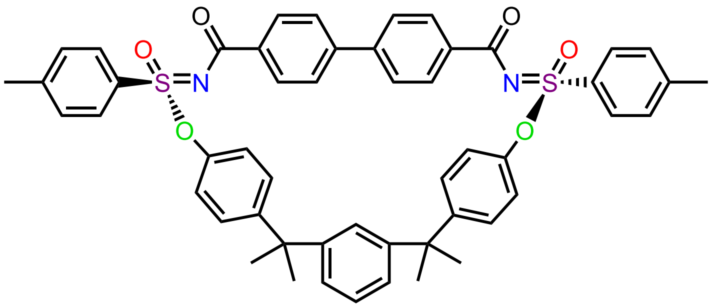

(*S*, *S*)-3y

(*S, S*)-3y was obtained as a white solid (159 mg, 0.19 mmol, 37%, >99% *es*). R_f = 0.27 (EtOAc/*n*-hexane = 1:2).^{1}H NMR (400 MHz, $CDCl_3$) δ 8.08 – 8.03 (m, 4H), 7.92 – 7.87 (m, 4H), 7.69 (t, *J* = 1.9 Hz, 1H), 7.48 – 7.41 (m, 8H), 7.36 – 7.31 (m, 4H), 7.19 – 7.13 (m, 4H), 6.37 (t, *J* = 7.8 Hz, 1H), 6.10 (dd, *J* = 7.8, 1.9 Hz, 2H), 2.51 (s, 6H), 1.69 (d, *J* = 2.2 Hz, 12H). $^{13}C\{^1H\}$ NMR (101 MHz, $CDCl_3$) δ 170.3, 150.8, 149.1, 147.8, 145.9, 143.7, 134.8, 134.6, 130.3, 130.3, 128.7, 128.3, 128.1, 127.0, 125.1, 122.7, 121.8, 43.4, 31.6, 31.3, 21.9. HRMS (ESI) *m/z* $[M + H]^+$ Calcd for $C_{52}H_{47}N_2O_6S_2$ 859.2870, found 859.2878.

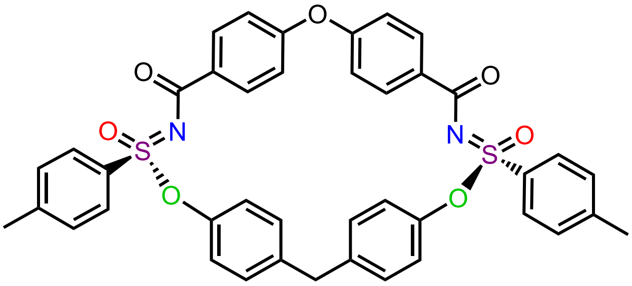

(*S*, *S*)-3Aa

(*S, S*)-3Aa was obtained as a white solid (47 mg, 0.06 mmol, 12%, 97% *es*). R_f = 0.29 (EtOAc/*n*-hexane = 1:2).^{1}H NMR (400 MHz, $CDCl_3$) δ 8.03 – 7.93 (m, 8H), 7.41 (d, *J* = 8.2 Hz, 4H), 7.12 (d, *J* = 8.6 Hz, 4H), 7.08 – 7.02 (m, 4H), 6.87 – 6.79 (m, 4H), 3.98 (s, 2H), 2.49 (s, 6H). $^{13}C\{^1H\}$ NMR (101 MHz, $CDCl_3$) δ 170.7, 161.8, 147.9, 146.0, 139.1, 133.5, 131.8, 131.3, 130.3, 130.2, 128.4, 123.2, 119.4, 40.6, 21.9. HRMS (ESI) *m/z* $[M + H]^+$ Calcd for $C_{41}H_{33}N_2O_7S_2$ 729.1724, found 729.1729.

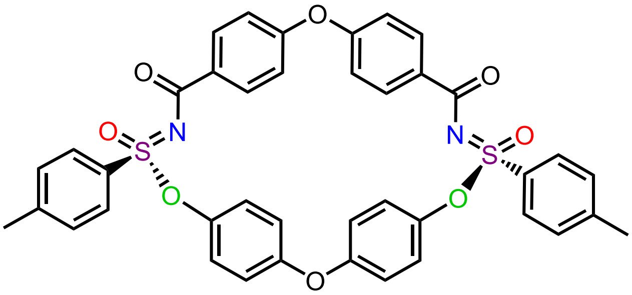

(*S*, *S*)-3Ab

(*S*, *S*)-3Ab was obtained as a white solid (52 mg, 0.07 mmol, 14%, 96% *es*). $R_f = 0.24$ (EtOAc/*n*-hexane = 1:2).^{1}H NMR (400 MHz, $CDCl_3$) δ 8.01 – 7.93 (m, 8H), 7.42 (d, J = 8.1 Hz, 4H), 7.06 – 6.99 (m, 4H), 6.94 – 6.88 (m, 4H), 6.87 – 6.81 (m, 4H), 2.50 (s, 6H). ^{13}C{1H} NMR (101 MHz, $CDCl_3$) δ 170.3, 161.8, 155.2, 146.2, 144.9, 133.3, 131.9, 131.2, 130.2, 128.4, 124.8, 119.3, 119.2, 22.0. HRMS (ESI) *m/z* $[M + H]^+$ Calcd for $C_{40}H_{31}N_2O_8S_2$ 731.1516, found 731.1515.

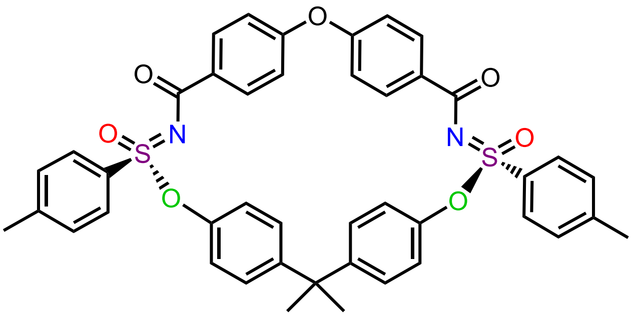

(*S*, *S*)-3Ac

(*S*, *S*)-3Ac was obtained as a white solid (60 mg, 0.08 mmol, 16%, 99% *es*). $R_f = 0.26$ (EtOAc/*n*-hexane = 1:2).^{1}H NMR (400 MHz, $CDCl_3$) δ 8.02 – 7.97 (m, 4H), 7.97 – 7.92 (m, 4H), 7.44 – 7.39 (m, 4H), 7.19 – 7.14 (m, 4H), 6.99 – 6.94 (m, 4H), 6.90 – 6.83 (m, 4H), 2.49 (s, 6H), 1.54 (s, 6H). ^{13}C{1H} NMR (101 MHz, $CDCl_3$) δ 170.9, 161.6, 149.3, 147.6, 146.0, 133.8, 131.8, 131.7, 130.2, 128.4, 128.3, 122.7, 119.3, 43.4, 32.0, 21.9. HRMS (ESI) *m/z* $[M + H]^+$ Calcd for $C_{43}H_{37}N_2O_7S_2$ 757.2037, found 757.2036.

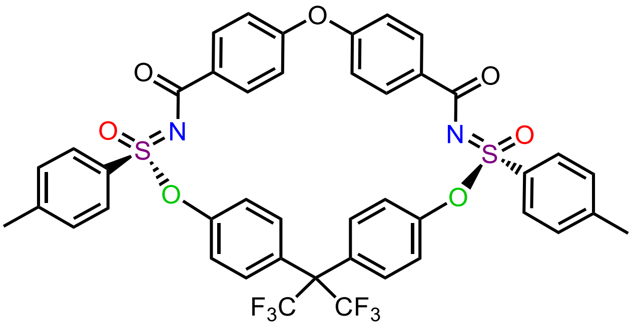

(*S*, *S*)-3Ad

(*S*, *S*)-3Ad was obtained as a white solid (88 mg, 0.10 mmol, 20%, 99% *es*). $R_f = 0.43$ (EtOAc/*n*-hexane = 1:2).^{1}H NMR (400 MHz, $CDCl_3$) δ 8.00 – 7.94 (m, 4H), 7.95 – 7.88 (m, 4H), 7.42 (d, J = 8.1 Hz, 4H), 7.32 (d, J = 8.6 Hz, 4H), 7.06 – 6.98 (m, 4H), 6.90 – 6.81 (m, 4H), 2.50 (s, 6H). ^{19}F NMR (376 MHz, $CDCl_3$) δ -63.94. ^{13}C{1H} NMR (101 MHz, $CDCl_3$) δ 170.2, 161.7, 150.2, 146.4, 133.3, 131.87, 131.7, 131.6, 131.3, 130.3, 128.4, 123.2, 119.3, 21.9. HRMS (ESI) *m/z* $[M + H]^+$ Calcd for $C_{43}H_{31}F_6N_2O_7S_2$ 865.1471, found 865.1477.

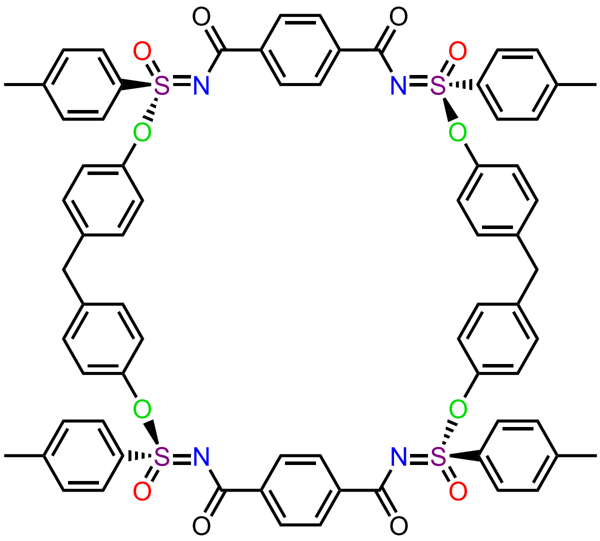

(*S*, *S*, *S*, *S*)-4a

The crude mixture was purified by preparative TLC (*n*-hexane/CH_2Cl_2/MeOH = 100:100:6) to afford ***(S, S, S, S)*-4a** as a white solid (35 mg, 0.03 mmol, 12%, 98% *es*). R_f = 0.08 (EtOAc/*n*-hexane = 1:2).^{1}H NMR (400 MHz, $CDCl_3$) δ 8.03 (s, 8H), 7.94 – 7.89 (m, 8H), 7.32 (d, *J* = 8.2 Hz, 8H), 7.02 (d, *J* = 1.2 Hz, 16H), 3.90 (s, 4H), 2.43 (s, 12H). ^{13}C{1H} NMR (101 MHz, $CDCl_3$) δ 171.1 (s), 147.7 (s), 146.0 (s), 139.9 (s), 138.6 (s), 133.0 (s), 130.2 (s), 130.1 (s), 129.4 (s), 128.4 (s), 123.1 (s), 40.6 (s), 21.9 (s). HRMS (ESI) *m/z* $[M + H]^+$ Calcd for $C_{70}H_{57}N_4O_{12}S_4$ 1273.2850, found 1273.2878.

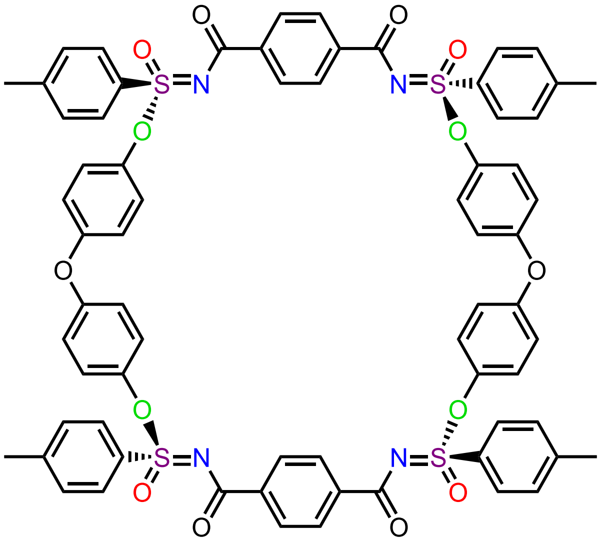

(*S*, *S*, *S*, *S*)-4b

The crude mixture was purified by preparative TLC (*n*-hexane/CH_2Cl_2/MeOH = 100:100:6) to afford ***(S, S, S, S)*-4b** as a white solid (50 mg, 0.04 mmol, 16%, >99% *es*). R_f = 0.06 (EtOAc/*n*-hexane = 1:3).^{1}H NMR (400 MHz, $CDCl_3$) δ 8.07 (s, 8H), 8.00 – 7.95 (m, 8H), 7.40 (d, *J* = 8.2 Hz, 8H), 7.13 – 7.04 (m, 8H), 6.88 – 6.77 (m, 8H), 2.47 (s, 12H). ^{13}C{1H} NMR (101 MHz, $CDCl_3$) δ 171.1, 155.9, 146.2, 144.8, 138.6, 132.9, 130.2, 129.4, 128.4, 124.5, 119.8, 21.9. HRMS (ESI) *m/z* $[M + H]^+$ Calcd for $C_{68}H_{53}N_4O_{14}S_4$ 1277.2436, found 1277.2416.

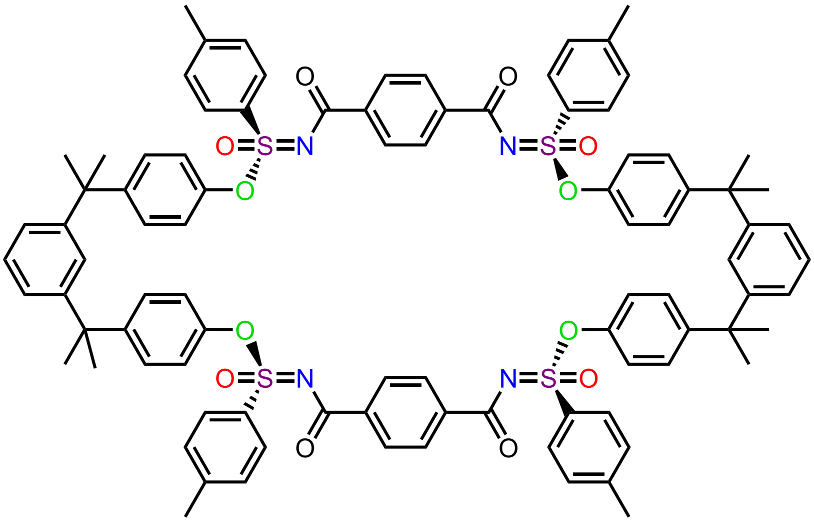

(*S*, *S*, *S*, *S*)-4c

The crude mixture was purified by preparative TLC (*n*-hexane/CH_2Cl_2/MeOH = 100:100:6) to afford ***(S, S, S, S)*-4c** as a white solid (28 mg, 0.018 mmol, 7%, 99% *es*). R_f = 0.3 (EtOAc/*n*-hexane = 2:3).^{1}H NMR (400 MHz, $CDCl_3$) δ 8.08 (s, 8H), 7.96 (d, *J* = 8.0 Hz, 8H), 7.35 (d, *J* = 8.0 Hz, 8H), 7.15 (t, *J* = 7.7 Hz, 2H), 7.00 (d, *J* = 8.5 Hz, 12H), 6.94 (d, *J* = 8.7 Hz, 8H), 6.67 (s, 2H), 2.44 (s, 12H), 1.51 (d, *J* = 5.7 Hz, 24H). ^{13}C{1H} NMR (101 MHz, $CDCl_3$) δ 171.3, 150.2, 149.8, 147.0, 145.9, 138.7, 133.4, 130.1, 129.5, 128.4, 128.1, 127.9, 126.4, 123.8, 122.2, 42.9, 30.7, 30.6, 21.9. HRMS (ESI) *m/z* $[M + H]^+$ Calcd for $C_{92}H_{85}N_4O_{12}S_4$ 1565.5041 found 1565.5061.

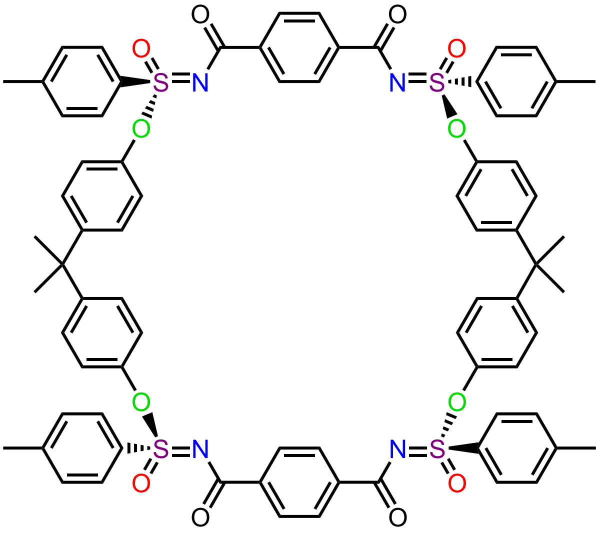

(*S*, *S*, *S*, *S*)-4d

The crude mixture was purified by preparative TLC (*n*-hexane/CH_2Cl_2/MeOH = 100:100:6) to afford ***(S, S, S, S)*-4d** as a white solid (38 mg, 0.03 mmol, 12%, 98% *es*). R_f = 0.08 (EtOAc/*n*-hexane = 1:2). ^{1}H NMR (400 MHz, $CDCl_3$) δ 8.02 (s, 8H), 7.92 (d, *J* = 8.0 Hz, 8H), 7.30 (d, *J* = 8.1 Hz, 8H), 7.08 – 7.00 (m, 16H), 2.42 (s, 12H), 1.59 (s, 12H).^{13}C{1H} NMR (101 MHz, $CDCl_3$) δ 171.1, 149.6, 147.2, 146.0, 138.7, 133.2, 130.1, 129.4, 128.3, 128.1, 122.5, 42.8, 30.9, 21.9. HRMS (ESI) *m/z* $[M + H]^+$ Calcd for $C_{74}H_{65}N_4O_{12}S_4$ 1329.3476, found 1329.3483.

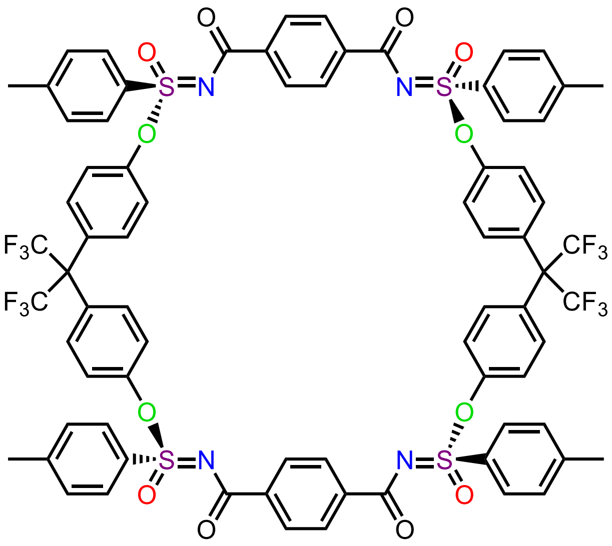

(*S*, *S*, *S*, *S*)-4e

The crude mixture was purified by preparative TLC (*n*-hexane/CH_2Cl_2/MeOH = 100:100:6) to afford **(*S*, *S*, *S*, *S*)-4e** as a white solid (82 mg, 0.05 mmol, 20%, 95% *es*). R_f = 0.09 (EtOAc/*n*-hexane = 1:3). ^{1}H NMR (400 MHz, $CDCl_3$) δ 8.06 (s, 8H), 7.98 (d, *J* = 8.0 Hz, 8H), 7.37 (d, *J* = 8.1 Hz, 8H), 7.25 (d, *J* = 8.1 Hz, 8H), 7.18 (d, *J* = 8.6 Hz, 8H), 2.45 (s, 12H). ^{19}F NMR (376 MHz, $CDCl_3$) δ -63.87. ^{13}C{1H} NMR (101 MHz, $CDCl_3$) δ 170.9, 149.8, 146.4, 138.6, 133.1, 132.3, 131.7, 130.2, 129.4, 128.3, 125.3, 122.9, 122.4, 64.3, 64.0, 63.8, 21.9. HRMS (ESI) *m/z* [*M* + Na]$^+$ Calcd for $C_{74}H_{52}F_{12}N_4O_{12}S_4Na$ 1567.2165, found 1567.2156.

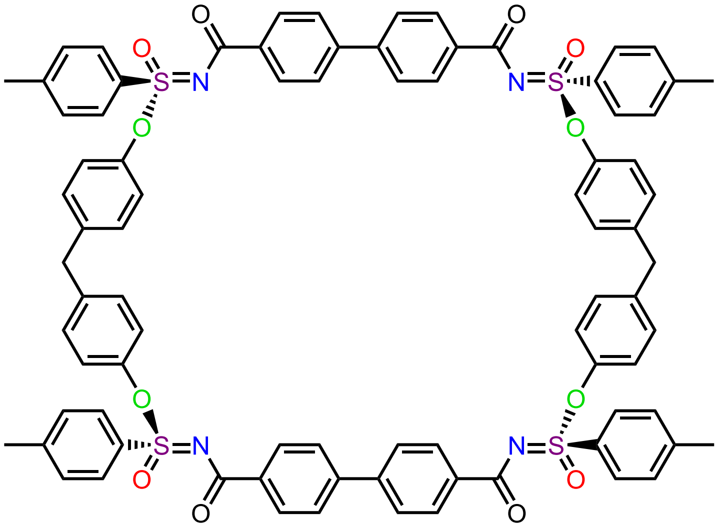

(*S*, *S*, *S*, *S*)-4f

The crude mixture was purified by preparative TLC (*n*-hexane/CH_2Cl_2/MeOH = 100:100:6) to afford **(*S*, *S*, *S*, *S*)-4f** as a white solid (100 mg, 0.07 mmol, 28%, 99% *es*). R_f = 0.3 (EtOAc/*n*-hexane = 1:1) ^{1}H NMR (400 MHz, $CDCl_3$) δ 8.12 (d, *J* = 8.3 Hz, 8H), 7.93 (d, *J* = 8.3 Hz, 8H), 7.61 (d, *J* = 8.3 Hz, 8H), 7.33 (d, *J* = 8.2 Hz, 8H), 7.03 (s, 16H), 3.88 (s, 4H), 2.44 (s, 12H). ^{13}C{1H} NMR (101 MHz, $CDCl_3$) δ 171.5, 147.7, 145.8, 144.1, 139.9, 134.7, 133.4, 130.3, 130.1, 130.0, 128.3, 127.0, 123.0, 40.6, 21.8. HRMS (ESI) *m/z* [*M* + H]$^+$ Calcd for $C_{82}H_{65}N_4O_{12}S_4$ 1425.3476, found 1425.3506.

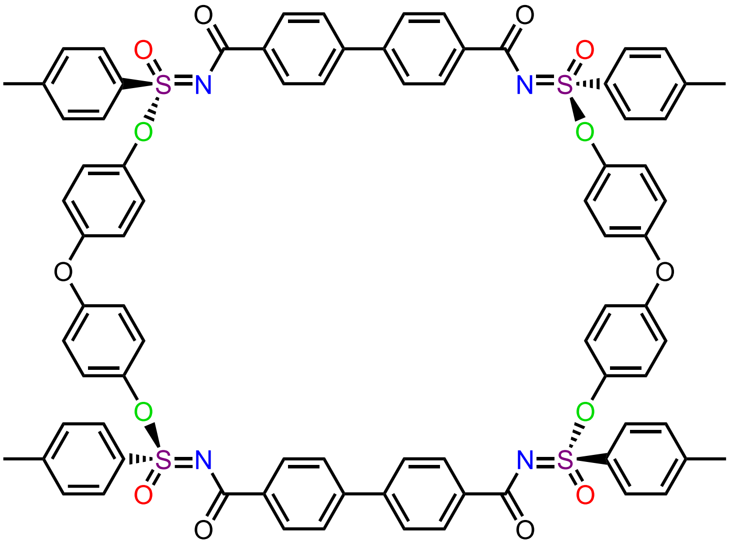

(*S*, *S*, *S*, *S*)-4g

The crude mixture was purified by preparative TLC (*n*-hexane/CH_2Cl_2/MeOH = 100:100:6) to afford **(*S*, *S*, *S*, *S*)-4g** as a white solid (94 mg, 0.07 mmol, 28%, >99% *es*). R_f = 0.27 (EtOAc/*n*-hexane = 1:1) ^{1}H NMR (400 MHz, $CDCl_3$) δ 8.16 (d, J = 8.2 Hz, 8H), 8.01 – 7.93 (m, 8H), 7.66 (d, J = 8.3 Hz, 8H), 7.38 (d, J = 8.2 Hz, 8H), 7.13 – 7.04 (m, 8H), 6.89 – 6.80 (m, 8H), 2.46 (s, 12H). ^{13}C{1H} NMR (101 MHz, $CDCl_3$) δ 171.4, 155.8, 146.0, 144.8, 144.2, 134.7, 133.2, 130.3, 130.1, 128.3, 127.1, 124.5, 119.8, 21.9. HRMS (ESI) m/z $[M + H]^+$ Calcd for $C_{80}H_{61}N_4O_{14}S_4$ 1429.3062, found 1429.3075.

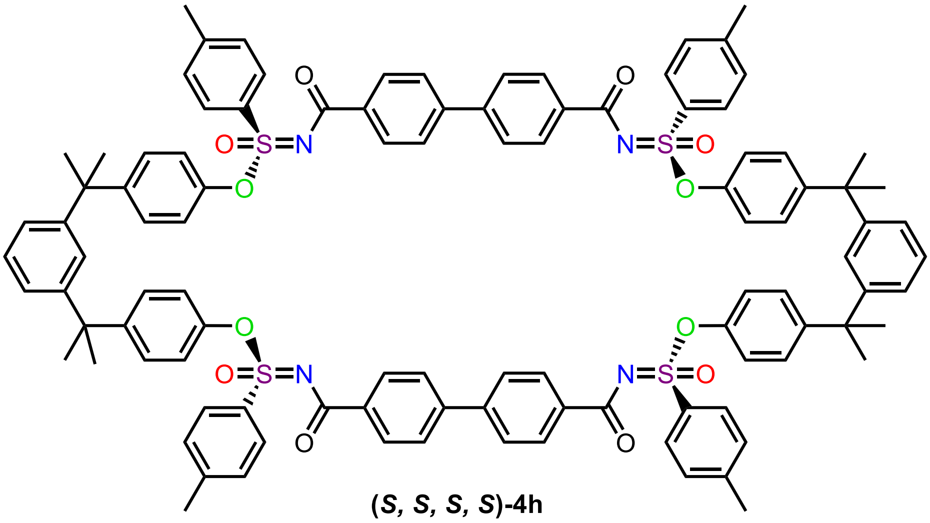

(*S*, *S*, *S*, *S*)-4h

The crude mixture was purified by preparative TLC (*n*-hexane/CH_2Cl_2/MeOH = 100:100:6) to afford **(*S*, *S*, *S*, *S*)-4h** as a white solid (53 mg, 0.03 mmol, 12%, >99% *es*). R_f = 0.4 (EtOAc/*n*-hexane = 2:3).^{1}H NMR (400 MHz, $CDCl_3$) δ 8.15 (d, J = 8.0 Hz, 8H), 7.99 (d, J = 8.1 Hz, 8H), 7.54 (d, J = 8.1 Hz, 8H), 7.37 (d, J = 8.0 Hz, 8H), 7.06 – 6.94 (m, 22H), 6.69 (s, 2H), 2.45 (s, 12H), 1.52 (d, J = 7.2 Hz, 24H). ^{13}C{1H} NMR (101 MHz, $CDCl_3$) δ 171.5, 150.1, 149.8, 147.1, 145.7, 144.1, 134.8, 133.6, 130.3, 130.1, 128.4, 128.1, 127.8, 127.0, 126.5, 123.7, 122.2, 42.9, 30.6, 30.6, 21.9. HRMS (ESI) m/z $[M + H]^+$ Calcd for $C_{104}H_{93}N_4O_{12}S_4$ 1717.5667, found 1717.5726.

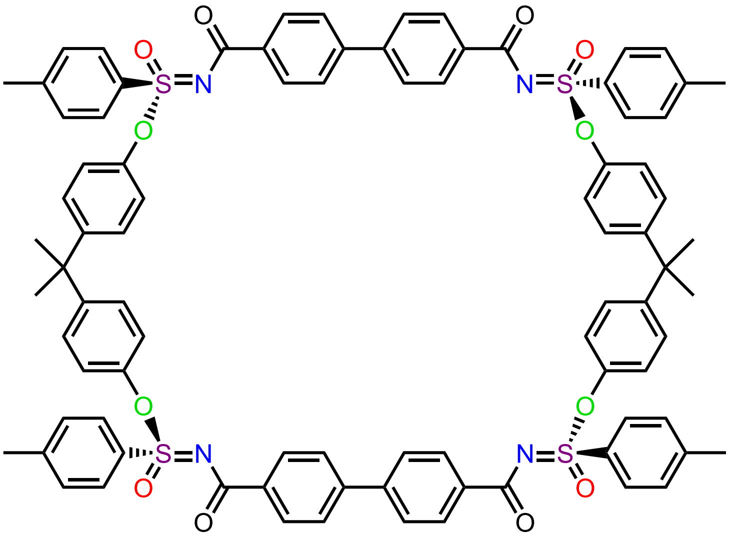

(*S*, *S*, *S*, *S*)-4i

The crude mixture was purified by preparative TLC (*n*-hexane/CH_2Cl_2/MeOH = 100:100:6) to afford **(*S, S, S, S*)-4i** as a white solid (47 mg, 0.032 mmol, 13%, >99% *es*). R_f = 0.29 (EtOAc/*n*-hexane = 1:2). ^{1}H NMR (400 MHz, $CDCl_3$) δ 8.11 (d, *J* = 8.1 Hz, 8H), 7.93 (d, *J* = 8.1 Hz, 8H), 7.62 (d, *J* = 8.1 Hz, 8H), 7.33 (d, *J* = 8.1 Hz, 8H), 7.09 (d, *J* = 8.5 Hz, 8H), 7.03 (d, *J* = 8.7 Hz, 8H), 2.45 (s, 12H), 1.61 (s, 12H). ^{13}C{1H} NMR (101 MHz, $CDCl_3$) δ 171.5, 149.6, 147.3, 145.8, 144.2, 134.8, 133.7, 130.3, 130.1, 128.3, 128.1, 127.1, 122.5, 42.7, 30.7, 21.9. HRMS (ESI) *m/z* [*M* + H]$^+$ Calcd for $C_{86}H_{73}N_4O_{12}S_4$ 1481.4102, found 1481.4110.

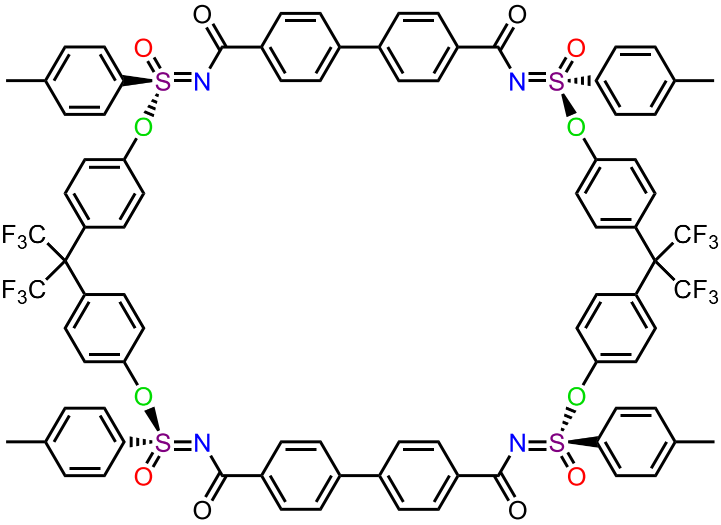

(*S*, *S*, *S*, *S*)-4j

The crude mixture was purified by preparative TLC (*n*-hexane/CH_2Cl_2/MeOH = 100:100:6) to afford **(*S, S, S, S*)-4j** as a white solid (61 mg, 0.04 mmol, 16%, >99% *es*). R_f = 0.1 ((EtOAc/*n*-hexane = 1:2). ^{1}H NMR (400 MHz, $CDCl_3$) δ 8.12 (d, *J* = 8.0 Hz, 8H), 7.99 (d, *J* = 8.2 Hz, 8H), 7.68 (d, *J* = 8.3 Hz, 8H), 7.39 (d, *J* = 8.0 Hz, 8H), 7.28 (d, *J* = 8.7 Hz, 8H), 7.19 (d, *J* = 9.0 Hz, 8H), 2.48 (s, 12H). ^{19}F NMR (376 MHz, $CDCl_3$) δ -63.85. ^{13}C{1H} NMR (101 MHz, $CDCl_3$) δ 171.2, 149.9, 146.2, 144.4, 134.6, 133.6, 132.4, 131.7, 130.3, 130.2, 128.2, 127.2, 122.9, 21.9. HRMS (ESI) *m/z* [*M* + H]$^+$ Calcd for $C_{86}H_{61}F_{12}N_4O_{12}S_4$ 1697.2972, found 1697.2975.

2.5 Synthesis of diastereomeric macrocycle on gram scale

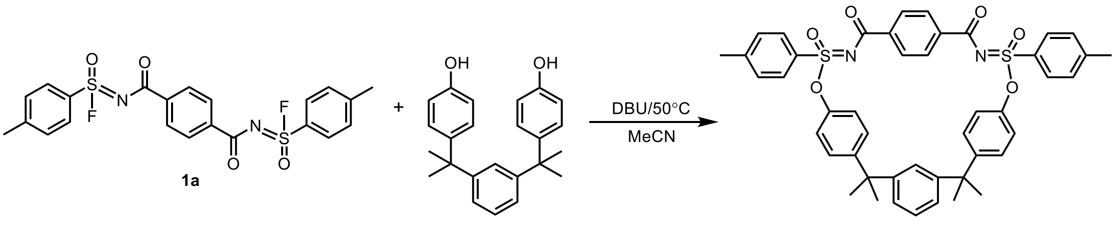

To a seal tube (150 mL) with a magnetic stirring bar, was added di-sulfonimidoyl fluoride compound **1a** (1.0 g, 2.1 mmol, 1.0 equiv.) and 4,4'-(1,3-phenylenebis(propane-2,2-diyl))diphenol (728 mg, 2.1 mmol, 1.0 equiv). Next 100 mL of anhydrous acetonitrile was added into the seal tube. Finally, DBU (660 μL, 4.41 mmol, 2.1 equiv) was added. The reaction mixture was allowed to stir for 6 h at 50 ºC. Then the reaction was quenched with 100 mL water. The solution was extracted with CH_2Cl_2 (3 × 200 mL), dried with anhydrous Na_2SO_4 and concentrated by reduced pressure. The resulting residual was purified by silica gel column chromatography (*n*-hexane/EtOAc = 2:1) to afford the product **3c** as a white solid (1.47g, 1.88 mmol, 89%). The NMR data see the section 2.2.

3. NMR spectra

Note: In order to distinguish [1+1] macrocycles and larger [2+2] macrocycles, we performed DOSY NMR to obtain their diffusion coefficient (*D*) respectively. According to the equation (5) to calculate the hydrodynamic radius of the solute (r_H).[3]

$$r_H = \frac{k_B T}{6\pi\eta D} \qquad (5)$$

Where k_B is the Boltzman's constant; T is temperature (K); η is viscosity of solvent (Pa·s); D is the diffusion coefficient (m^2s^{-1}).

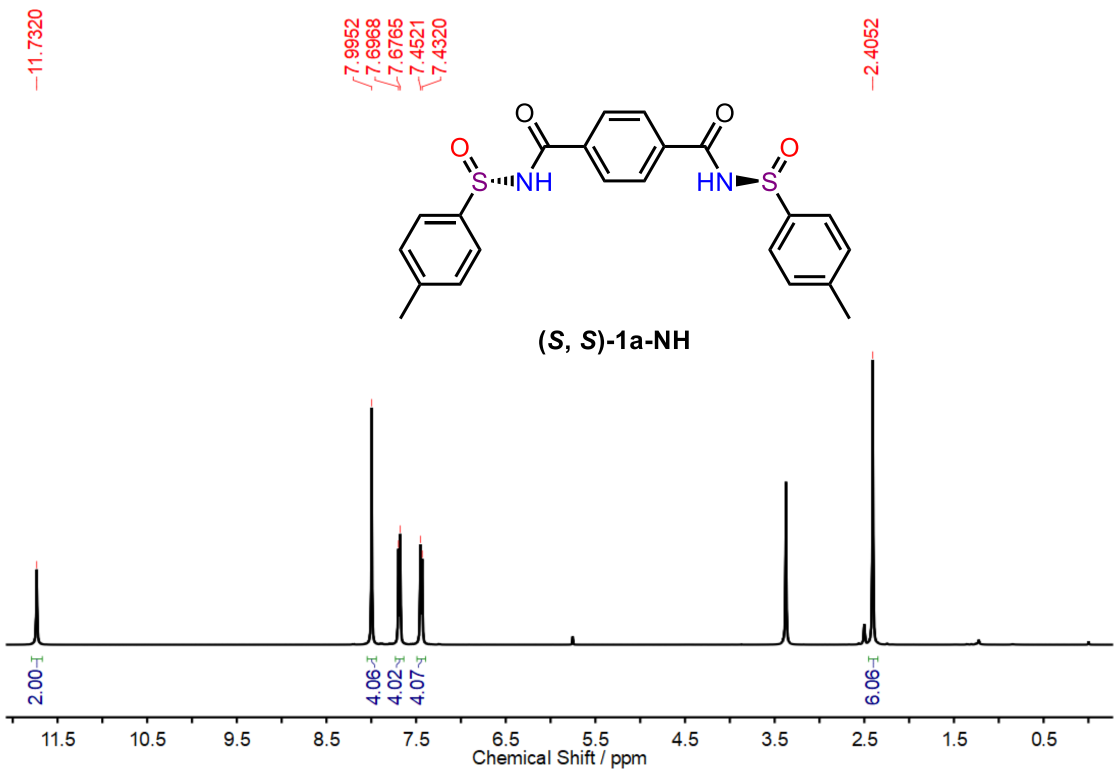

Figure S2. ^{1}H NMR (400 MHz) spectra of compound ***(S, S)*-1a-NH** ($(CD_3)_2SO$, 298 K).

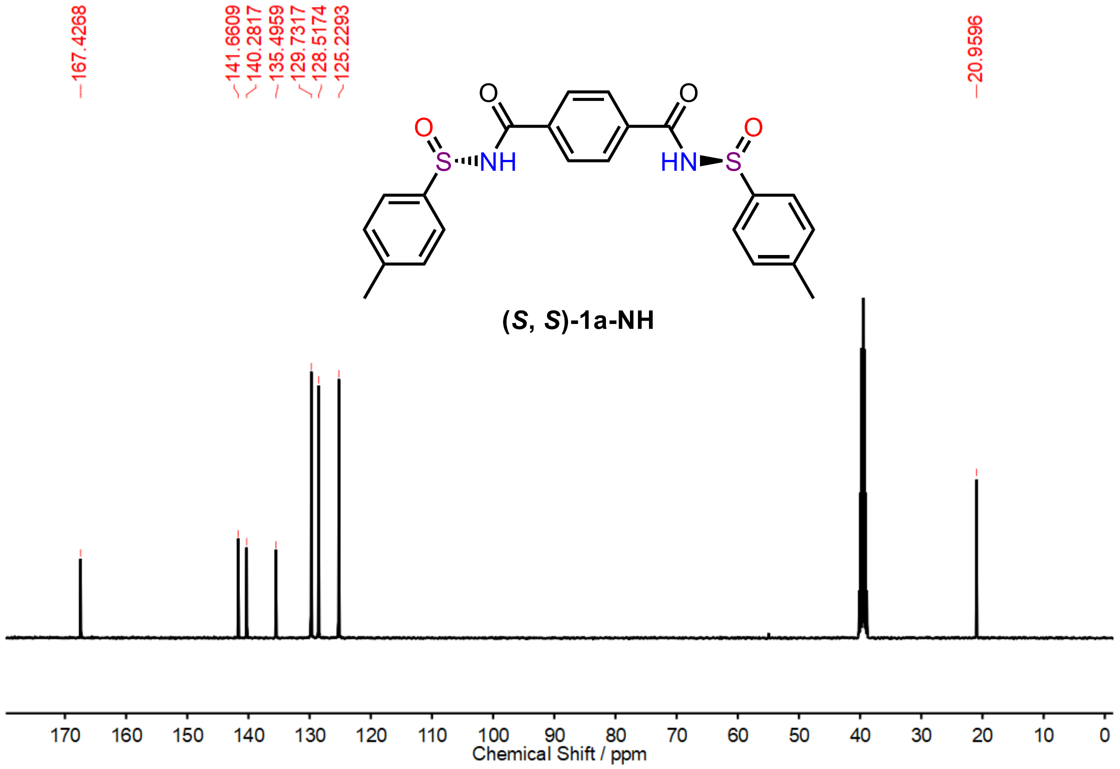

Figure S3. ^{13}C{1H} NMR (101 MHz) spectra of compound ***(S, S)*-1a-NH** ($(CD_3)_2SO$, 298 K).

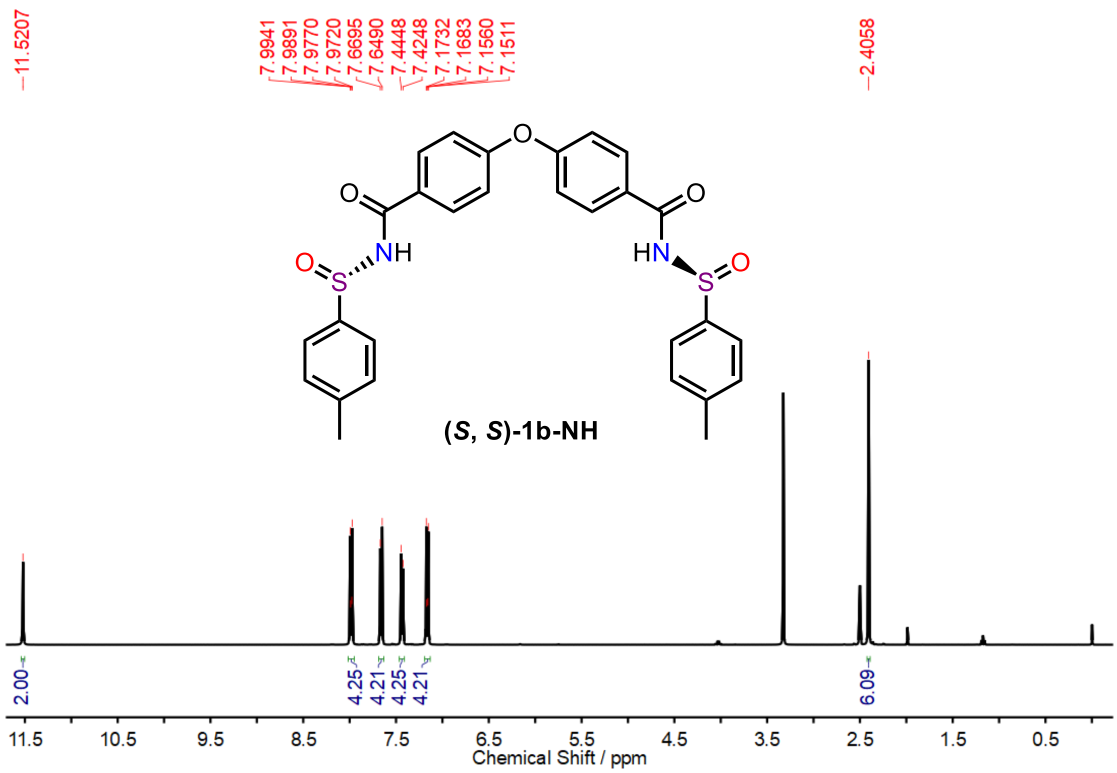

Figure S4. ^{1}H NMR (400 MHz) spectra of compound ***(S, S)*-1b-NH** ($(CD_3)_2SO$, 298 K).

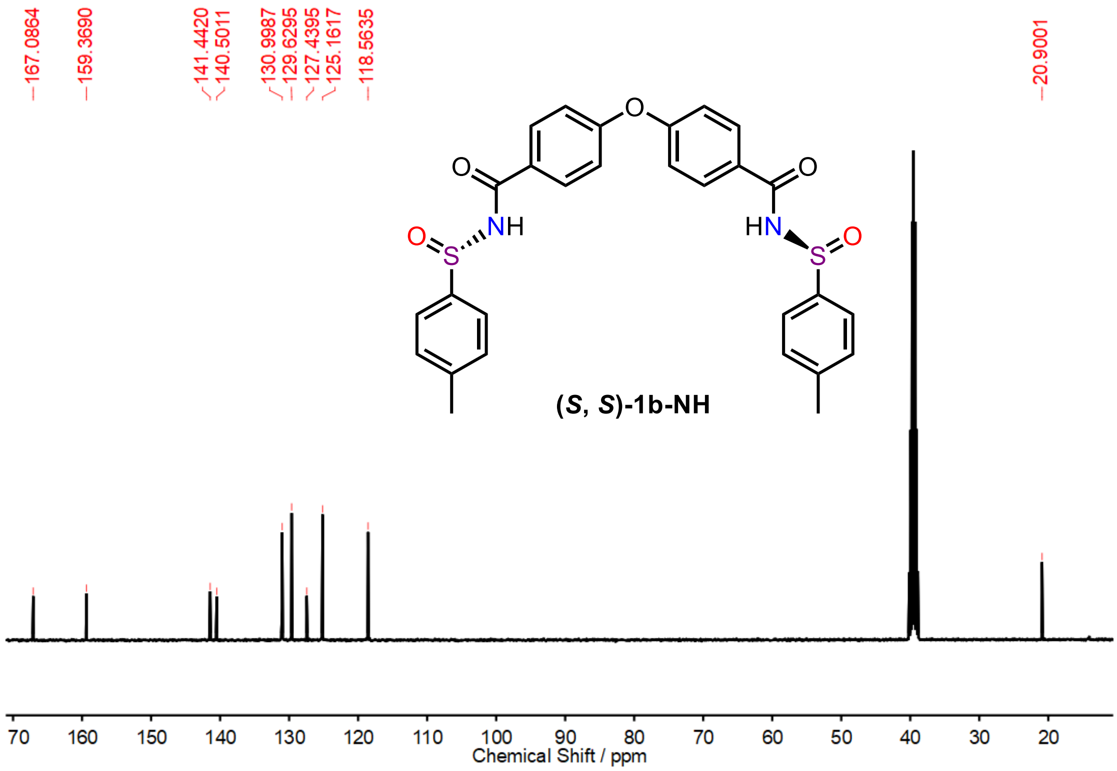

Figure S5. ^{13}C{1H} NMR (101 MHz) spectra of compound ***(S, S)*-1b-NH** ($(CD_3)_2SO$, 298 K).

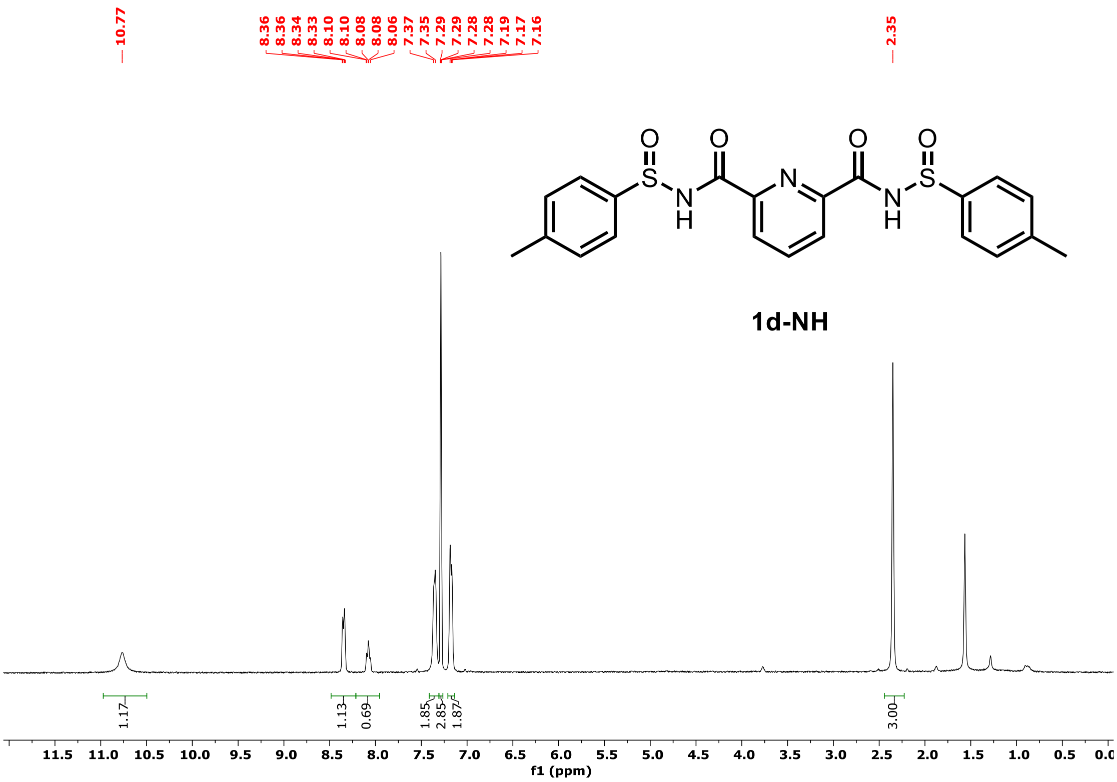

Figure S6. ^{1}H NMR (400 MHz) spectra of compound diastereomeric **1d-NH** ($CDCl_3$, 298 K).

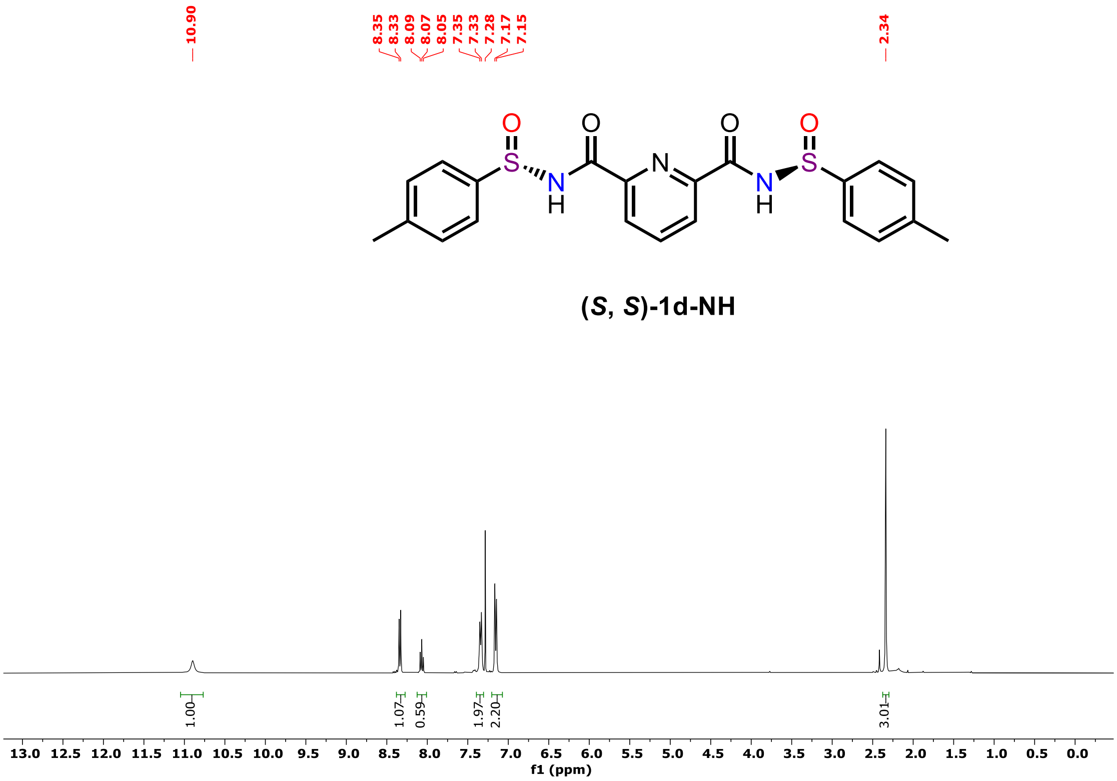

Figure S7. ^{1}H NMR (400 MHz) spectra of compound (***S, S***)-**1d-NH** ($CDCl_3$, 298 K).

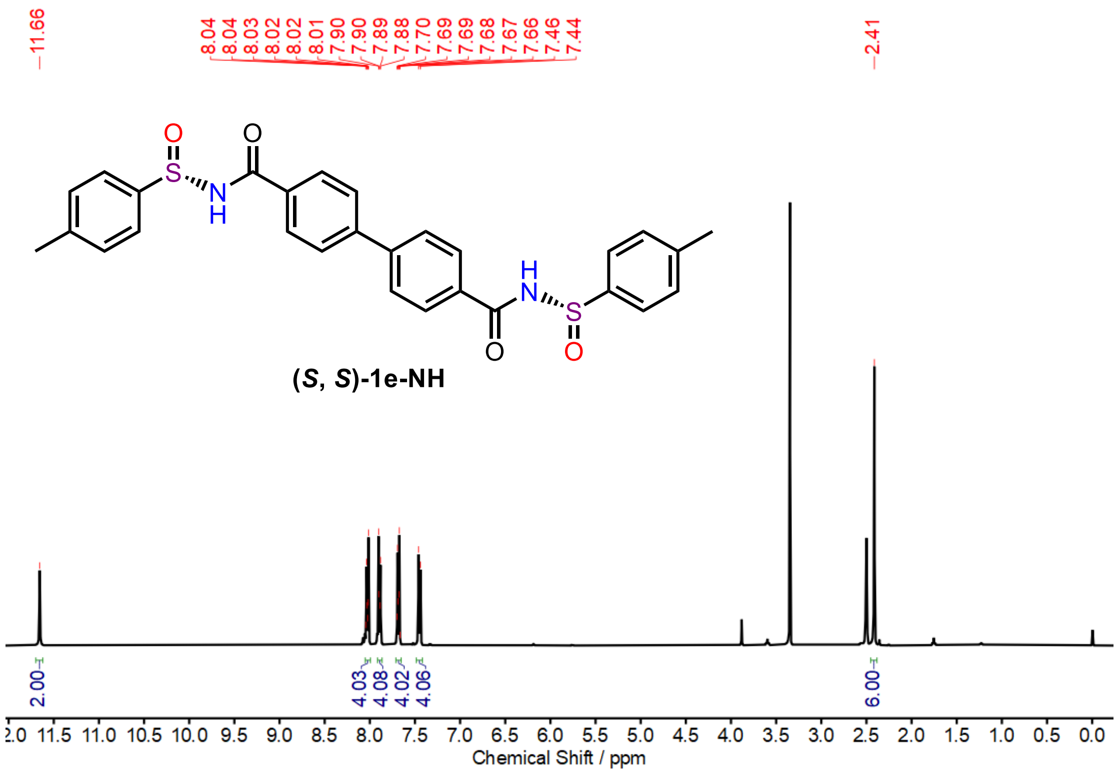

Figure S8. ^{1}H NMR (400 MHz) spectra of compound ***(S, S)*-1e-NH** (($(CD_3)_2SO$, 298 K).

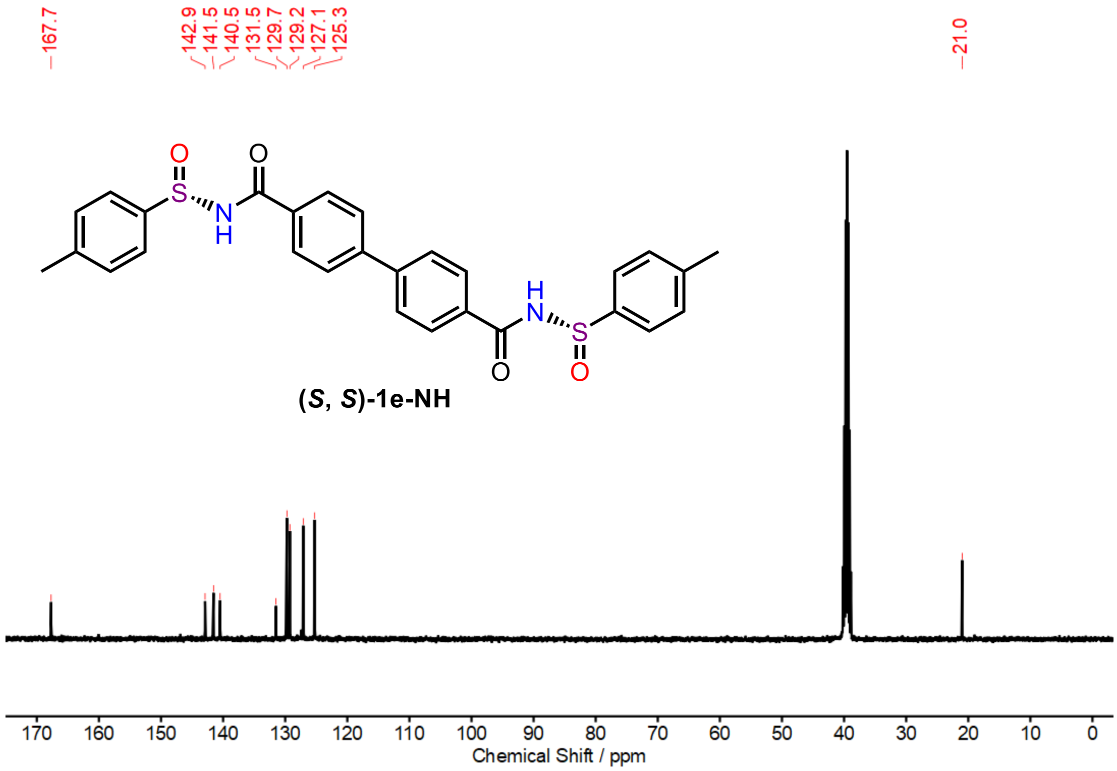

Figure S9. $^{13}C\{1H\}$ NMR (101 MHz) spectra of compound ***(S, S)*-1e-NH** ($(CD_3)_2SO$, 298 K).

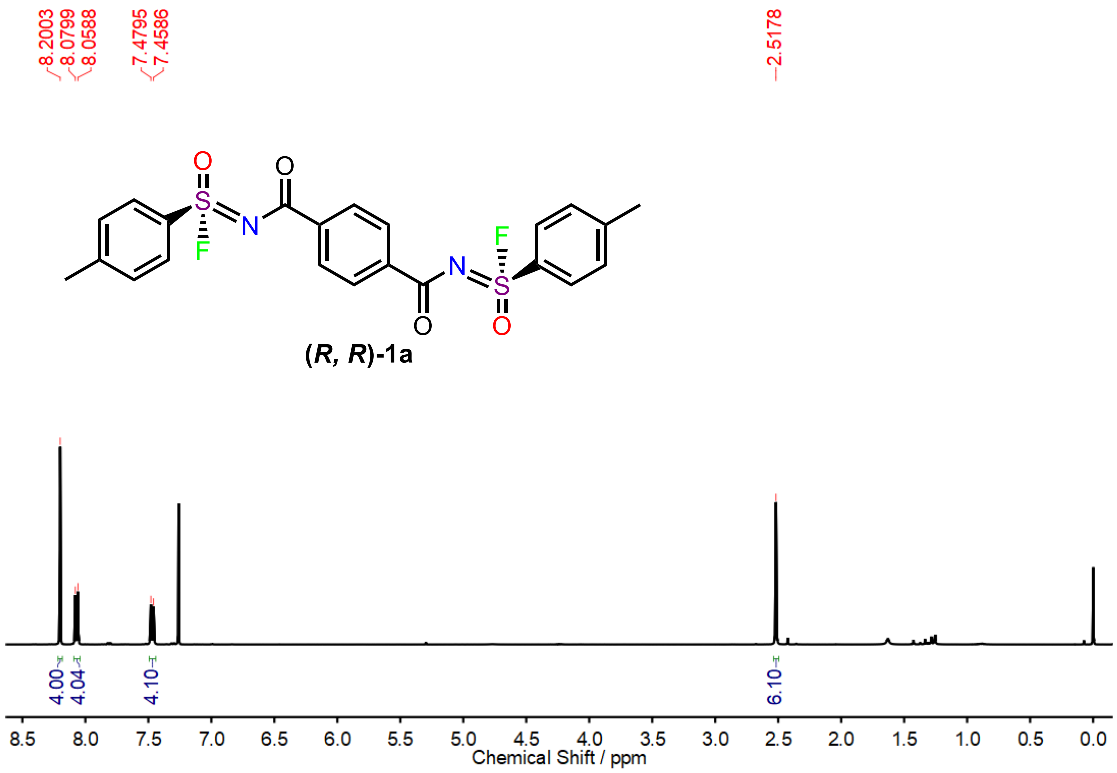

Figure S10. ^{1}H NMR (400 MHz) spectra of compound ***(R, R)*-1a** ($CDCl_3$, 298 K).

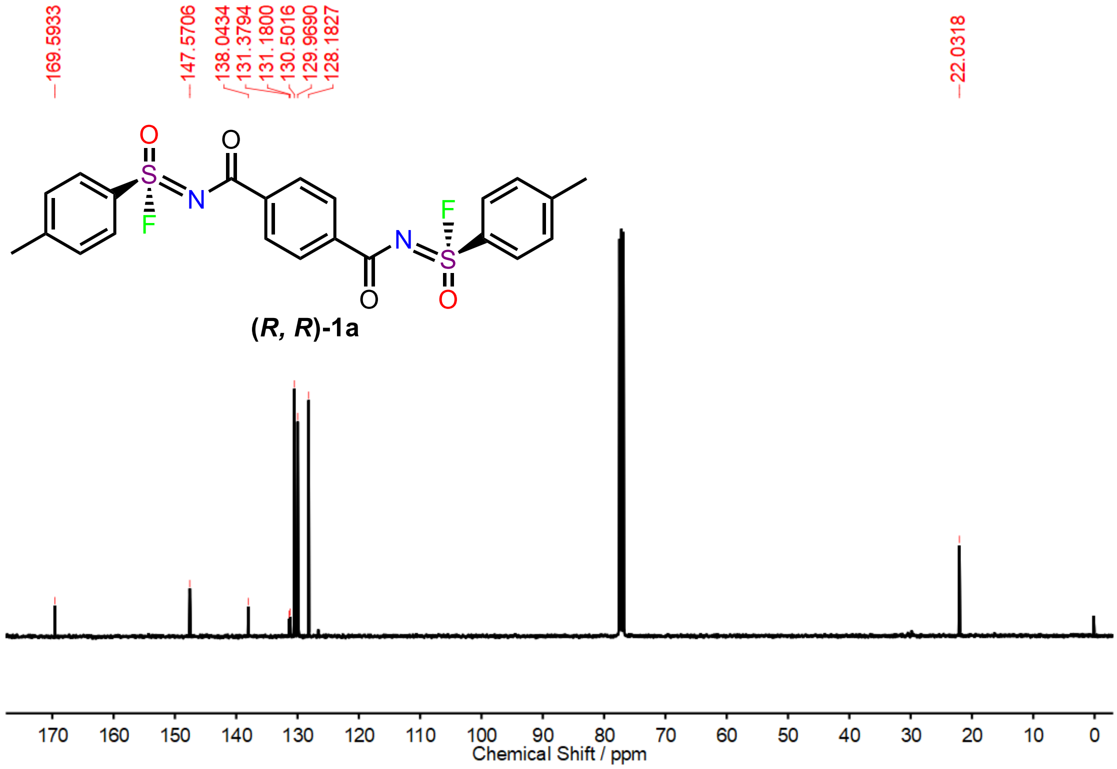

Figure S11. ^{13}C{1H} NMR (101 MHz) spectra of compound ***(R, R)*-1a** ($CDCl_3$, 298 K).

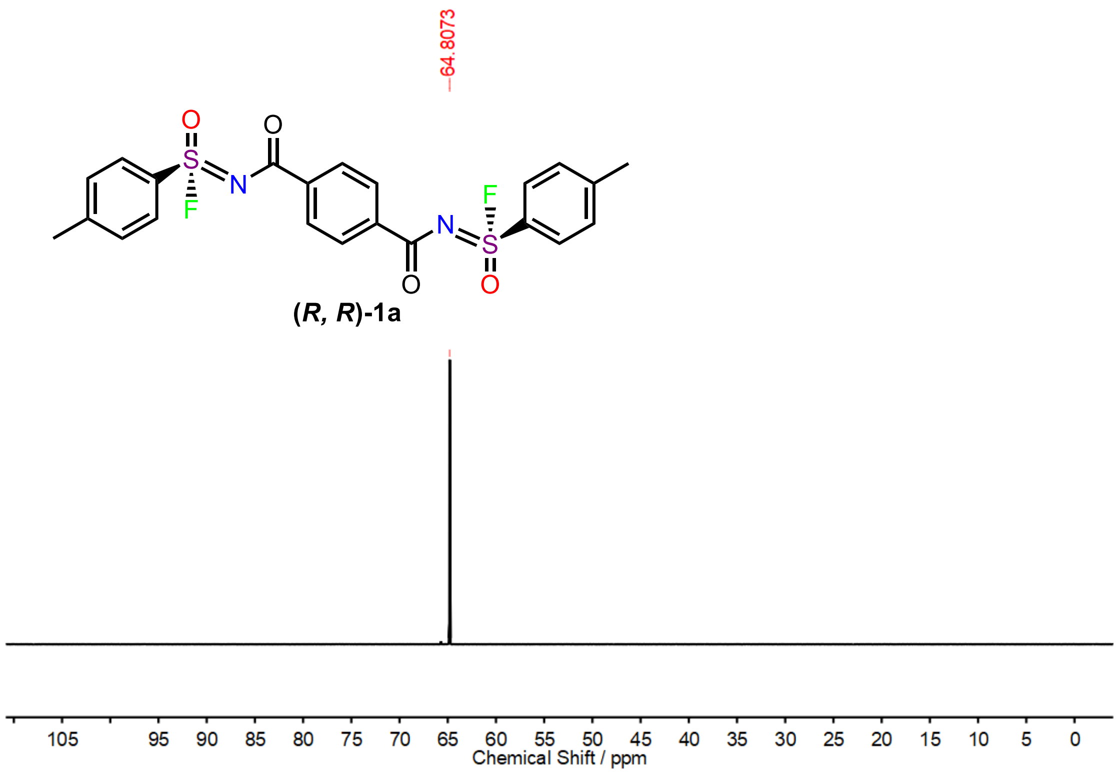

Figure S12. ^{19}F NMR (376 MHz) spectra of compound **(*R, R*)-1a** ($CDCl_3$, 298 K).

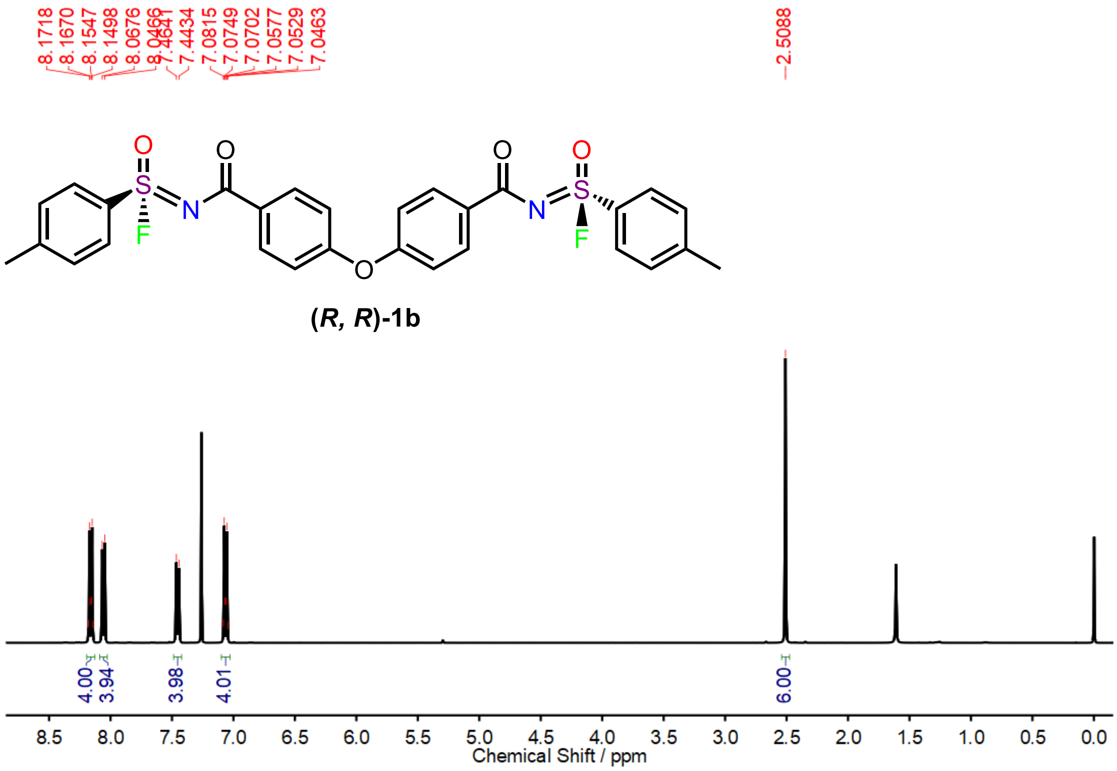

Figure S13. ^{1}H NMR (400 MHz) spectra of compound **(*R, R*)-1b** ($CDCl_3$, 298 K).

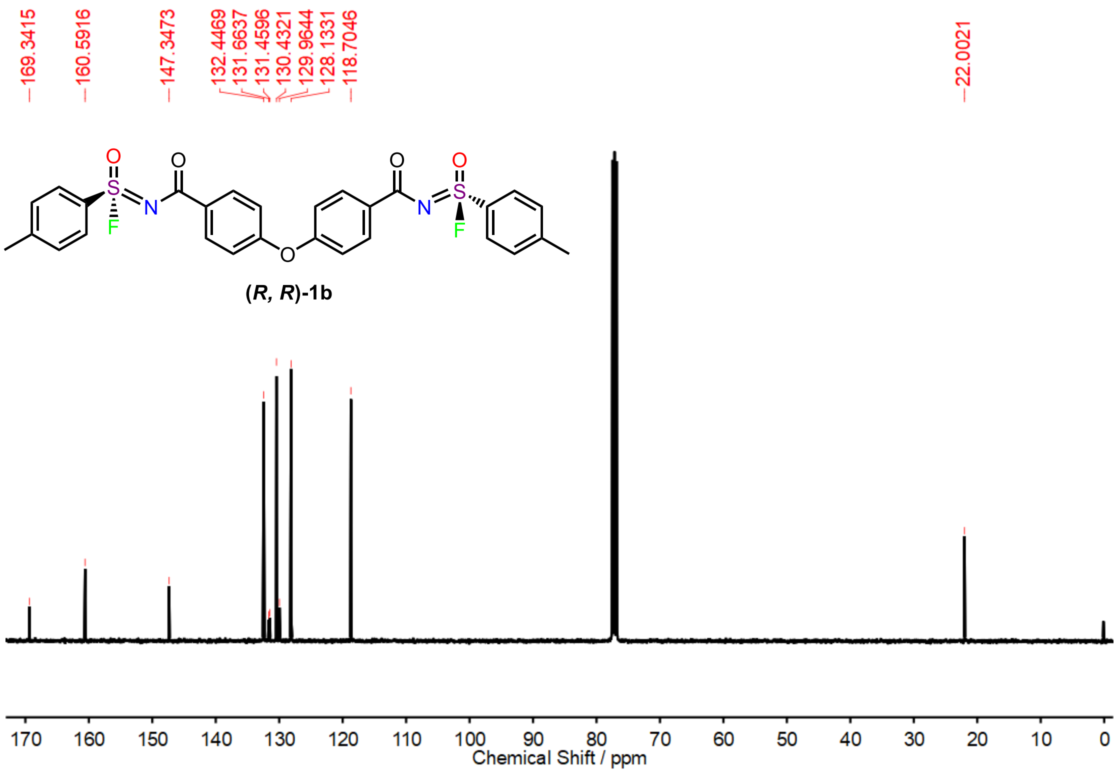

Figure S14. $^{13}C\{^1H\}$ NMR (101 MHz) spectra of compound **(*R*, *R*)-1b** ($CDCl_3$, 298 K).

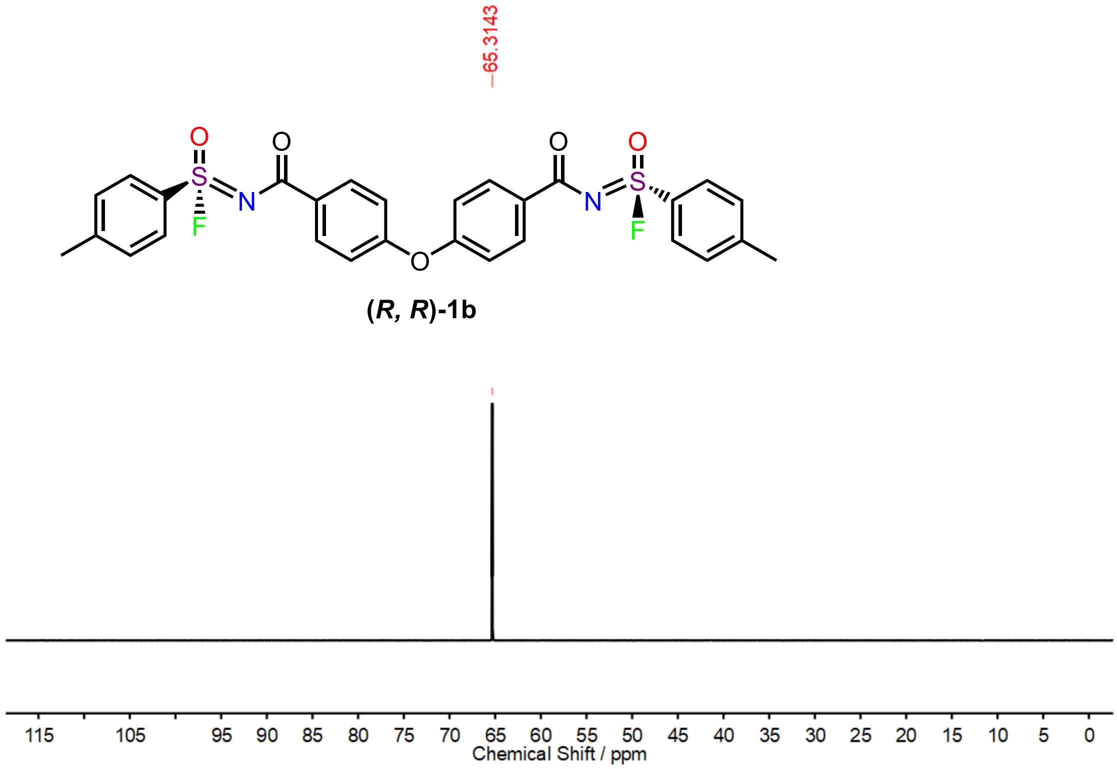

Figure S15. ^{19}F NMR (376 MHz) spectra of compound **(*R*, *R*)-1b** ($CDCl_3$, 298 K).

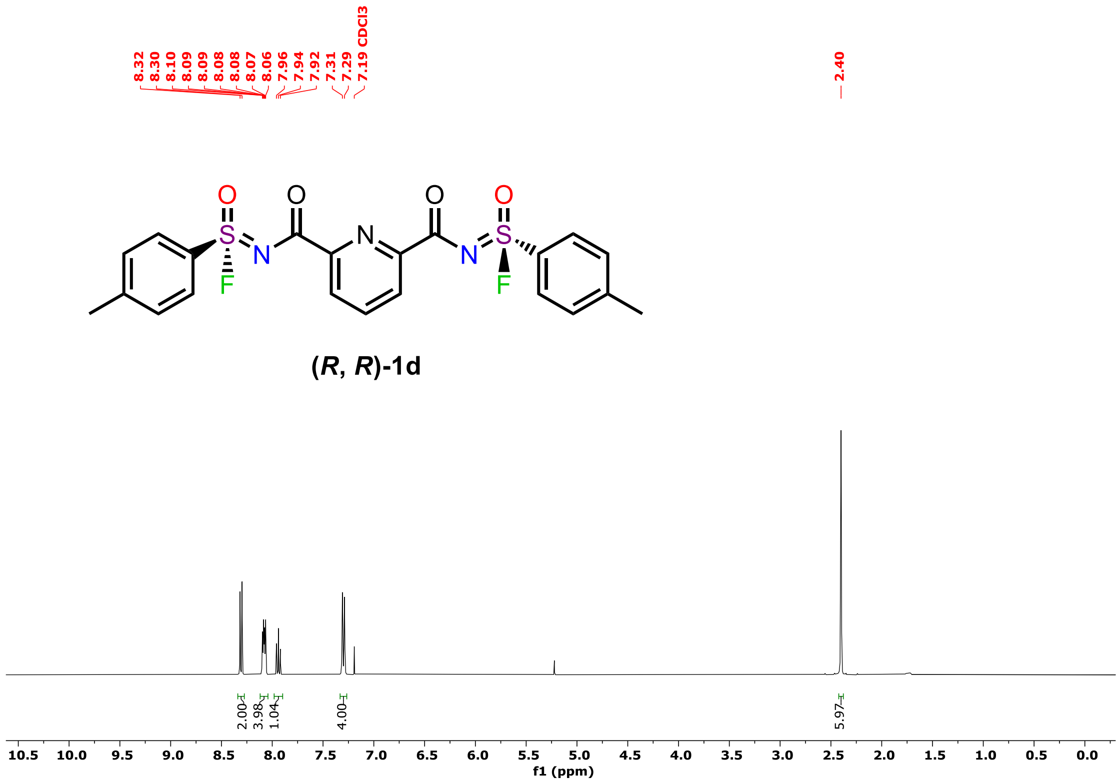

Figure S16. ^{1}H NMR (400 MHz) spectra of compound (***R*, *R***)-**1d** ($CDCl_3$, 298 K).

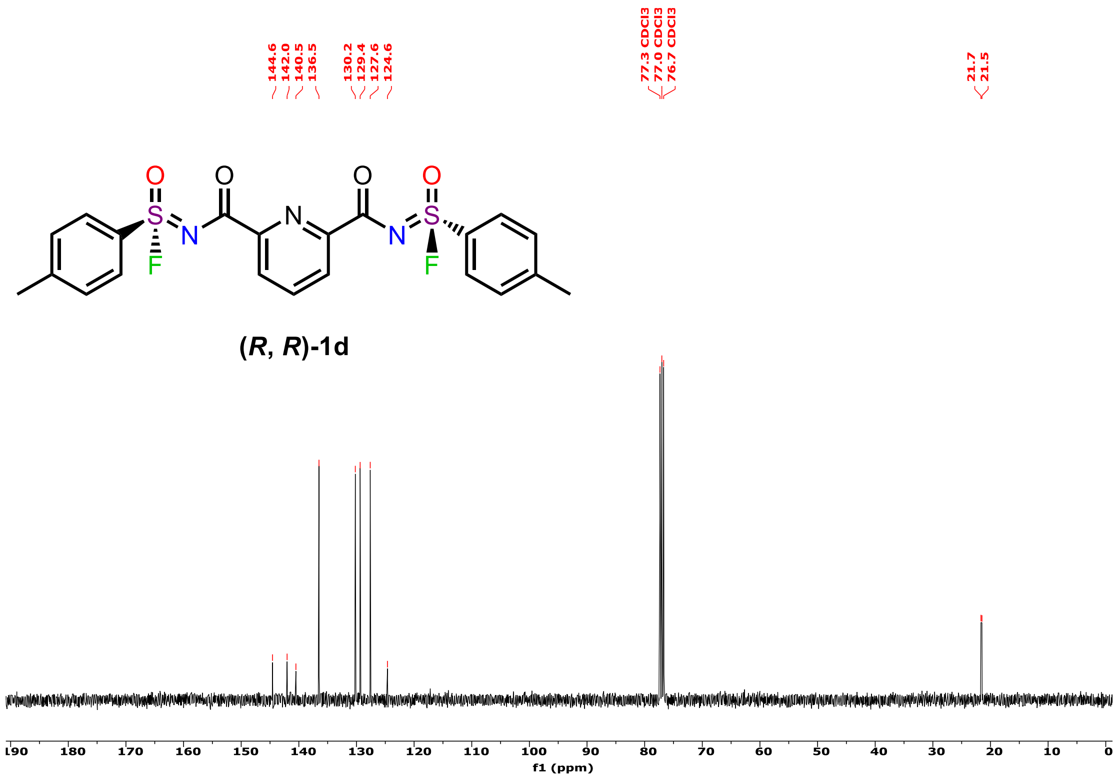

Figure S17. ^{1}H NMR (101 MHz) spectra of compound (***R*, *R***)-**1d** ($CDCl_3$, 298 K).

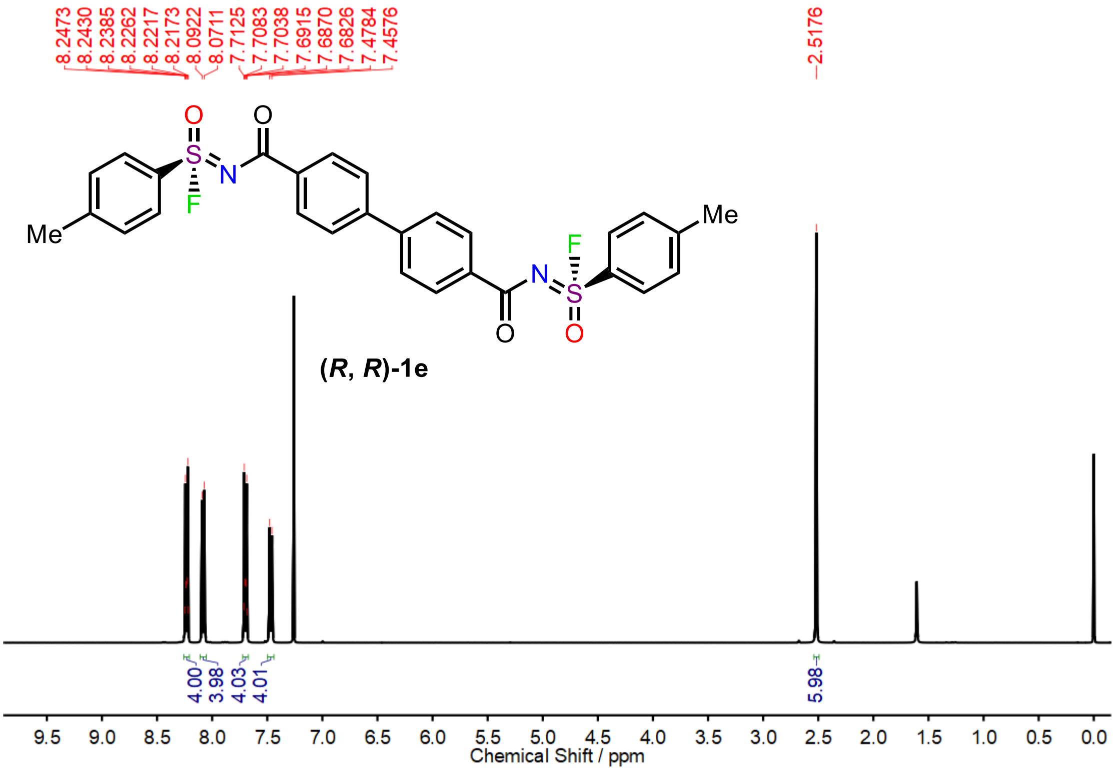

Figure S18. ^{1}H NMR (400 MHz) spectra of compound ***(R, R)*-1e** ($CDCl_3$, 298 K).

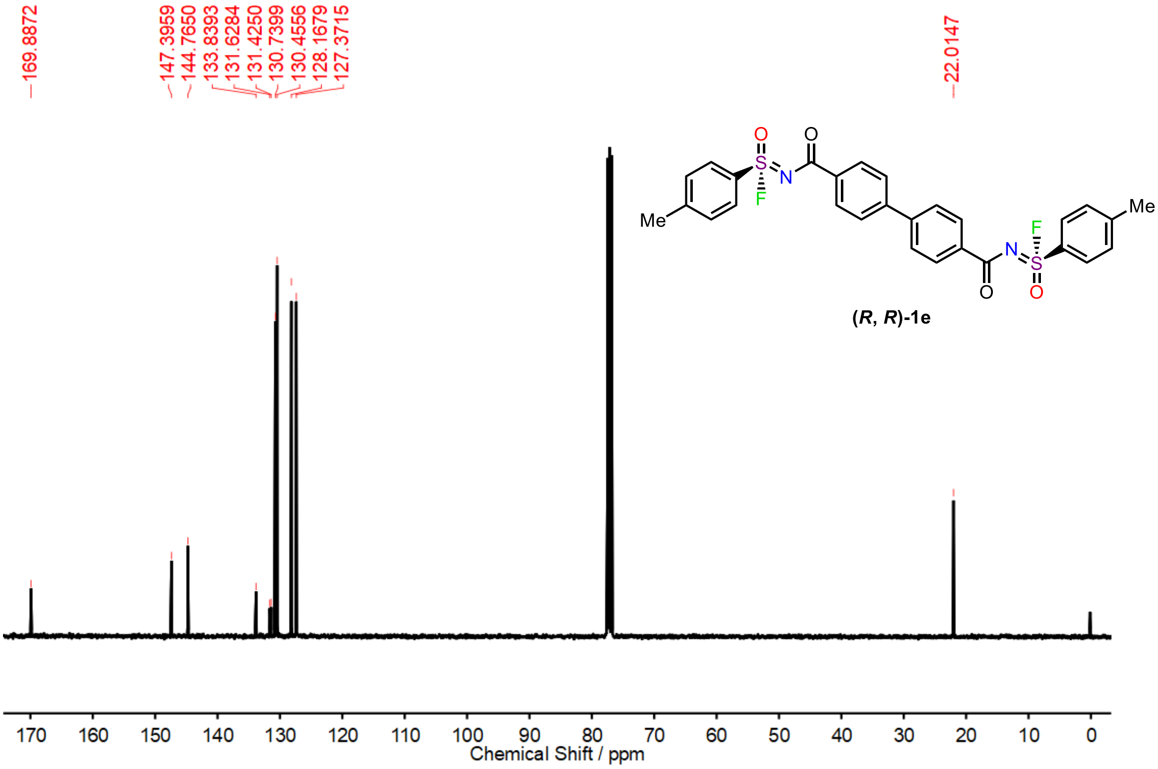

Figure S19. ^{13}C{1H} NMR (101 MHz) spectra of compound ***(R, R)*-1e** ($CDCl_3$, 298 K).

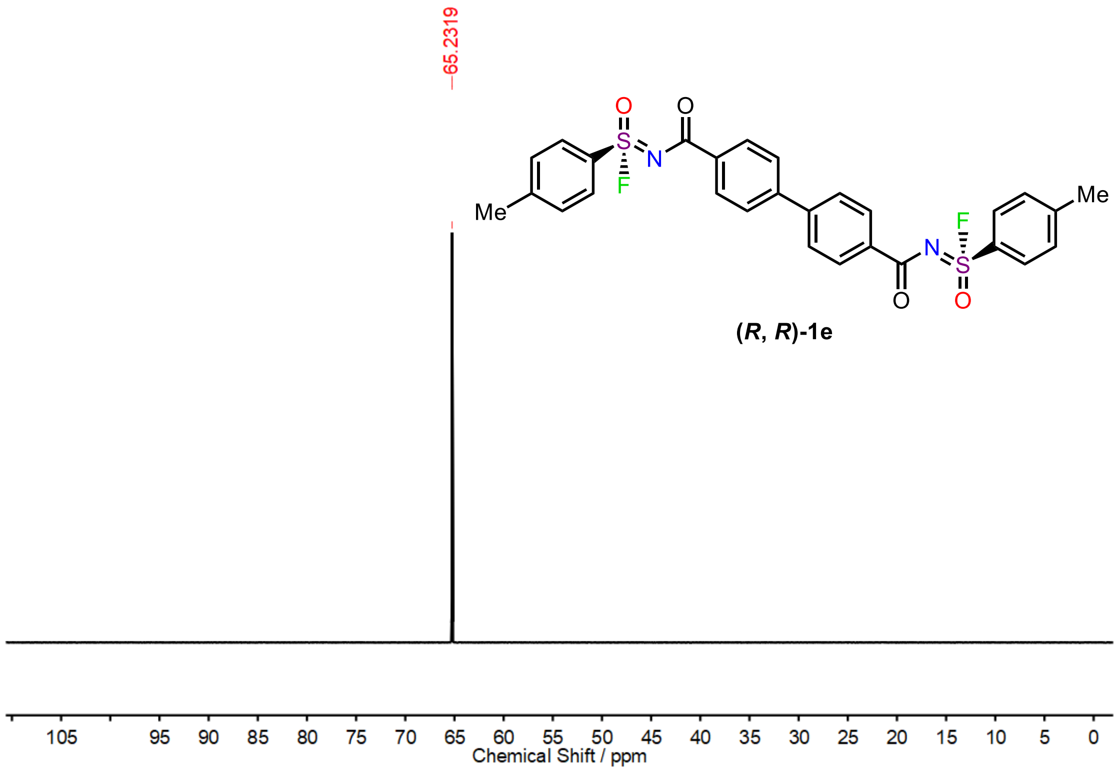

Figure S20. ^{19}F NMR (376 MHz) spectra of compound **(*R, R*)-1e** ($CDCl_3$, 298 K).

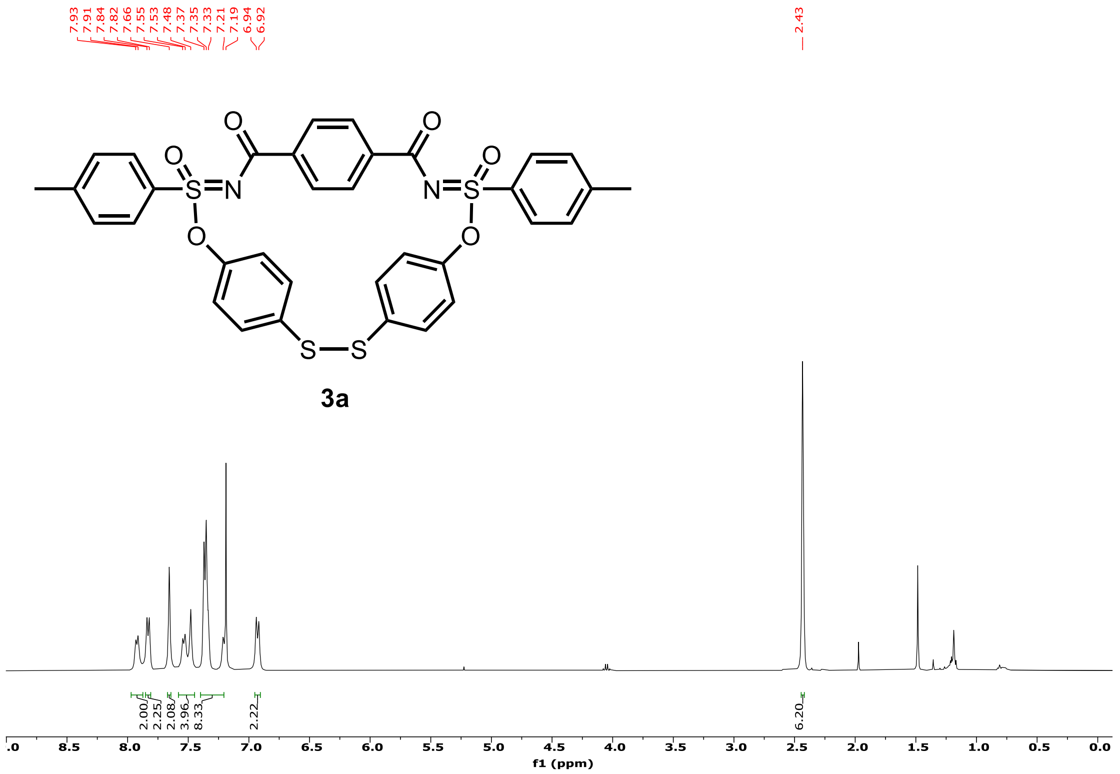

Figure S21. ^{1}H NMR (400 MHz) spectra of compound **3a** ($CDCl_3$, 298 K).

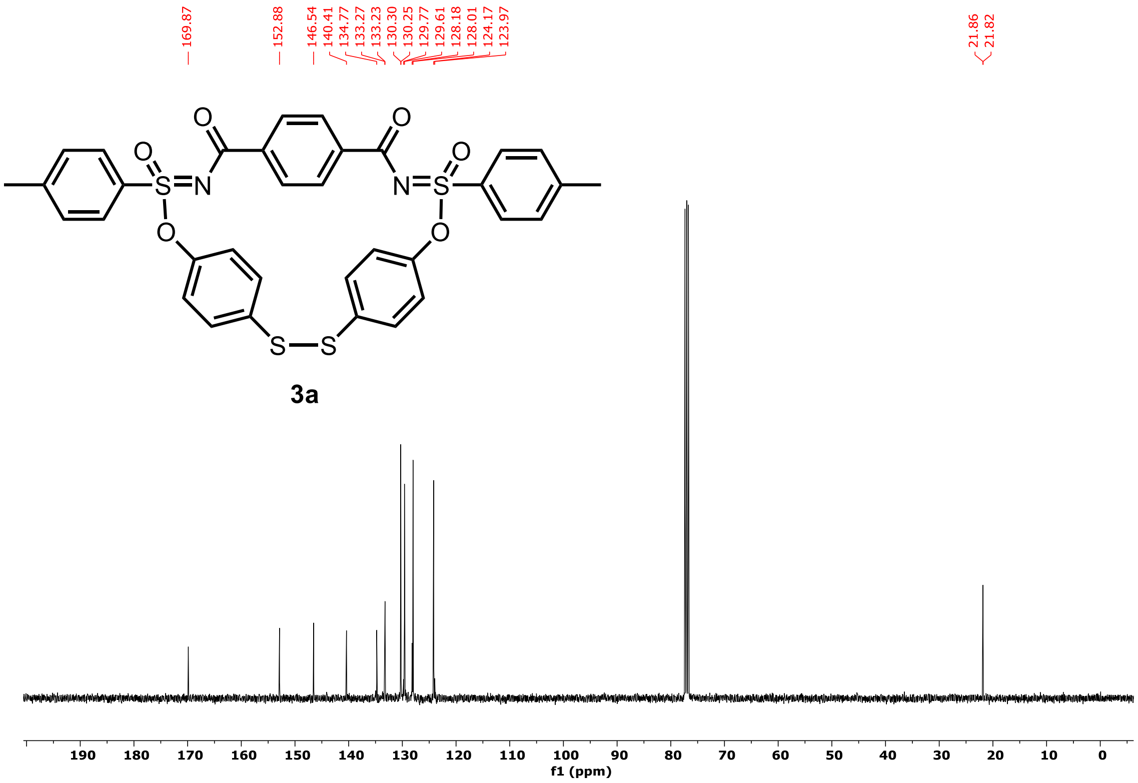

Figure S22. $^{13}C\{1H\}$ NMR (101 MHz) spectra of compound **3a** ($CDCl_3$, 298 K).

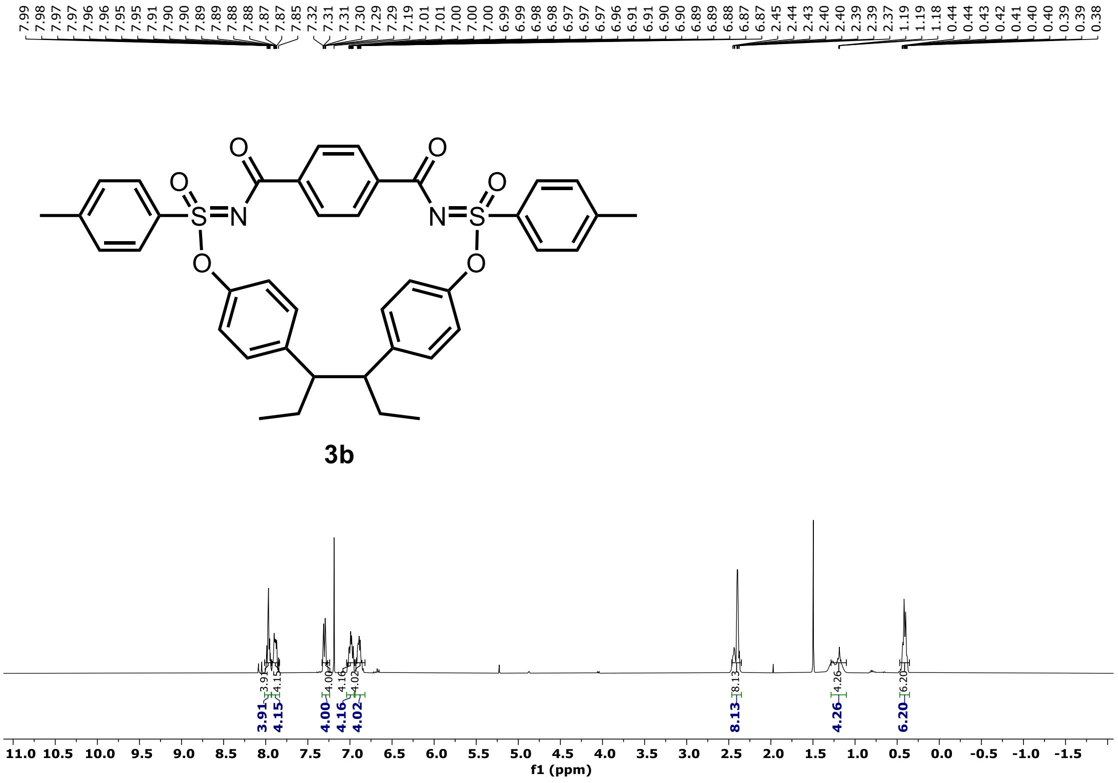

Figure S23. ^{1}H NMR (400 MHz) spectra of compound **3b** ($CDCl_3$, 298 K).

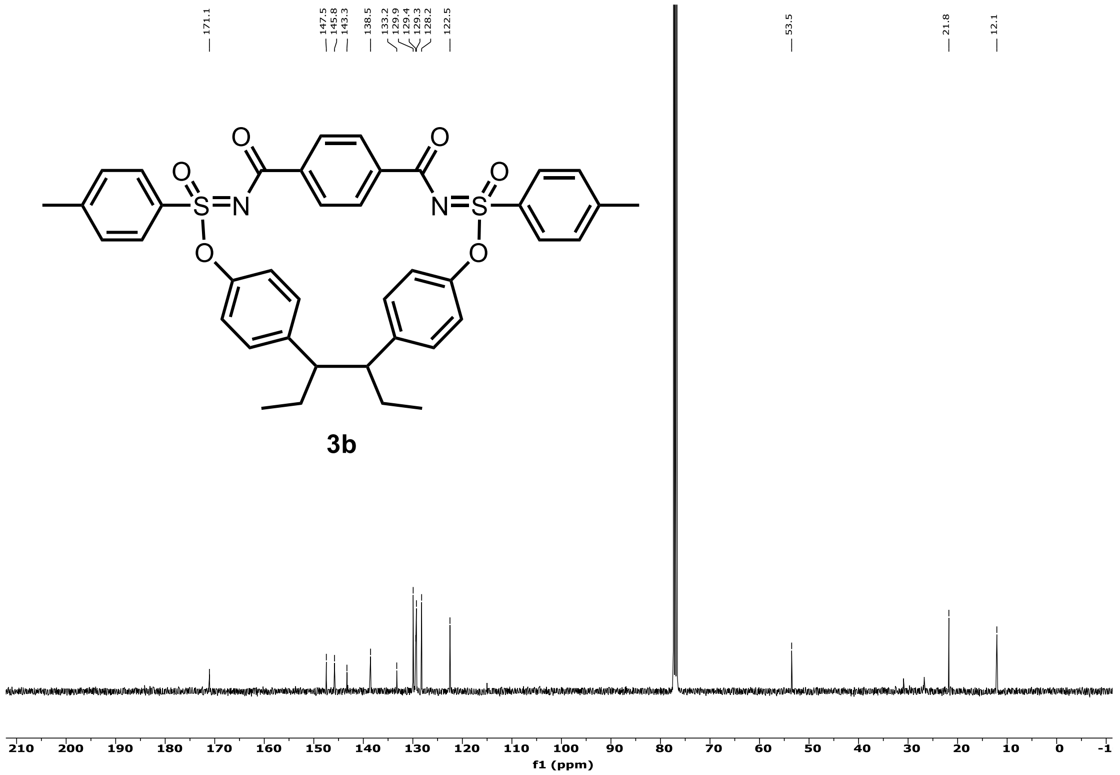

Figure S24. $^{13}C\{1H\}$ NMR (101 MHz) spectra of compound **3b** ($CDCl_3$, 298 K).

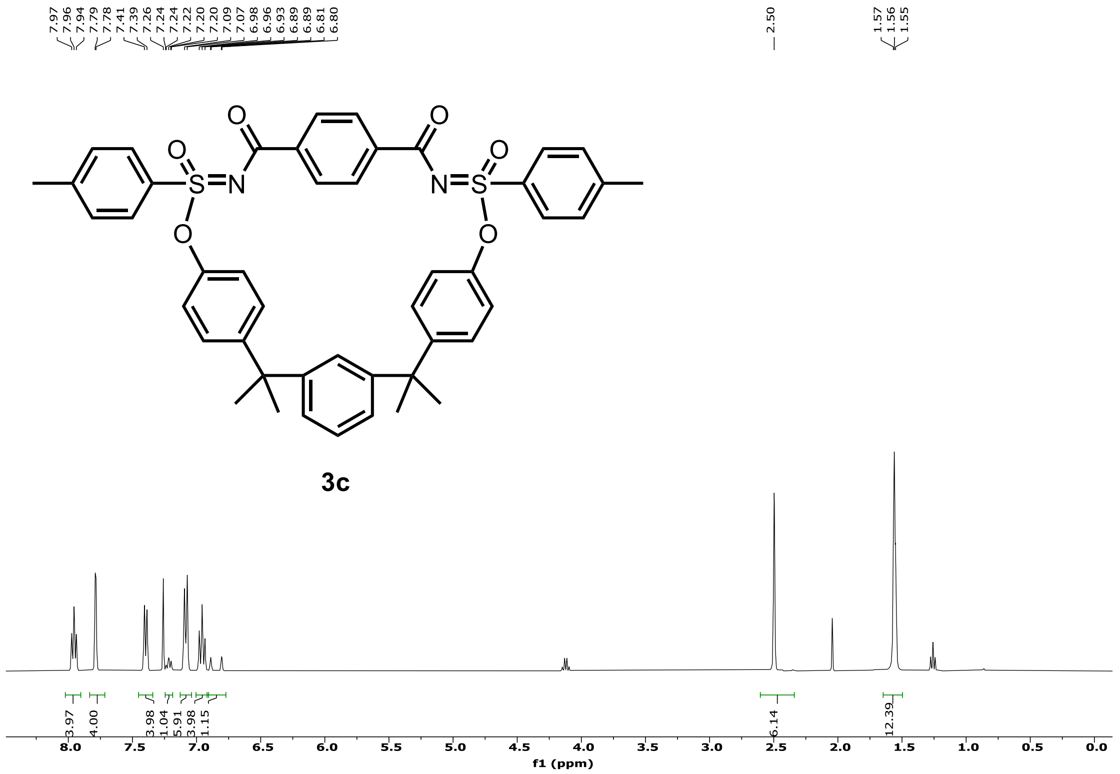

Figure S25. ^{1}H NMR (400 MHz) spectra of compound **3c** ($CDCl_3$, 298 K).

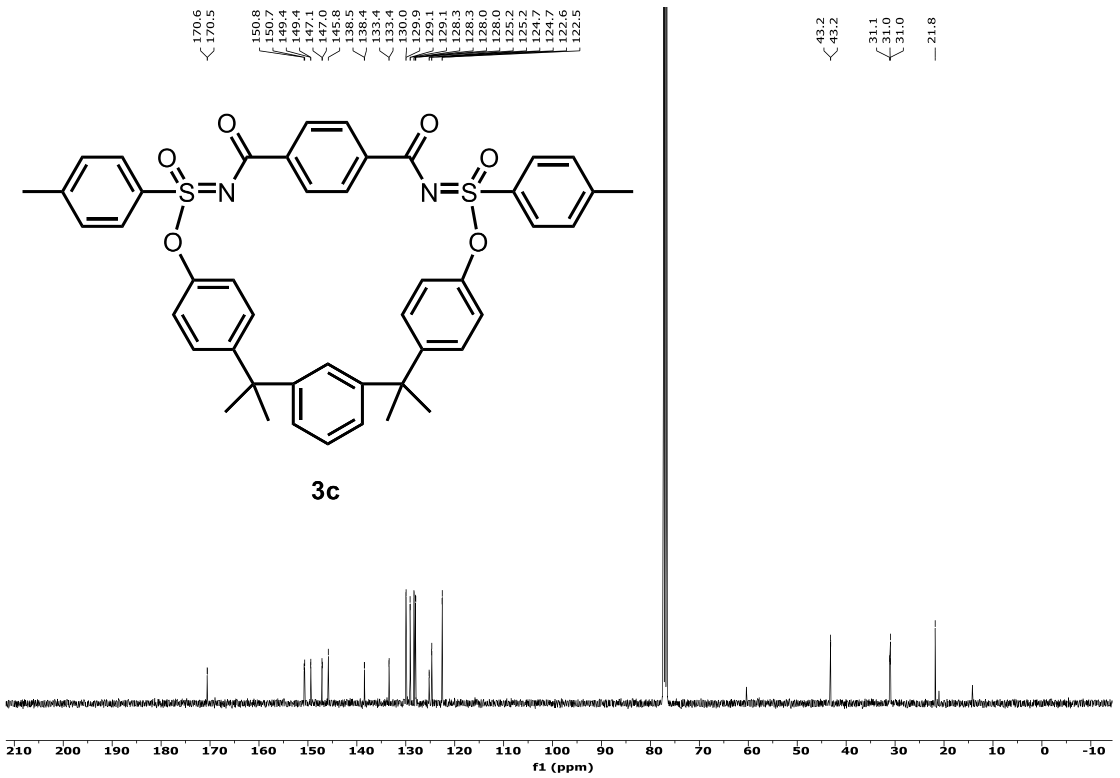

Figure S26. $^{13}C\{1H\}$ NMR (101 MHz) spectra of compound **3c** ($CDCl_3$, 298 K).

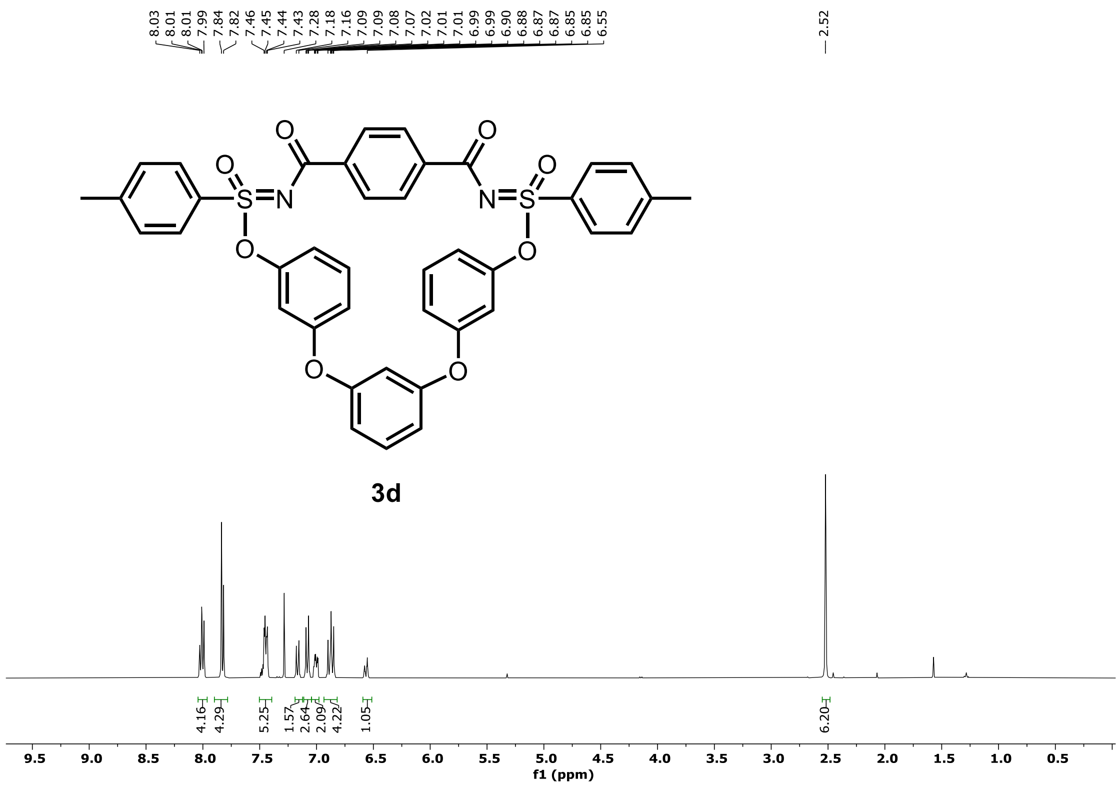

Figure S27. ^{1}H NMR (400 MHz) spectra of compound **3d** ($CDCl_3$, 298 K).

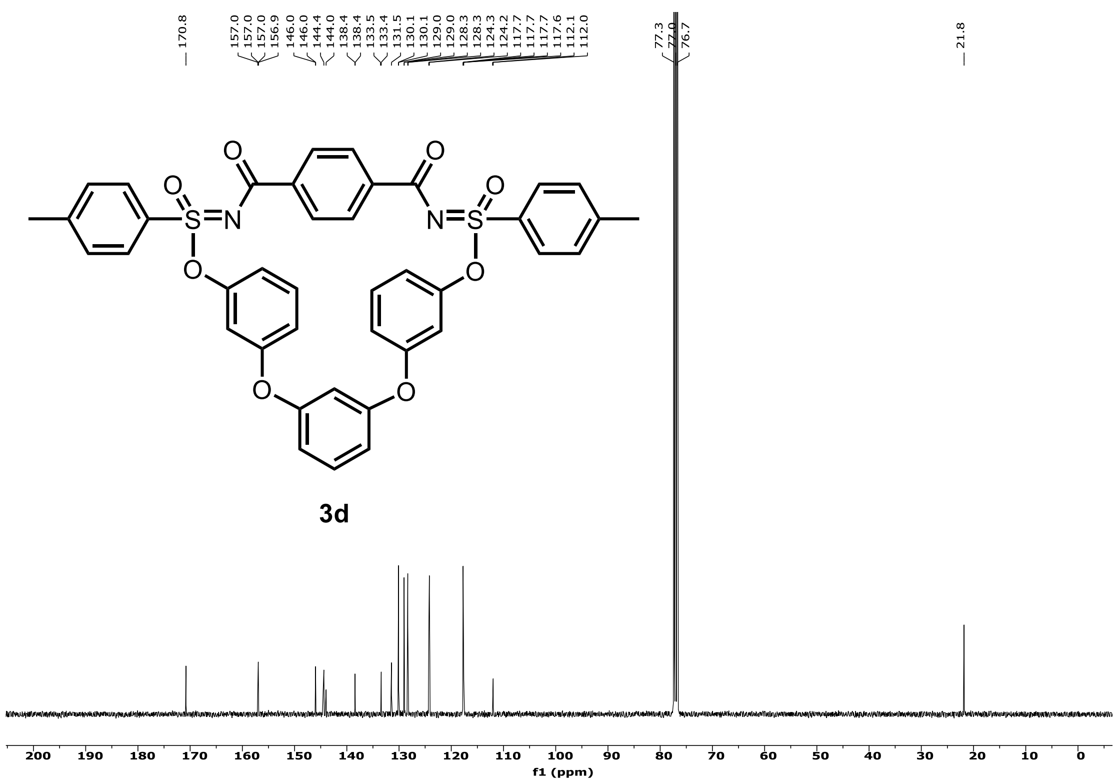

3d

Figure S28. $^{13}C\{1H\}$ NMR (101 MHz) spectra of compound **3d** ($CDCl_3$, 298 K).

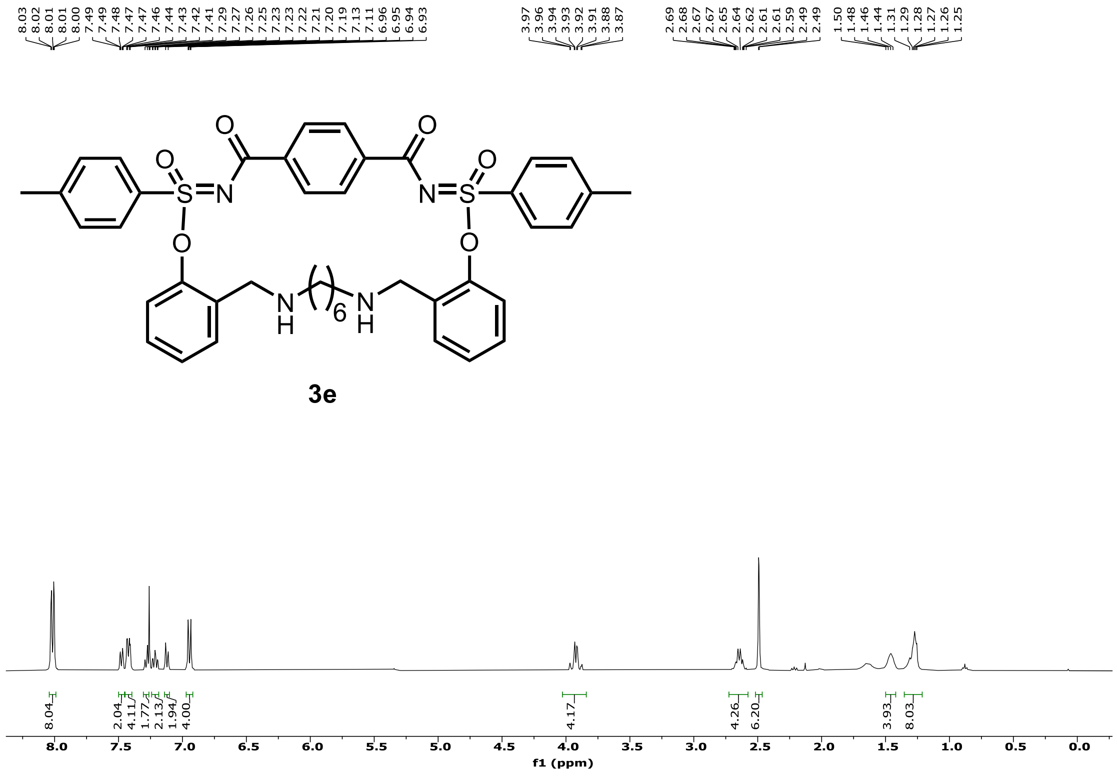

3e

Figure S29. ^{1}H NMR (400 MHz) spectra of compound **3e** ($CDCl_3$, 298 K).

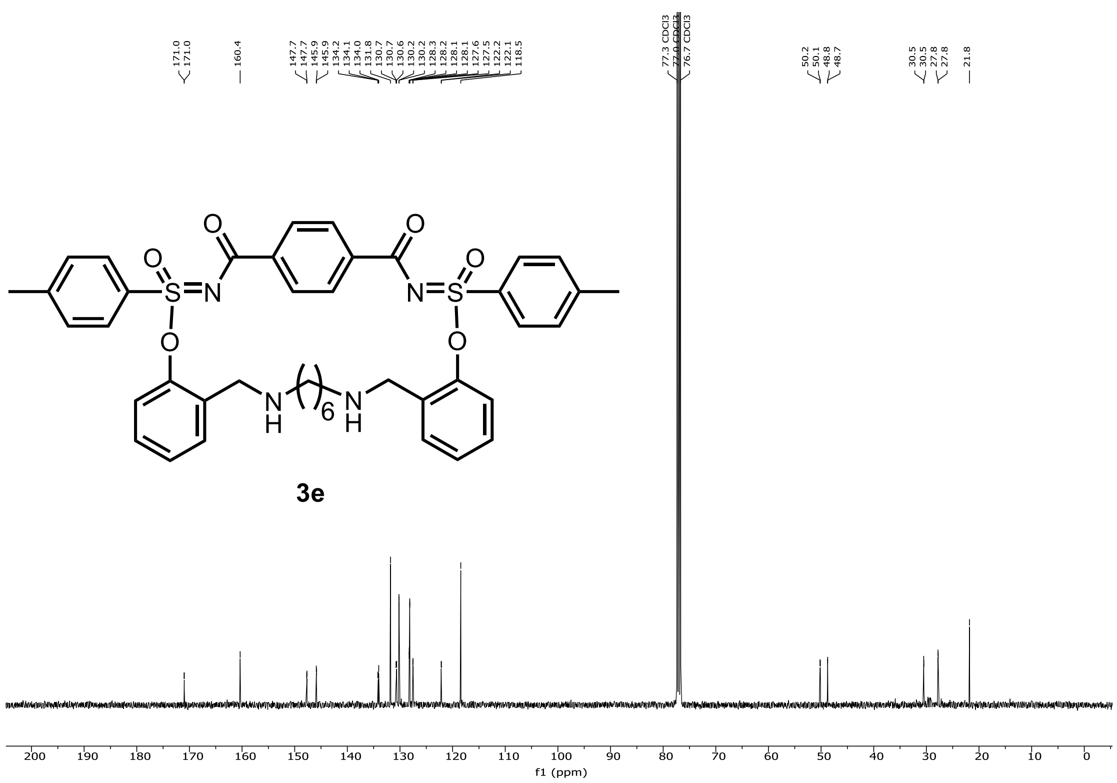

Figure S30. $^{13}C\{^1H\}$ NMR (101 MHz) spectra of compound **3e** ($CDCl_3$, 298 K).

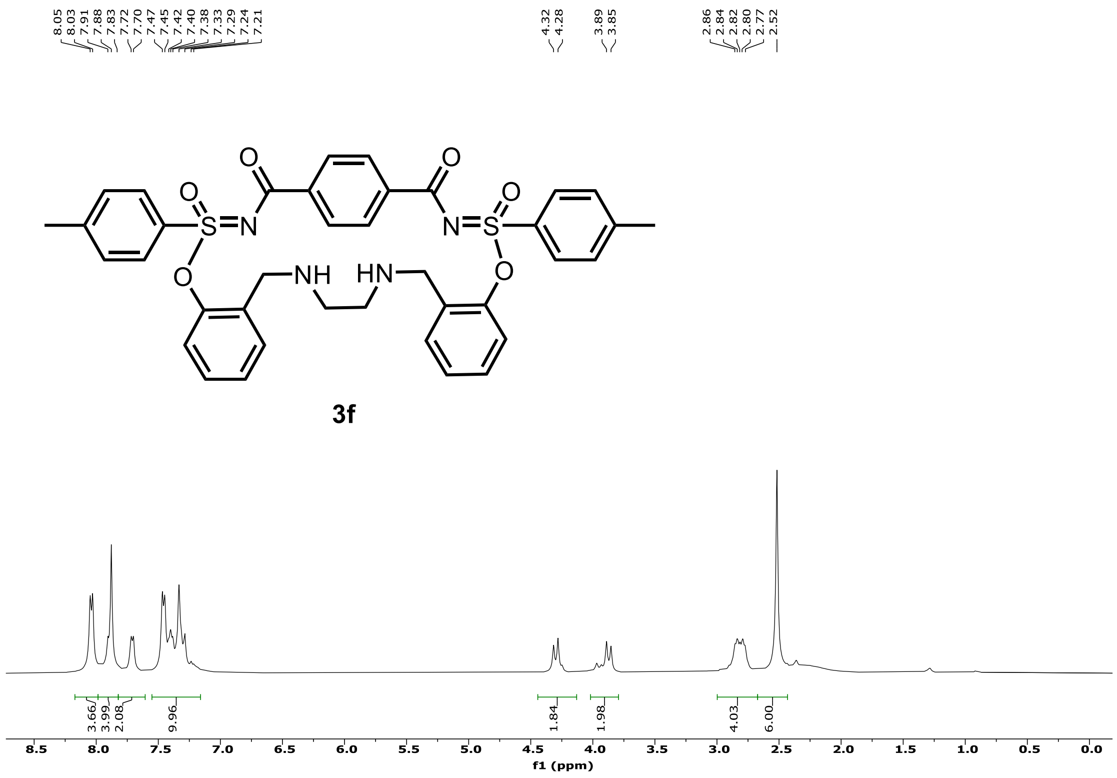

Figure S31. ^1H NMR (400 MHz) spectra of compound **3f** ($CDCl_3$, 298 K).

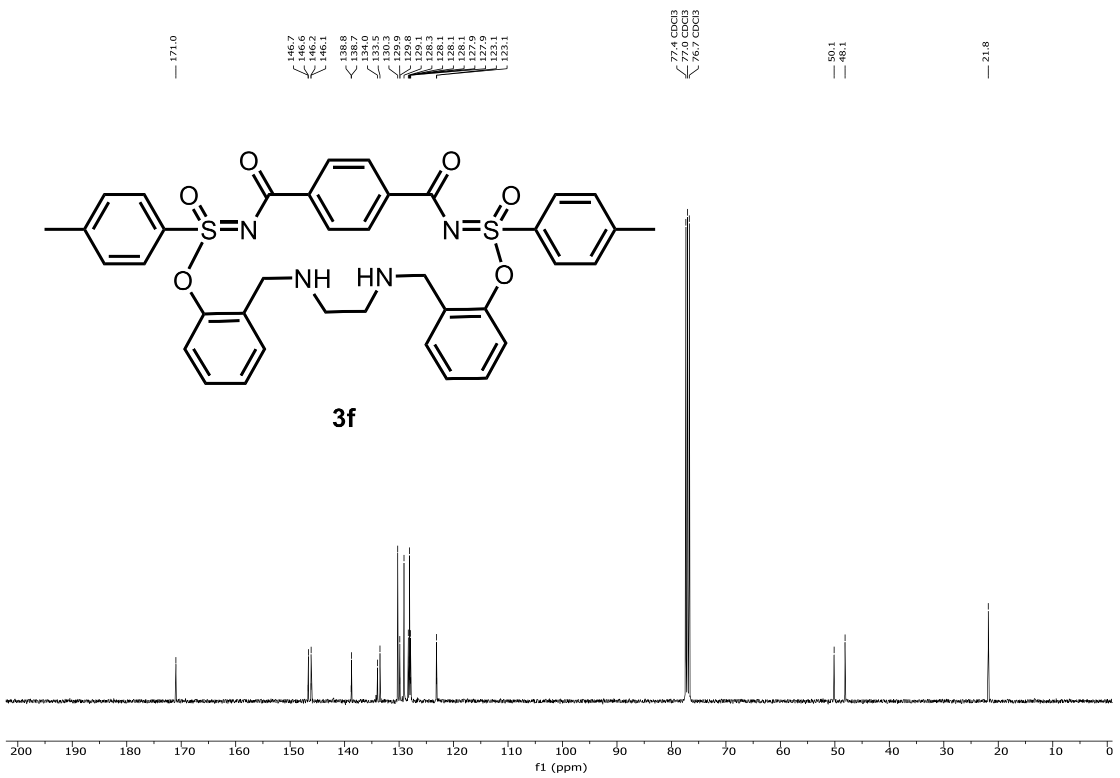

Figure S32. $^{13}C\{1H\}$ NMR (101 MHz) spectra of compound **3f** ($CDCl_3$, 298 K).

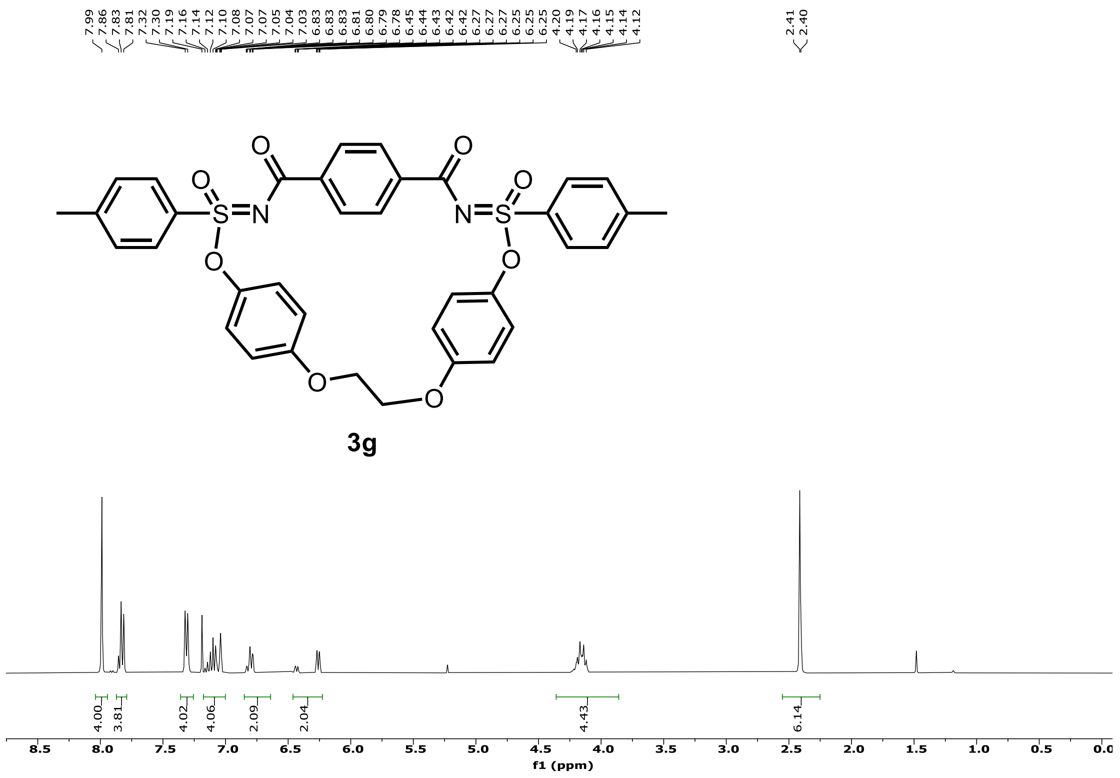

Figure S33. ^{1}H NMR (400 MHz) spectra of compound **3g** ($CDCl_3$, 298 K).

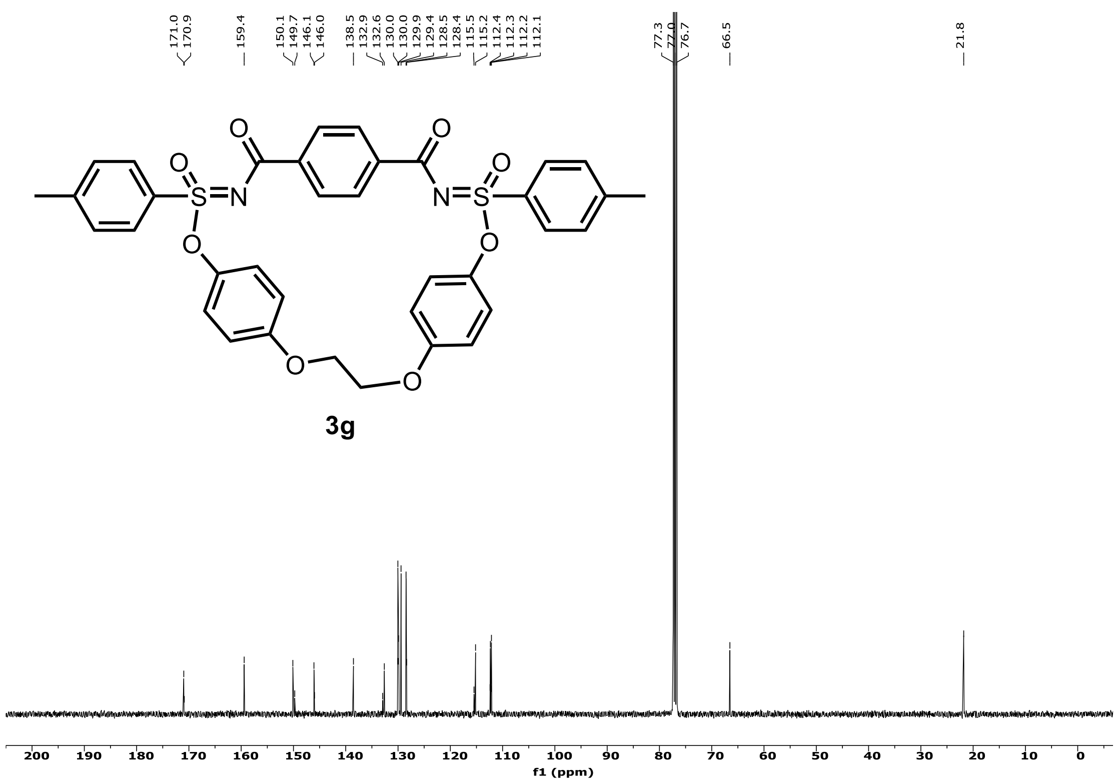

Figure S34. $^{13}C\{1H\}$ NMR (101 MHz) spectra of compound **3g** ($CDCl_3$, 298 K).

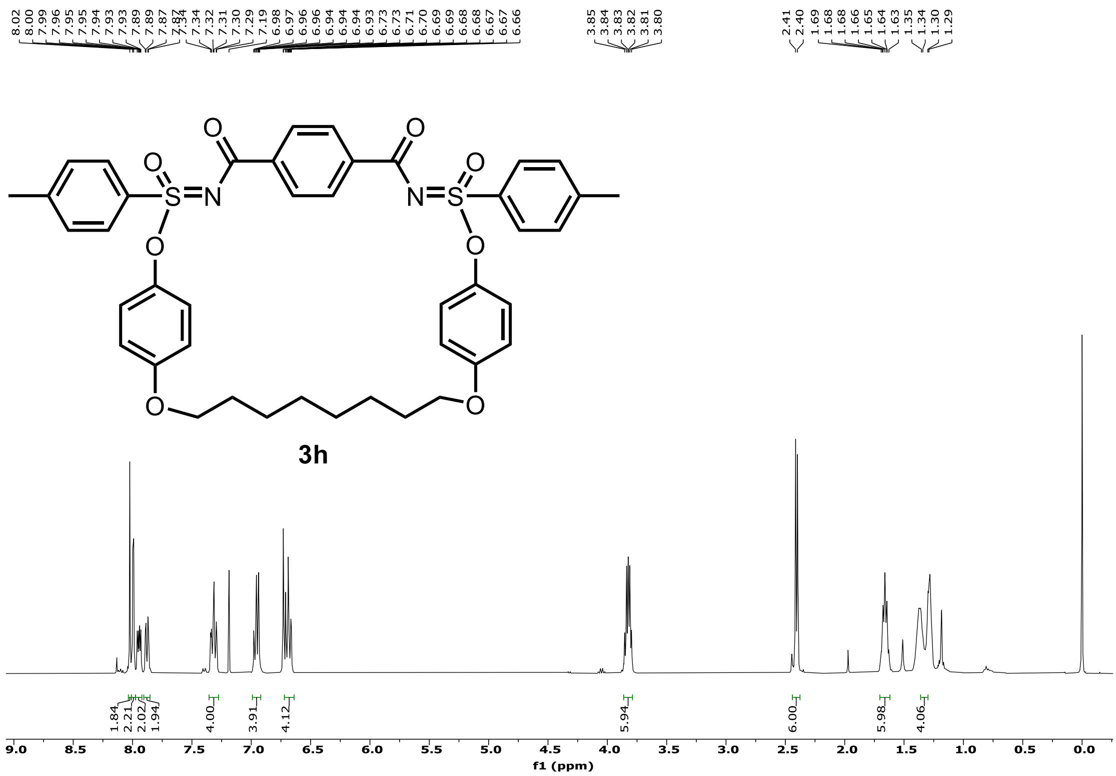

Figure S35. ^{1}H NMR (400 MHz) spectra of compound **3h** ($CDCl_3$, 298 K).

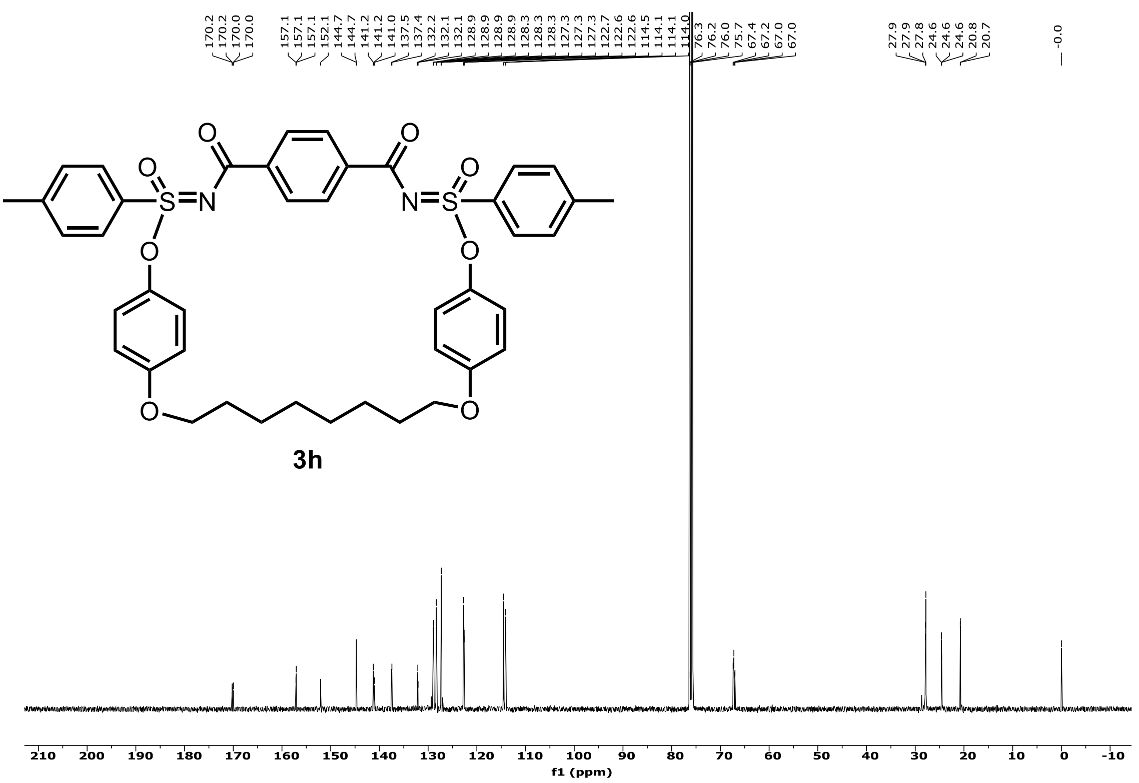

Figure S36. ^{13}C{1H} NMR (101 MHz) spectra of compound **3h** ($CDCl_3$, 298 K).

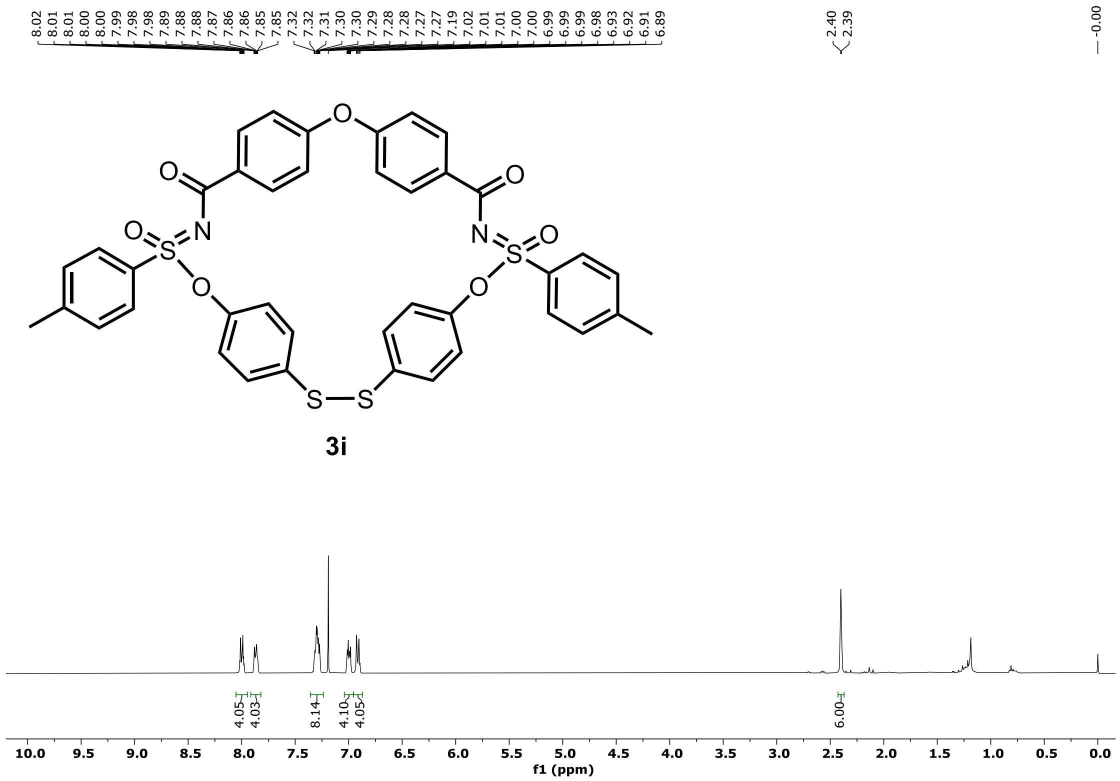

Figure S37. ^{1}H NMR (400 MHz) spectra of compound **3i** ($CDCl_3$, 298 K).

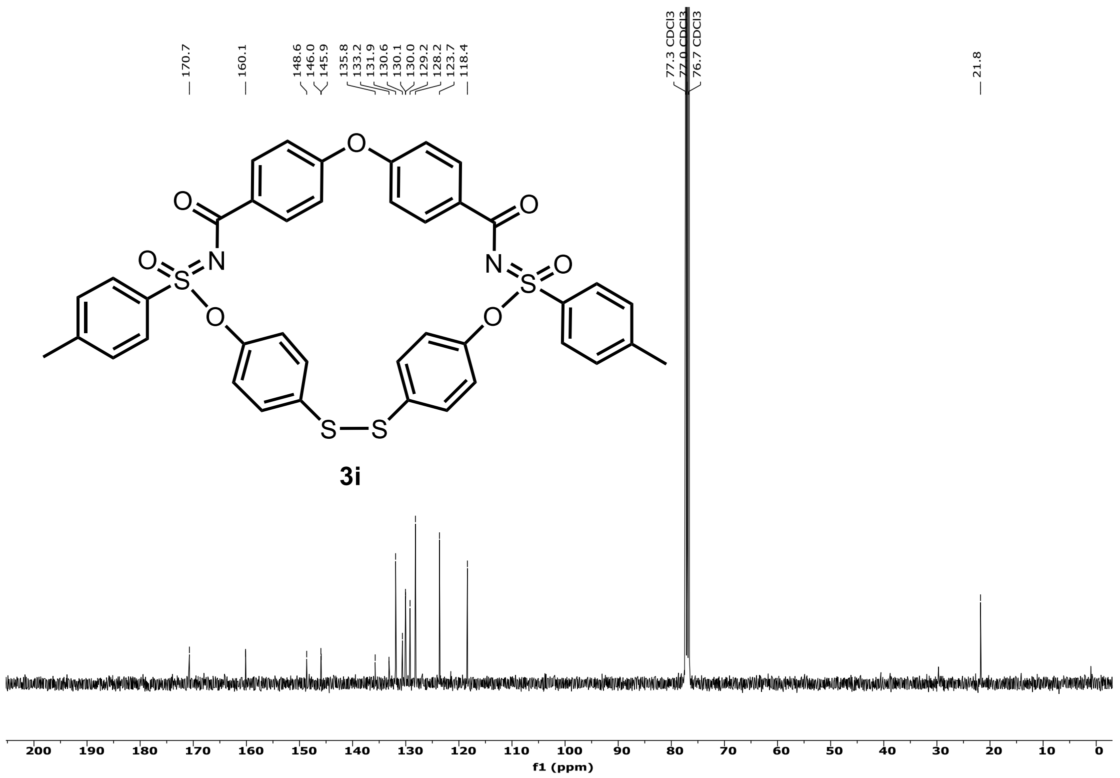

Figure S38. $^{13}C\{1H\}$ NMR (101 MHz) spectra of compound **3i** ($CDCl_3$, 298 K).

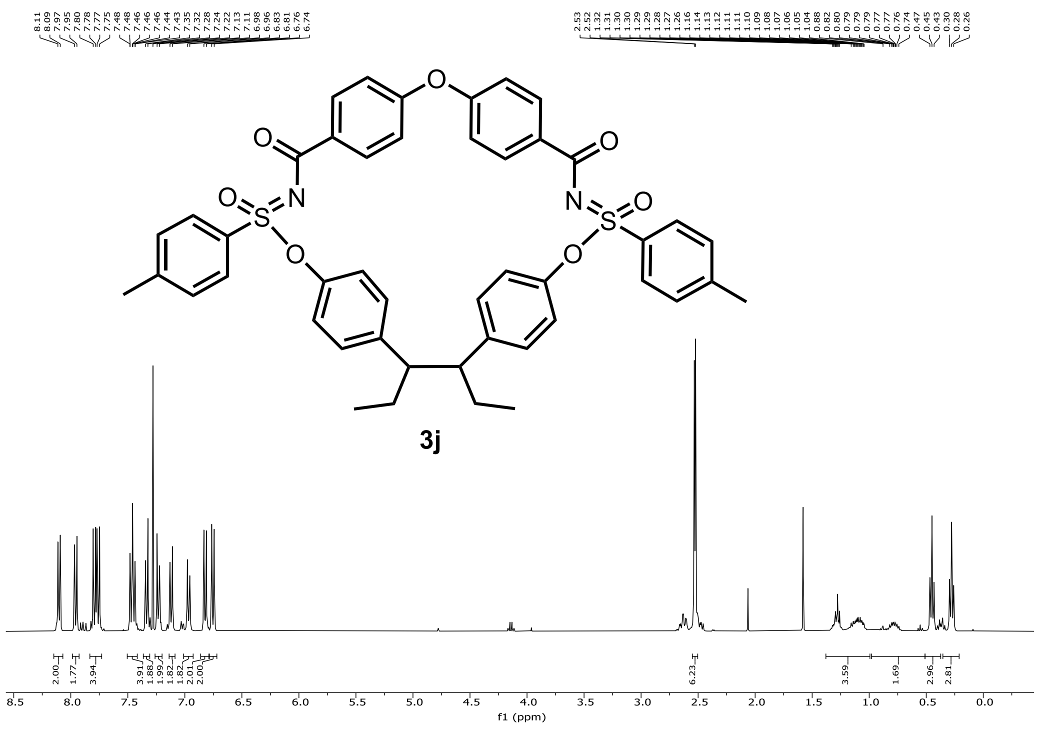

Figure S39. ^{1}H NMR (400 MHz) spectra of compound **3j** ($CDCl_3$, 298 K).

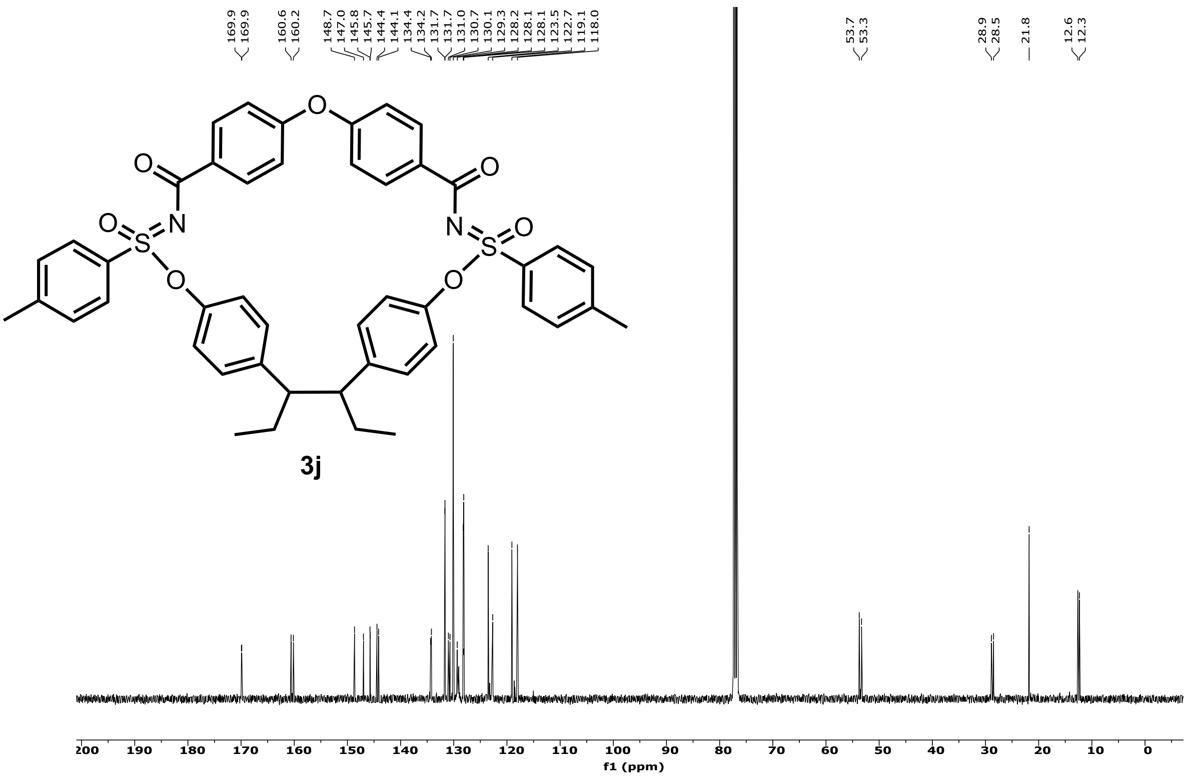

Figure S40. $^{13}C\{1H\}$ NMR (101 MHz) spectra of compound **3j** ($CDCl_3$, 298 K).

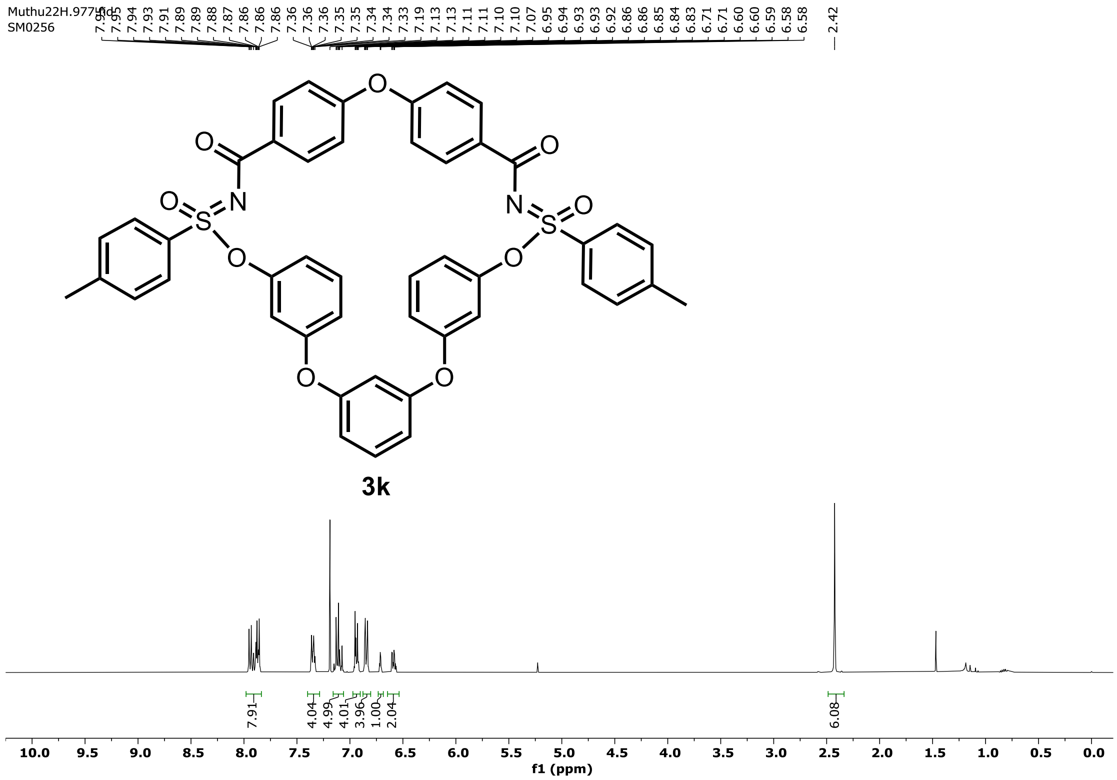

Figure S41. ^{1}H NMR (400 MHz) spectra of compound **3k** ($CDCl_3$, 298 K).

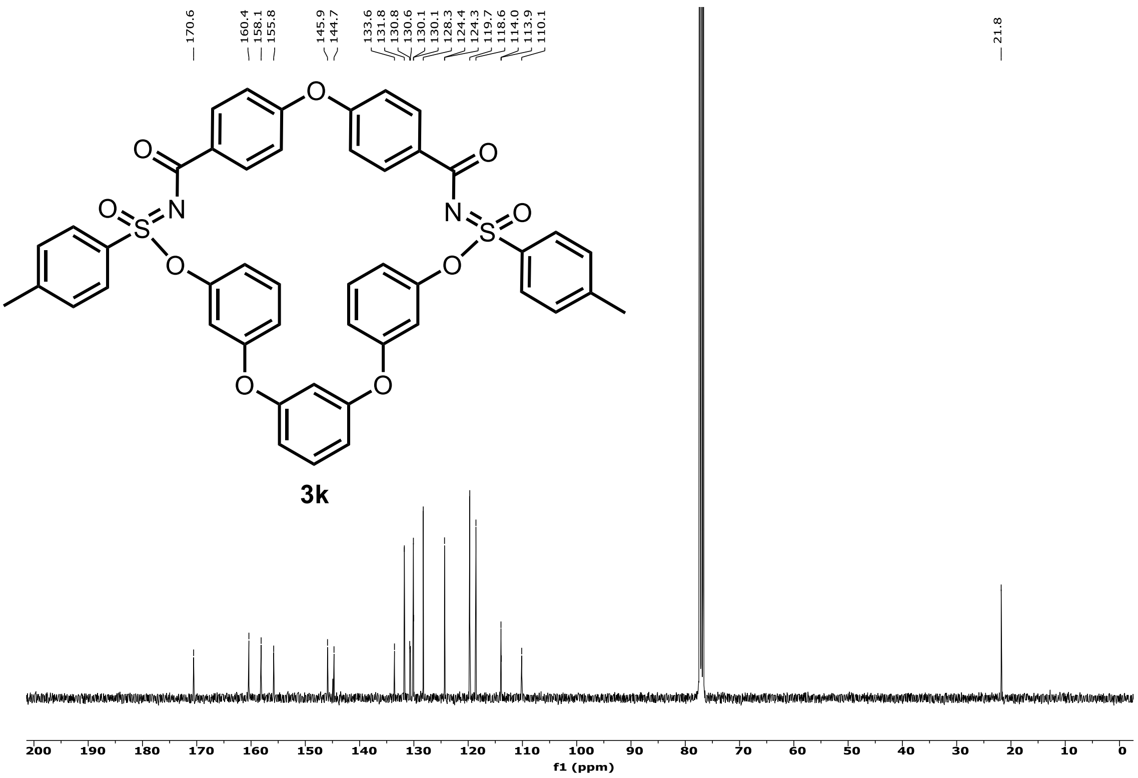

Figure S42. $^{13}C\{1H\}$ NMR (101 MHz) spectra of compound **3k** ($CDCl_3$, 298 K).

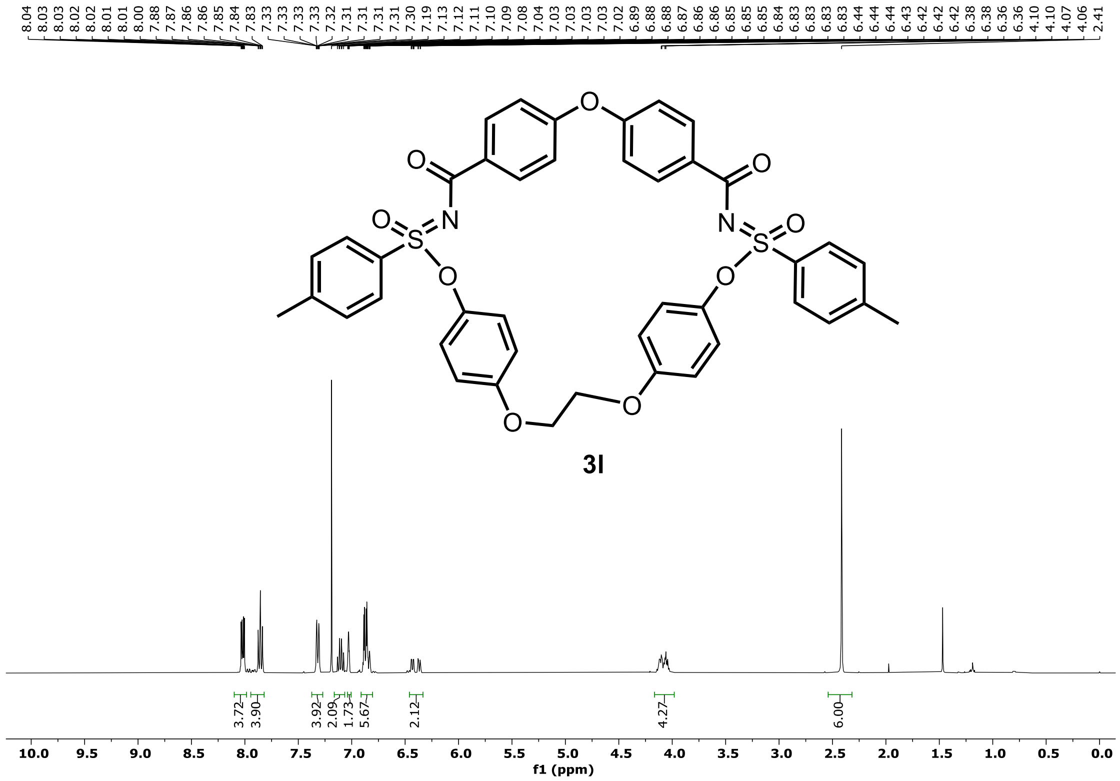

Figure S43. ^{1}H NMR (400 MHz) spectra of compound **3l** ($CDCl_3$, 298 K).

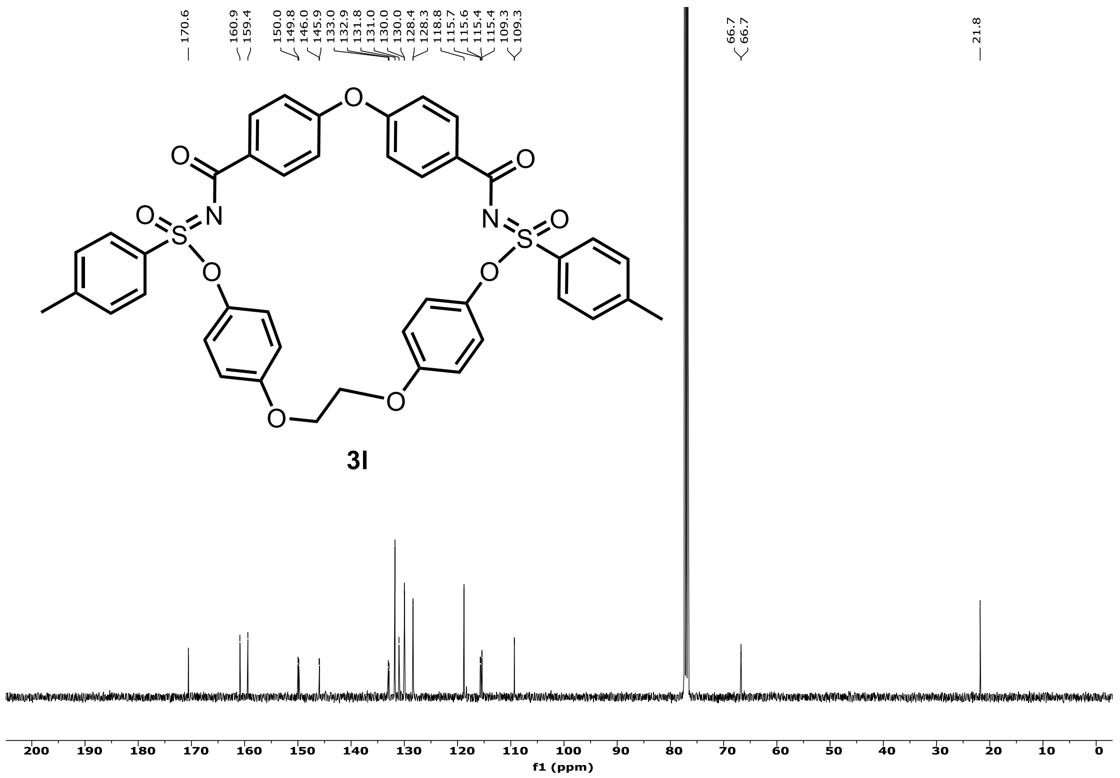

Figure S44. $^{13}C\{1H\}$ NMR (101 MHz) spectra of compound **3l** ($CDCl_3$, 298 K).

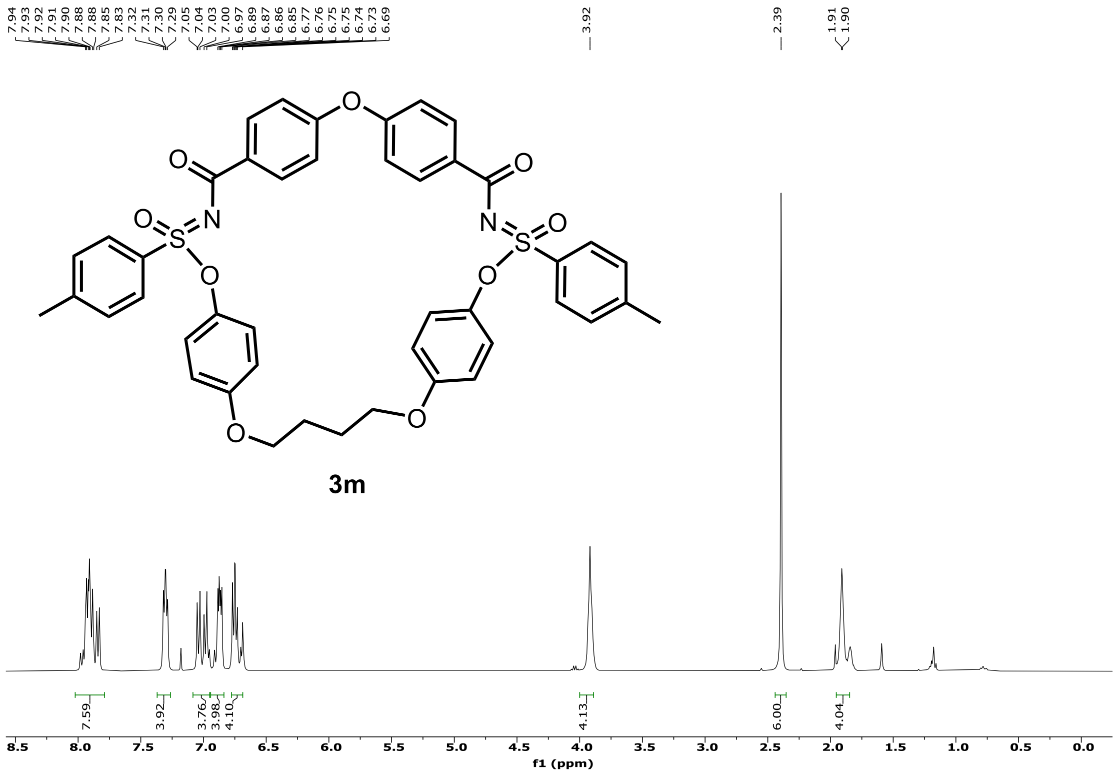

Figure S45. ^{1}H NMR (400 MHz) spectra of compound **3m** ($CDCl_3$, 298 K).

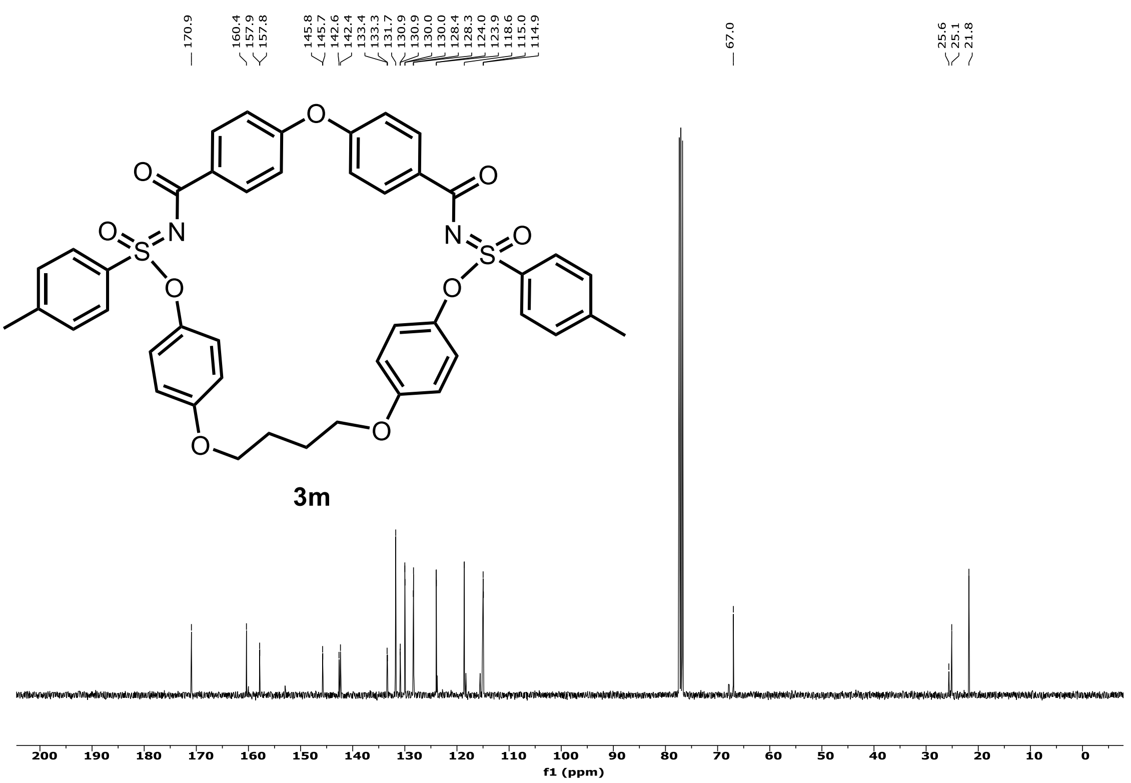

Figure S46. $^{13}C\{1H\}$ NMR (101 MHz) spectra of compound **3m** ($CDCl_3$, 298 K).

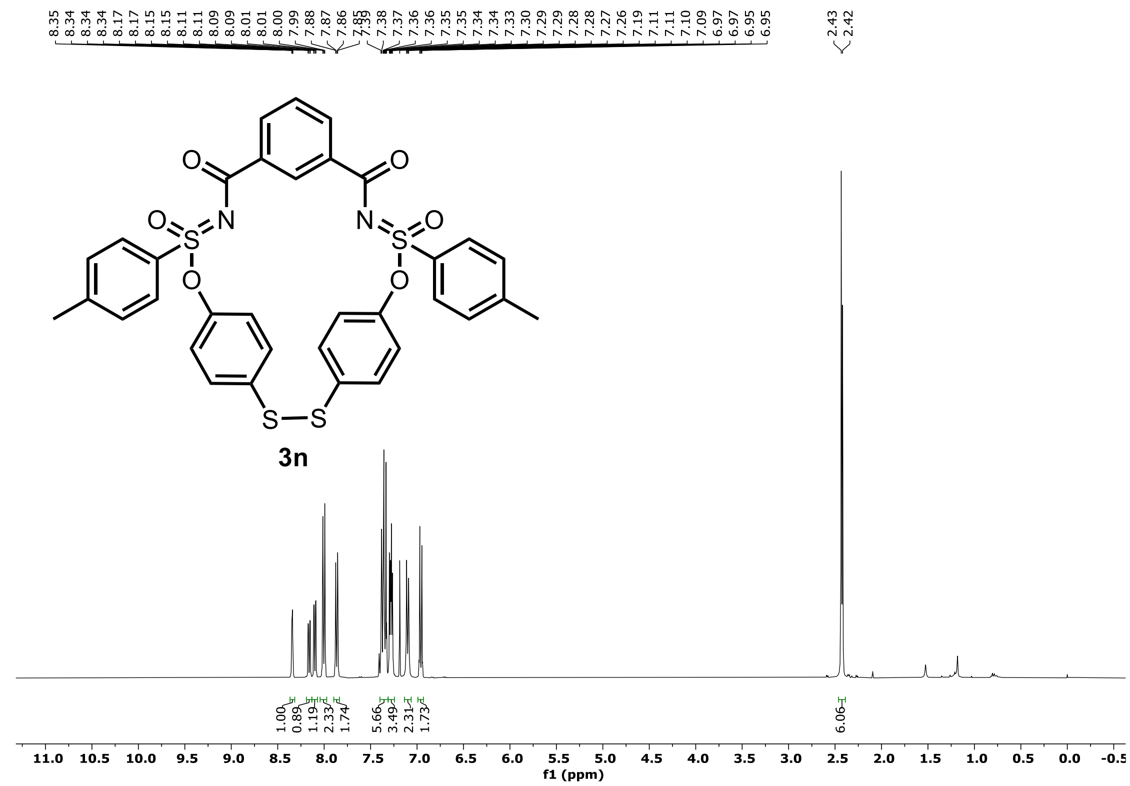

Figure S47. ^{1}H NMR (400 MHz) spectra of compound **3n** ($CDCl_3$, 298 K).

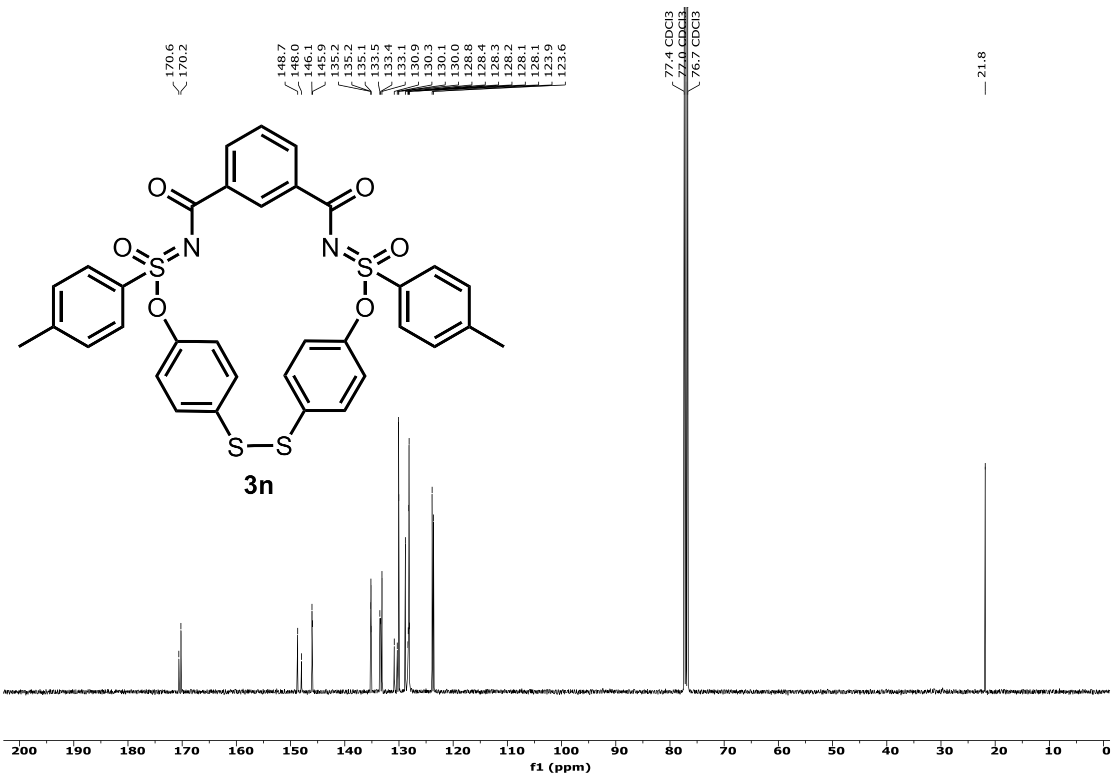

Figure S48. $^{13}C\{1H\}$ NMR (101 MHz) spectra of compound **3n** ($CDCl_3$, 298 K).

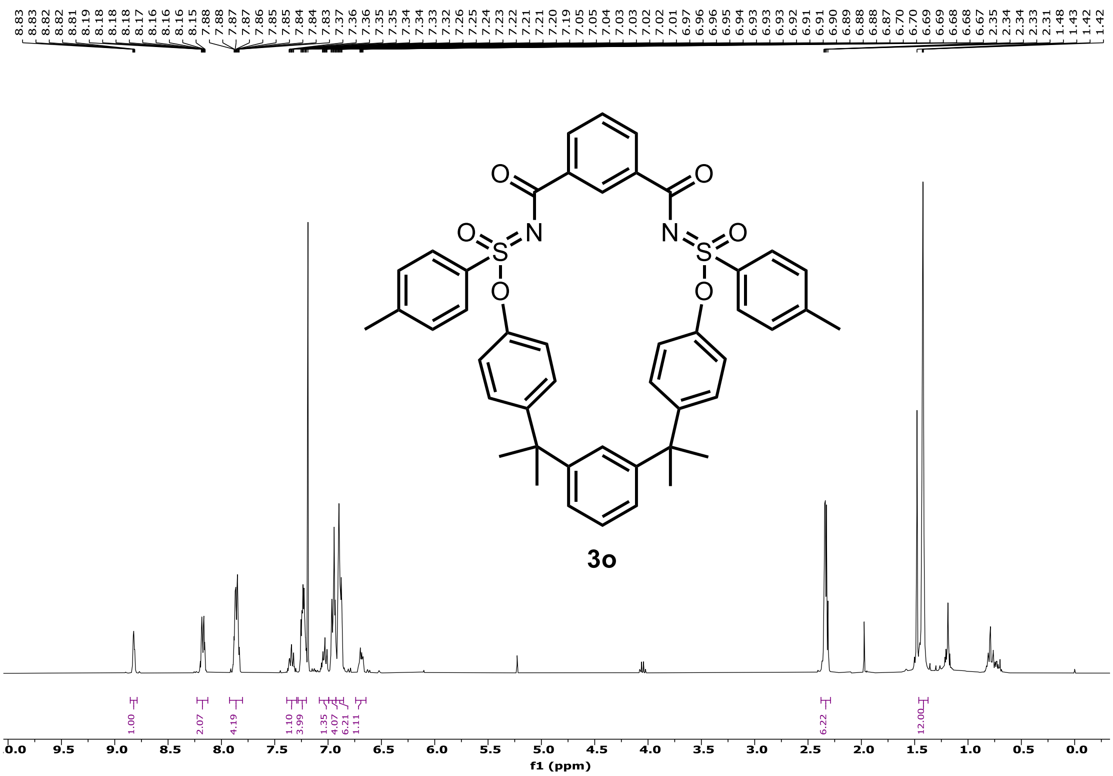

Figure S49. ^{1}H NMR (400 MHz) spectra of compound **3o** ($CDCl_3$, 298 K).

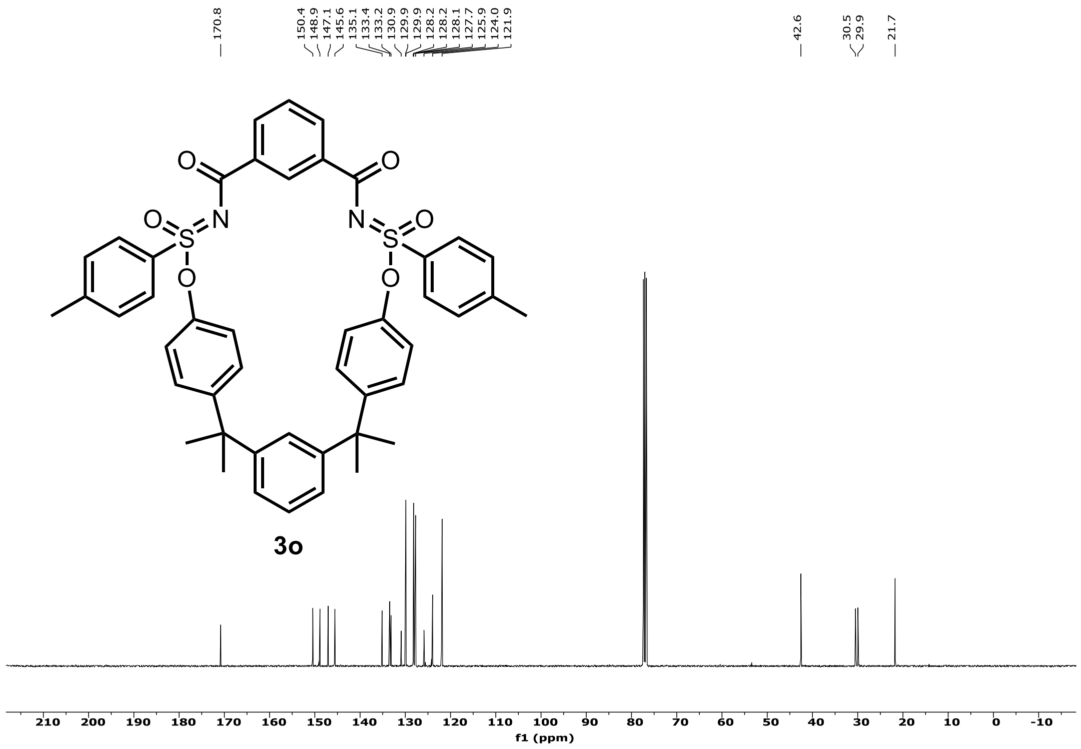

Figure S50. ^{13}C{1H} NMR (101 MHz) spectra of compound **3o** ($CDCl_3$, 298 K).

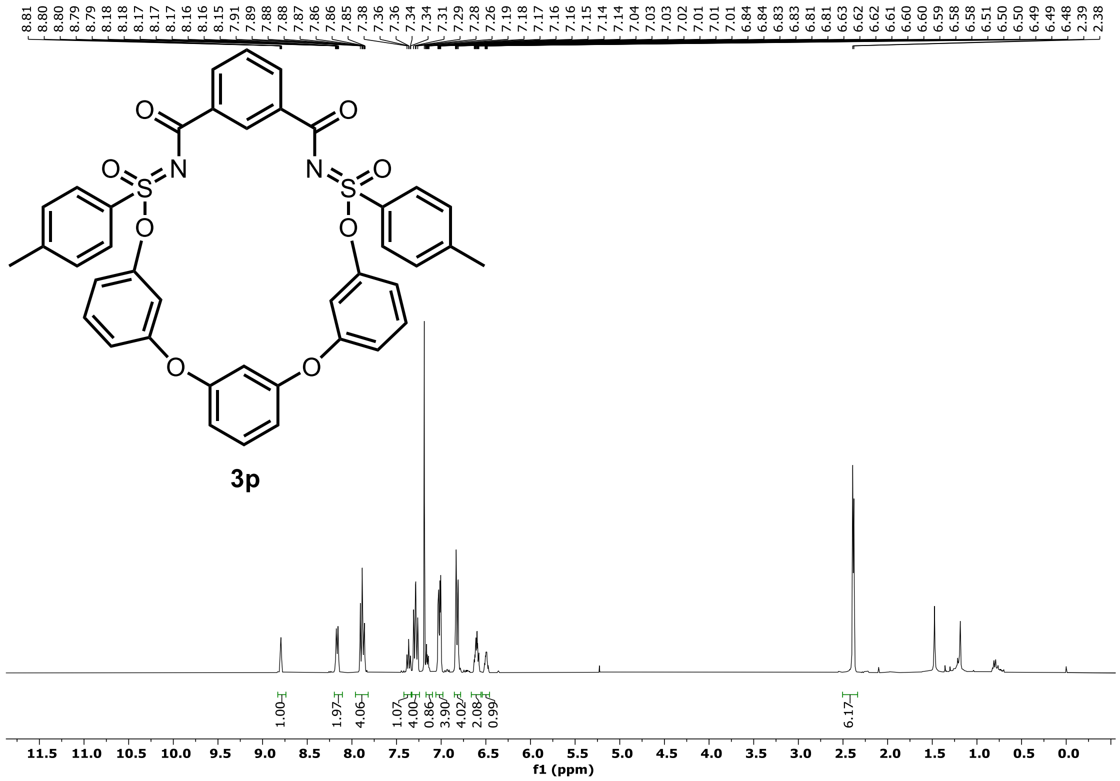

Figure S51. ^{1}H NMR (400 MHz) spectra of compound **3p** ($CDCl_3$, 298 K).

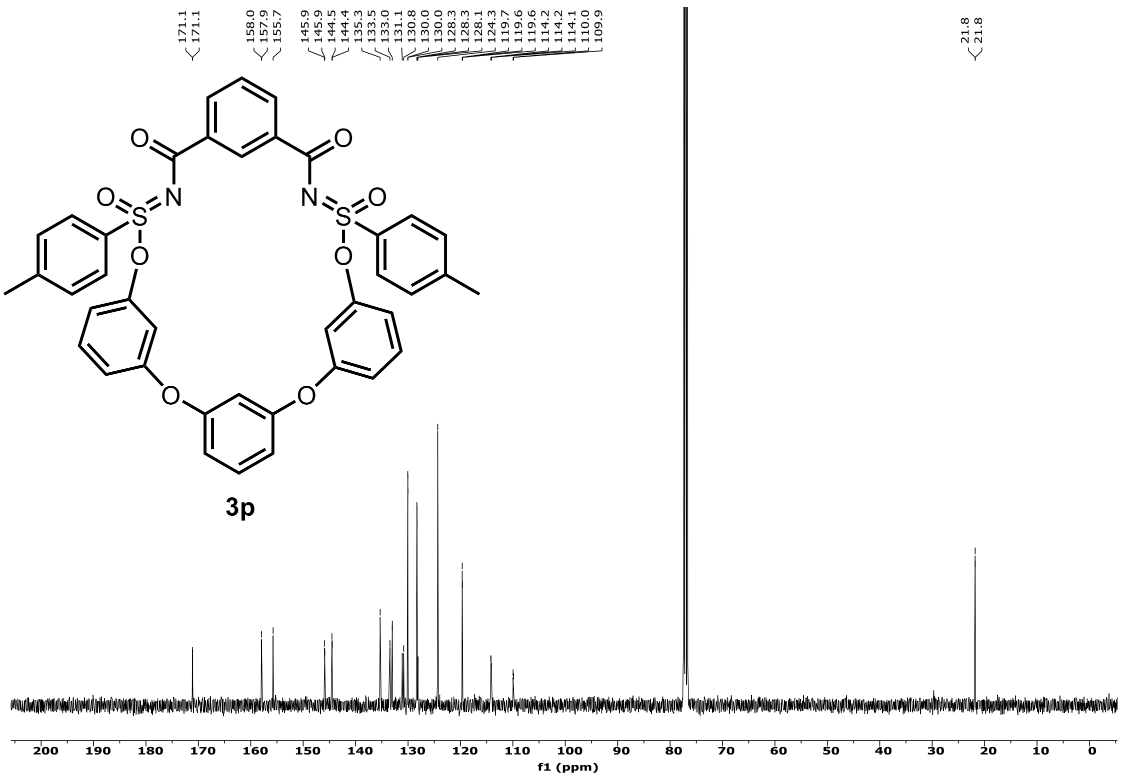

Figure S52. $^{13}C\{1H\}$ NMR (101 MHz) spectra of compound **3p** ($CDCl_3$, 298 K).

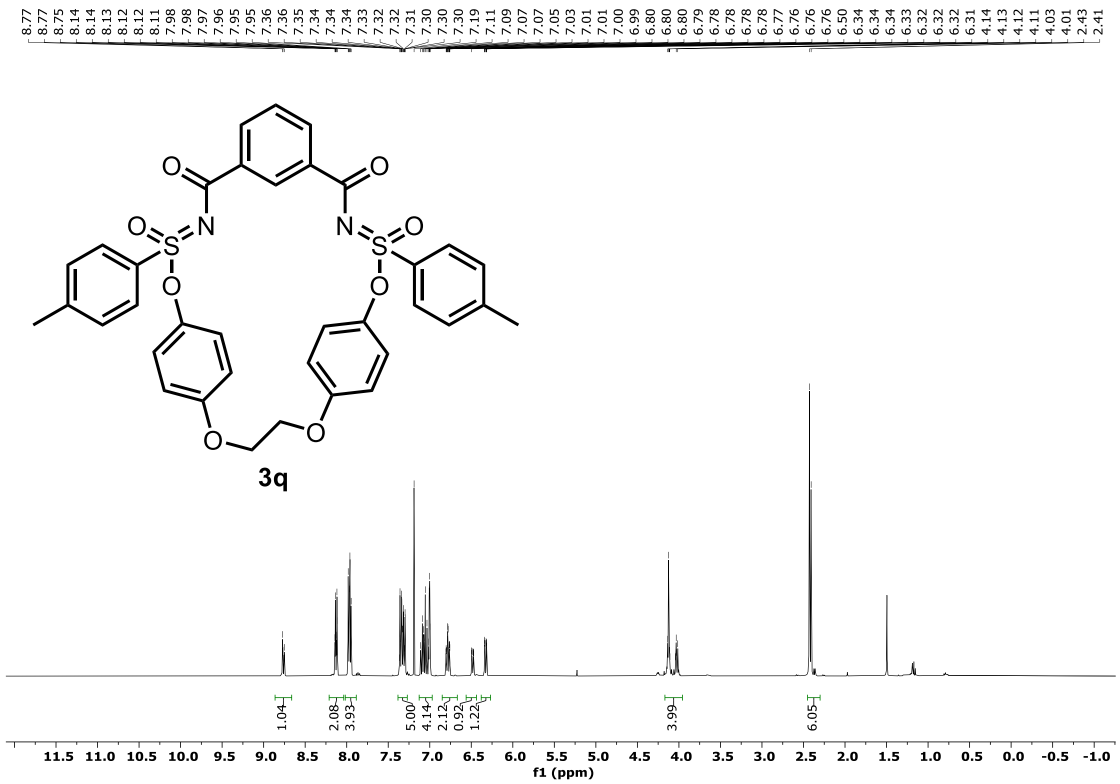

Figure S53. ^{1}H NMR (400 MHz) spectra of compound **3q** ($CDCl_3$, 298 K).

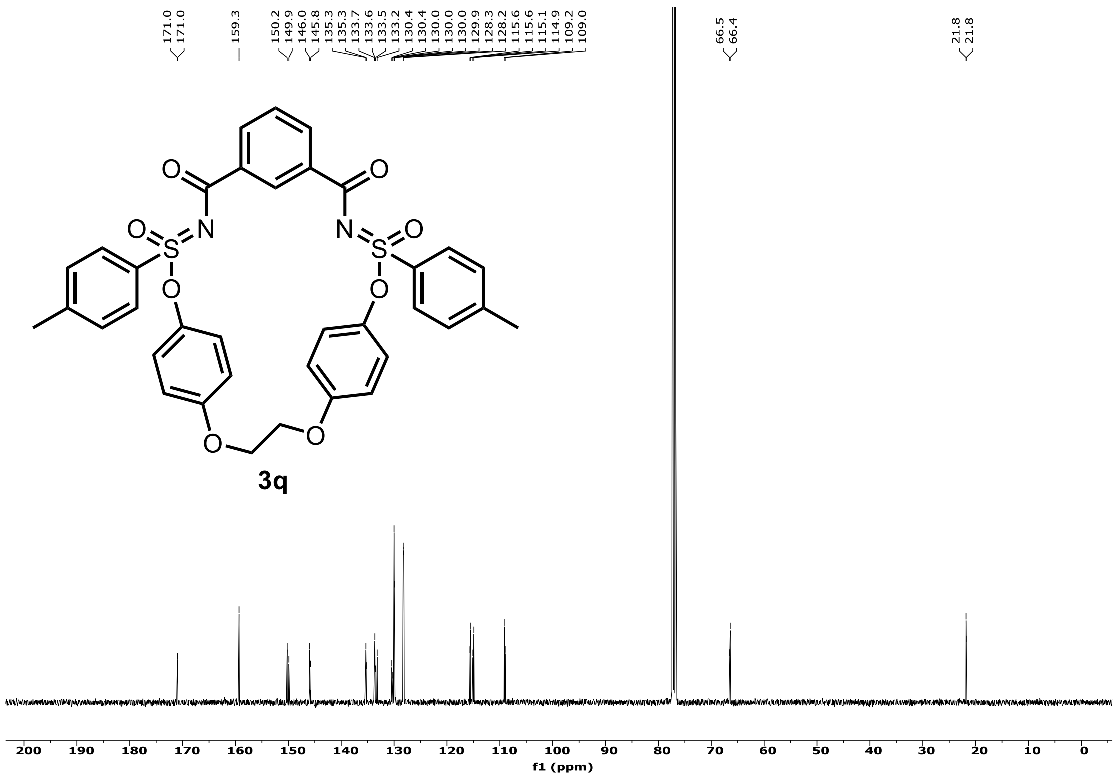

Figure S54. $^{13}C\{1H\}$ NMR (101 MHz) spectra of compound **3q** ($CDCl_3$, 298 K).

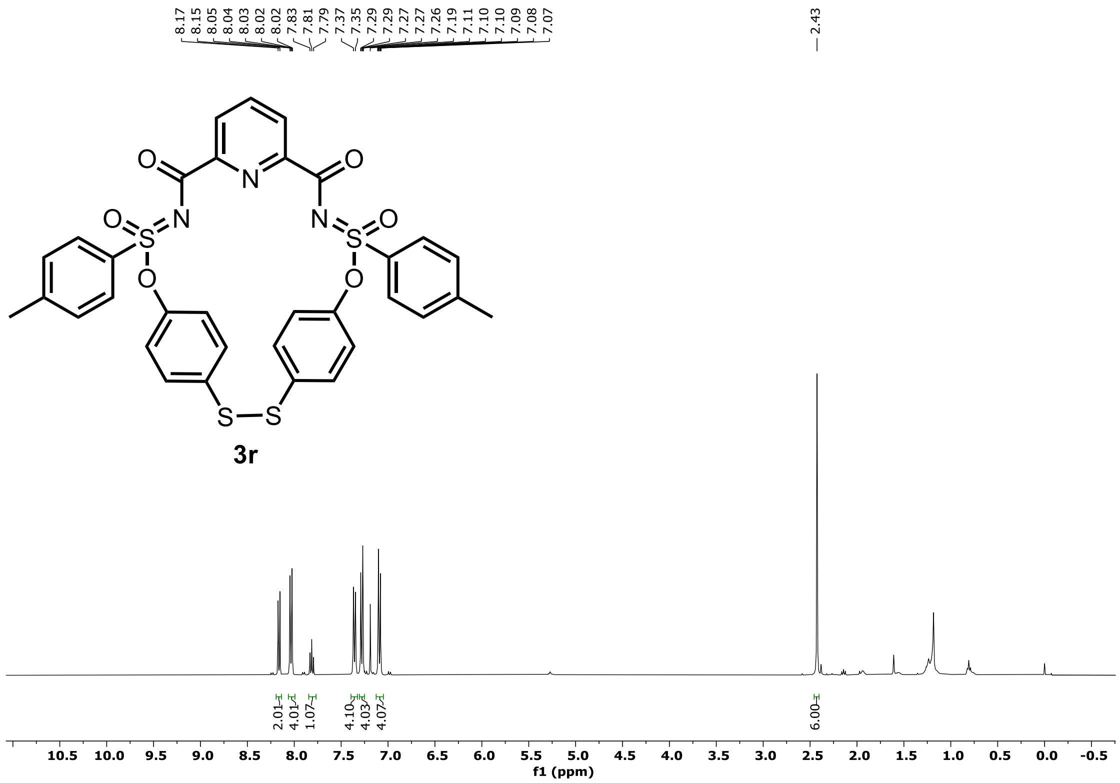

Figure S55. ^{1}H NMR (400 MHz) spectra of compound **3r** ($CDCl_3$, 298 K).

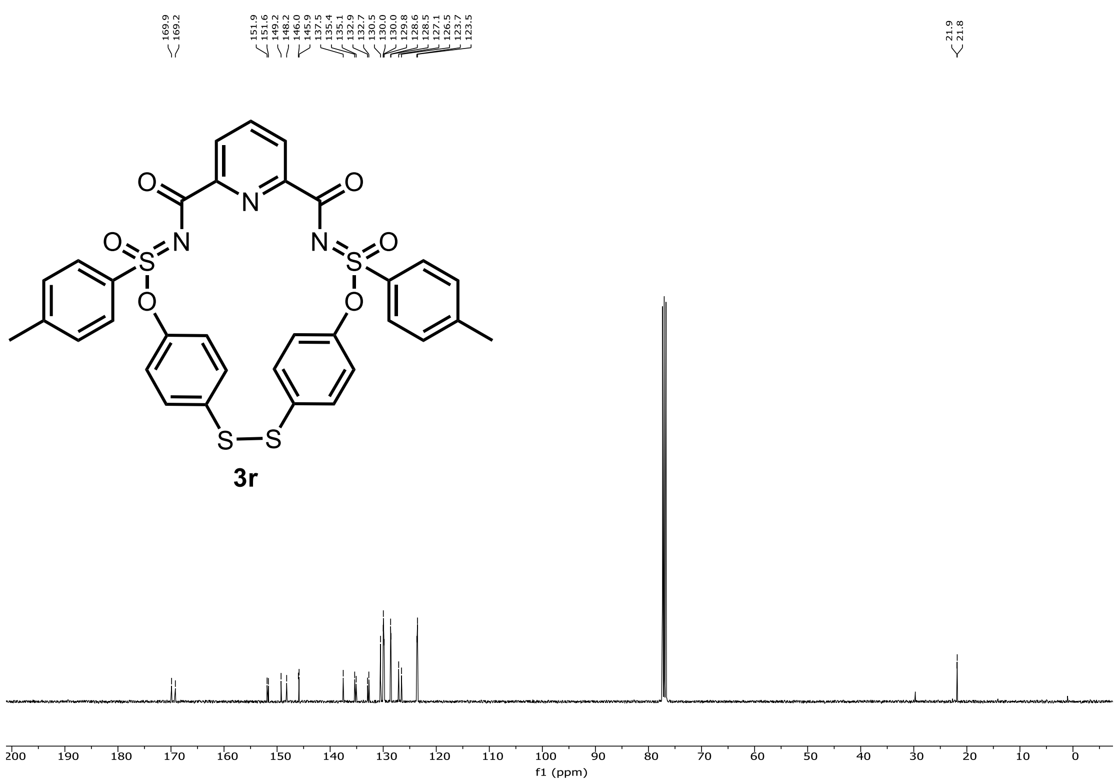

Figure S56. $^{13}C\{1H\}$ NMR (101 MHz) spectra of compound **3r** ($CDCl_3$, 298 K).

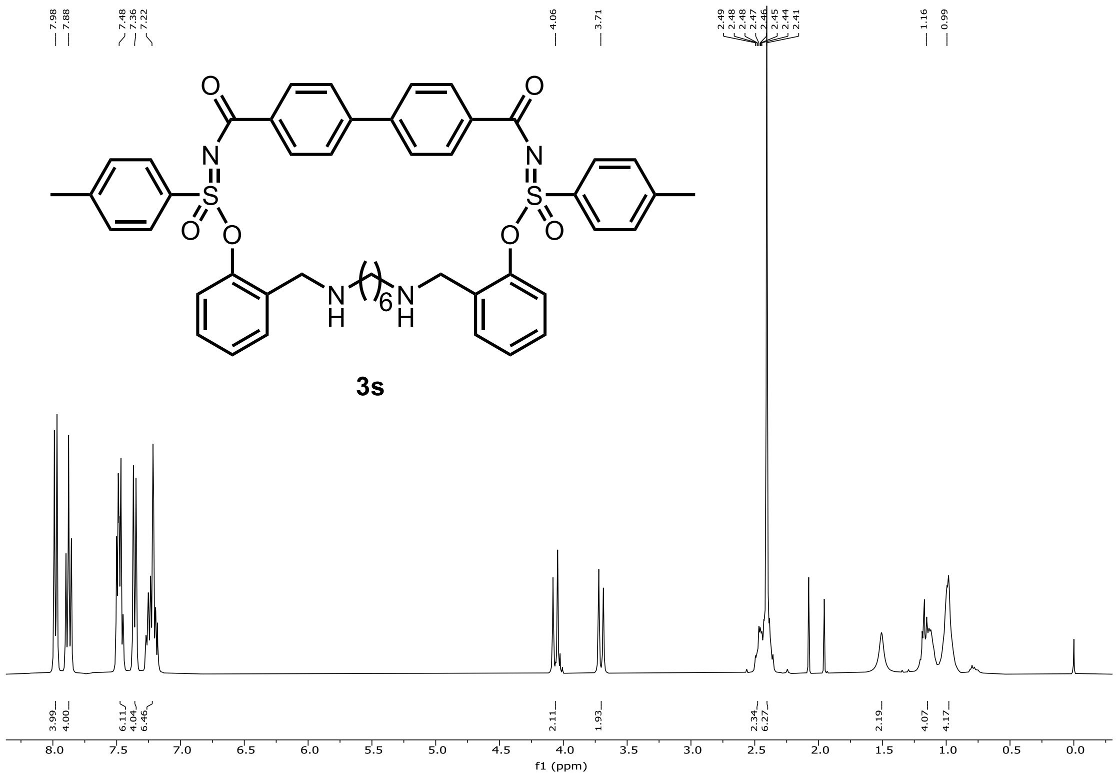

Figure S57. ^{1}H NMR (400 MHz) spectra of compound **3s** ($CDCl_3$, 298 K).

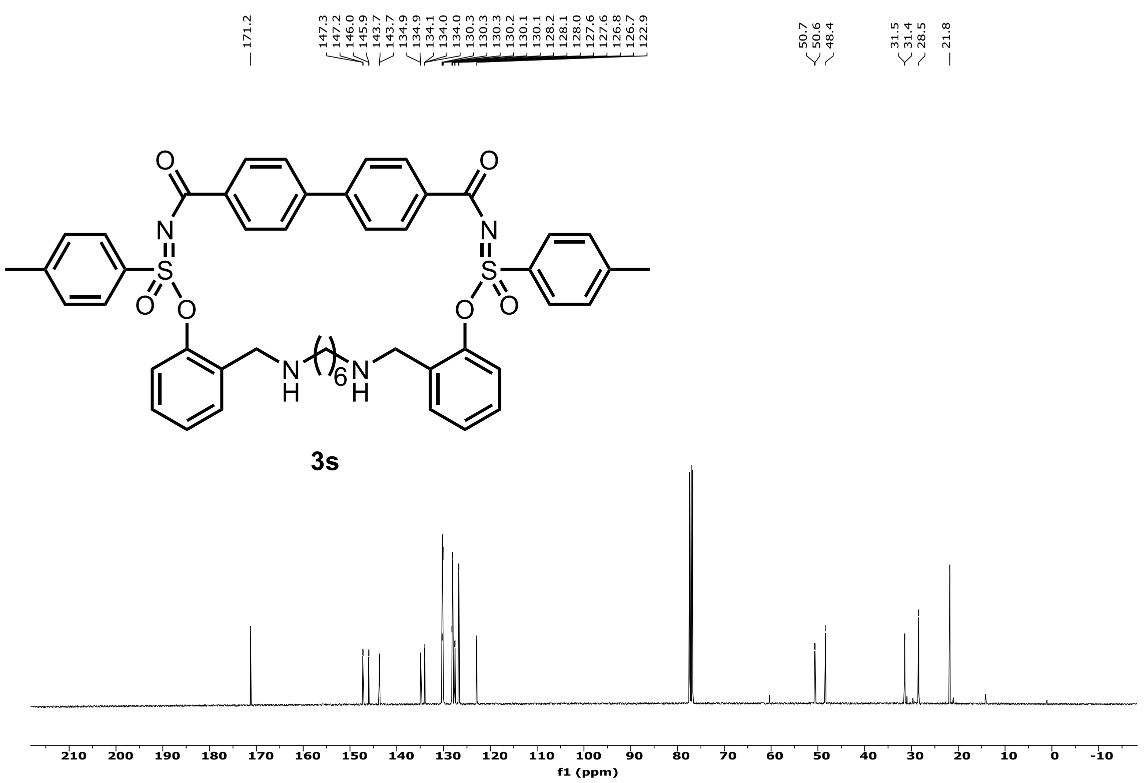

Figure S58. $^{13}C\{1H\}$ NMR (101 MHz) spectra of compound **3s** ($CDCl_3$, 298 K).

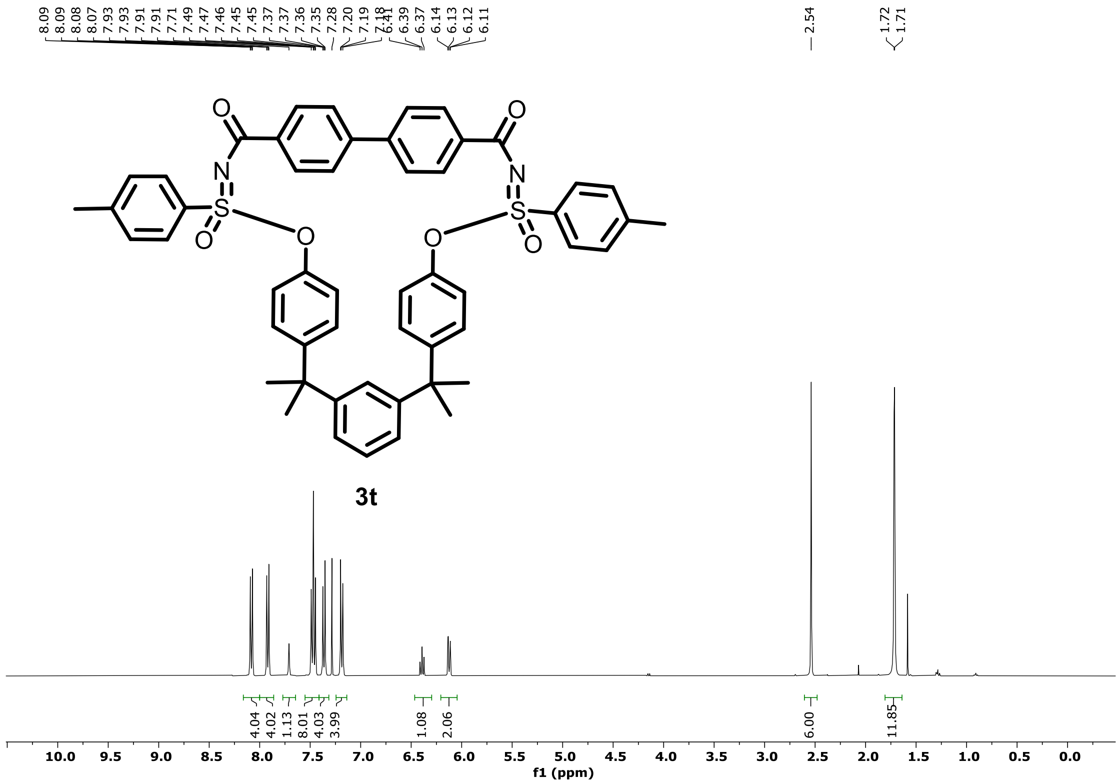

Figure S59. ^{1}H NMR (400 MHz) spectra of compound **3t** ($CDCl_3$, 298 K).

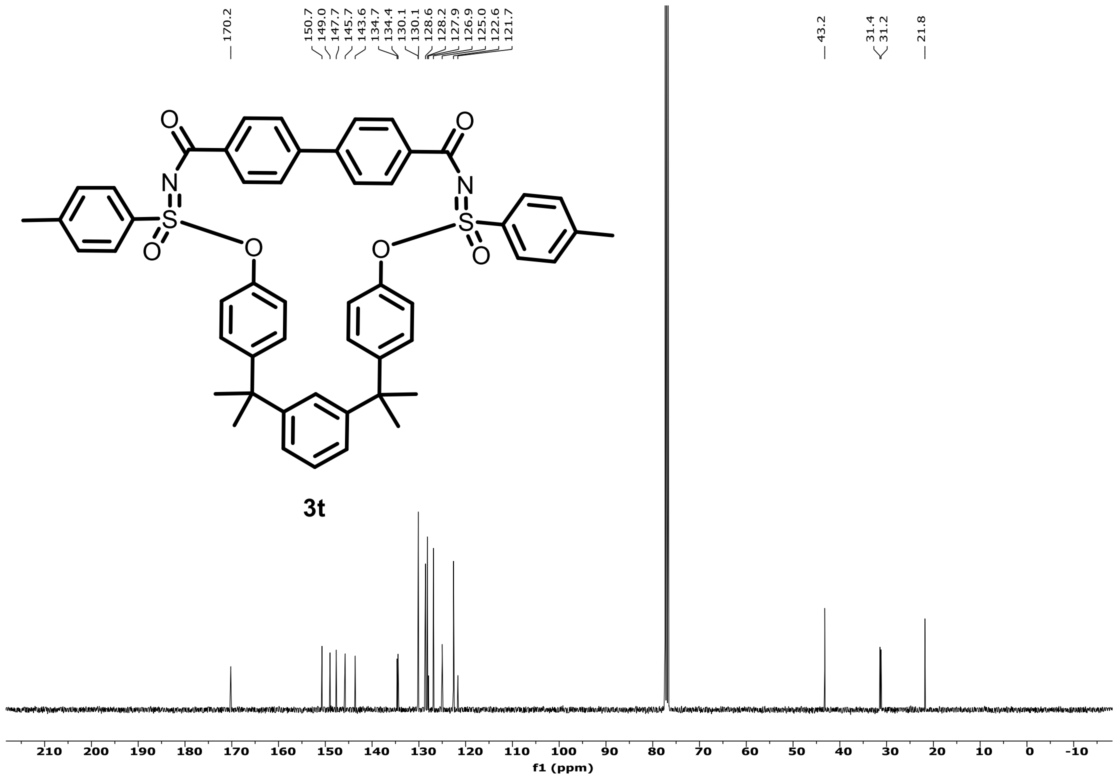

Figure S60. $^{13}C\{1H\}$ NMR (101 MHz) spectra of compound **3t** ($CDCl_3$, 298 K).

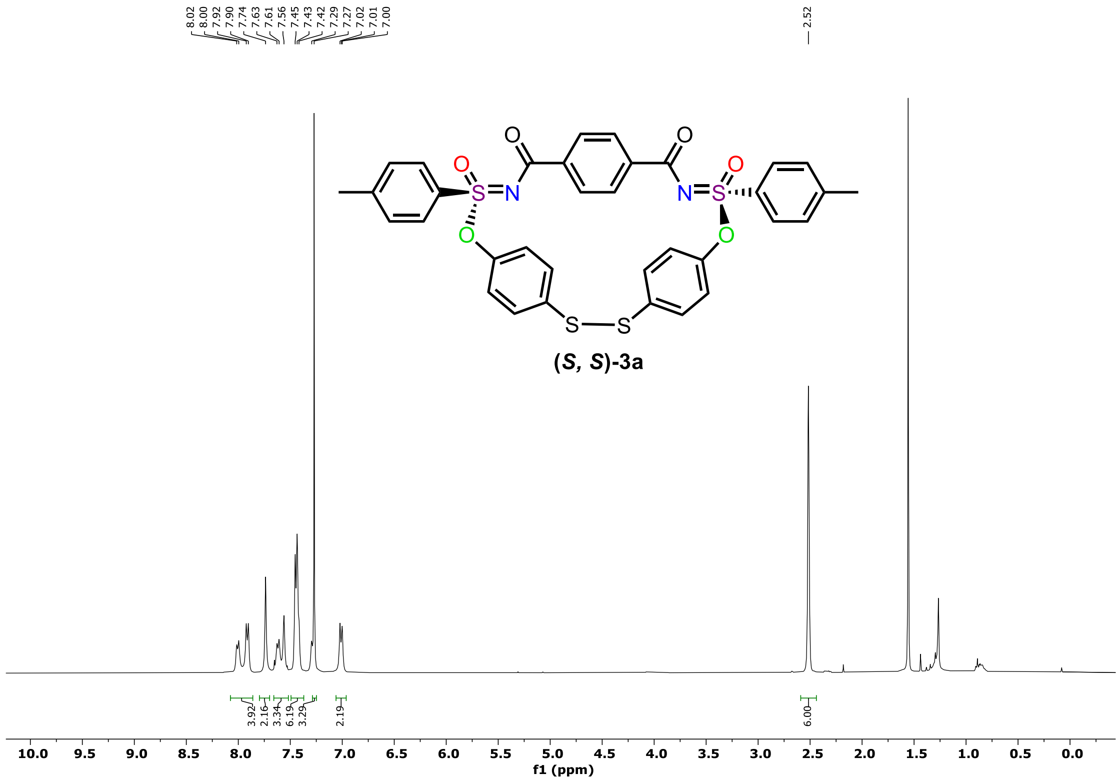

Figure S61. ^{1}H NMR (400 MHz) spectra of compound **(*S, S*)-3a** ($CDCl_3$, 298 K).

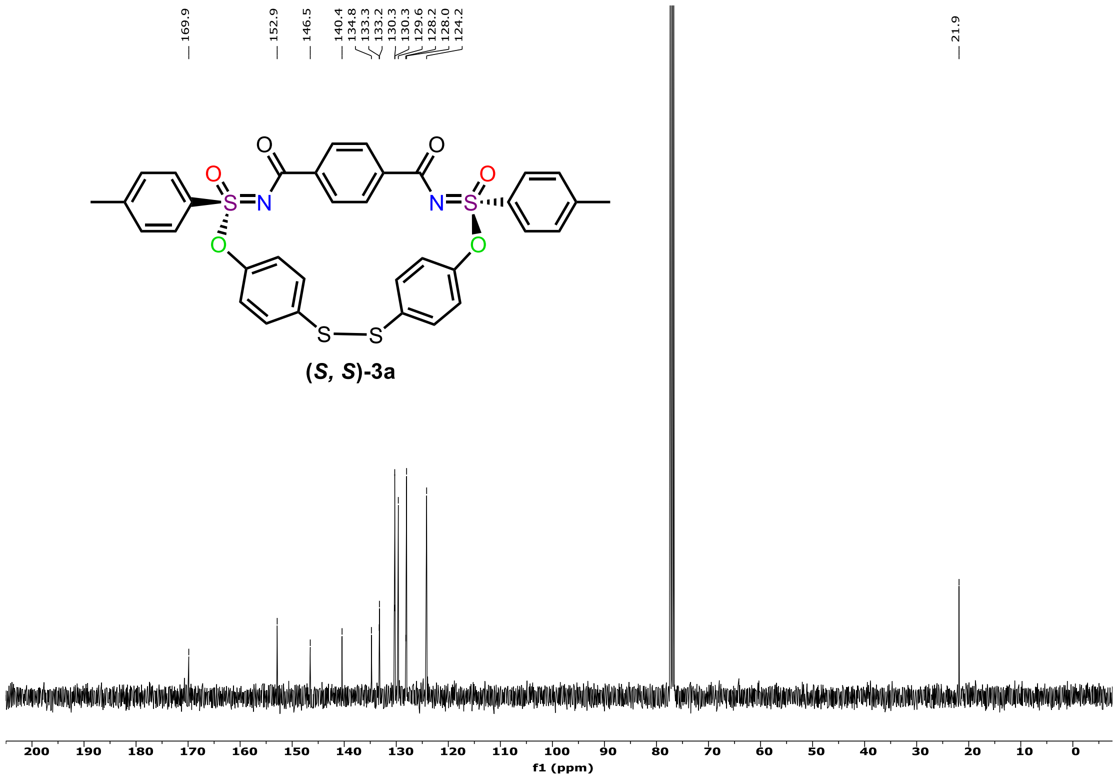

Figure S62. $^{13}C\{1H\}$ NMR (101 MHz) spectra of compound **(*S*, *S*)-3a** ($CDCl_3$, 298 K).

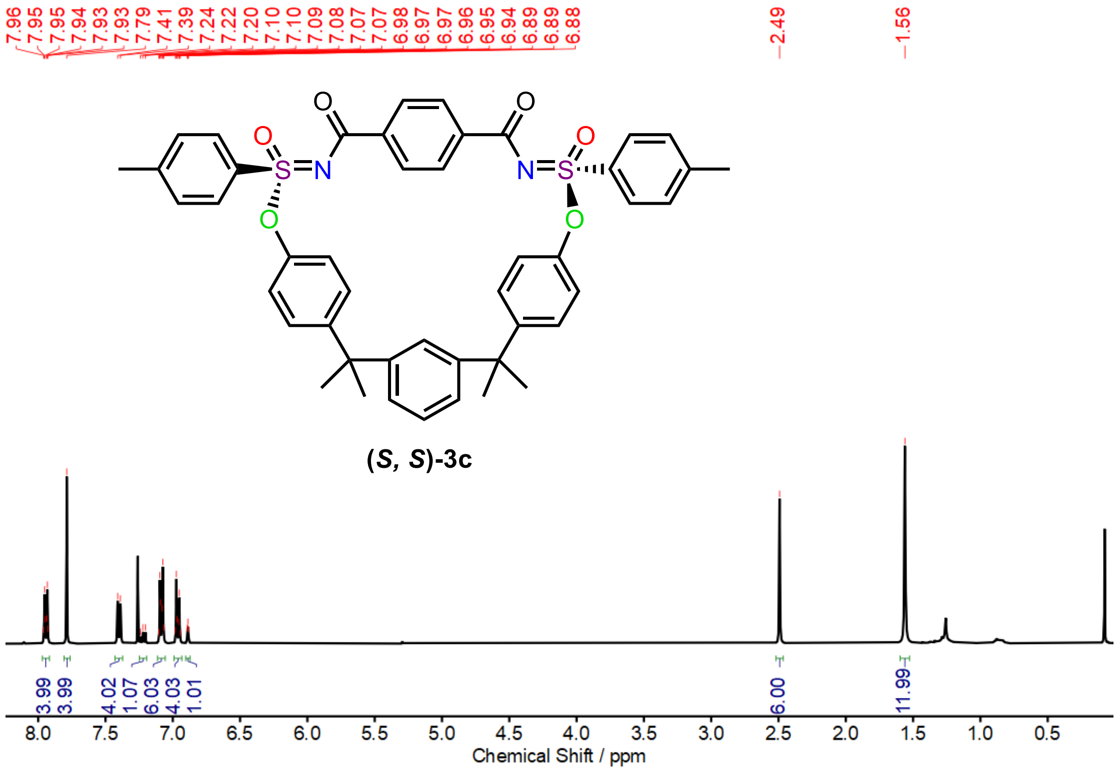

Figure S63. ^{1}H NMR (400 MHz) spectra of compound **(*S*, *S*)-3c** ($CDCl_3$, 298 K).

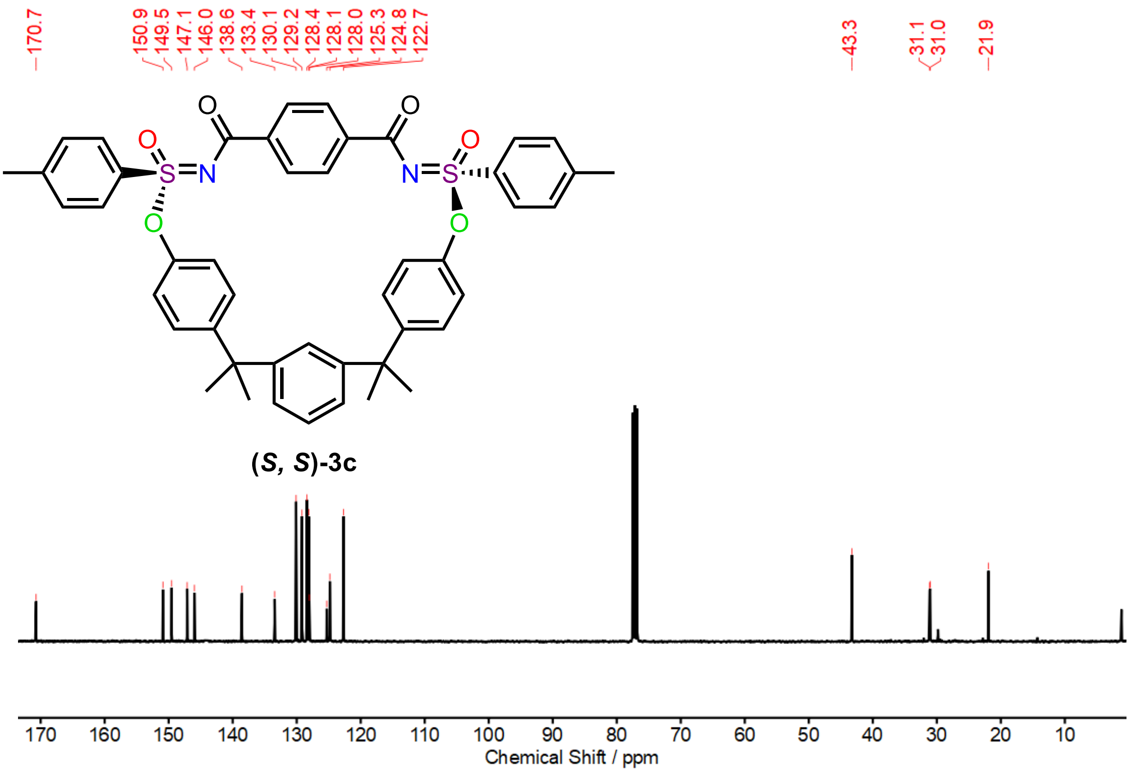

Figure S64. $^{13}C\{1H\}$ NMR (101 MHz) spectra of compound ***(S, S)*-3c** ($CDCl_3$, 298 K).

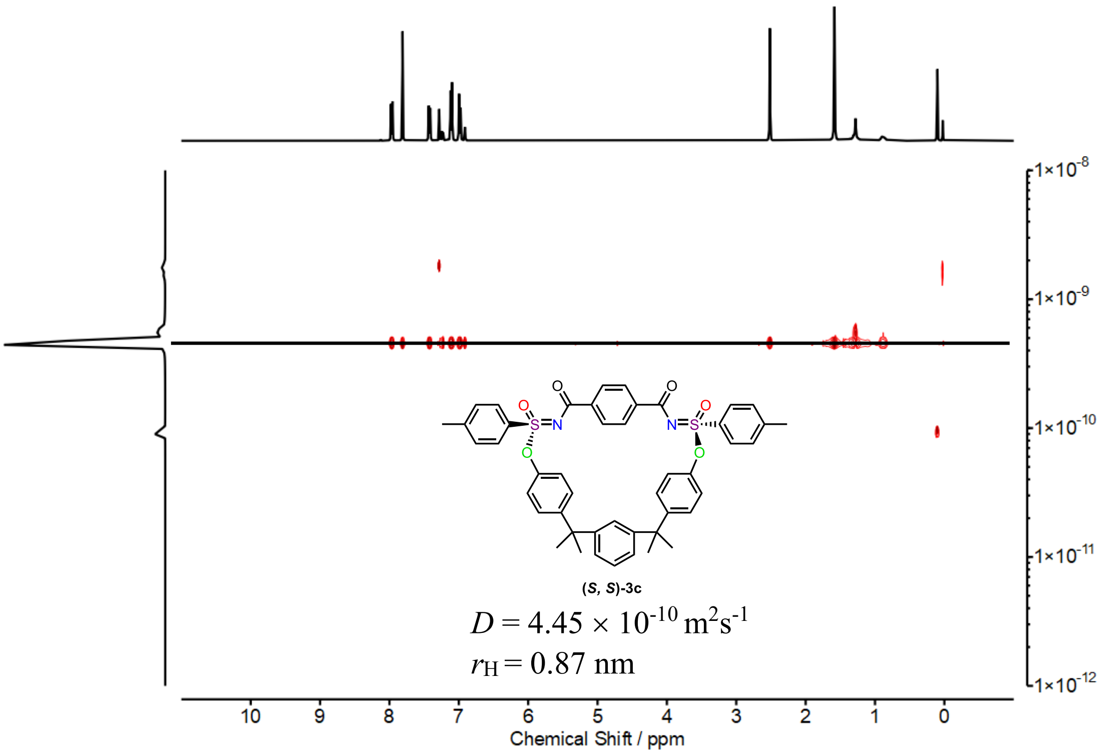

Figure S65. DOSY (400 MHz) spectra of compound ***(S, S)*-3c** ($CDCl_3$, 298 K).

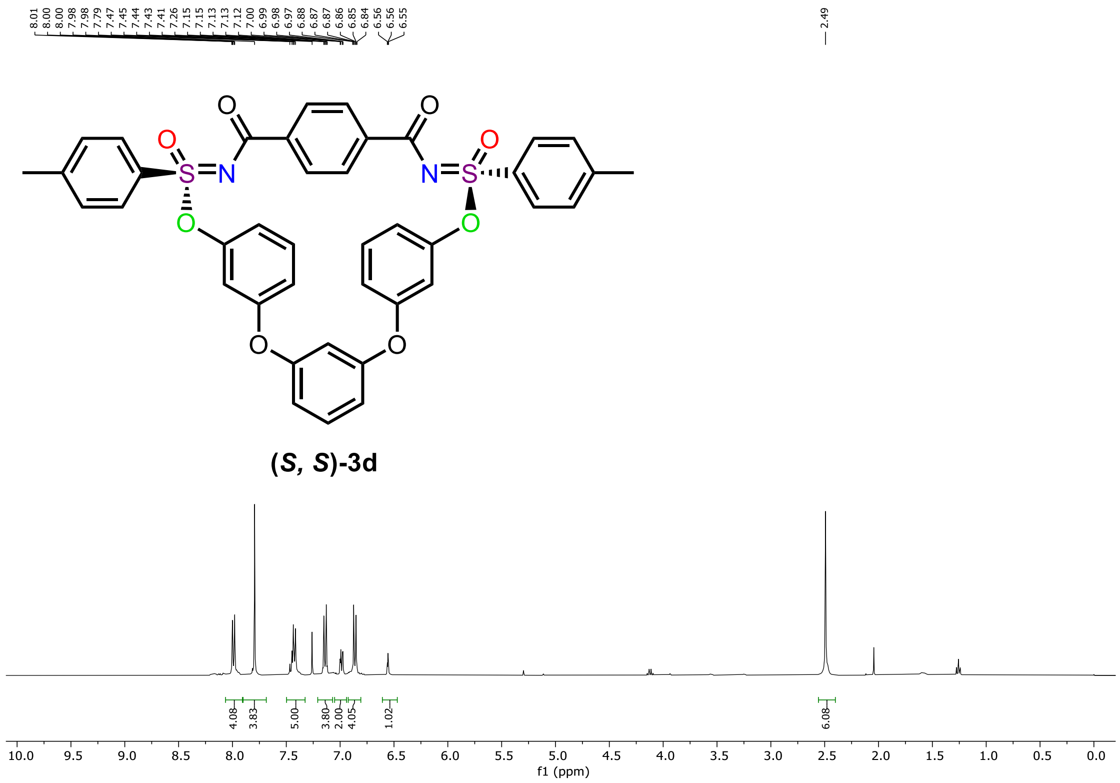

Figure S66. ^{1}H NMR (400 MHz) spectra of compound ***(S, S)*-3d** ($CDCl_3$, 298 K).

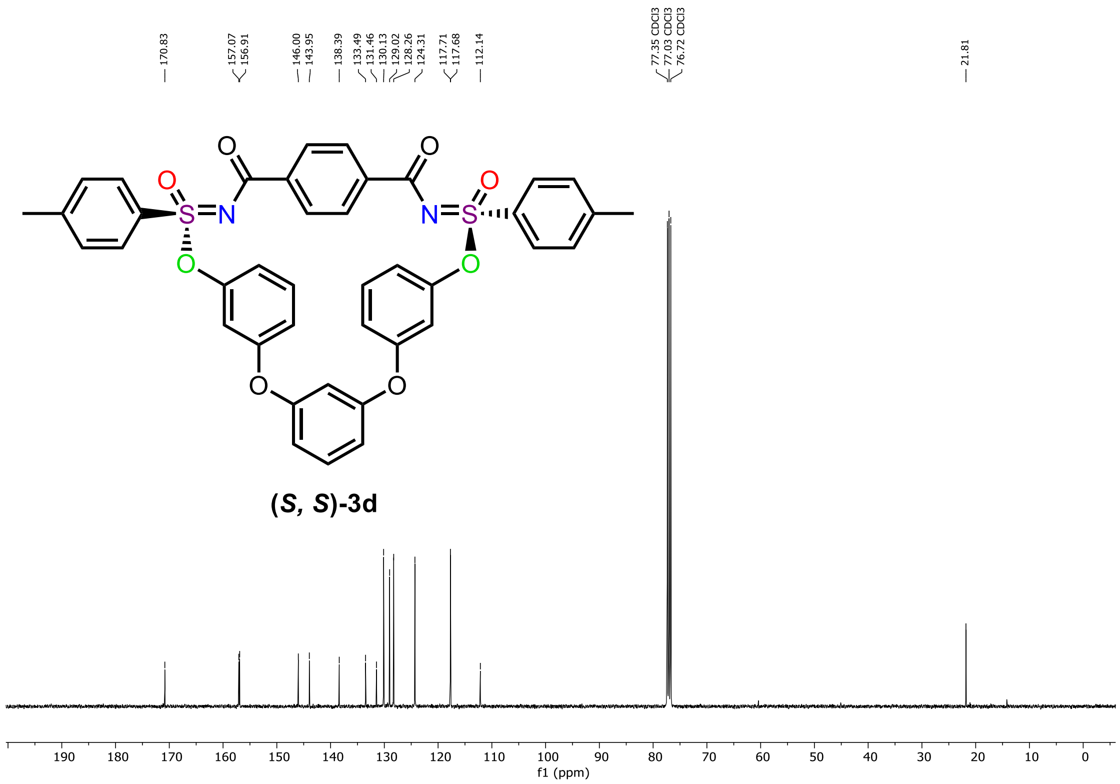

Figure S67. ^{13}C{1H} NMR (101 MHz) spectra of compound ***(S, S)*-3d** ($CDCl_3$, 298 K).

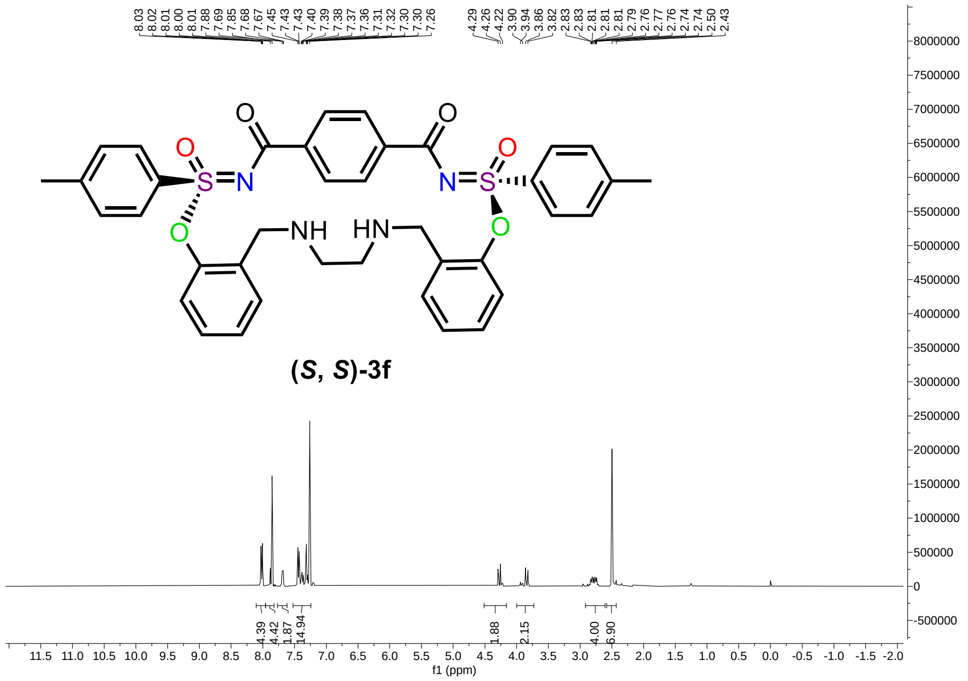

Figure S68. ^{1}H NMR (400 MHz) spectra of compound ***(S, S)*-3f** ($CDCl_3$, 298 K).

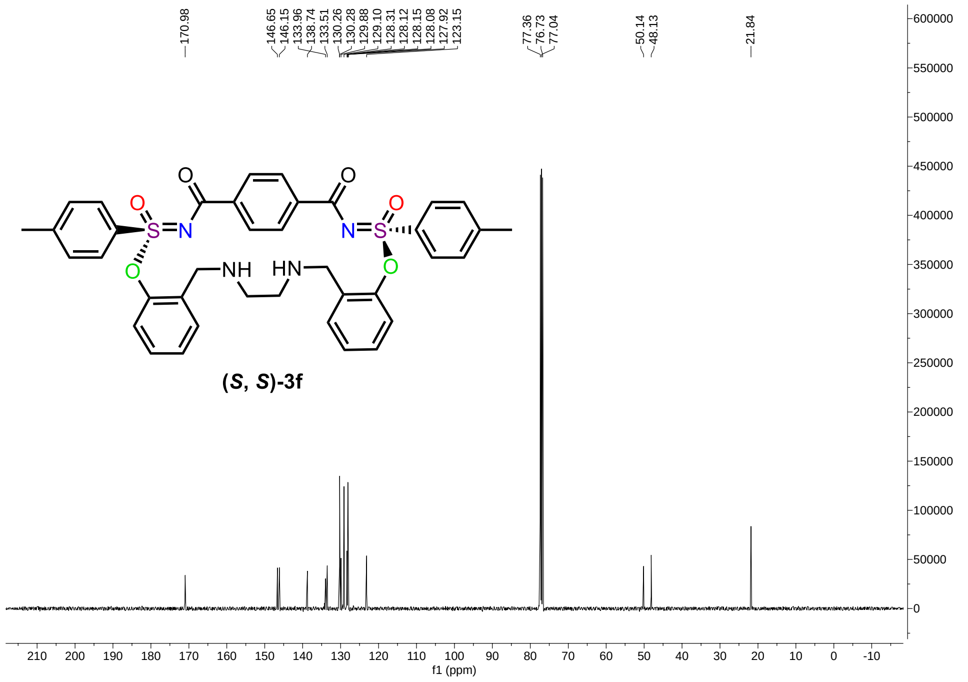

Figure S69. ^{13}C{1H} NMR (101 MHz) spectra of compound ***(S, S)*-3f** ($CDCl_3$, 298 K).

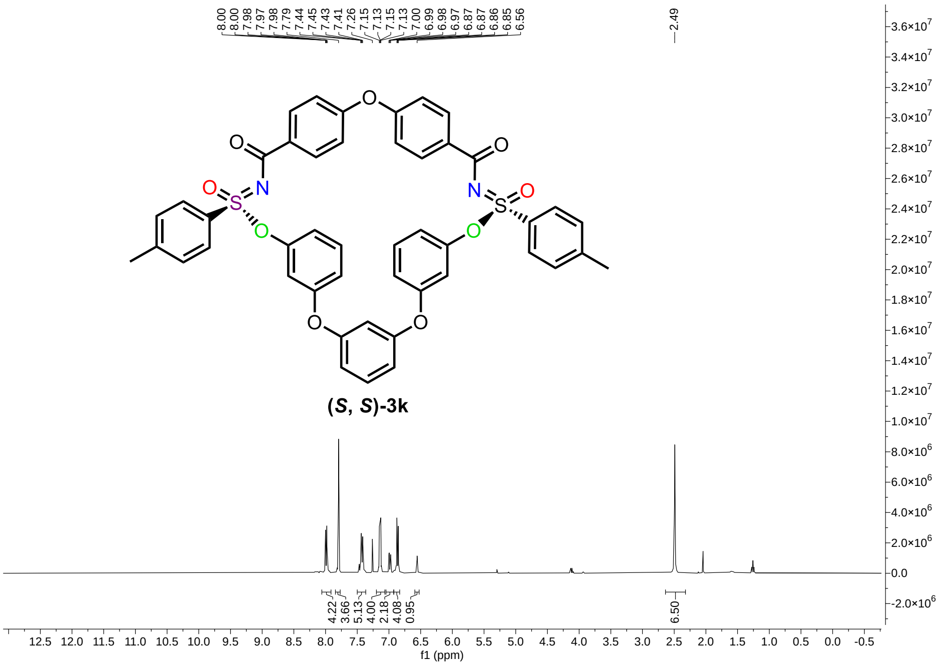

Figure S70. ^{1}H NMR (400 MHz) spectra of compound **(*S*, *S*)-3k** ($CDCl_3$, 298 K).

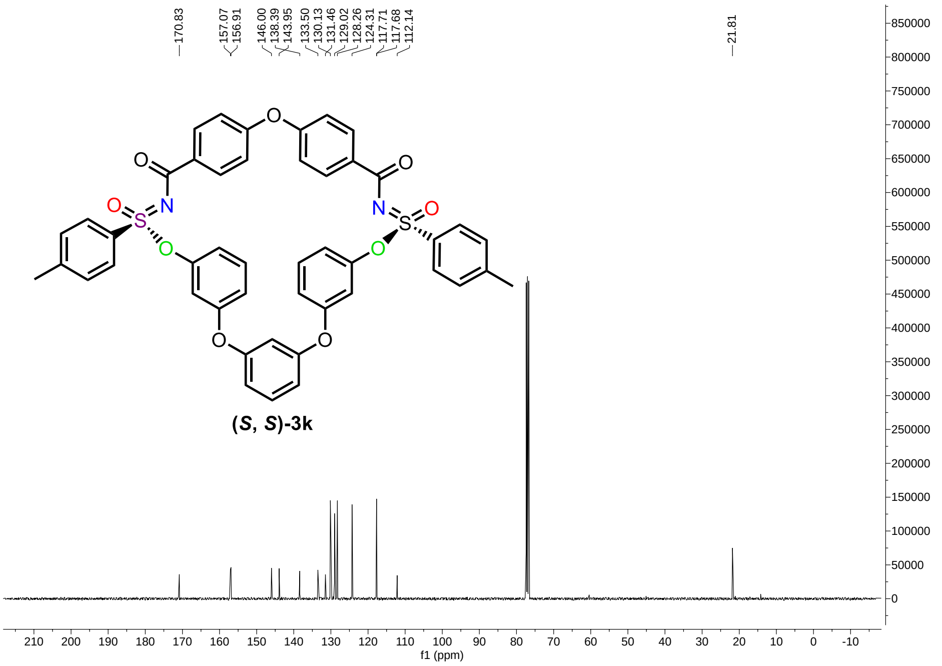

Figure S71. $^{13}C\{1H\}$ NMR (101 MHz) spectra of compound **(*S*, *S*)-3k** ($CDCl_3$, 298 K).

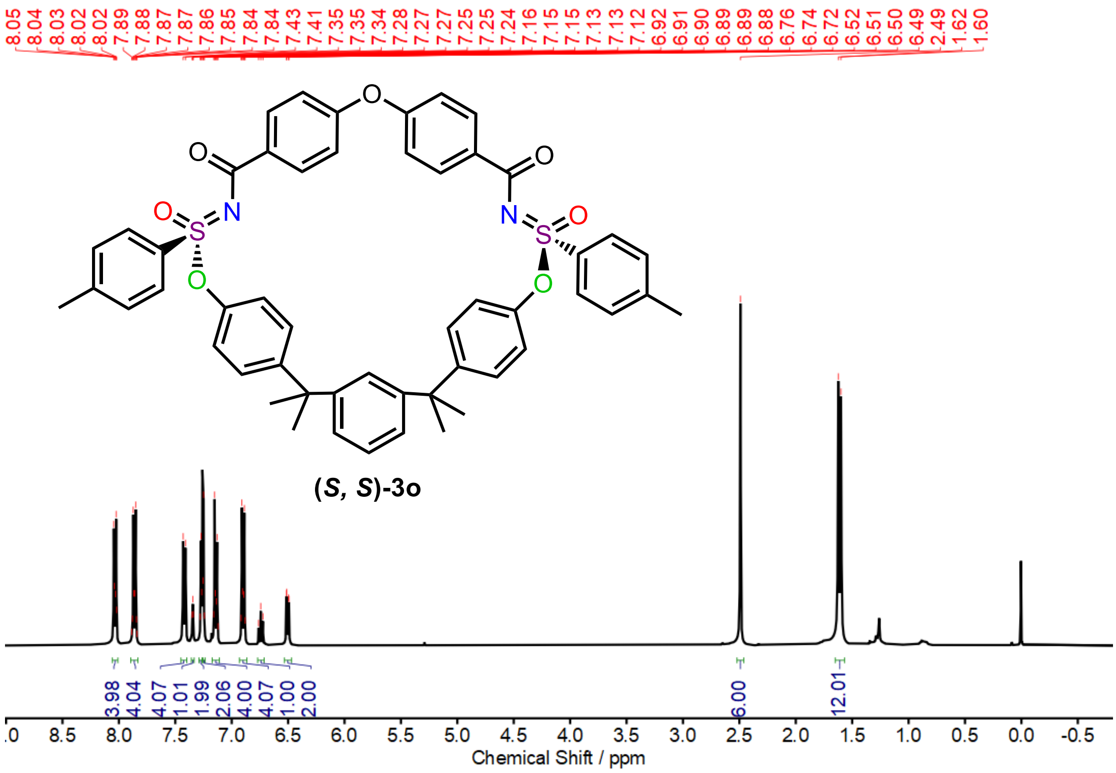

Figure S72. ^{1}H NMR (400 MHz) spectra of compound ***(S, S)*-3o** ($CDCl_3$, 298 K).

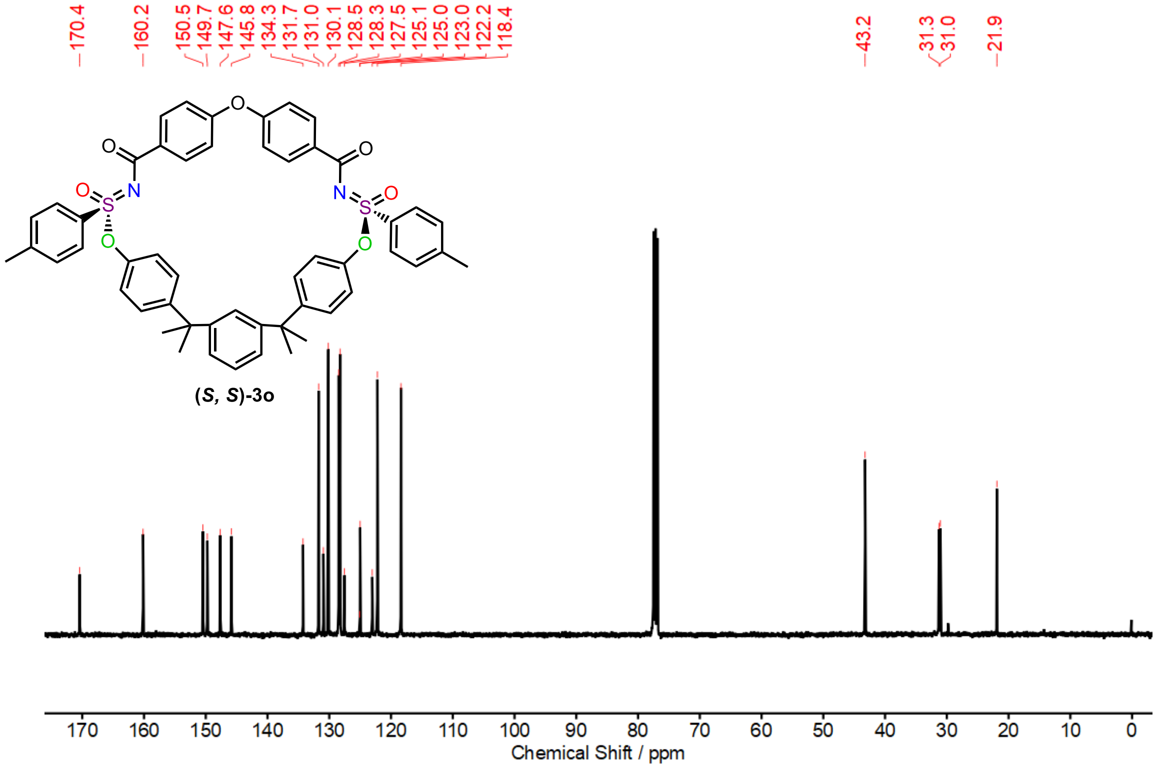

Figure S73. ^{13}C{1H} NMR (101 MHz) spectra of compound ***(S, S)*-3o** ($CDCl_3$, 298 K).

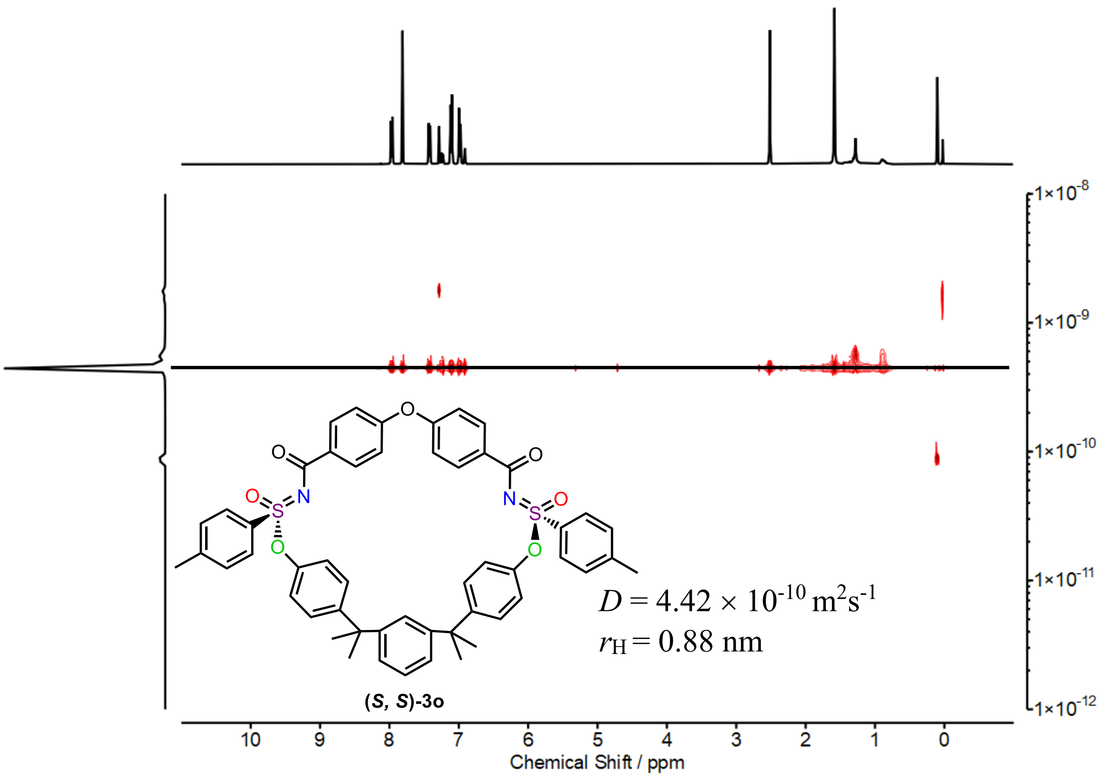

Figure S74. DOSY (400 MHz) spectra of compound ***(S, S)*-3o** ($CDCl_3$, 298 K).

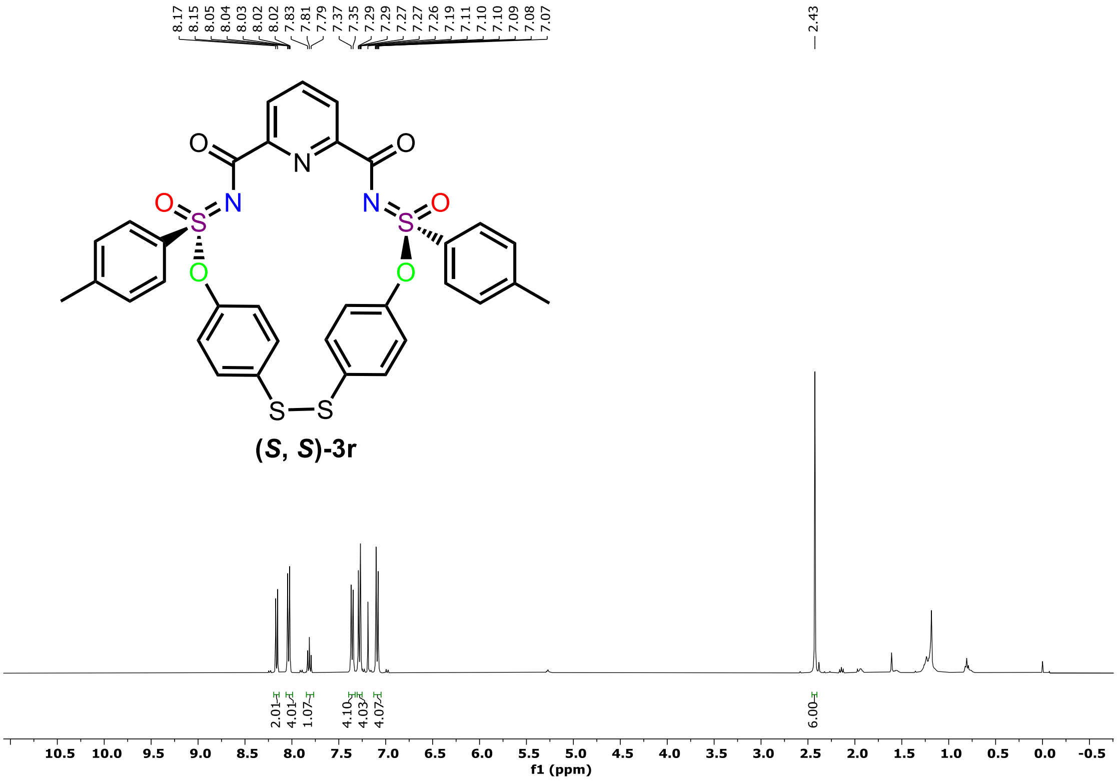

Figure S75. ^{1}H NMR (400 MHz) spectra of compound ***(S, S)*-3r** ($CDCl_3$, 298 K).

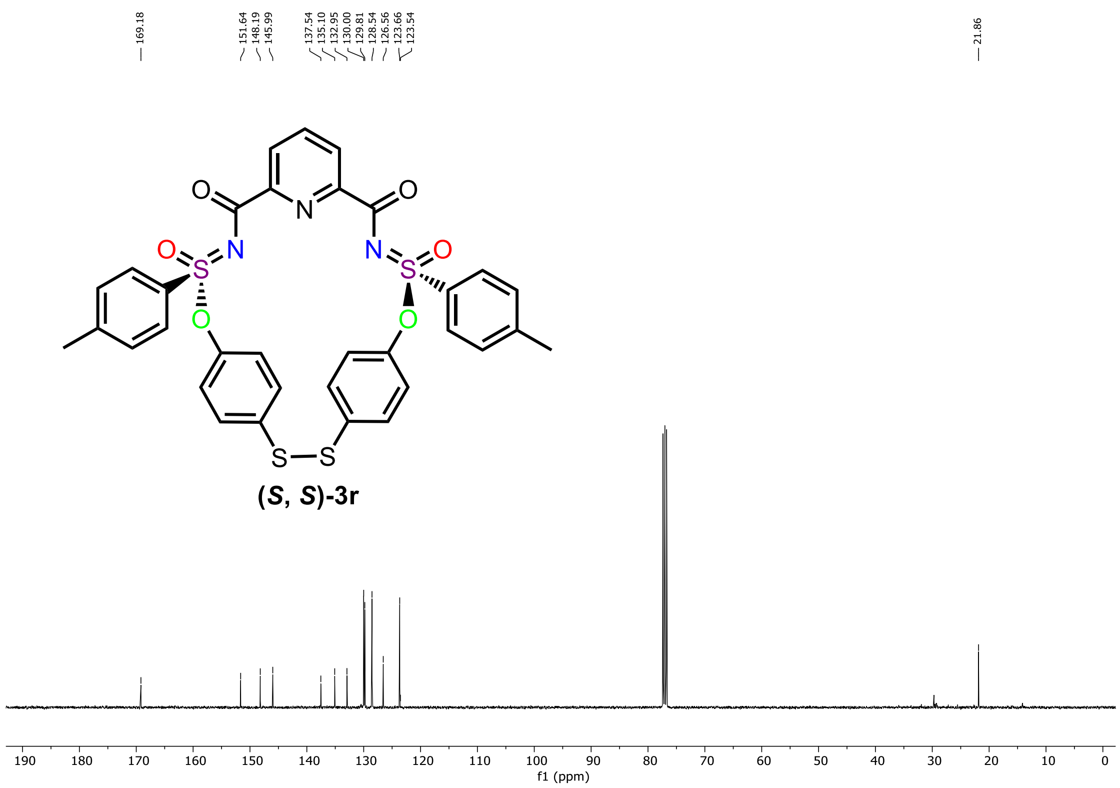

Figure S76.^{13}C{1H} NMR (101 MHz) spectra of compound ***(S, S)*-3r** ($CDCl_3$, 298 K).

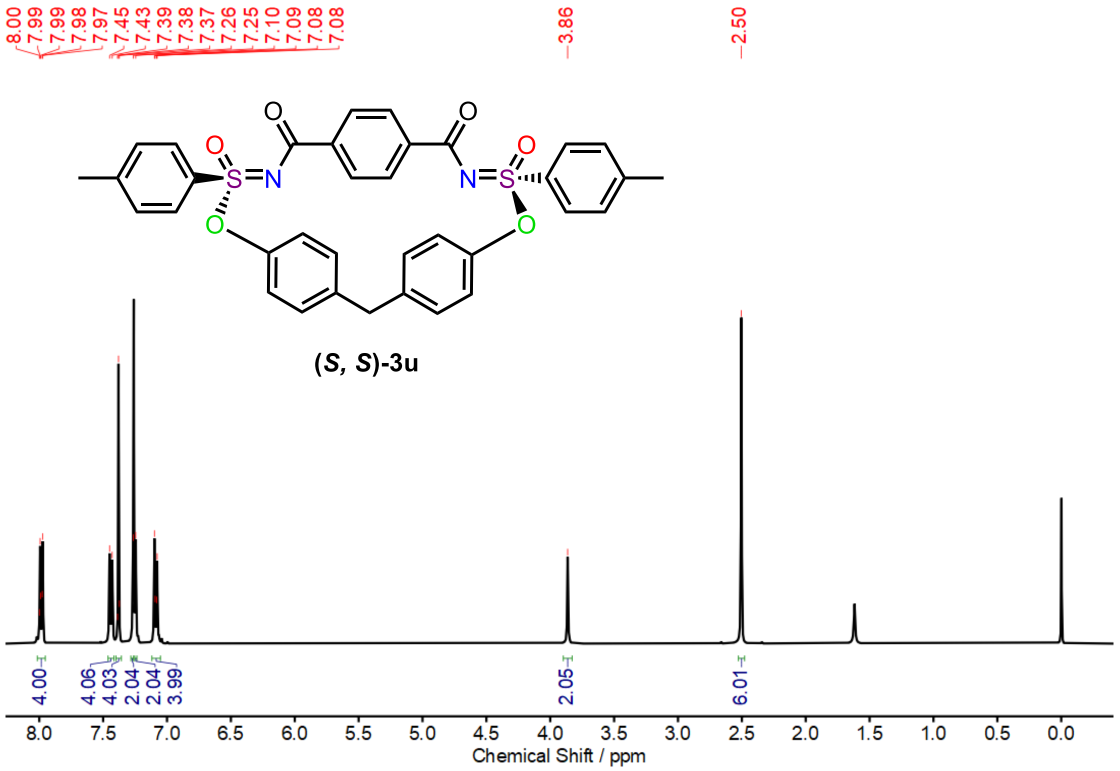

Figure S77. ^{1}H NMR (400 MHz) spectra of compound ***(S, S)*-3u** ($CDCl_3$, 298 K).

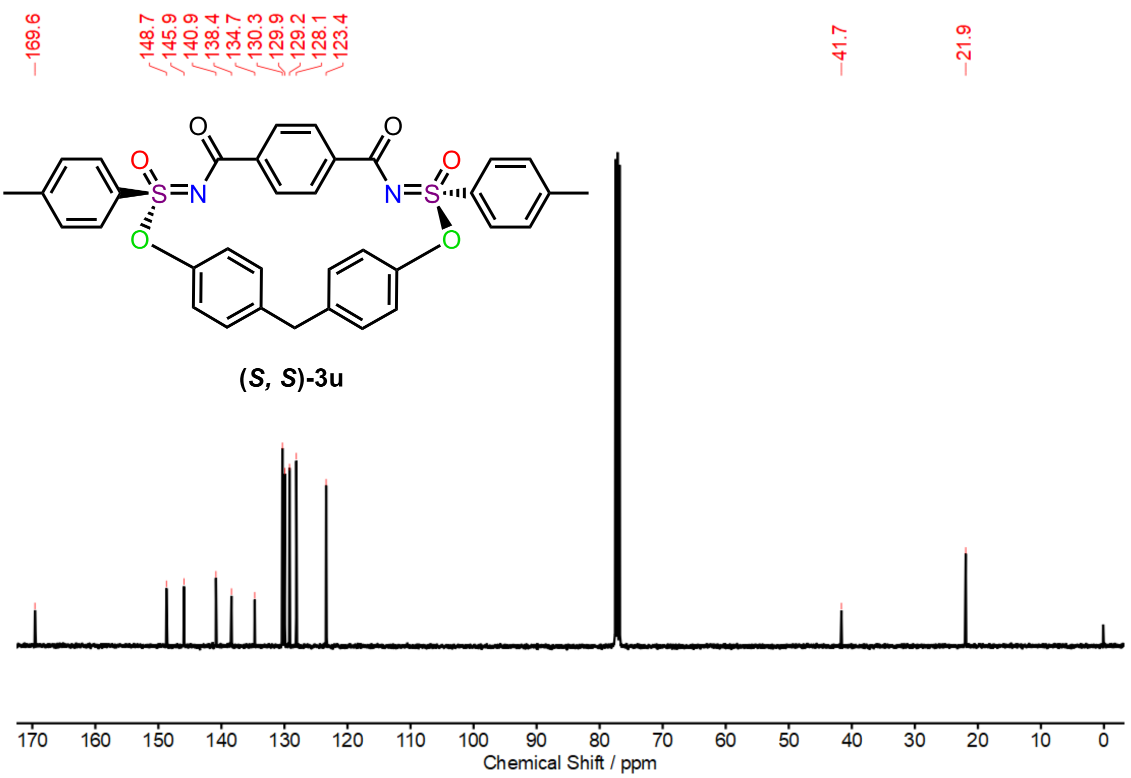

Figure S78. $^{13}C\{1H\}$ NMR (101 MHz) spectra of compound **(*S, S*)-3u** ($CDCl_3$, 298 K).

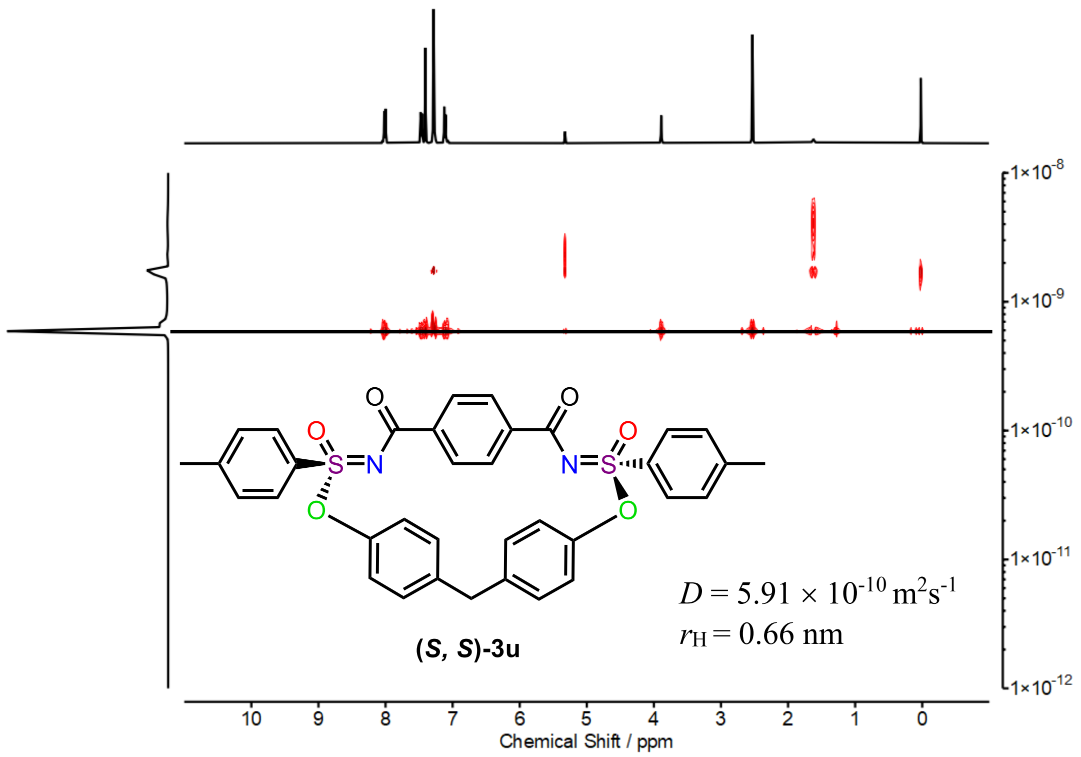

Figure S79. DOSY (400 MHz) spectra of compound **(*S, S*)-3u** ($CDCl_3$, 298 K).

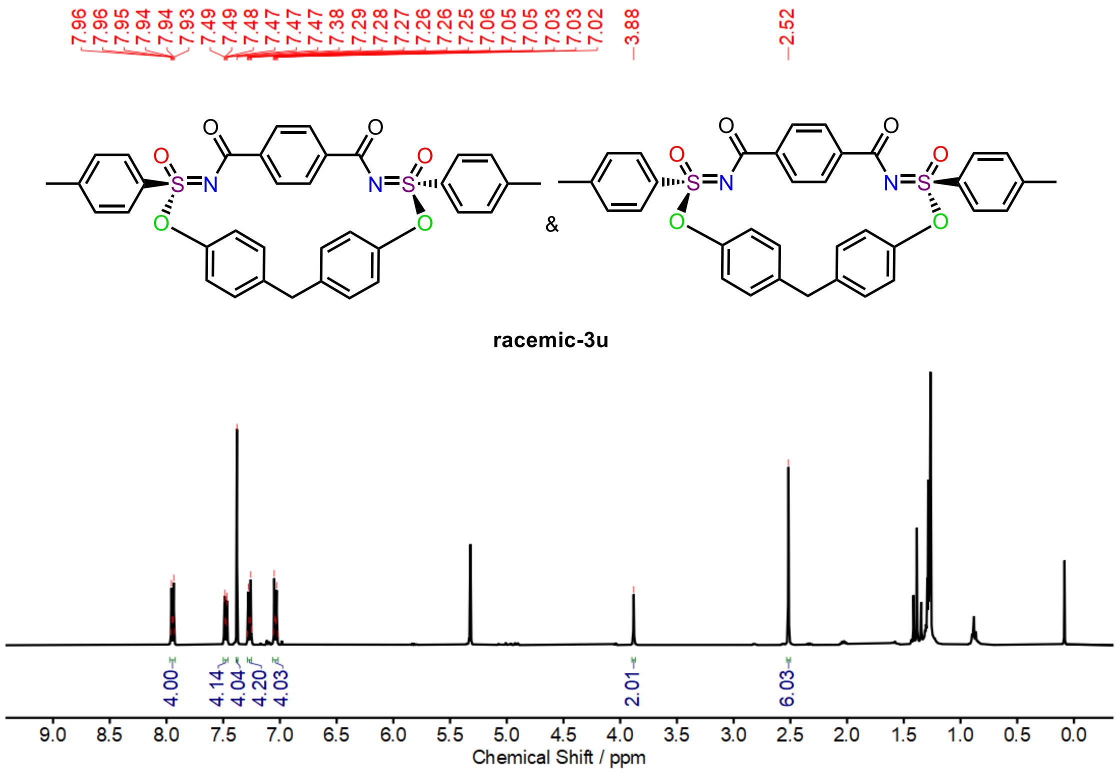

Figure S80. ^{1}H NMR (400 MHz) spectra of compound **racemic-3u** (CD_2Cl_2, 298 K).

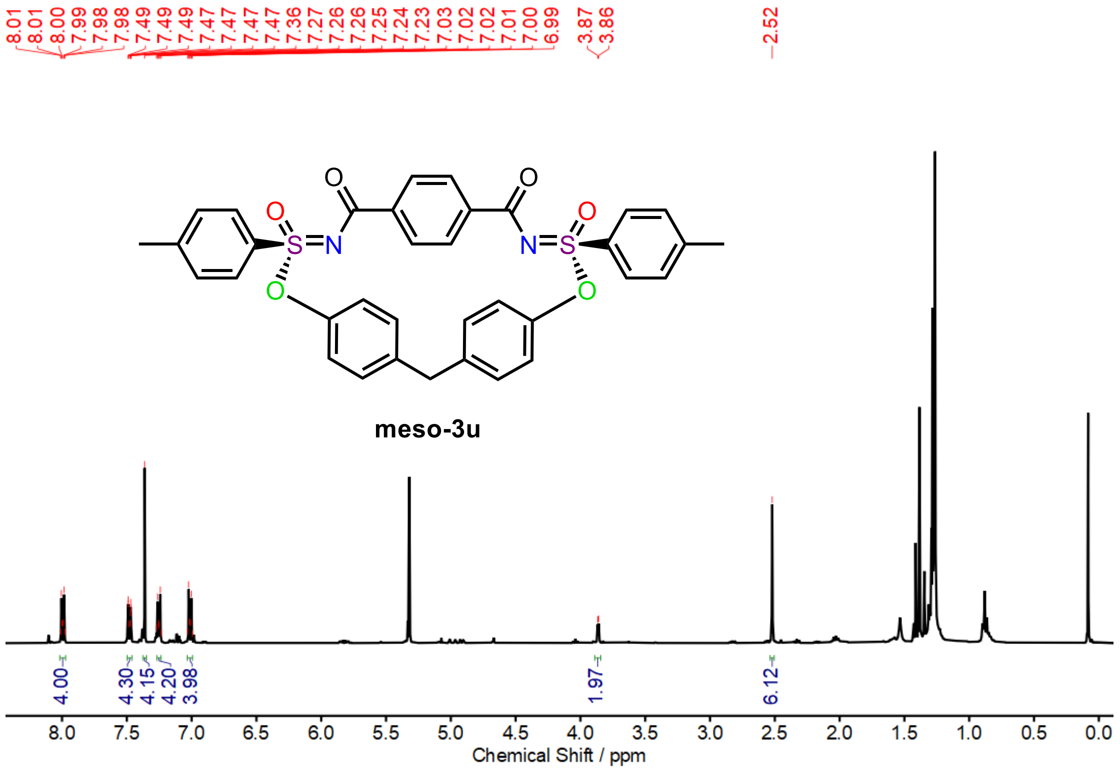

Figure S81. ^{1}H NMR (400 MHz) spectra of compound **meso-3u** (CD_2Cl_2, 298 K).

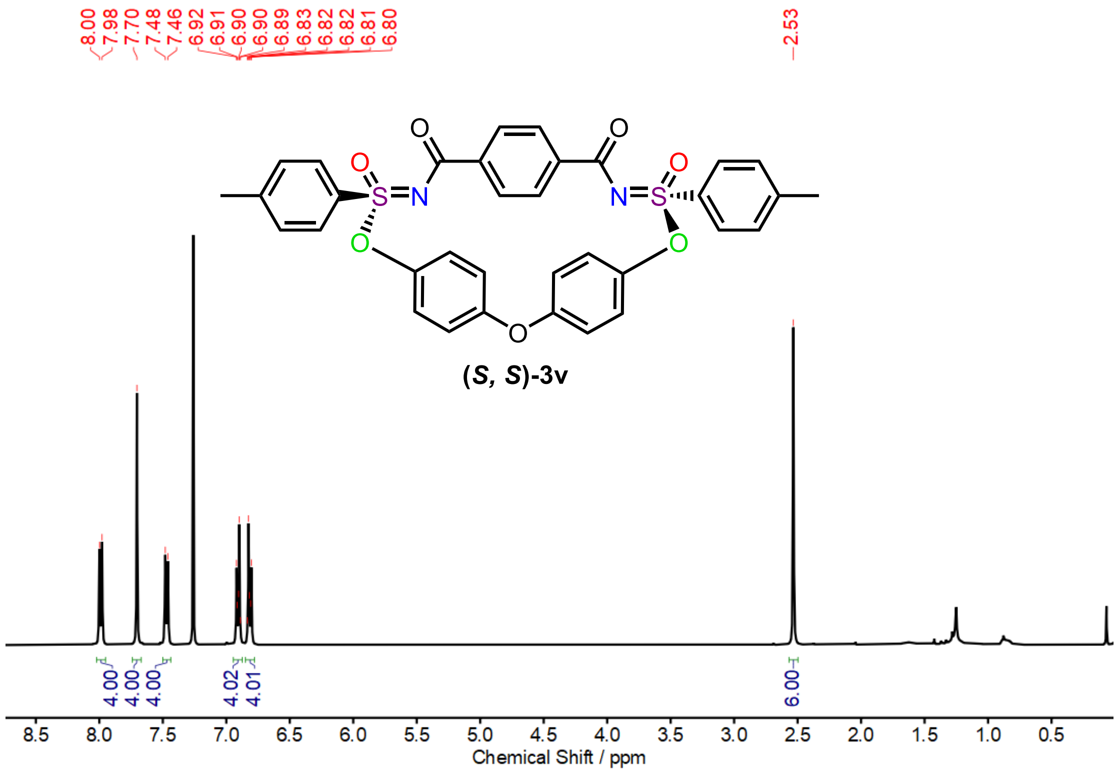

Figure S82. ^{1}H NMR (400 MHz) spectra of compound ***(S, S)*-3v** ($CDCl_3$, 298 K).

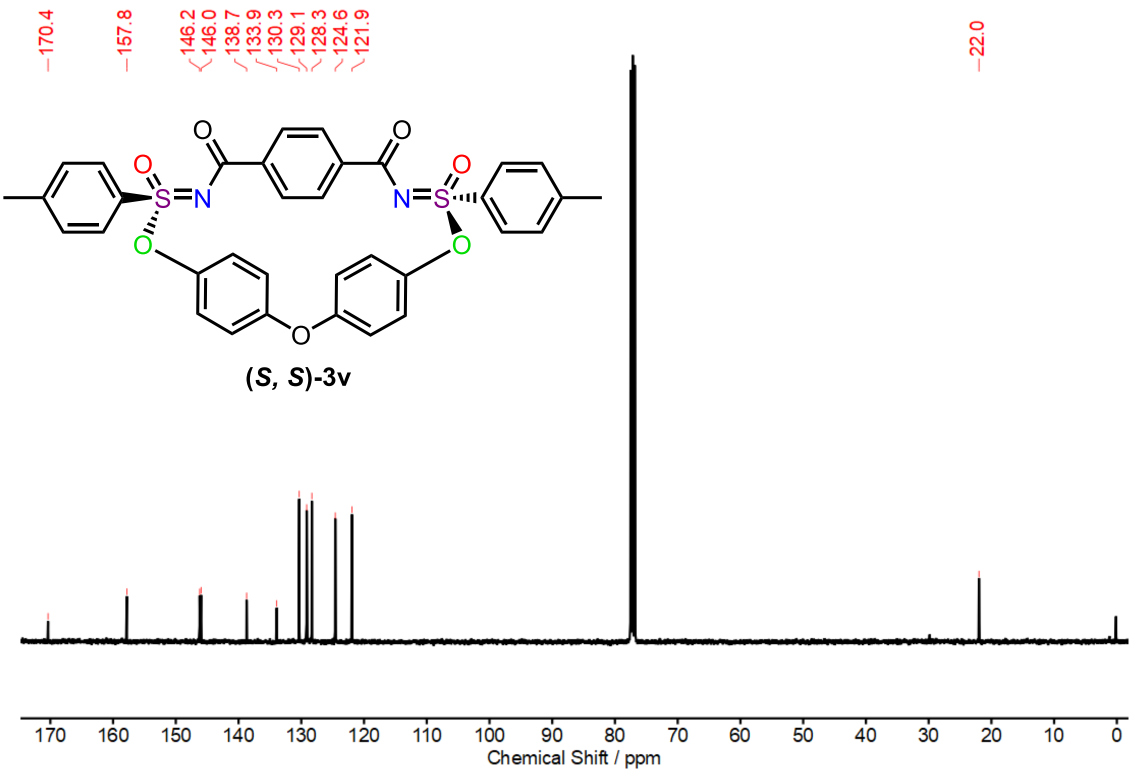

Figure S83. ^{13}C{1H} NMR (101 MHz) spectra of compound ***(S, S)*-3v** ($CDCl_3$, 298 K).

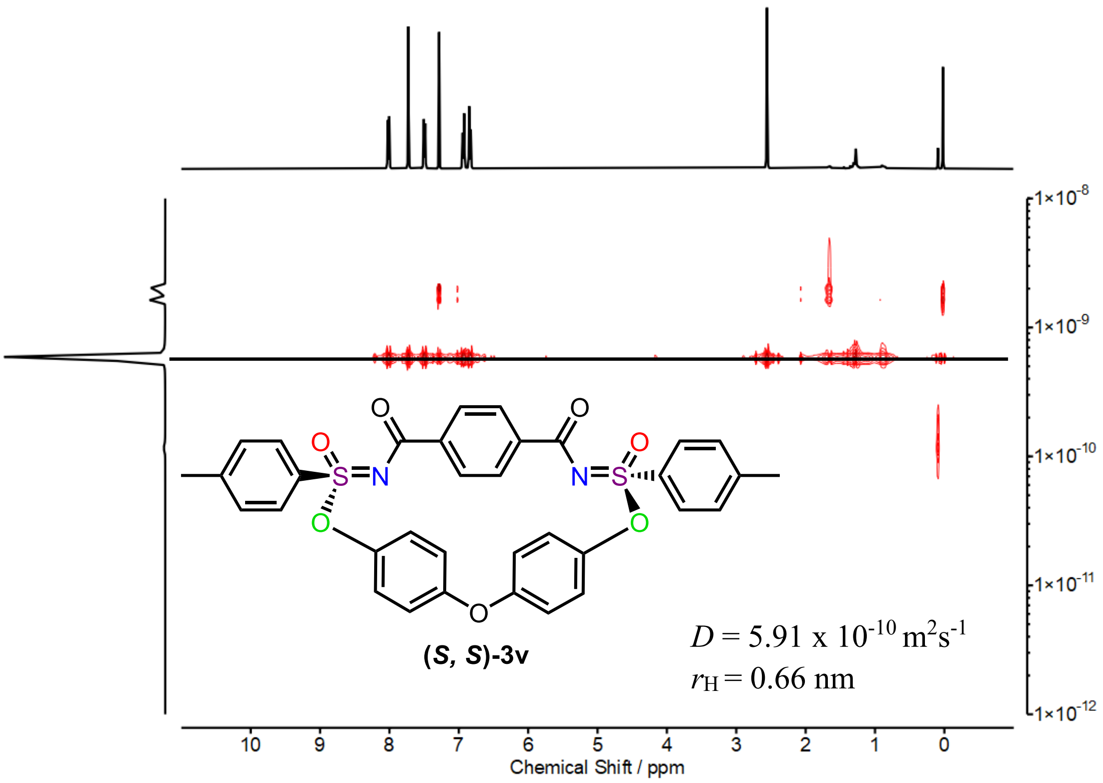

Figure S84. DOSY (400 MHz) spectra of compound ***(S, S)*-3v** ($CDCl_3$, 298 K).

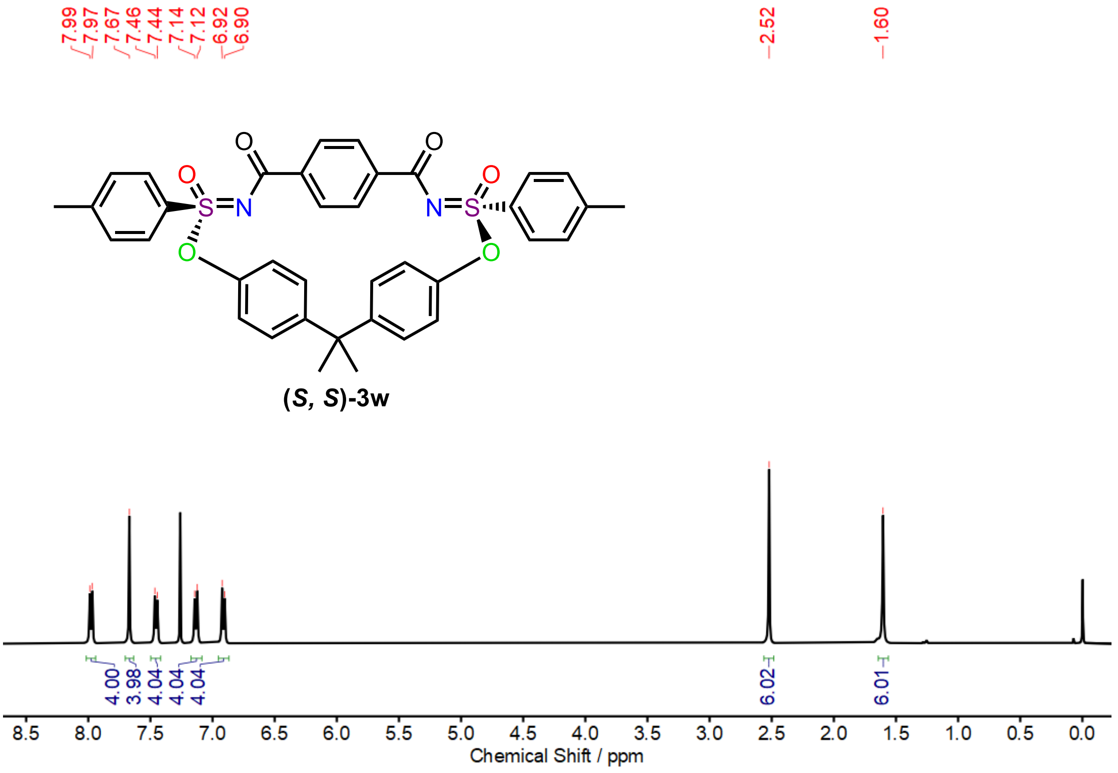

Figure S85. ^{1}H NMR (400 MHz) spectra of compound ***(S, S)*-3w** ($CDCl_3$, 298 K).

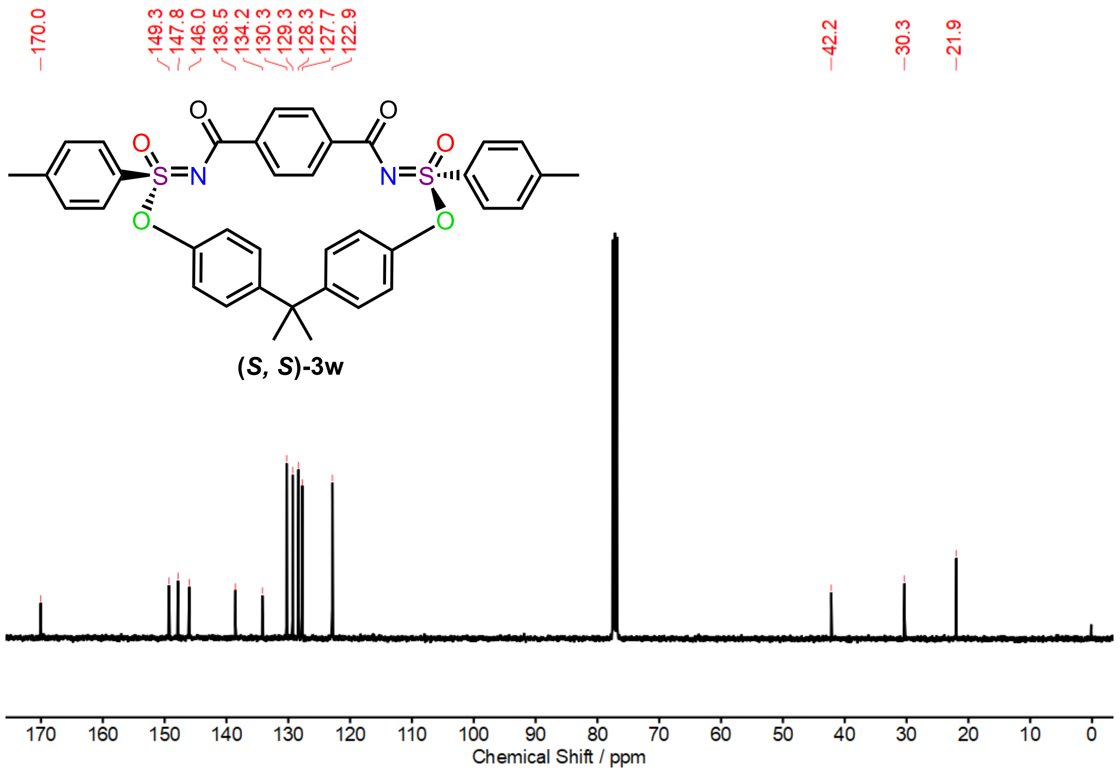

Figure S86. $^{13}C\{1H\}$ NMR (101 MHz) spectra of compound ***(S, S)*-3w** ($CDCl_3$, 298 K).

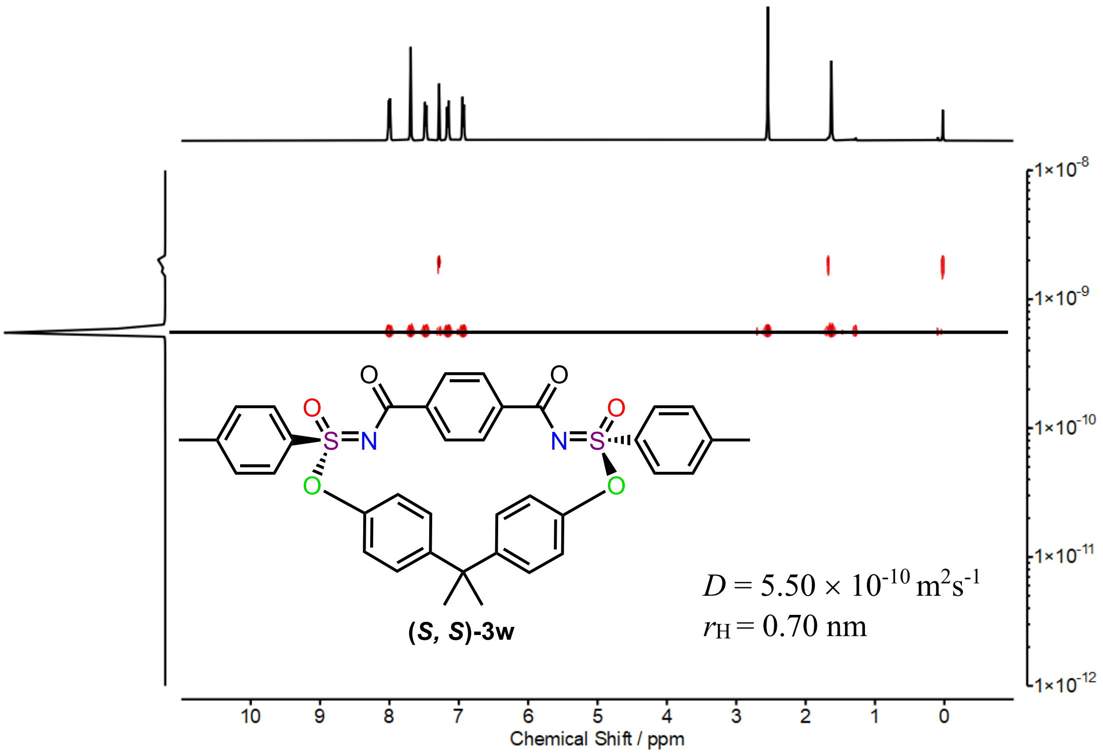

Figure S87. DOSY (400 MHz) spectra of compound ***(S, S)*-3w** ($CDCl_3$, 298 K).

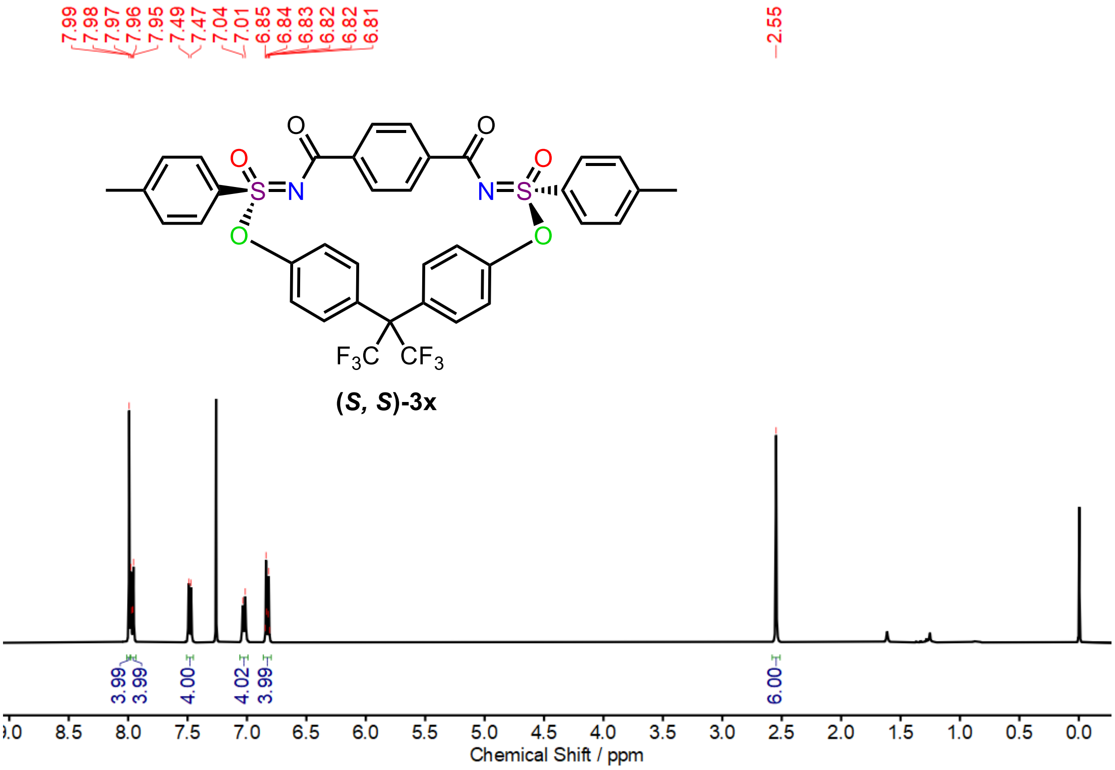

Figure S88. ^{1}H NMR (400 MHz) spectra of compound **(*S, S*)-3x** ($CDCl_3$, 298 K).

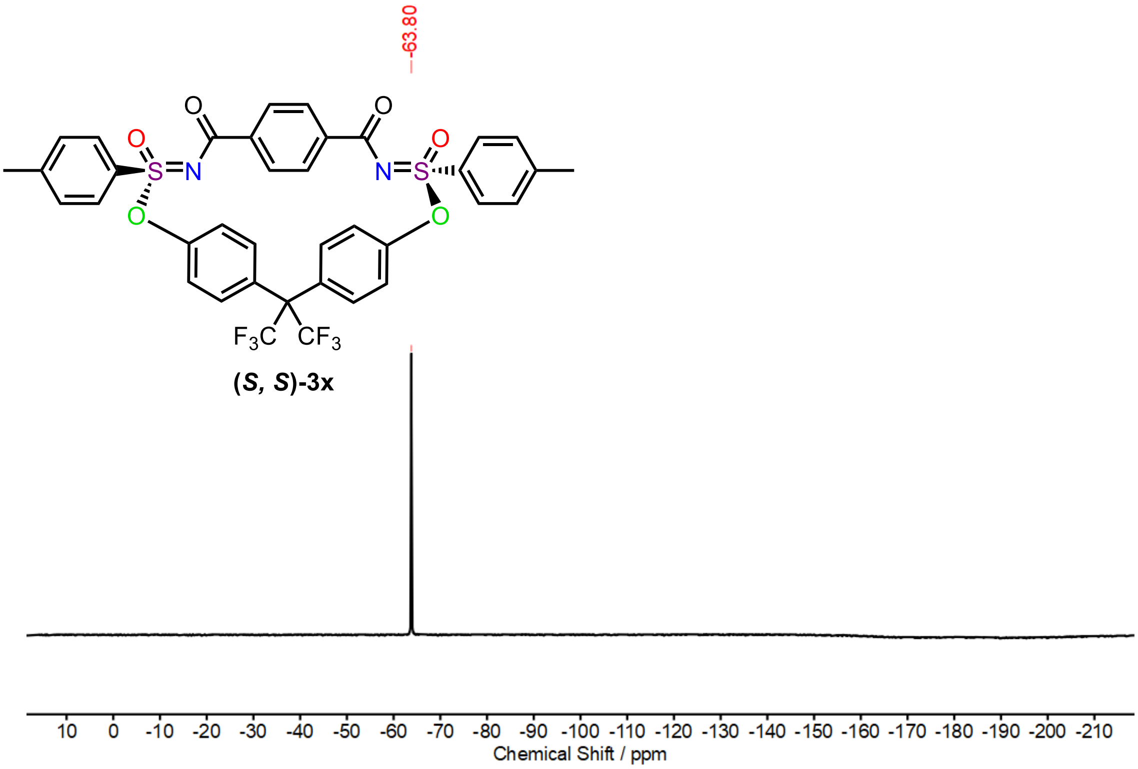

Figure S89. ^{19}F NMR (376 MHz) spectra of compound **(*S, S*)-3x** ($CDCl_3$, 298 K).

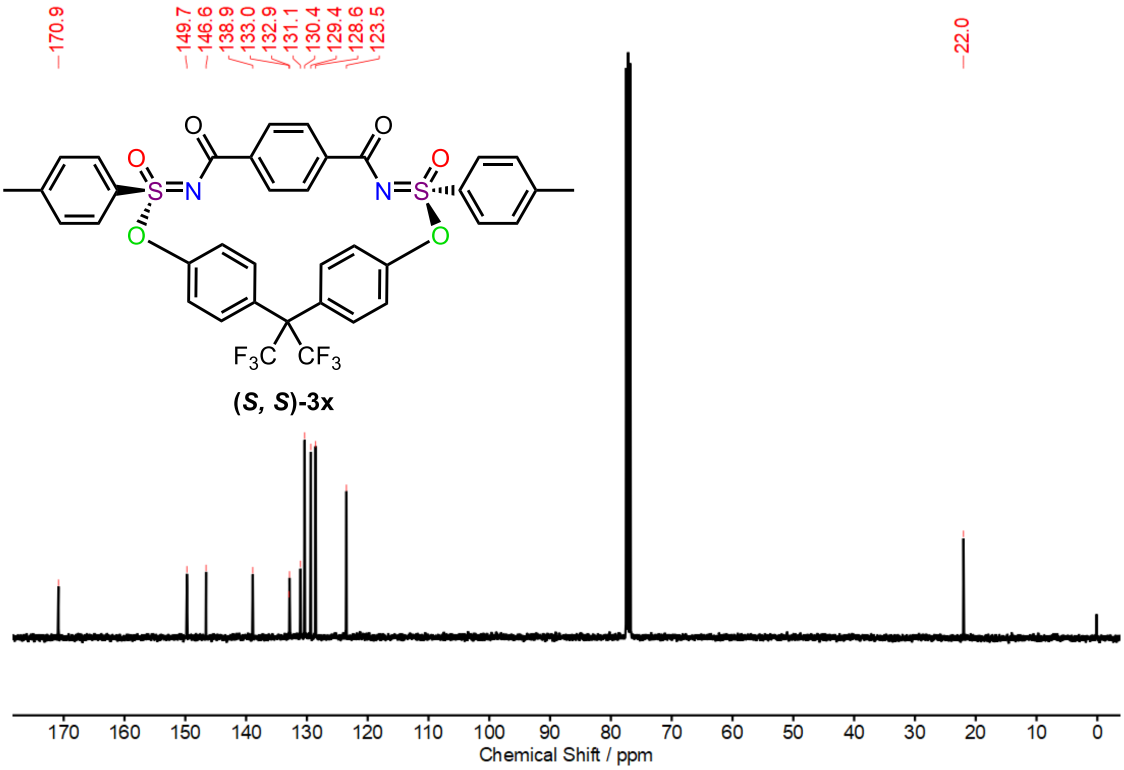

Figure S90. $^{13}C\{1H\}$ NMR (101 MHz) spectra of compound ***(S, S)*-3x** ($CDCl_3$, 298 K).

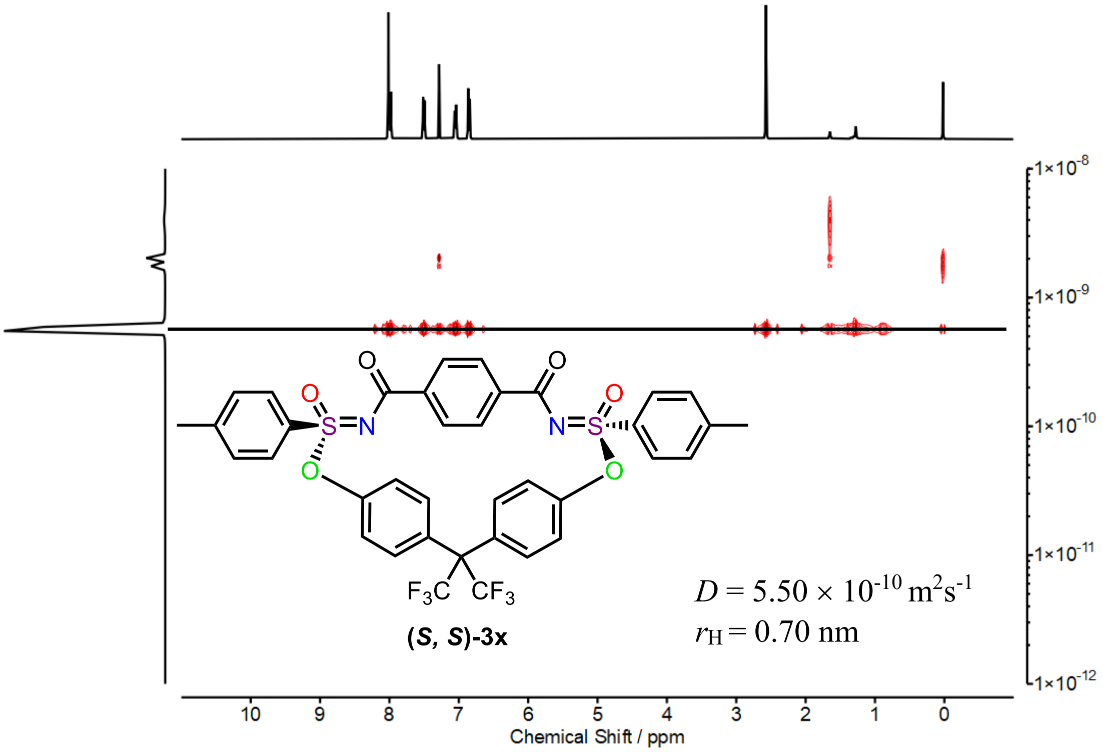

Figure S91. DOSY (400 MHz) spectra of compound ***(S, S)*-3x** ($CDCl_3$, 298 K).

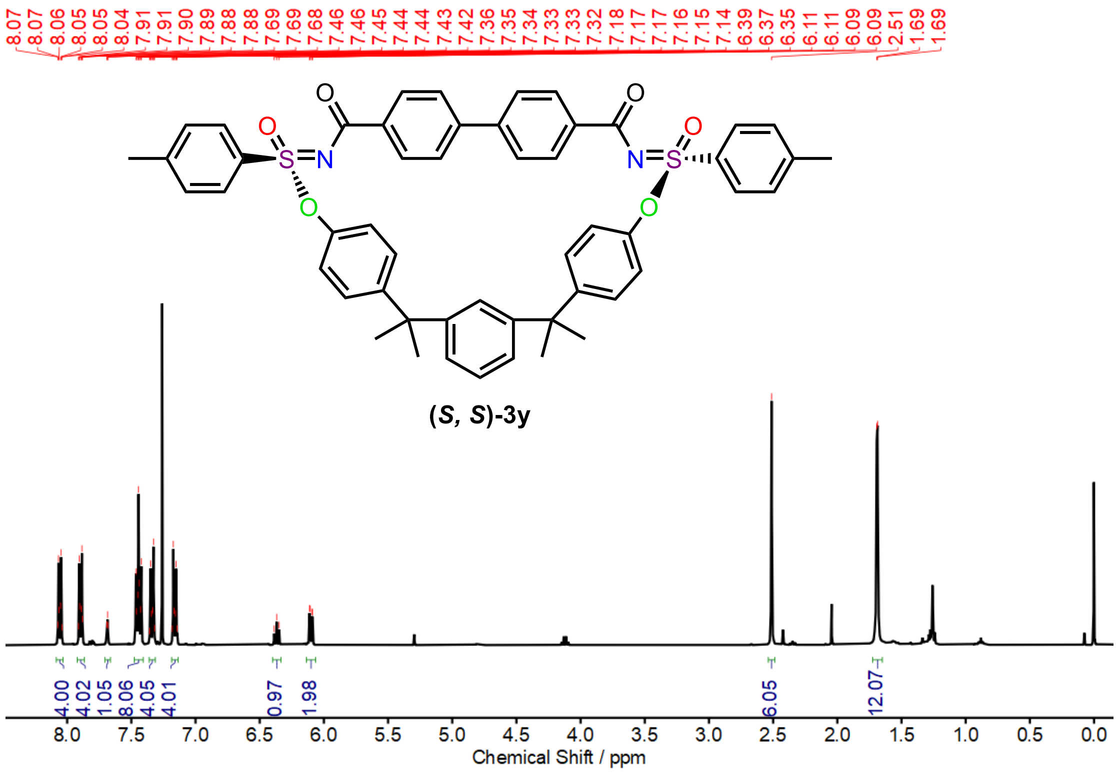

Figure S92. ^{1}H NMR (400 MHz) spectra of compound ***(S, S)*-3y** ($CDCl_3$, 298 K).

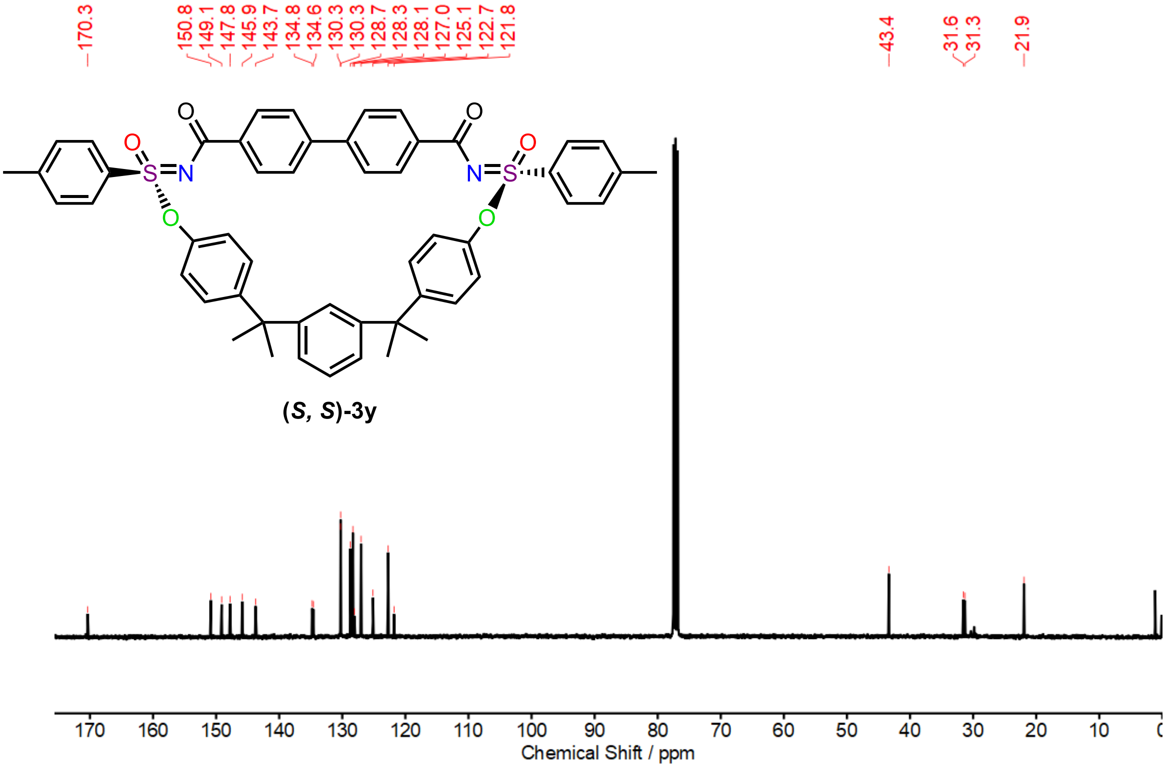

Figure S93. ^{13}C{1H} NMR (101 MHz) spectra of compound ***(S, S)*-3y** ($CDCl_3$, 298 K).

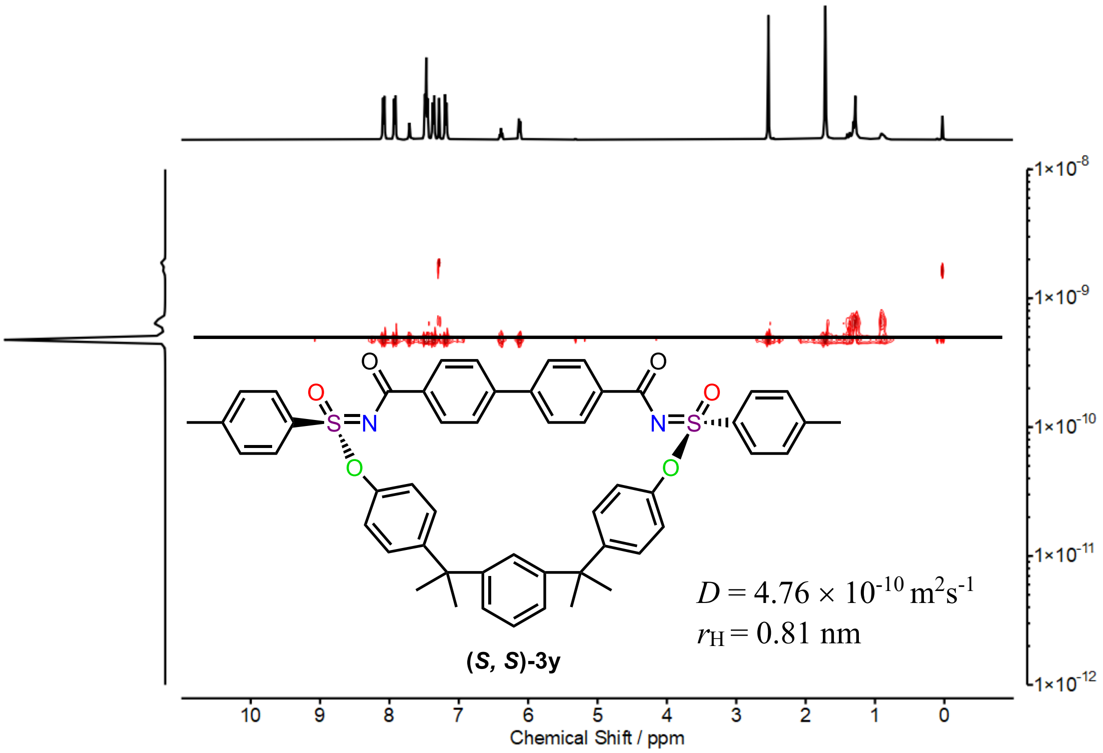

Figure S94. DOSY (400 MHz) spectra of compound ***(S, S)*-3y** ($CDCl_3$, 298 K).

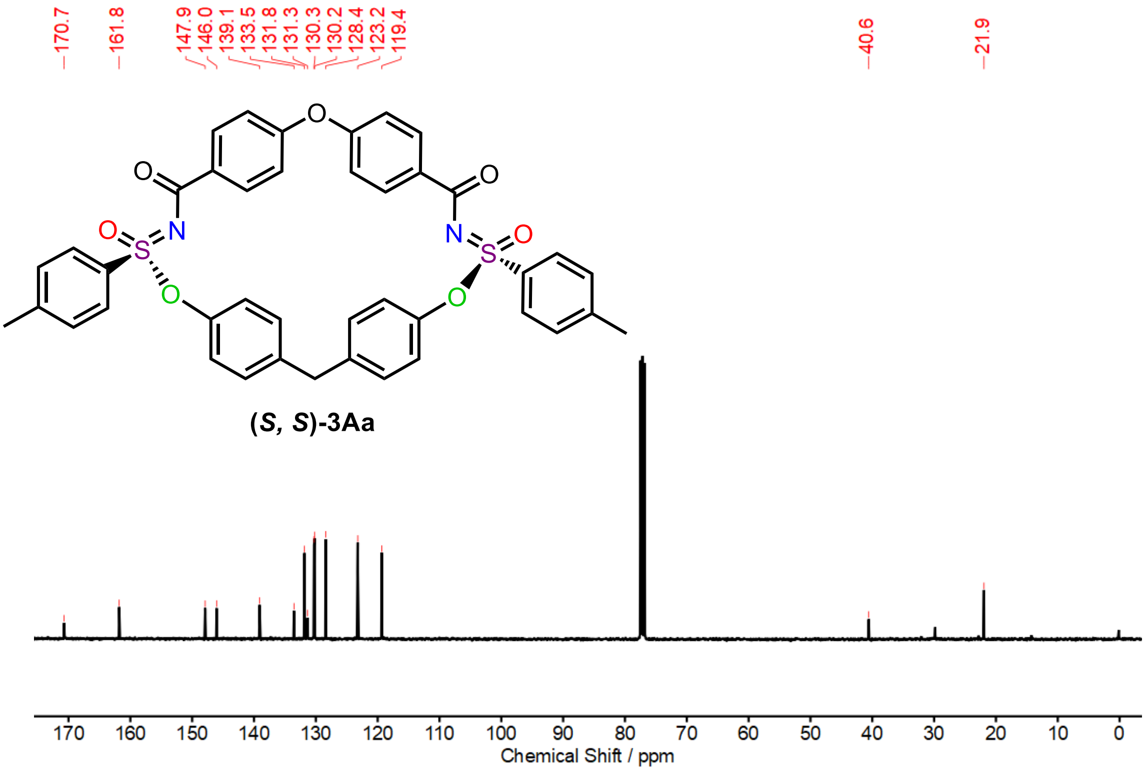

Figure S95. ^{1}H NMR (400 MHz) spectra of compound ***(S, S)*-3Aa** ($CDCl_3$, 298 K).

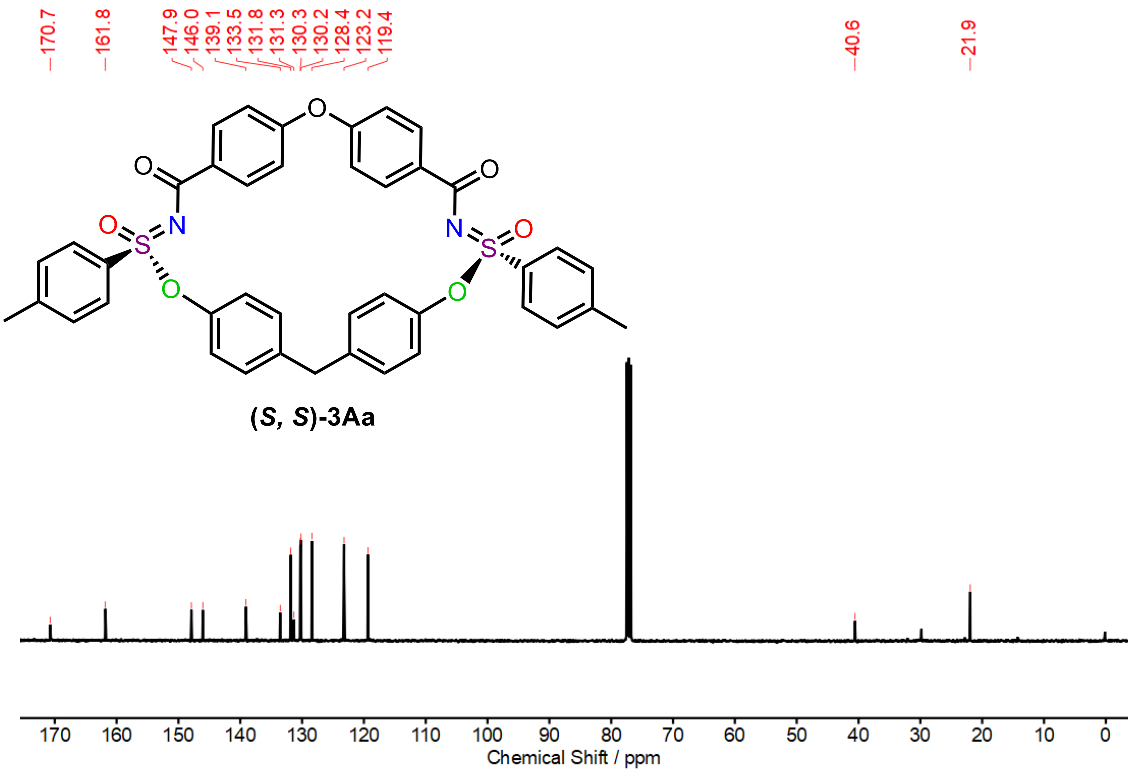

Figure S96. $^{13}C\{1H\}$ NMR (101 MHz) spectra of compound ***(S, S)*-3Aa** ($CDCl_3$, 298 K).

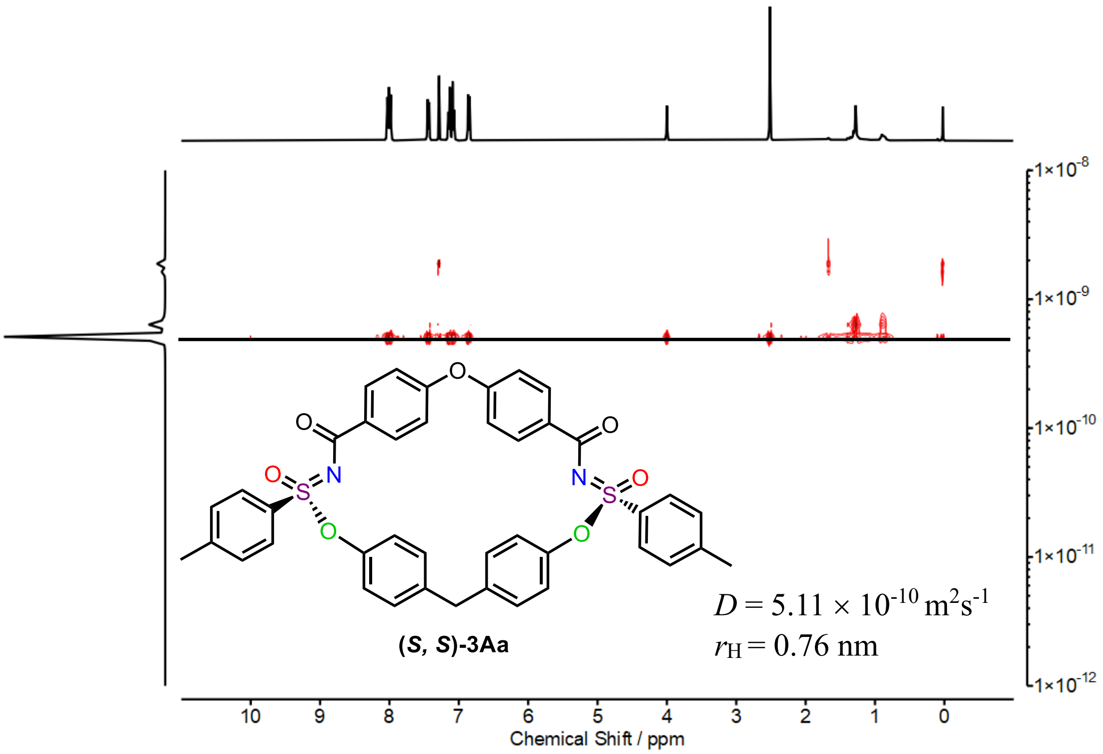

Figure S97. DOSY (400 MHz) spectra of compound ***(S, S)*-3Aa** ($CDCl_3$, 298 K).

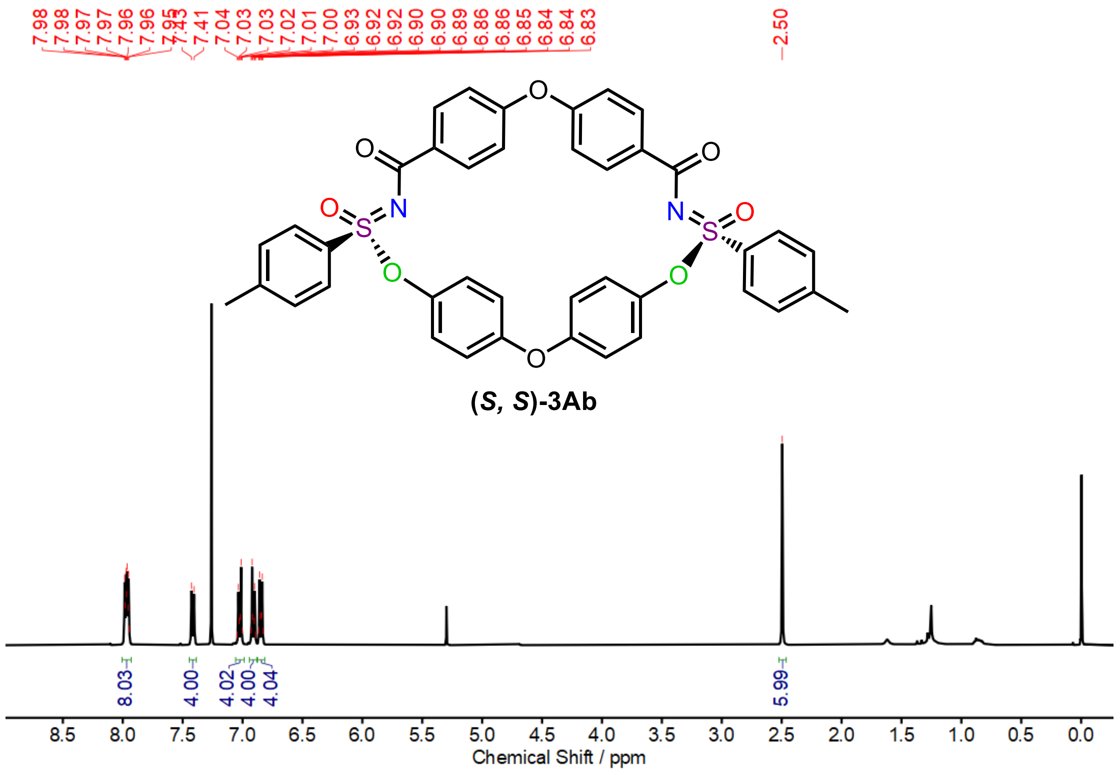

Figure S98. ^{1}H NMR (400 MHz) spectra of compound ***(S, S)*-3Ab** ($CDCl_3$, 298 K).

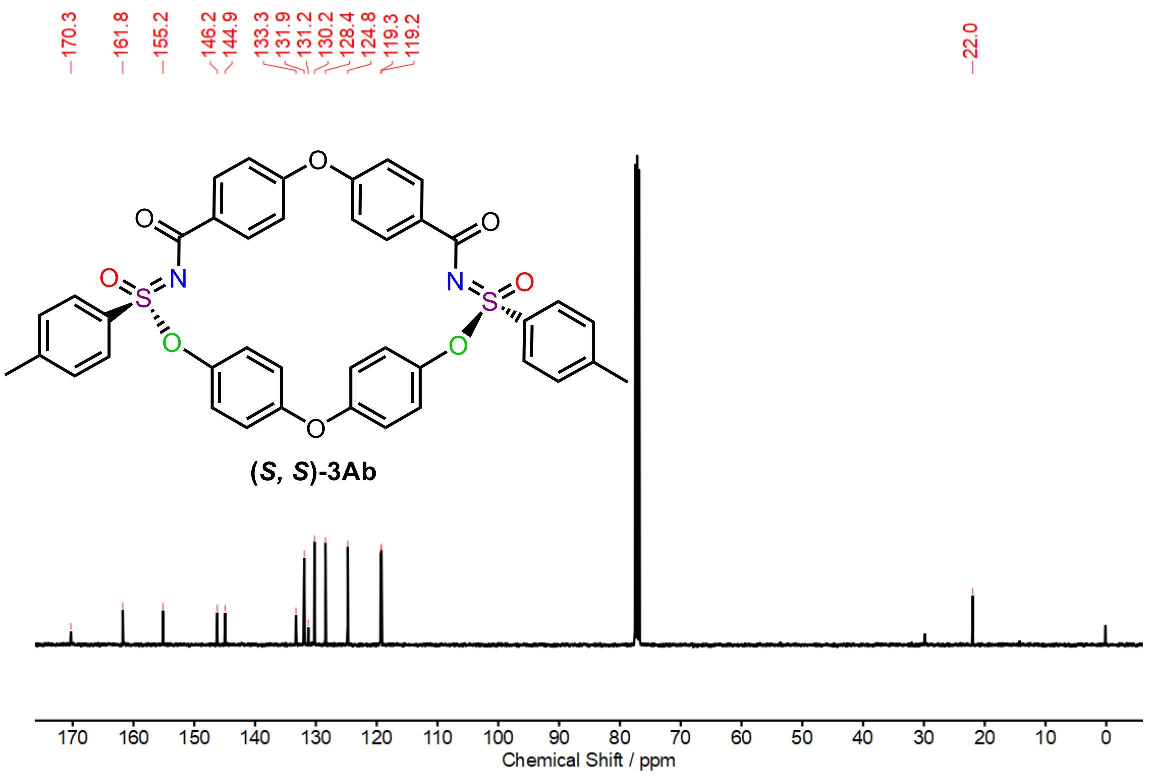

Figure S99. ^{13}C{1H} NMR (101 MHz) spectra of compound ***(S, S)*-3Ab** ($CDCl_3$, 298 K).

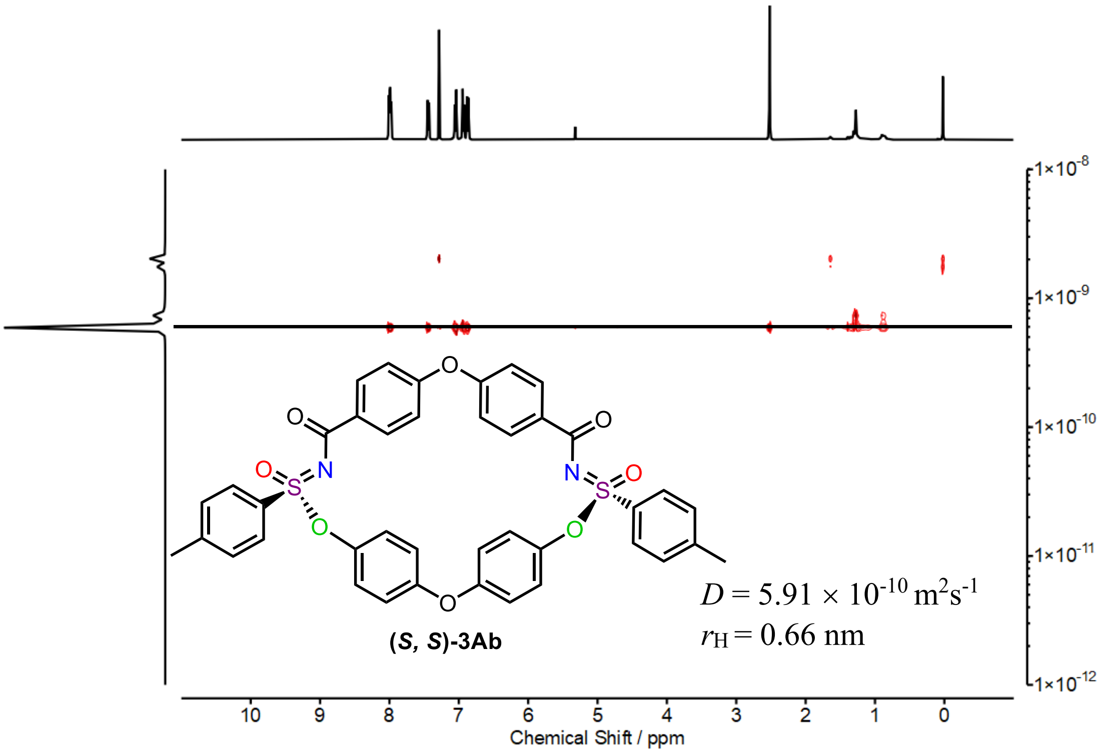

Figure S100. DOSY (400 MHz) spectra of compound **(*S, S*)-3Ab** ($CDCl_3$, 298 K).

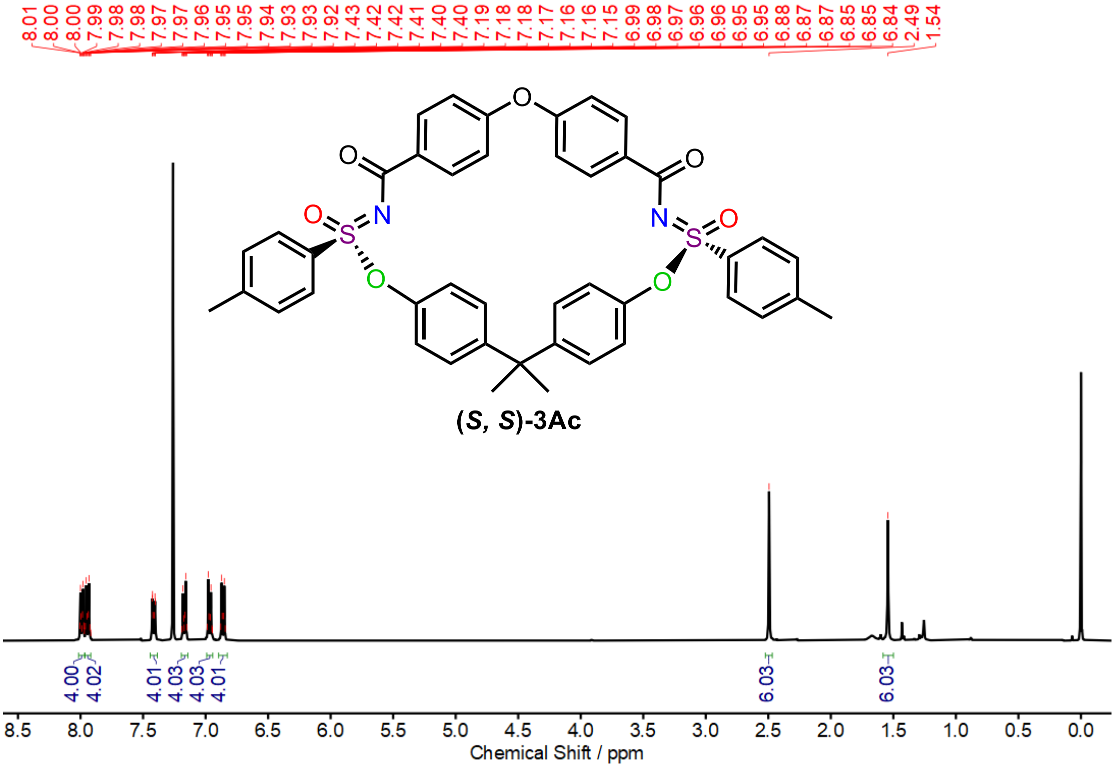

Figure S101. ^{1}H NMR (400 MHz) spectra of compound **(*S, S*)-3Ac** ($CDCl_3$, 298 K).

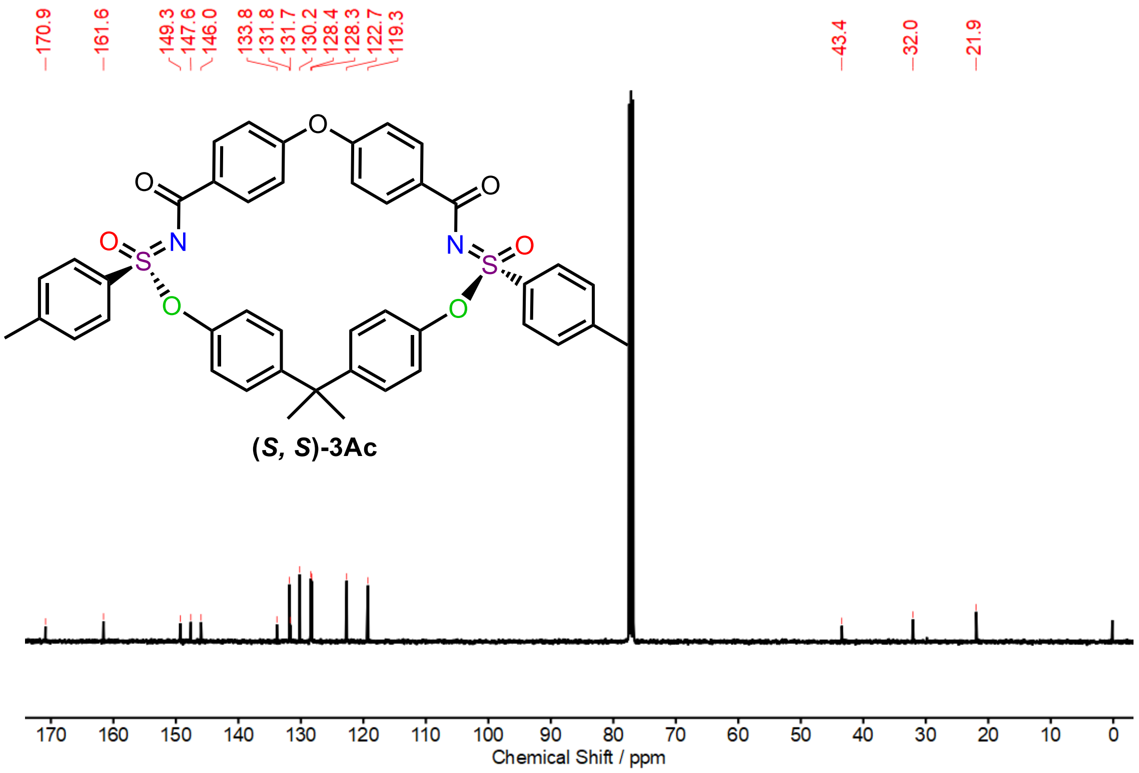

Figure S102. ^{13}C{1H} NMR (101 MHz) spectra of compound ***(S, S)*-3Ac** ($CDCl_3$, 298 K).

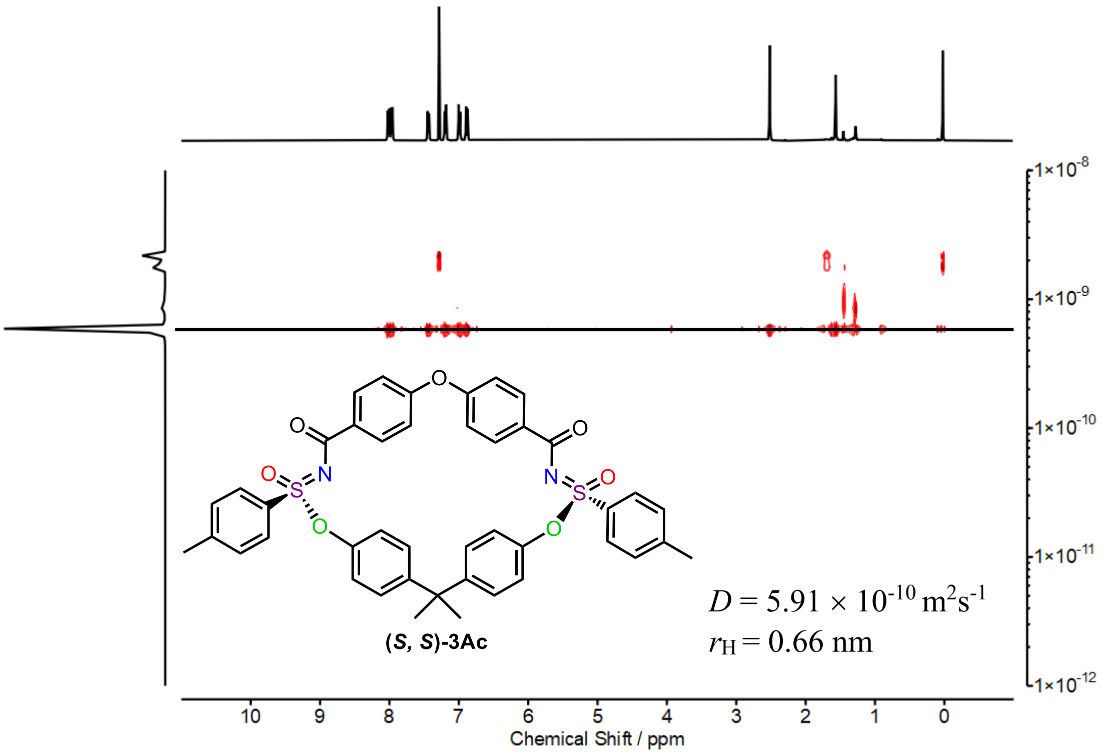

Figure S103. DOSY (400 MHz) spectra of compound ***(S, S)*-3Ac** ($CDCl_3$, 298 K).

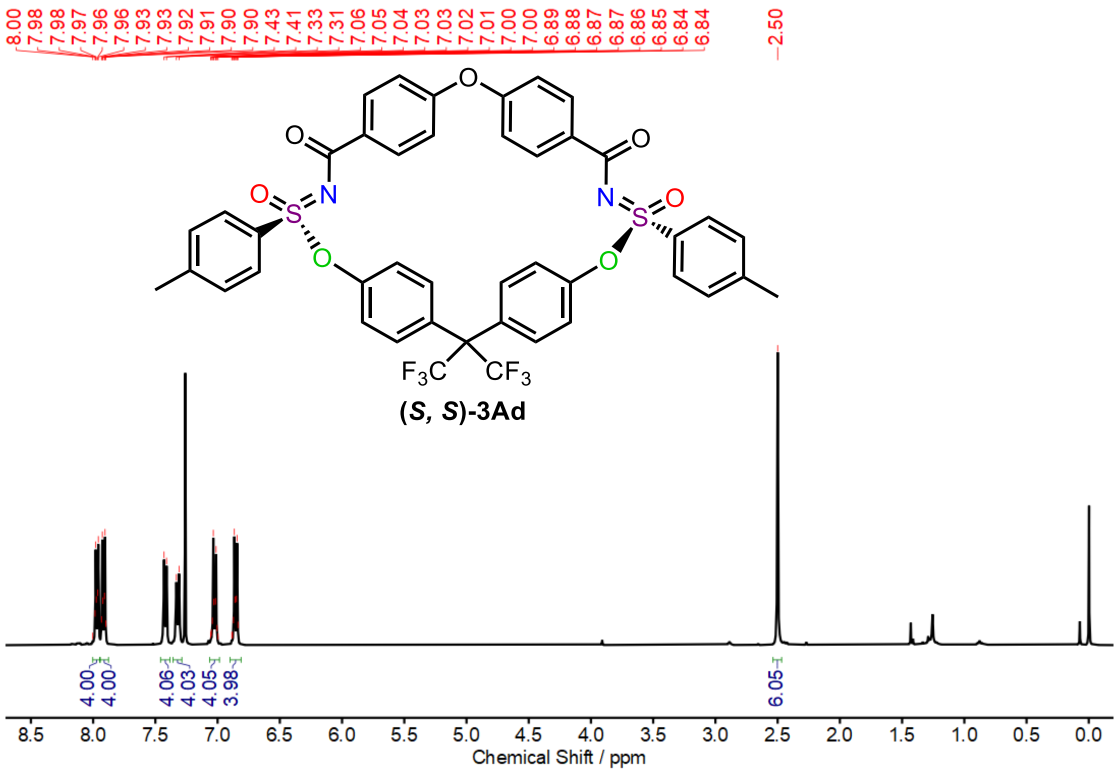

Figure S104. ^{1}H NMR (400 MHz) spectra of compound **(*S, S*)-3Ad** ($CDCl_3$, 298 K).

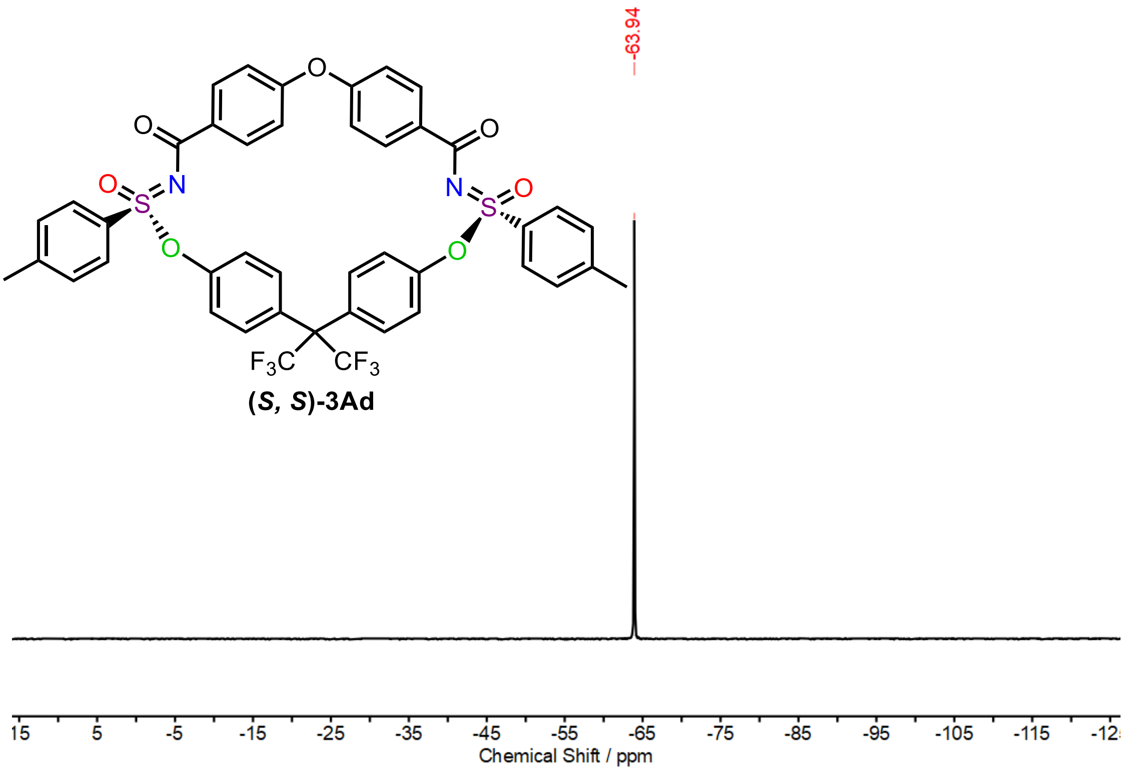

Figure S105. ^{19}F NMR (376 MHz) spectra of compound **(*S, S*)-3Ad** ($CDCl_3$, 298 K).

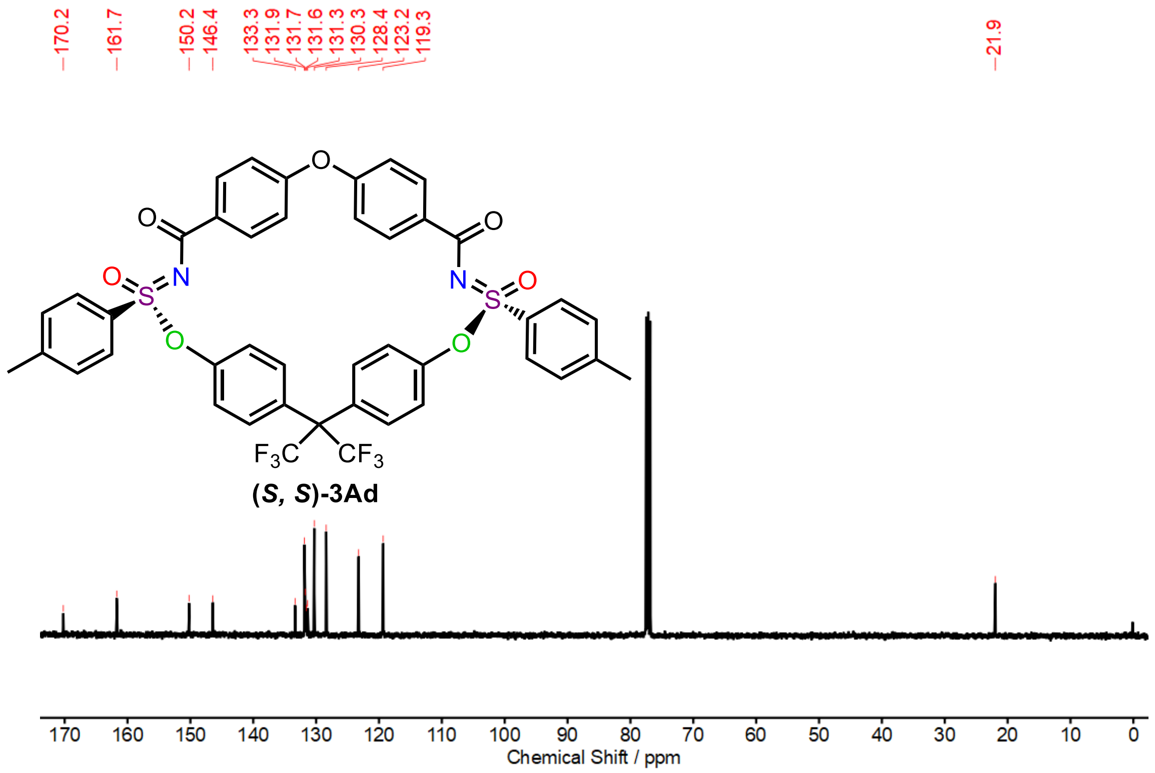

Figure S106. $^{13}C\{1H\}$ NMR (101 MHz) spectra of compound **(*S, S*)-3Ad** ($CDCl_3$, 298 K).

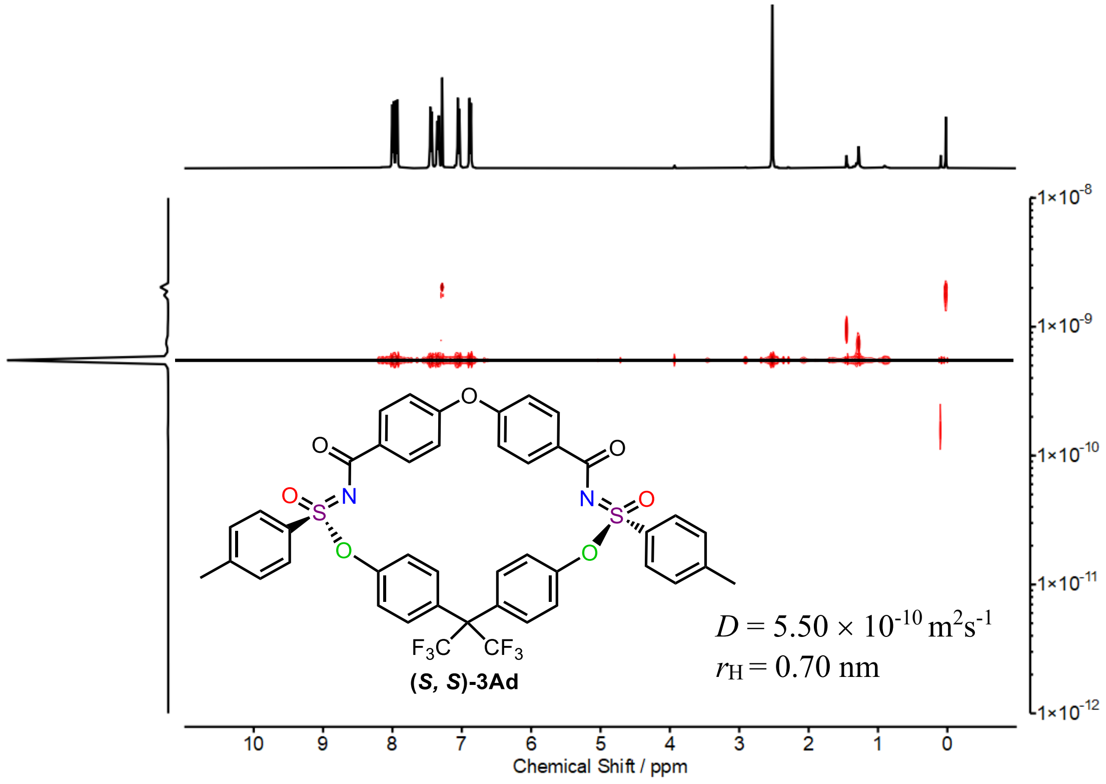

Figure S107. DOSY (400 MHz) spectra of compound **(*S, S*)-3Ad** ($CDCl_3$, 298 K)

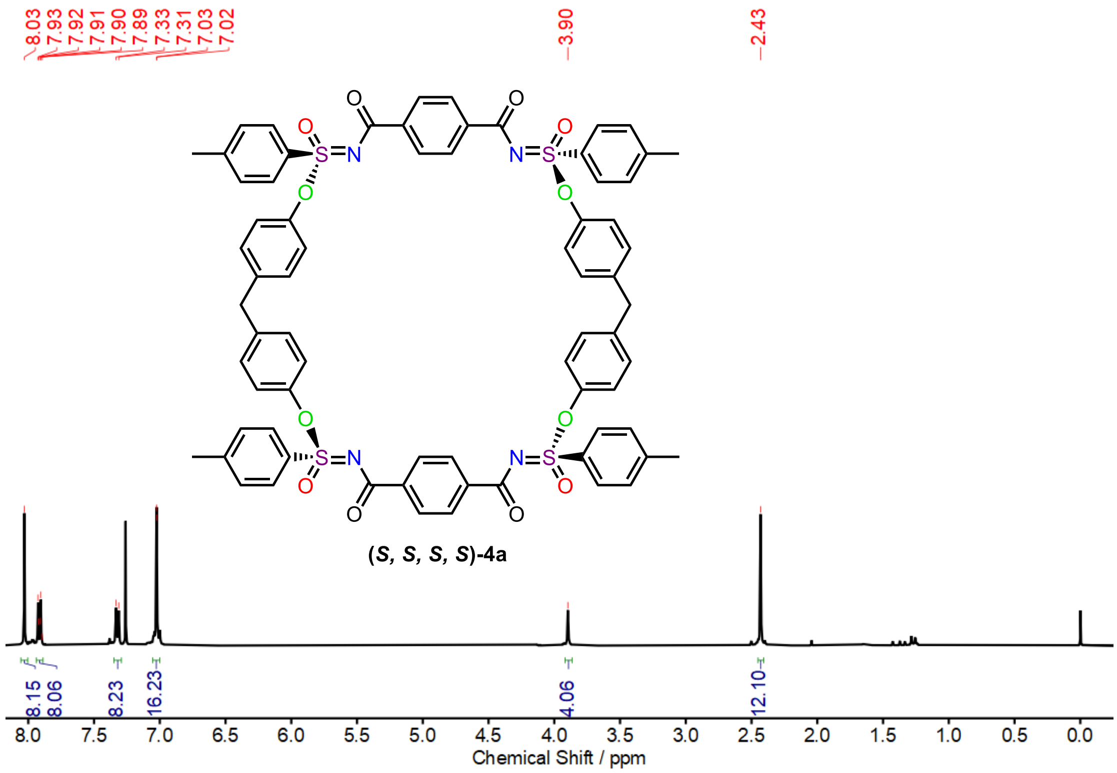

Figure S108. ^{1}H NMR (400 MHz) spectra of compound **(*S*, *S*, *S*, *S*)-4a** ($CDCl_3$, 298 K).

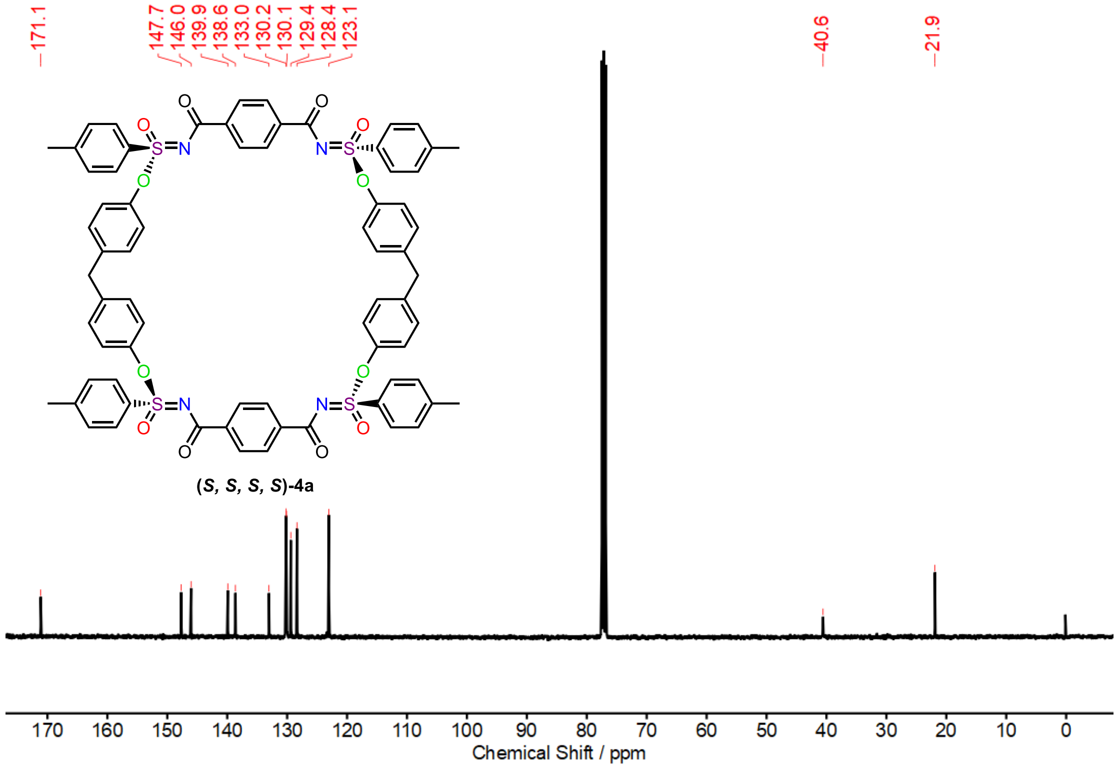

Figure S109. ^{13}C{1H} NMR (101 MHz) spectra of compound **(*S*, *S*, *S*, *S*)-4a** ($CDCl_3$, 298 K).

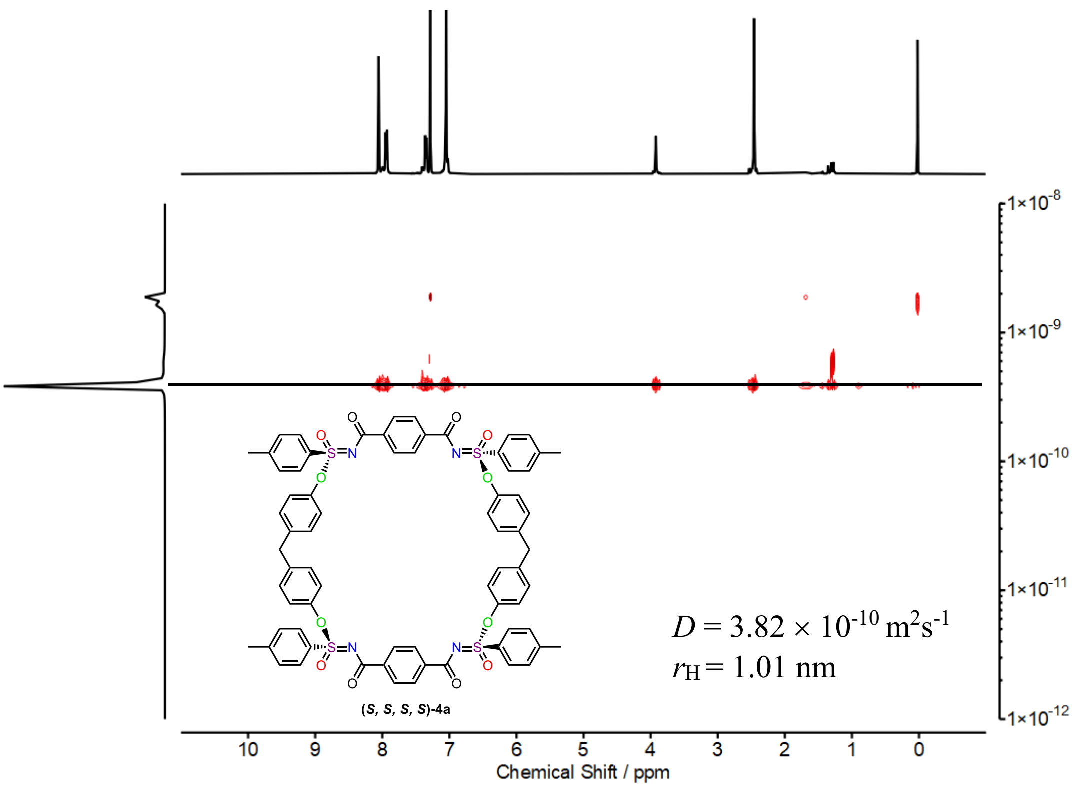

Figure S110. DOSY (400 MHz) spectra of compound **(*S, S, S, S*)-4a** ($CDCl_3$, 298 K).

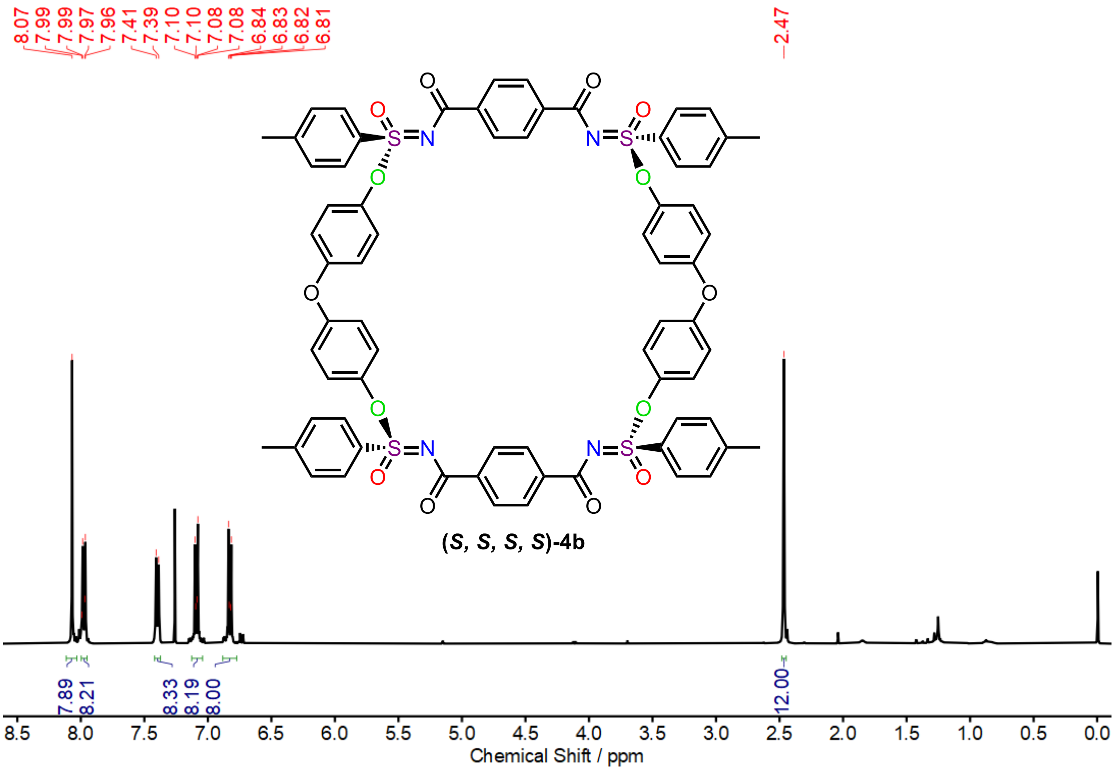

Figure S111. ^{1}H NMR (400 MHz) spectra of compound **(*S, S, S, S*)-4b** ($CDCl_3$, 298 K).

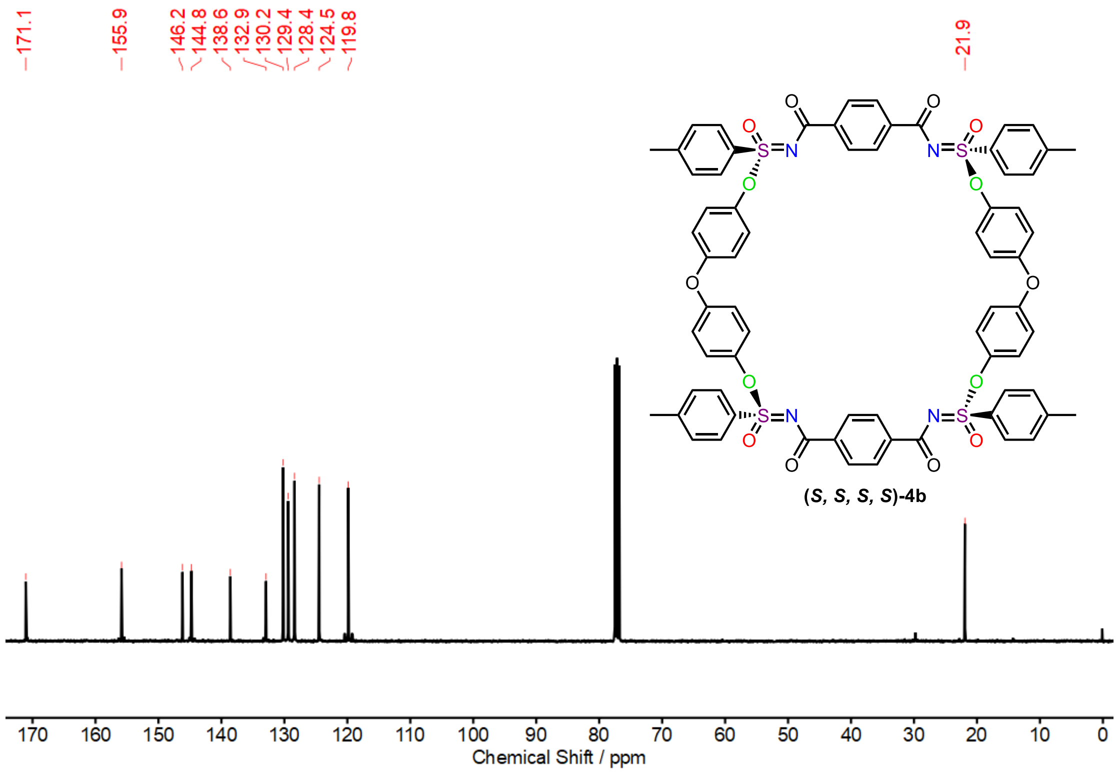

Figure S112. $^{13}C\{1H\}$ NMR (101 MHz) spectra of compound ***(S, S, S, S)*-4b** ($CDCl_3$, 298 K).

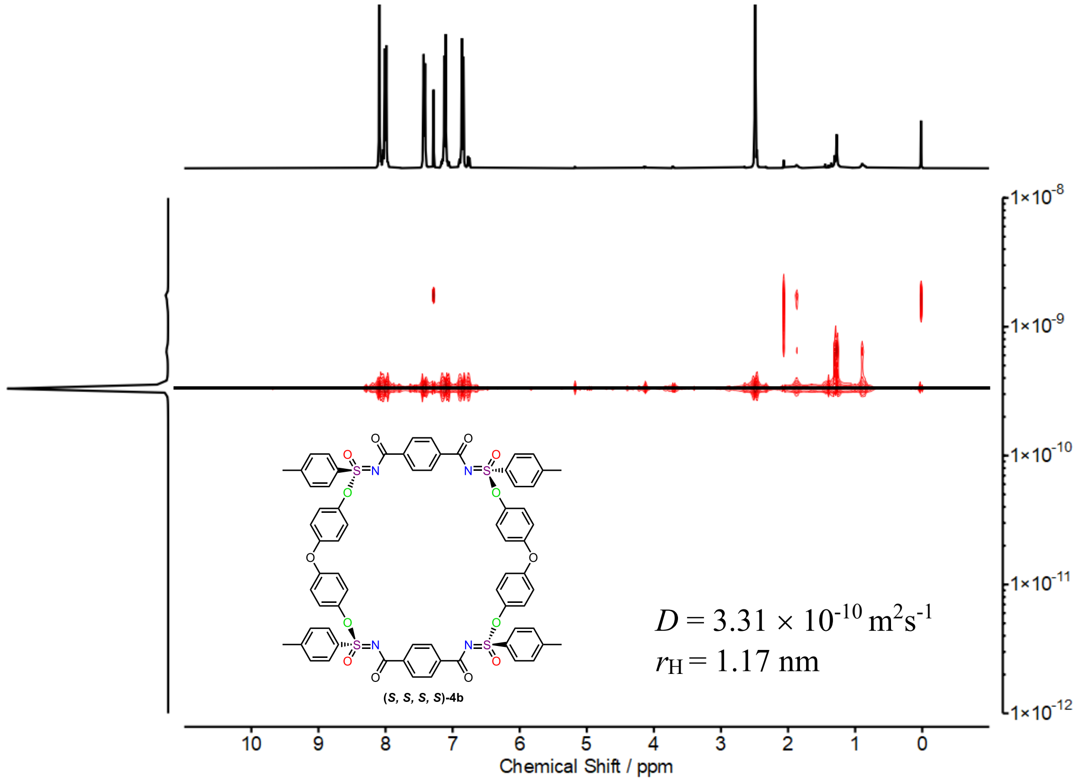

Figure S113. DOSY (400 MHz) spectra of compound ***(S, S, S, S)*-4b** ($CDCl_3$, 298 K).

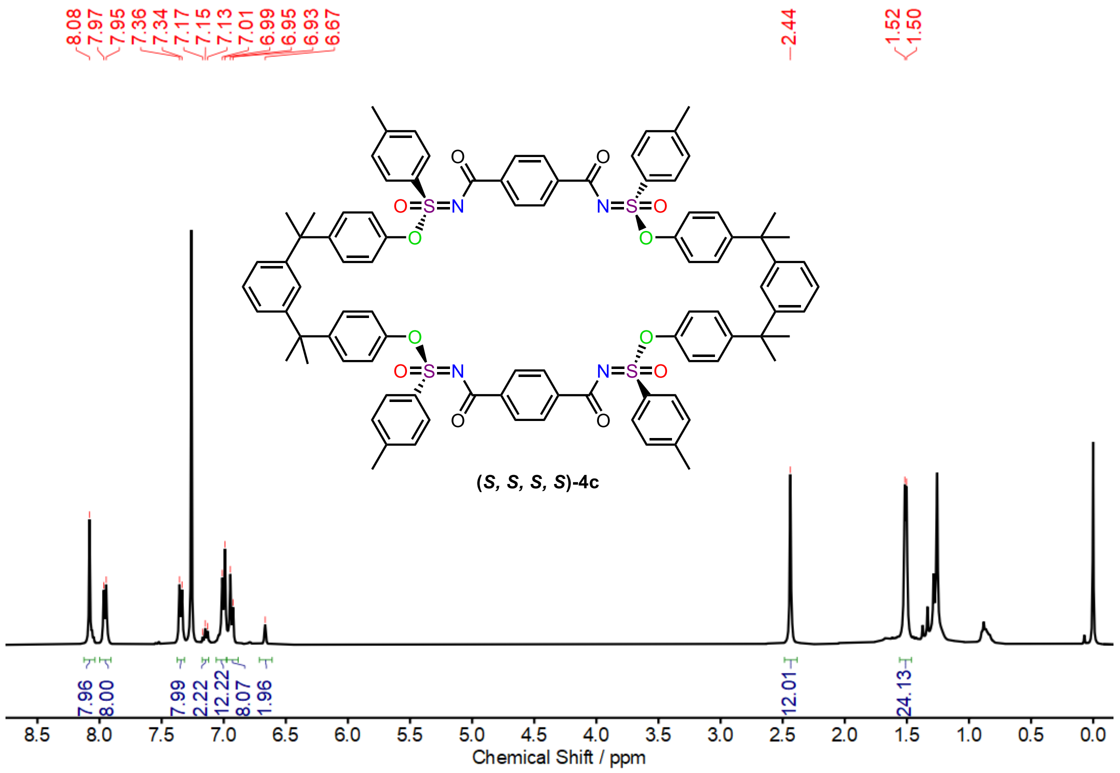

Figure S114. ^{1}H NMR (400 MHz) spectra of compound **(*S, S, S, S*)-4c** ($CDCl_3$, 298 K).

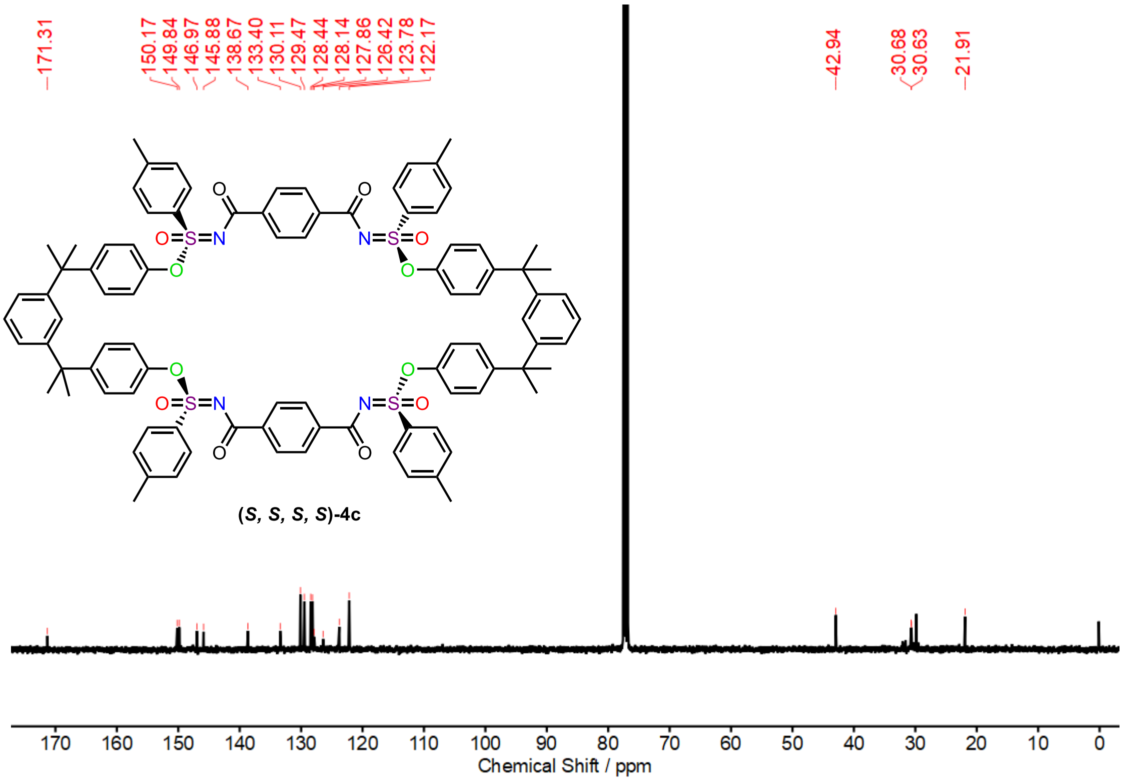

Figure S115. ^{13}C{1H} NMR (101 MHz) spectra of compound **(*S, S, S, S*)-4c** ($CDCl_3$, 298 K).

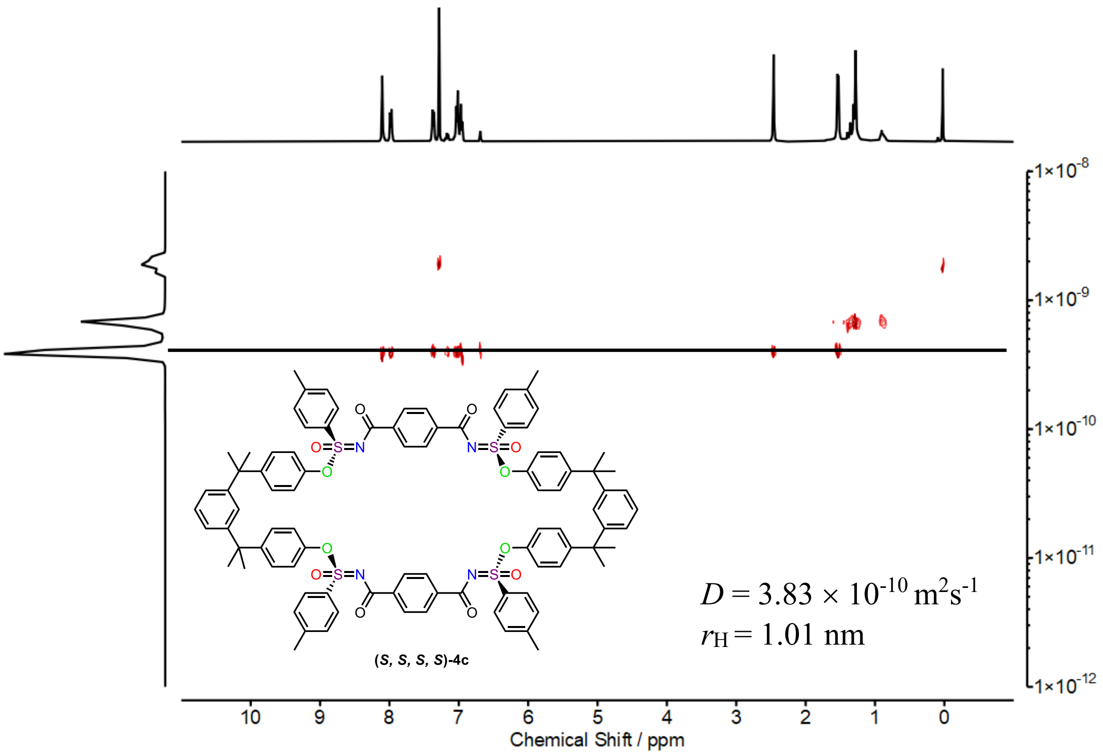

Figure S116. DOSY (400 MHz) spectra of compound ***(S, S, S, S)*-4c** (CDCl₃, 298 K).

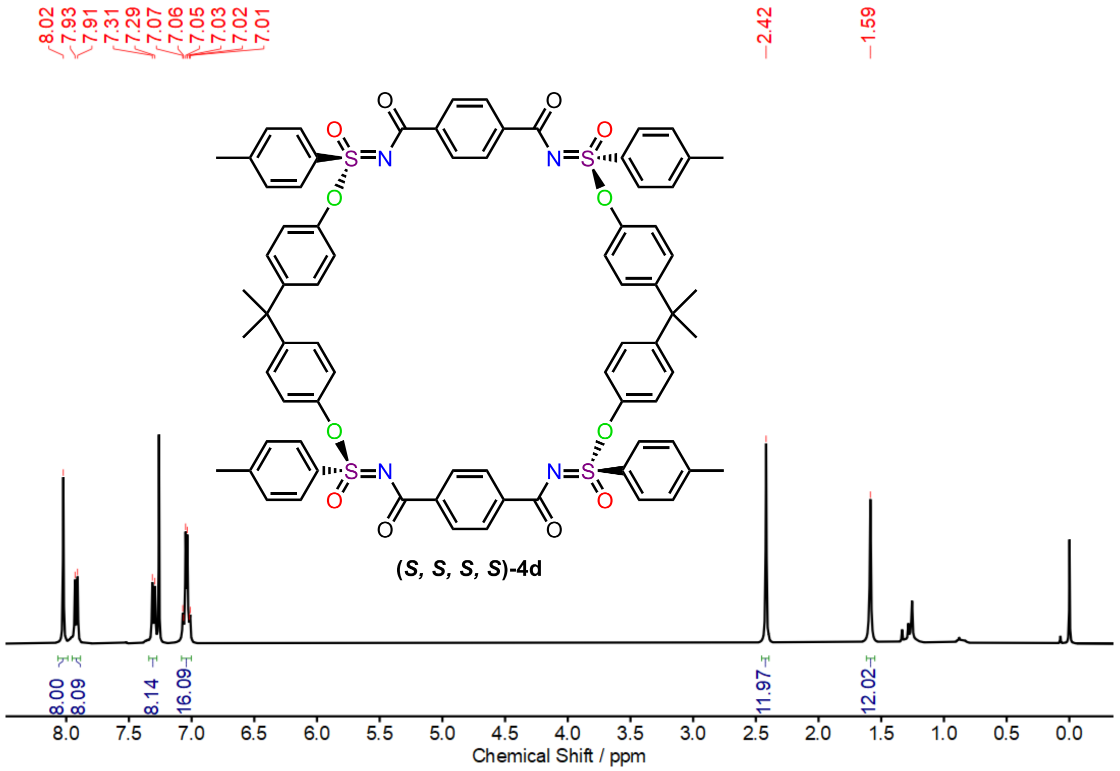

Figure S117. ^{1}H NMR (400 MHz) spectra of compound ***(S, S, S, S)*-4d** ($CDCl_3$, 298 K).

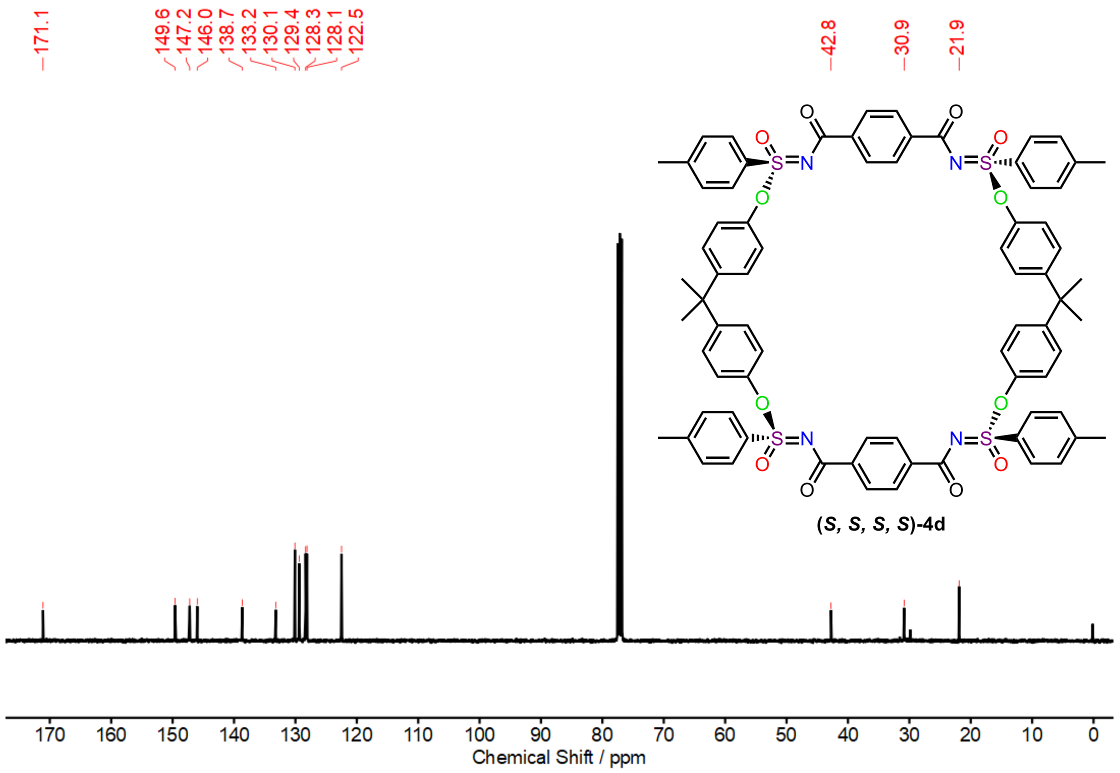

Figure S118. $^{13}C\{1H\}$ NMR (101 MHz) spectra of compound ***(S, S, S, S)*-4d** ($CDCl_3$, 298 K).

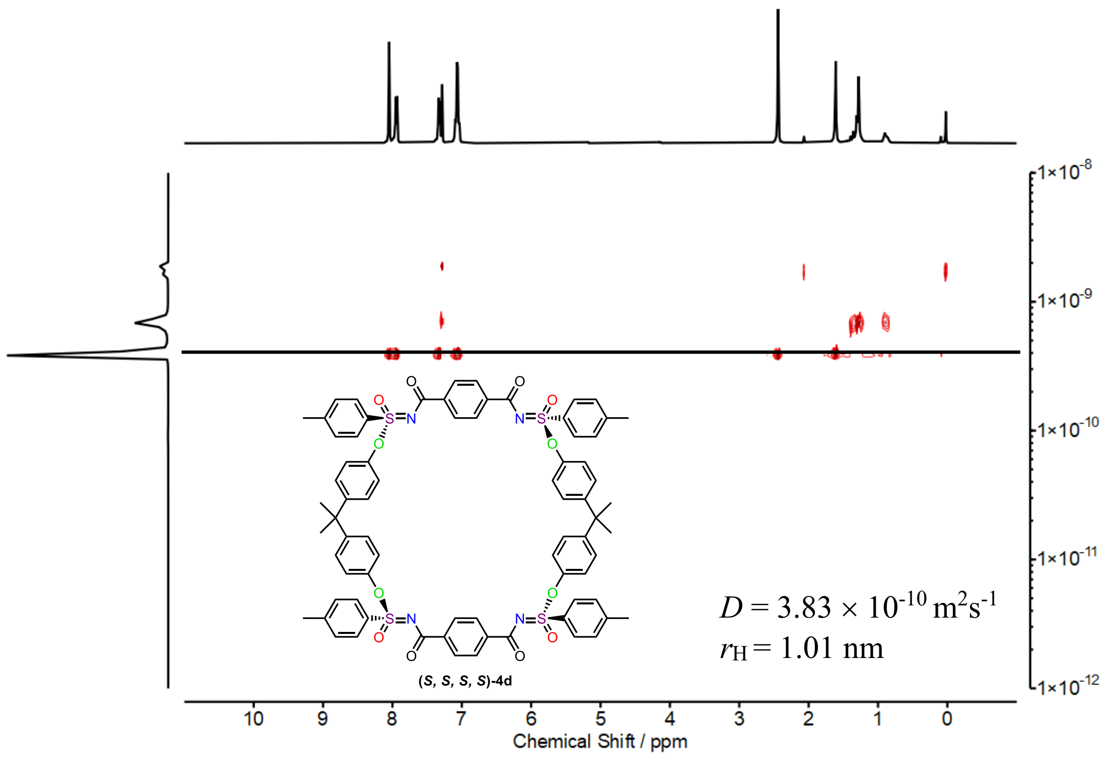

Figure S119. DOSY (400 MHz) spectra of compound ***(S, S, S, S)*-4d** ($CDCl_3$, 298 K).

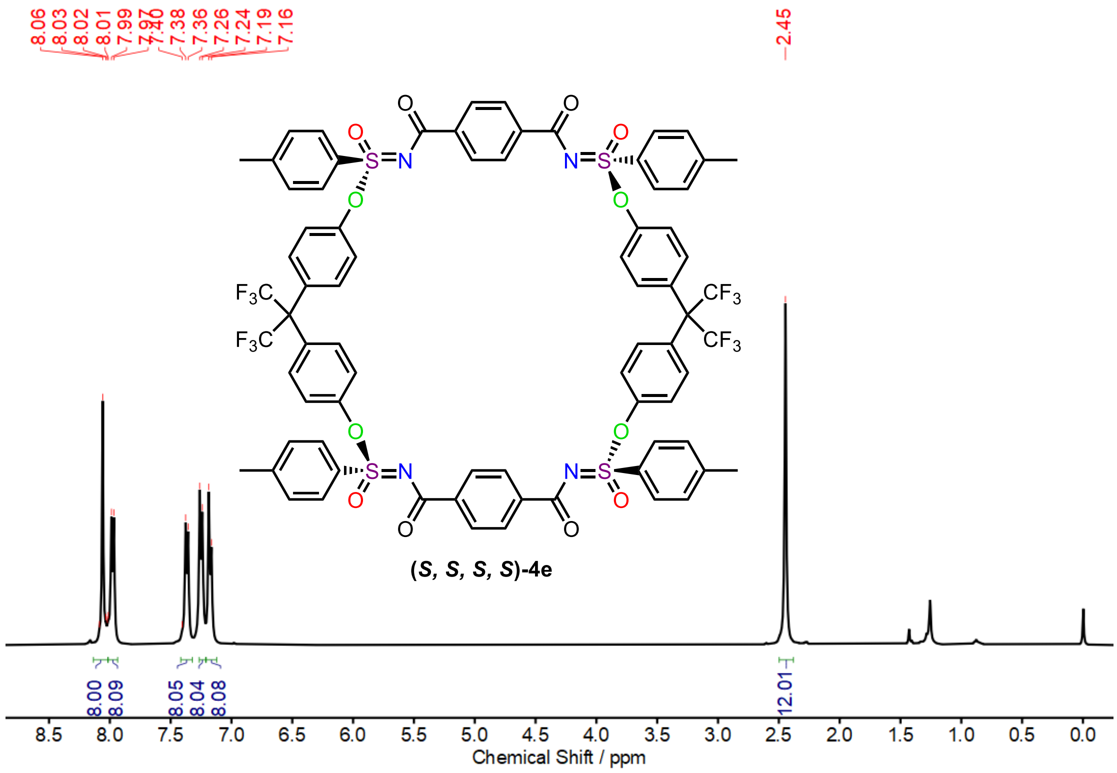

Figure S120. ^{1}H NMR (400 MHz) spectra of compound ***(S, S, S, S)*-4e** ($CDCl_3$, 298 K).

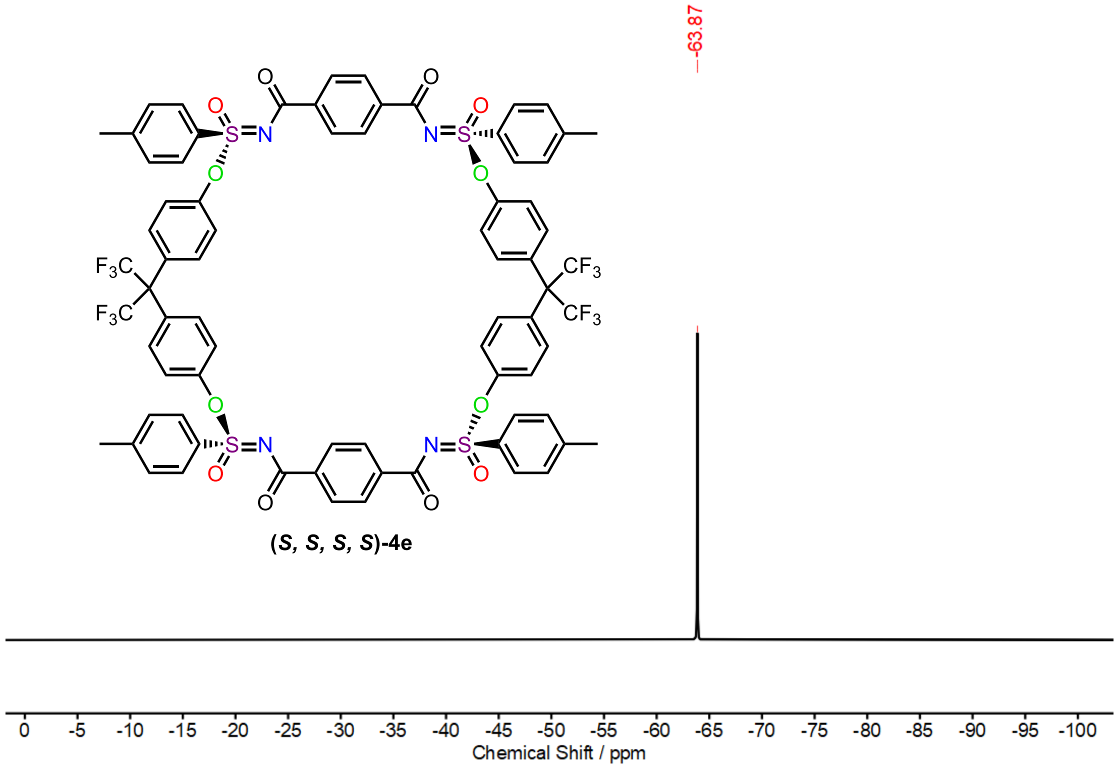

Figure S121. ^{19}F NMR (376 MHz) spectra of compound ***(S, S, S, S)*-4e** ($CDCl_3$, 298 K).

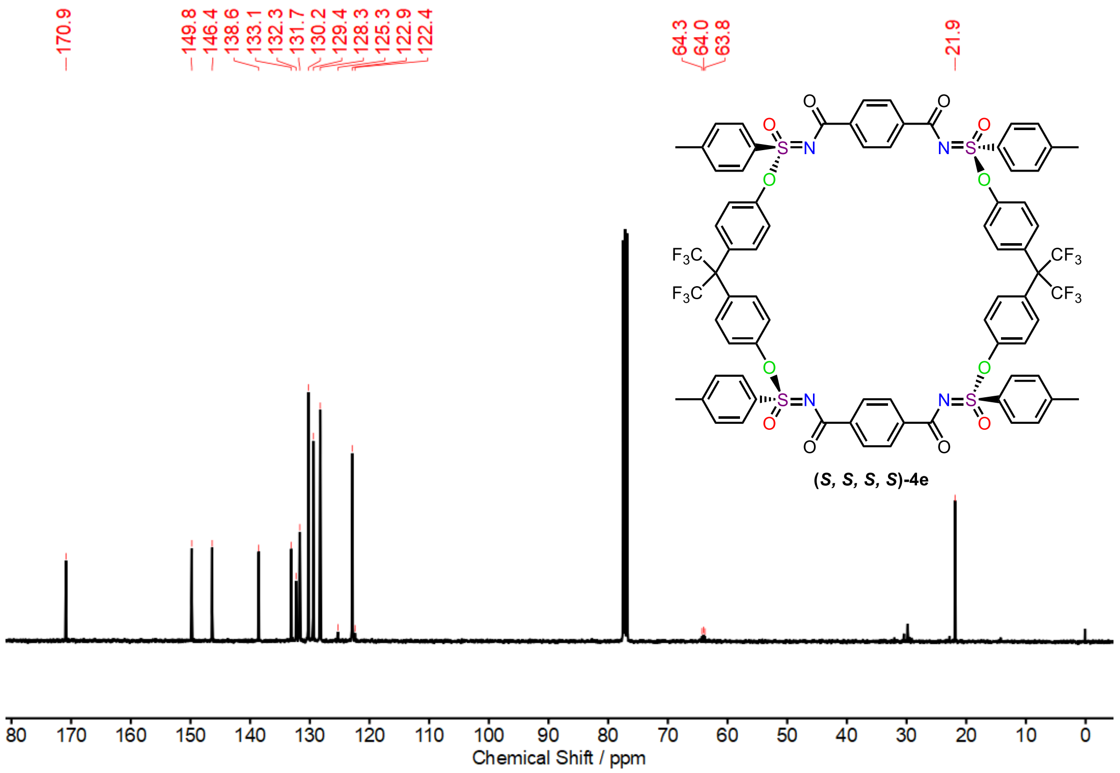

Figure S122. $^{13}C\{1H\}$ NMR (101 MHz) spectra of compound **(*S*, *S*, *S*, *S*)-4e** ($CDCl_3$, 298 K).

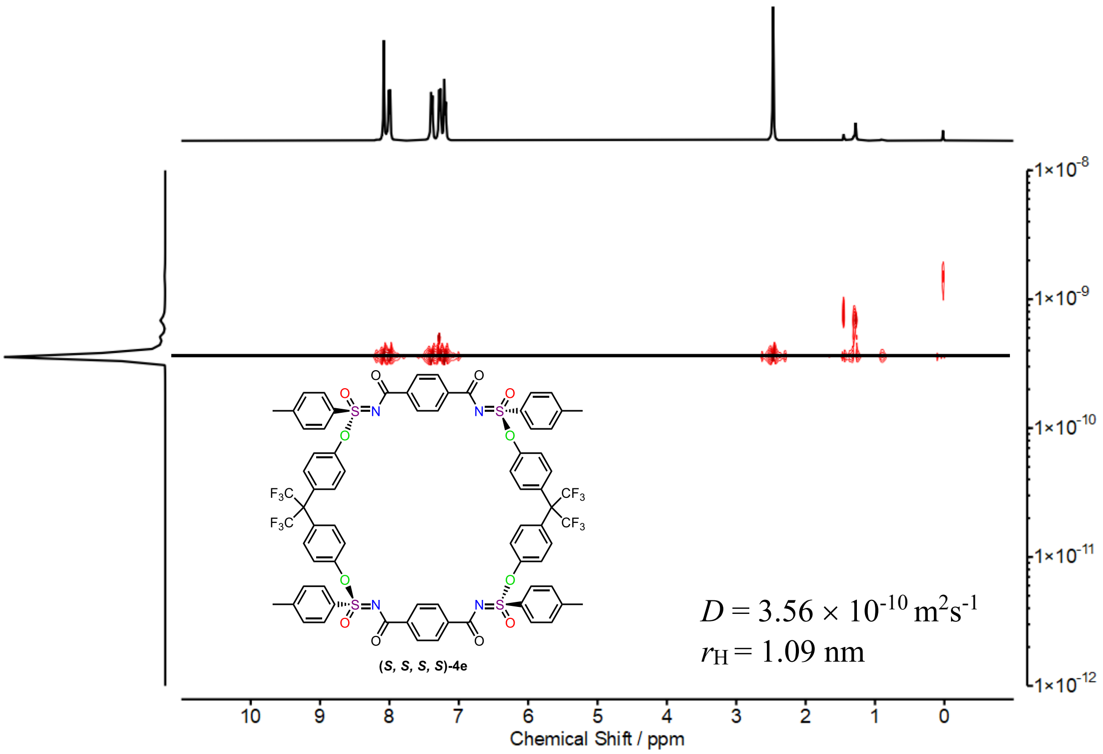

Figure S123. DOSY (400 MHz) spectra of compound **(*S*, *S*, *S*, *S*)-4e** ($CDCl_3$, 298 K).

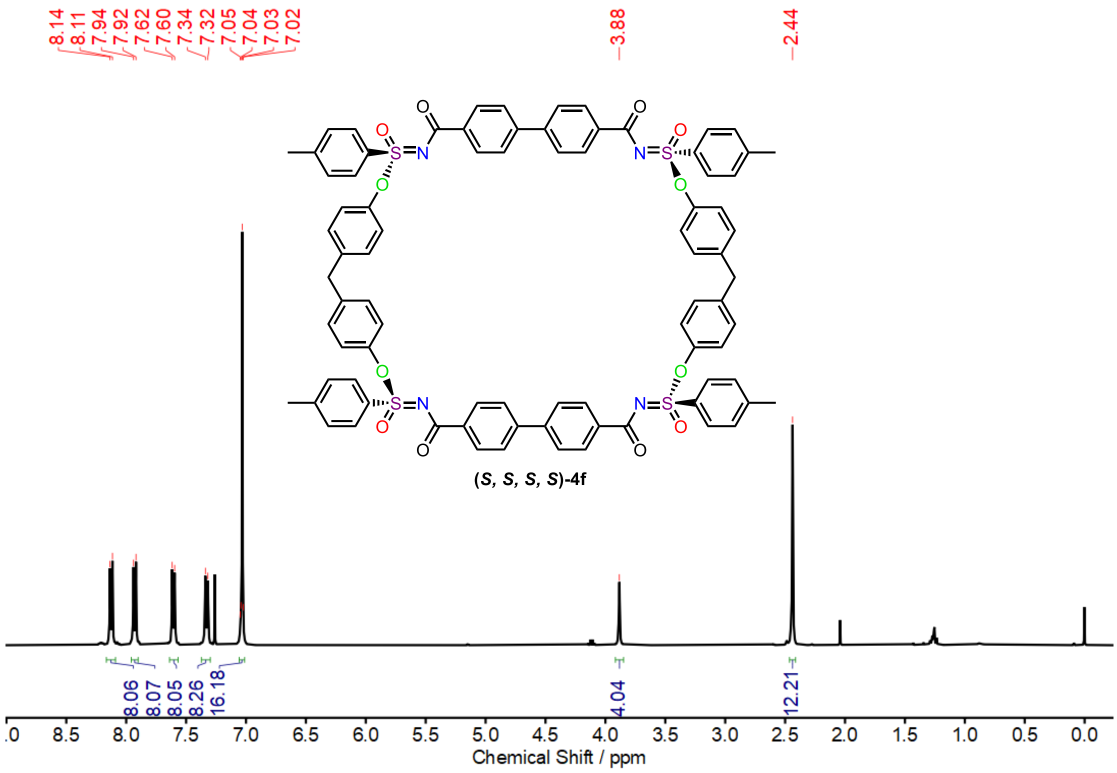

Figure S124. ^{1}H NMR (400 MHz) spectra of compound ***(S, S, S, S)*-4f** (CDCl3, 298 K).

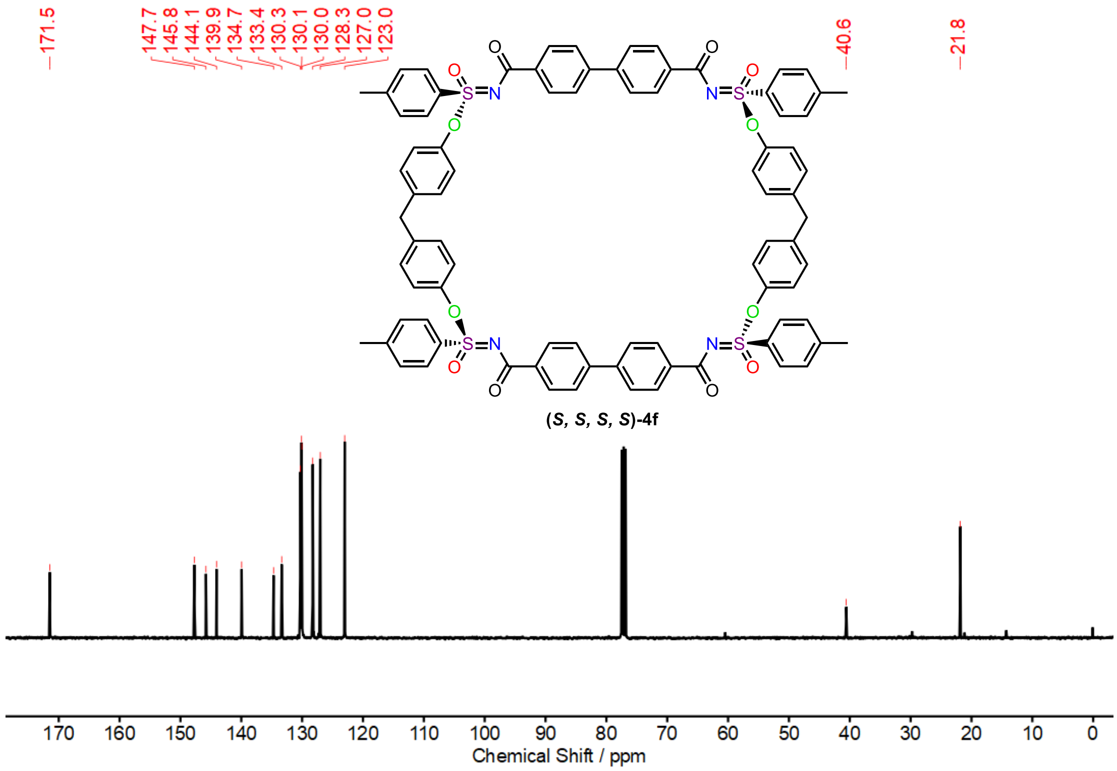

Figure S125. ^{13}C{1H} NMR (101 MHz) spectra of compound ***(S, S, S, S)*-4f** (CDCl3, 298 K).

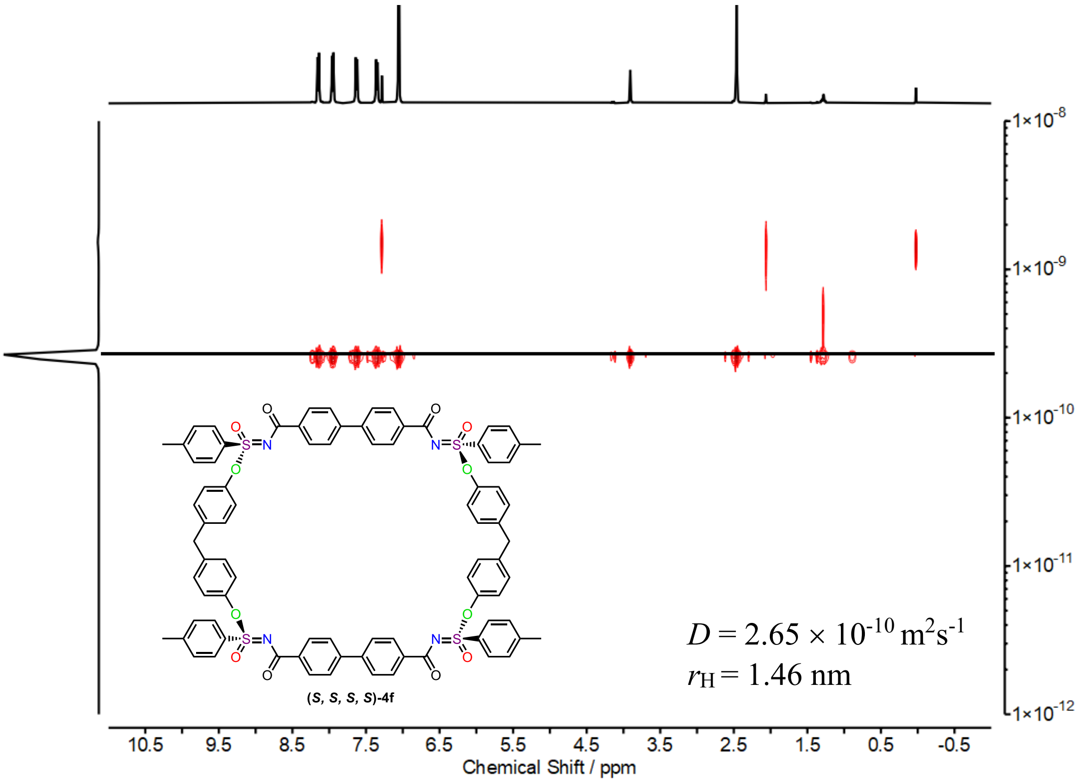

Figure S126. DOSY (400 MHz) spectra of compound ***(S, S, S, S)*-4f** ($CDCl_3$, 298 K).

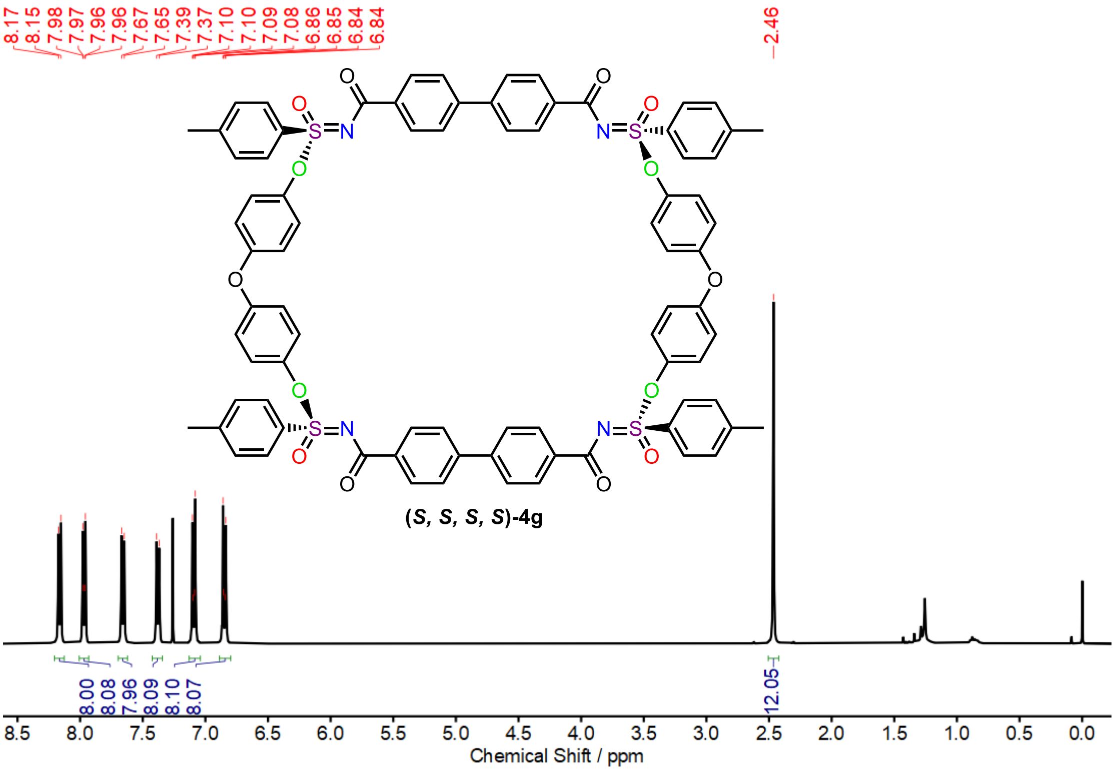

Figure S127. ^{1}H NMR (400 MHz) spectra of compound ***(S, S, S, S)*-4g** ($CDCl_3$, 298 K).

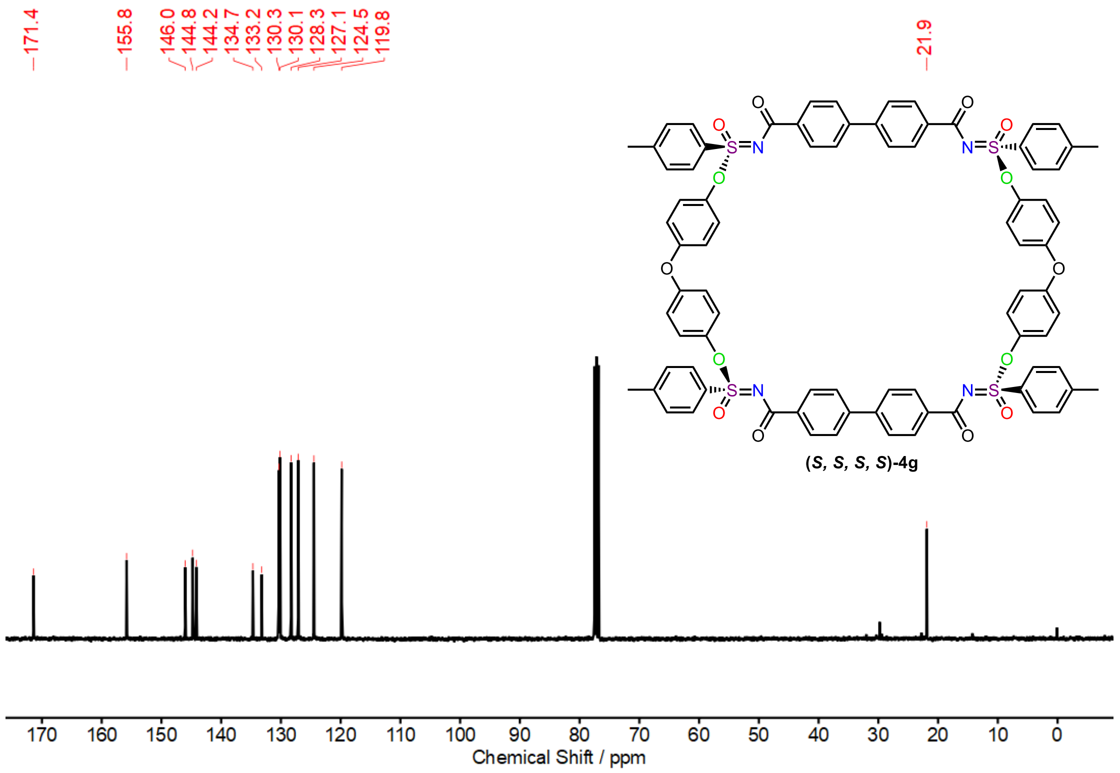

Figure S128. $^{13}C\{1H\}$ NMR (101 MHz) spectra of compound **(*S*, *S*, *S*, *S*)-4g** ($CDCl_3$, 298 K).

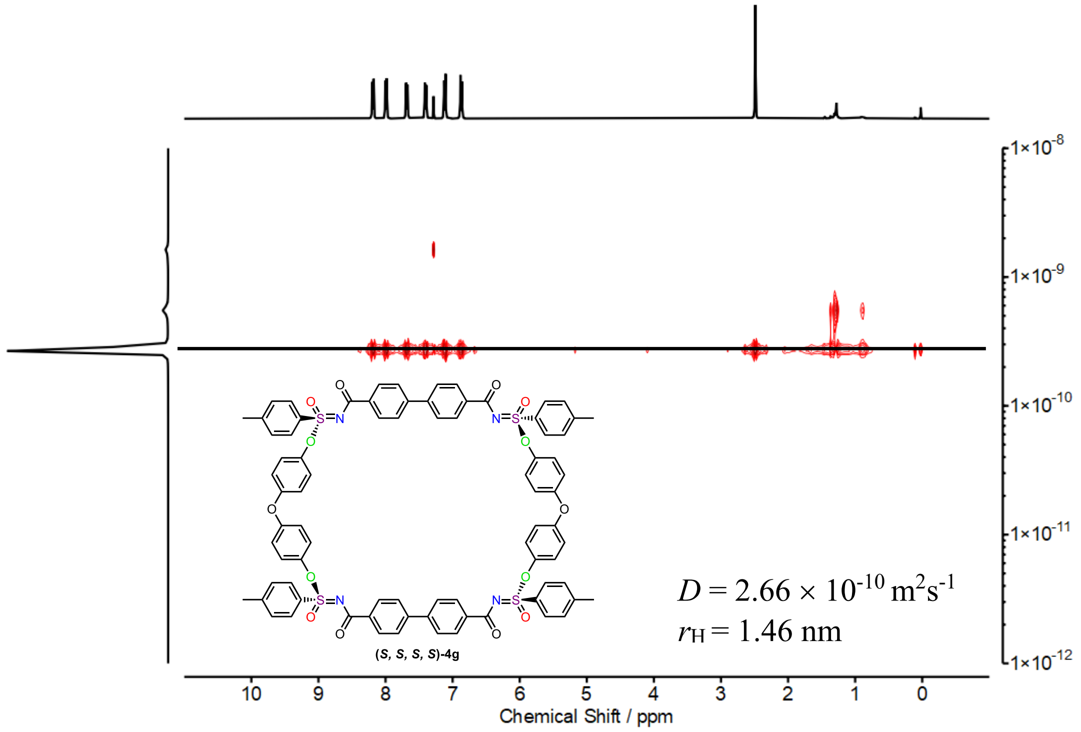

Figure S129. DOSY (400 MHz) spectra of compound **(*S*, *S*, *S*, *S*)-4g** ($CDCl_3$, 298 K).

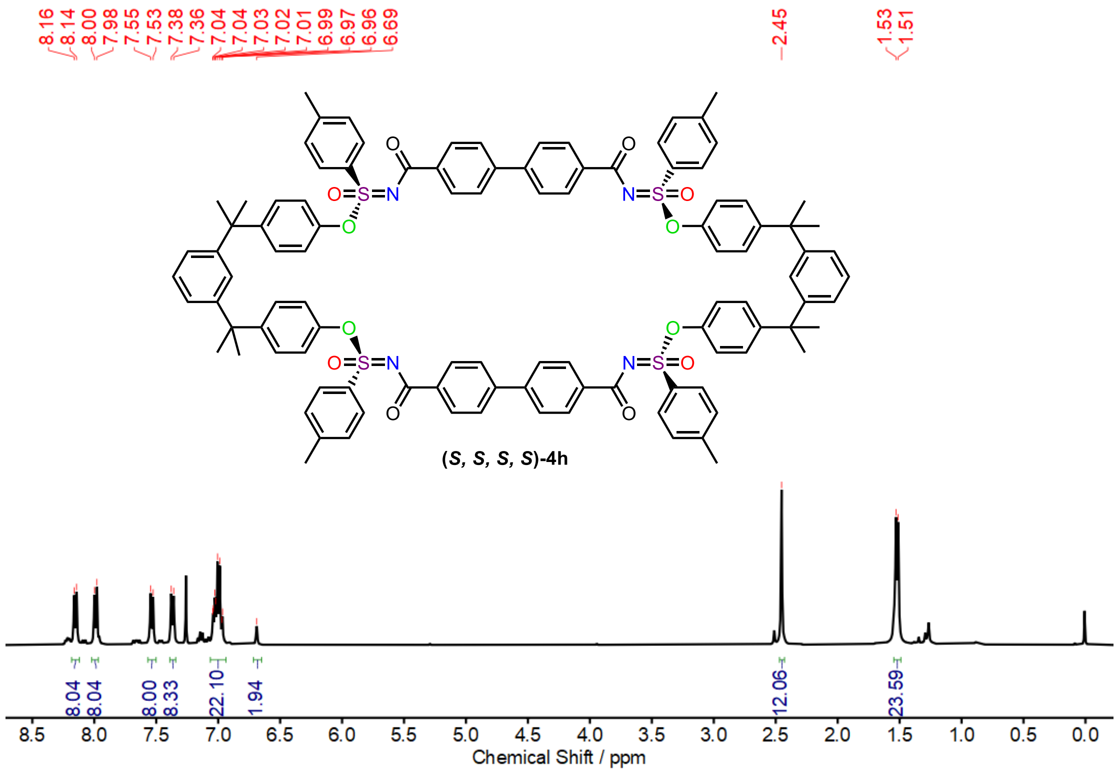

Figure S130. ^{1}H NMR (400 MHz) spectra of compound ***(S, S, S, S)*-4h** ($CDCl_3$, 298 K).

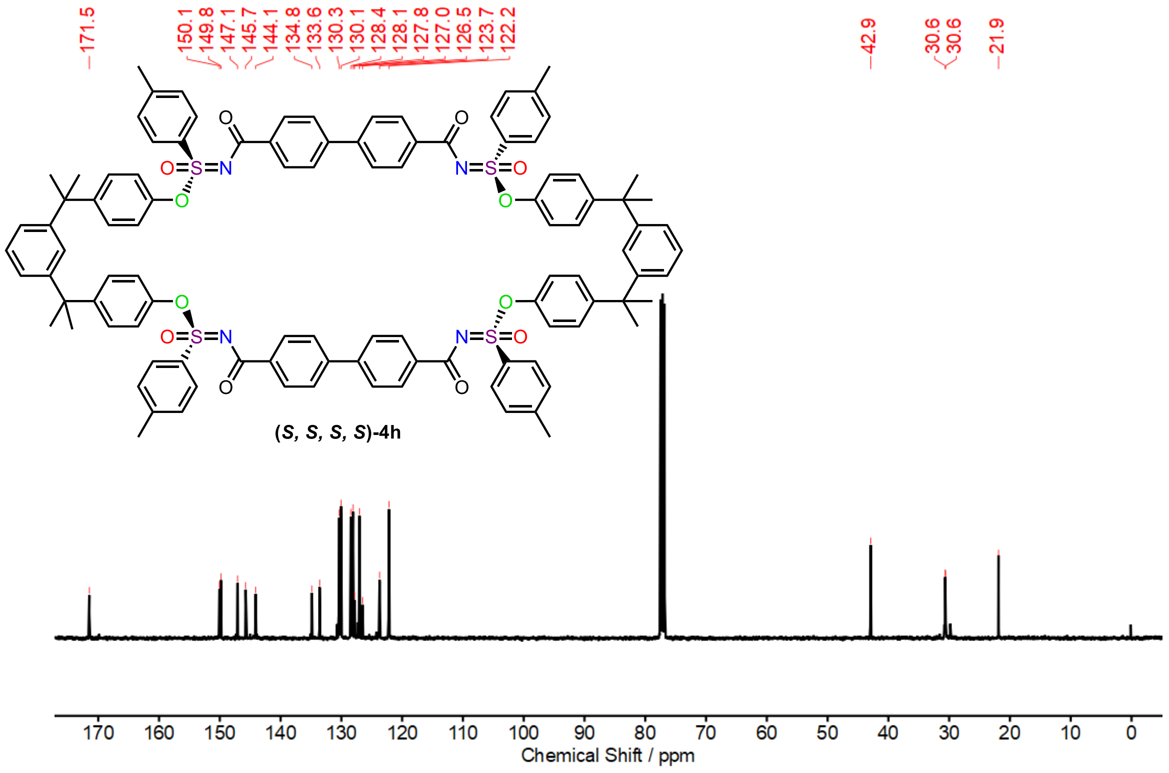

Figure S131. ^{13}C{1H} NMR (101 MHz) spectra of compound ***(S, S, S, S)*-4h** ($CDCl_3$, 298 K).

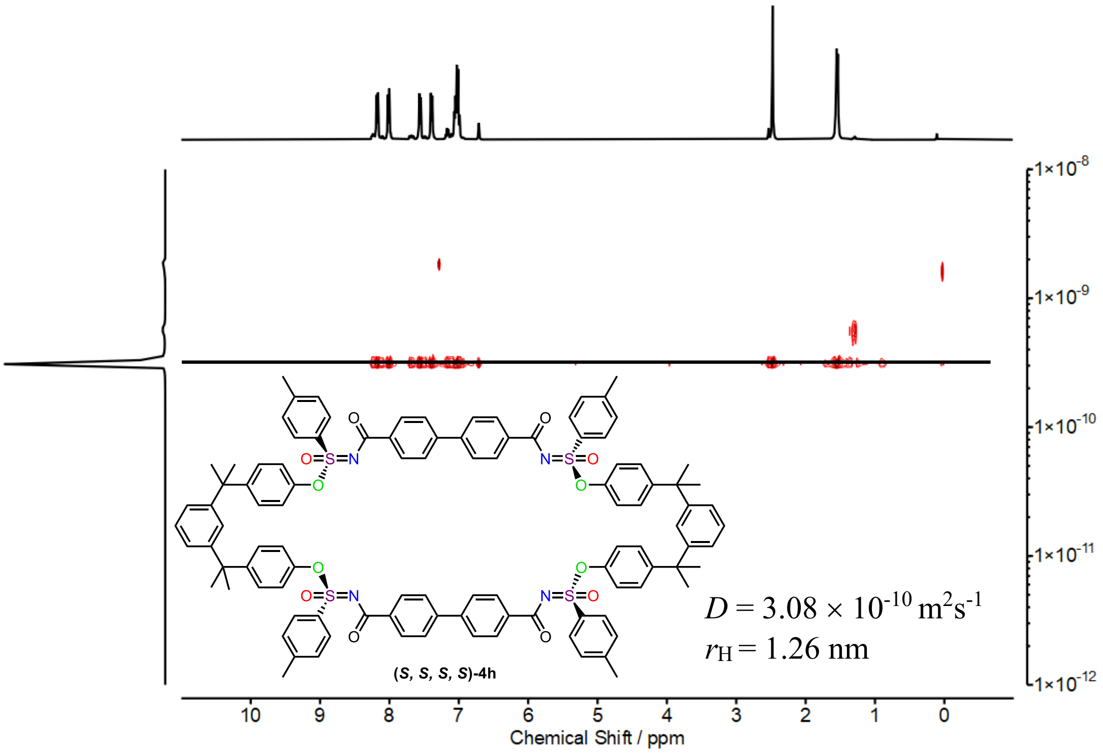

Figure S132. DOSY (400 MHz) spectra of compound ***(S, S, S, S)*-4h** ($CDCl_3$, 298 K).

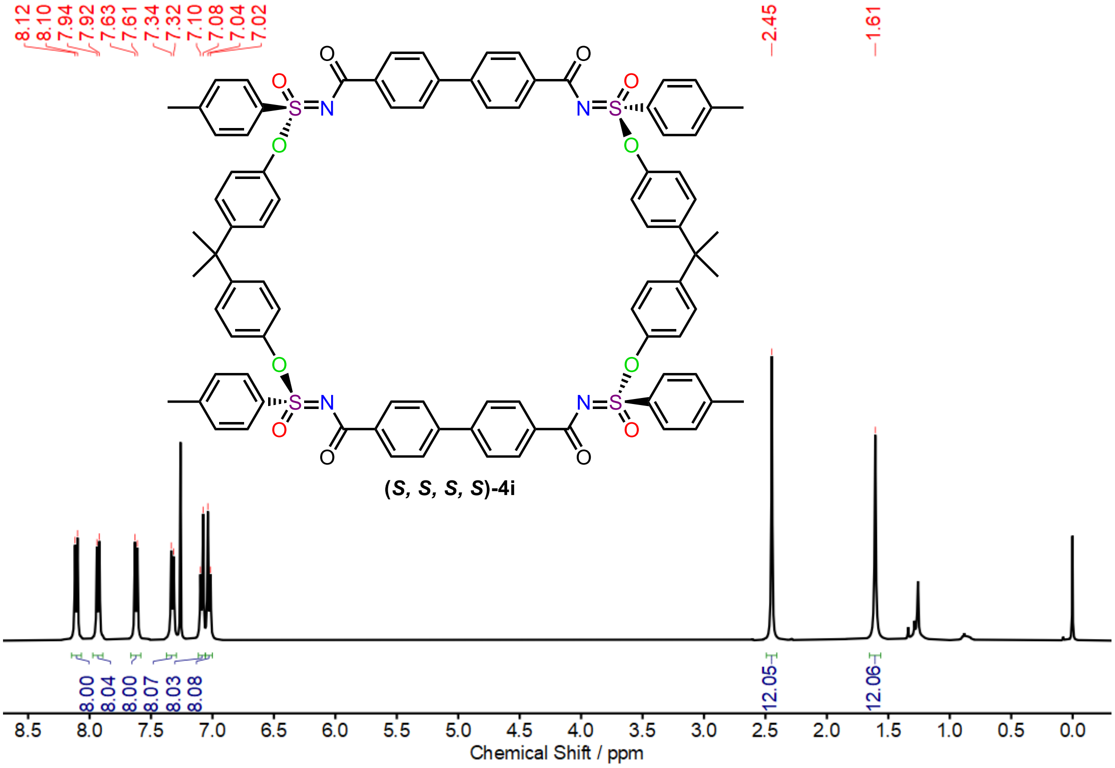

Figure S133. ^{1}H NMR (400 MHz) spectra of compound ***(S, S, S, S)*-4i** ($CDCl_3$, 298 K).

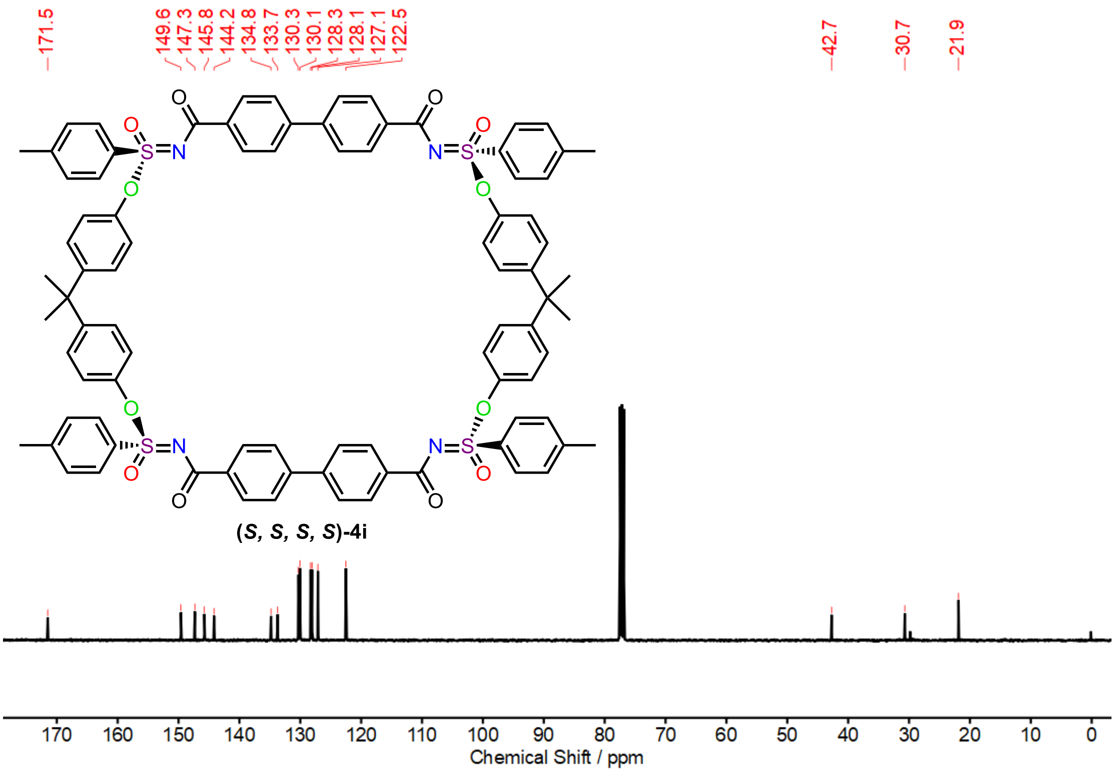

Figure S134. $^{13}C\{1H\}$ NMR (101 MHz) spectra of compound ***(S, S, S, S)*-4i** ($CDCl_3$, 298 K).

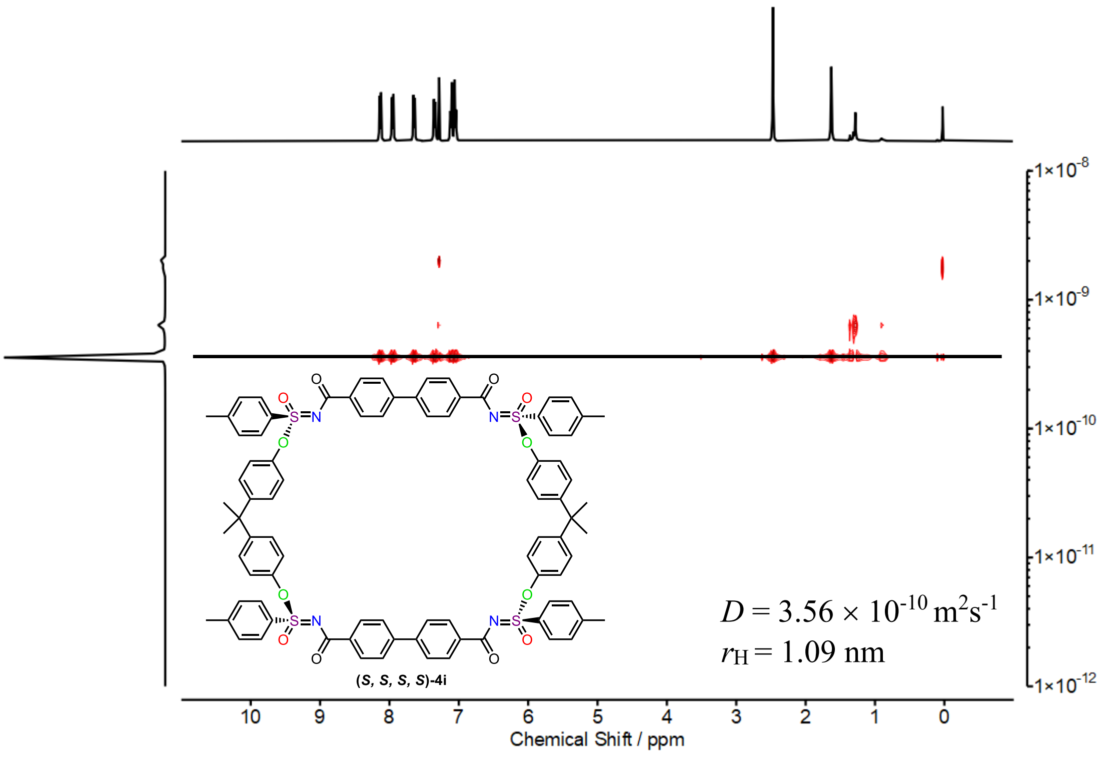

Figure S135. DOSY (400 MHz) spectra of compound ***(S, S, S, S)*-4i** ($CDCl_3$, 298 K).

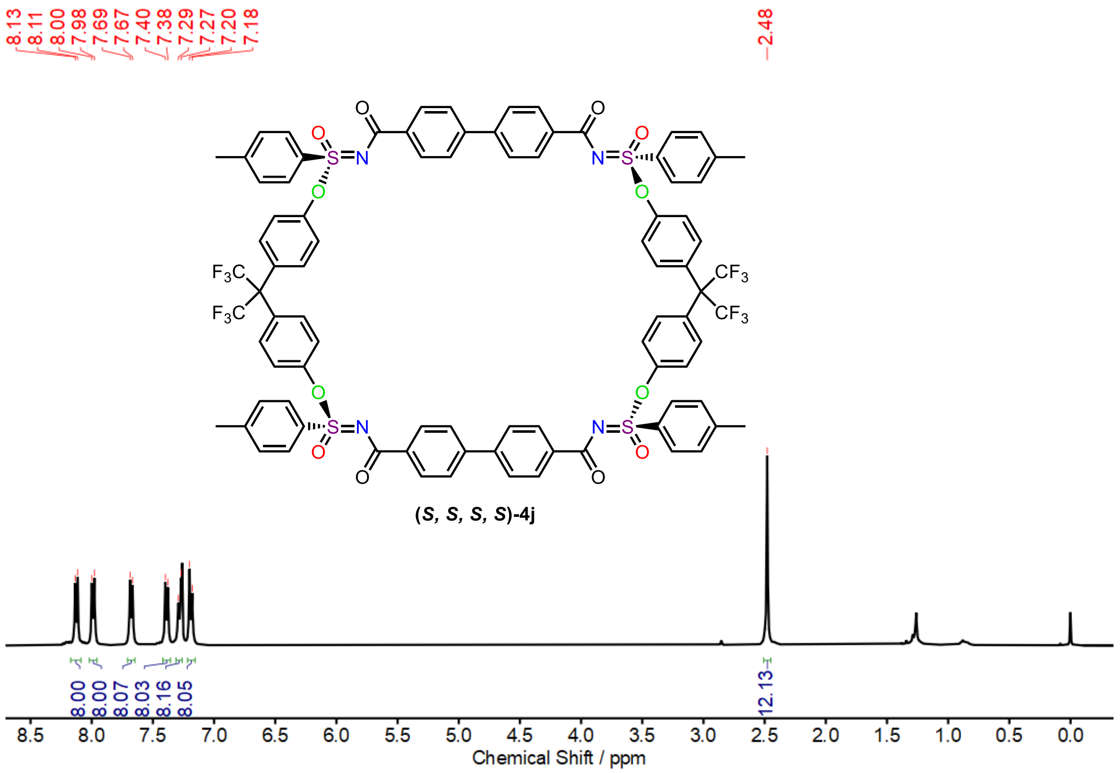

Figure S136. ^{1}H NMR (400 MHz) spectra of compound ***(S, S, S, S)*-4j** ($CDCl_3$, 298 K).

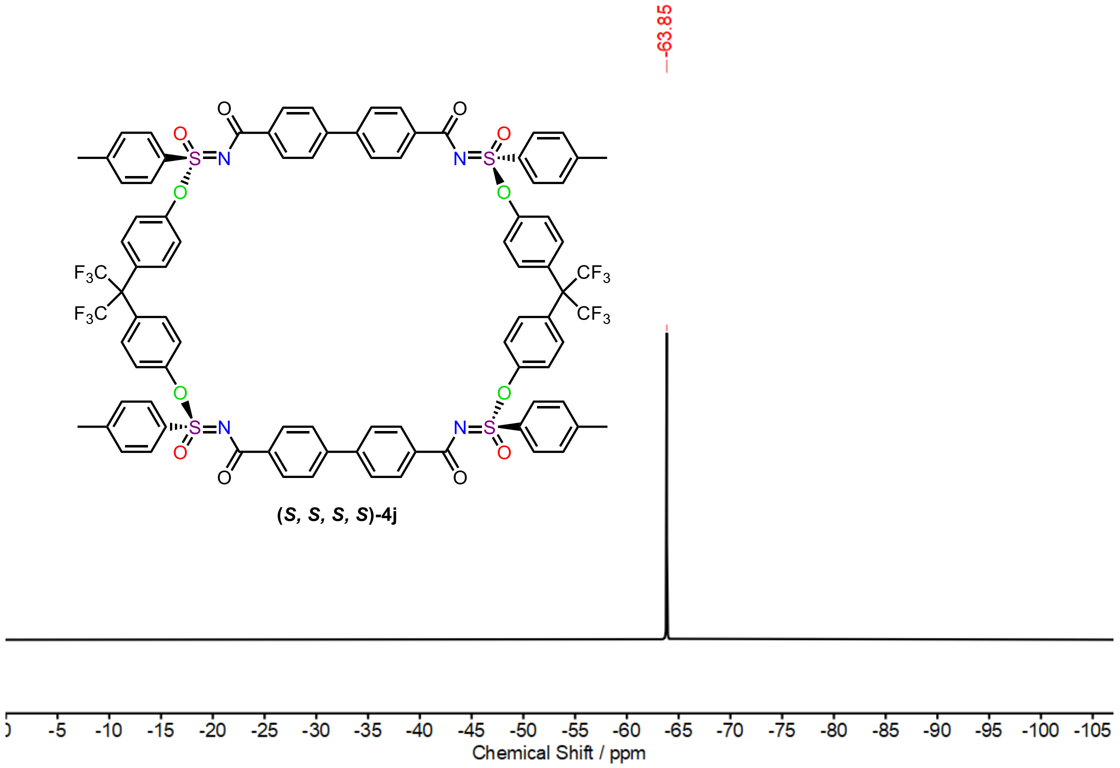

Figure S137. ^{19}F NMR (376 MHz) spectra of compound ***(S, S, S, S)*-4j** ($CDCl_3$, 298 K).

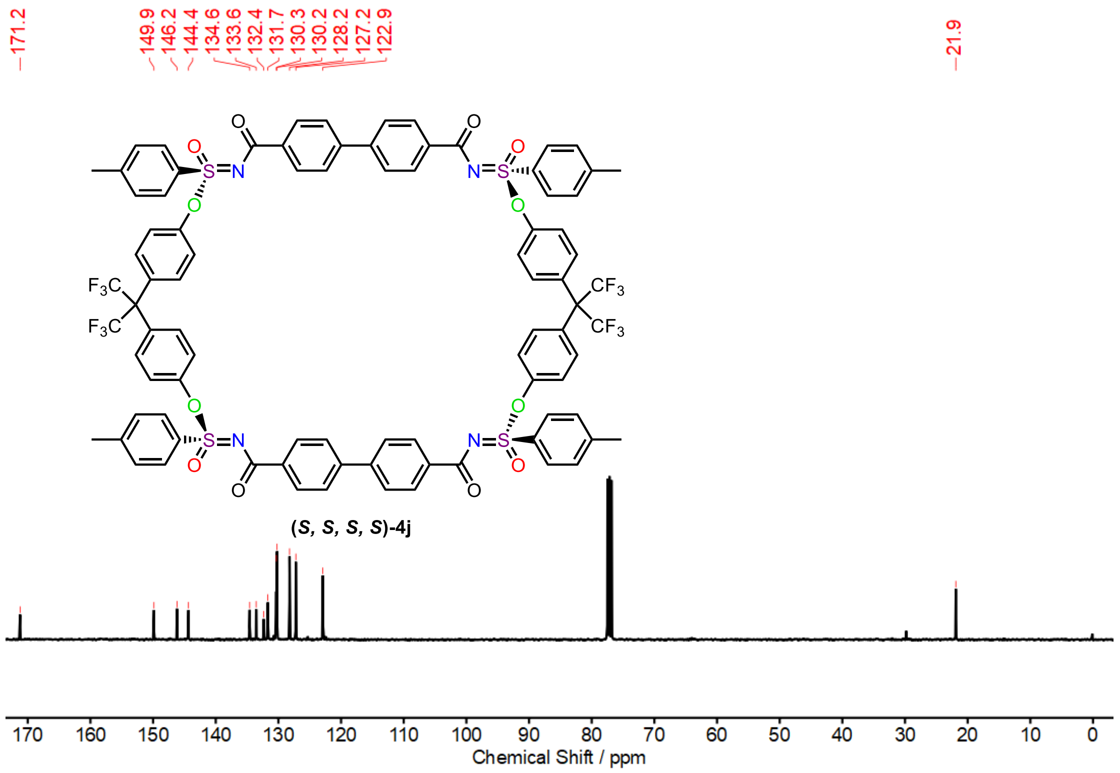

Figure S138. $^{13}C\{1H\}$ NMR (101 MHz) spectra of compound ***(S, S, S, S)*-4j** ($CDCl_3$, 298 K).

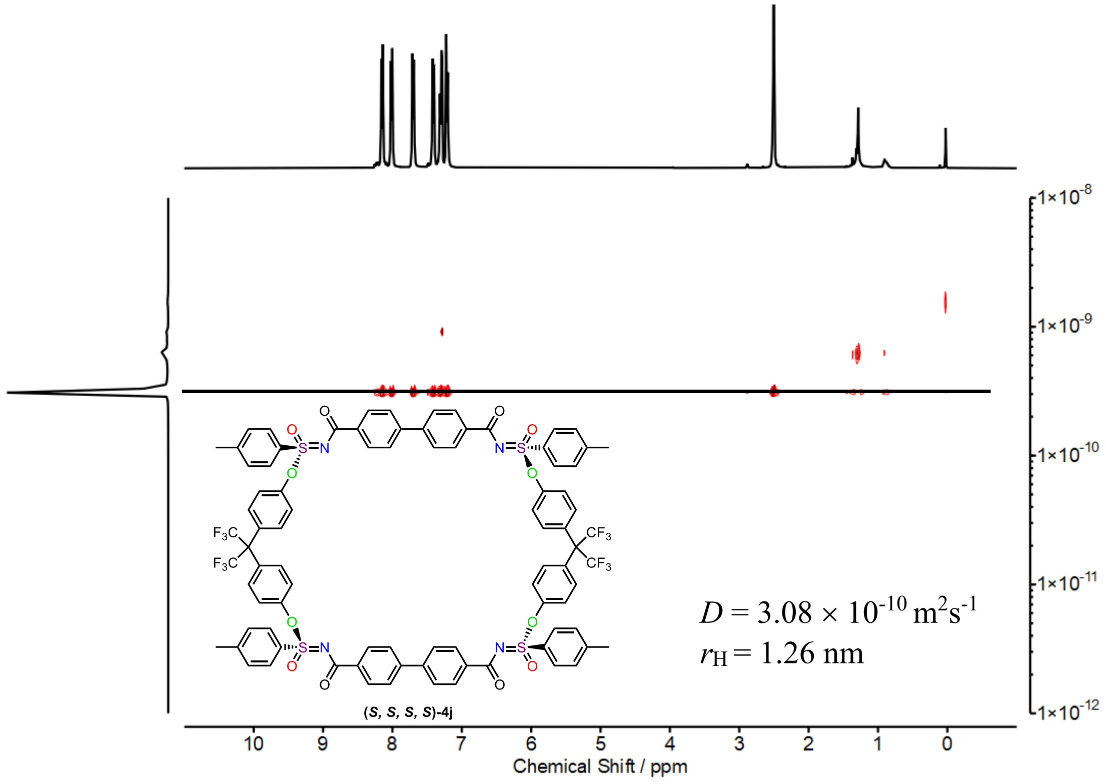

Figure S139. DOSY (400 MHz) spectra of compound ***(S, S, S, S)*-4j** ($CDCl_3$, 298 K).

4. HPLC data

a)

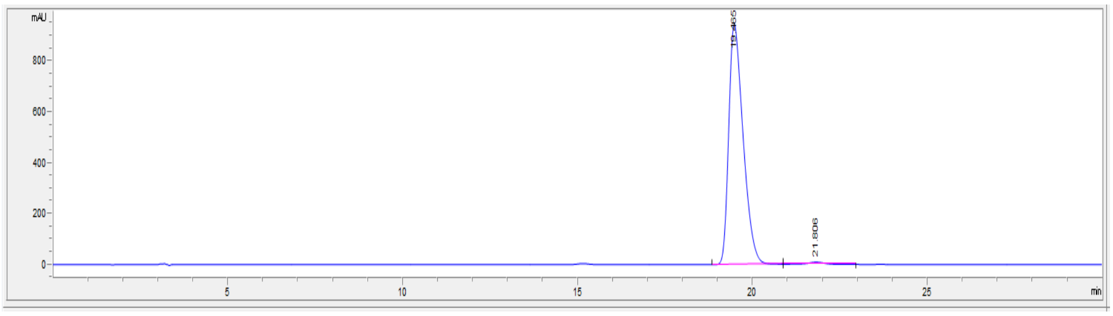

#	Time	Type	Area	Height	Width	Area%
1	19.465	BB	27132.5	948.8	0.4356	98.993
2	21.806	BB	276	8.1	0.5129	1.007

b)

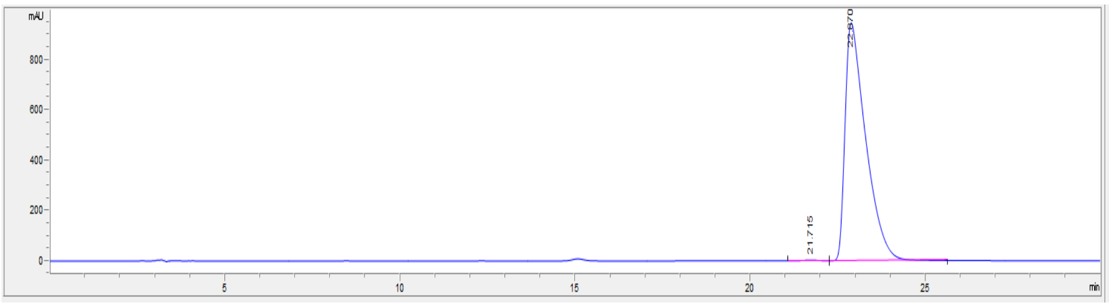

#	Time	Type	Area	Height	Width	Area%
1	21.715	BB	87.2	3	0.4347	0.224
2	22.87	BB	38867.6	948.8	0.6019	99.776

c)

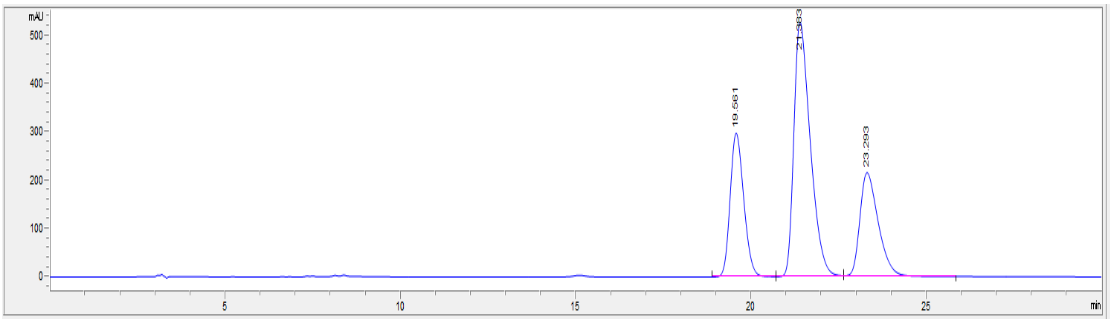

#	Time	Type	Area	Height	Width	Area%
1	19.561	BB	7987.7	296.8	0.416	24.236
2	21.383	BV	17122.4	525.7	0.494	51.951
3	23.293	VB	7848.5	214.7	0.5566	23.813

Figure S140: HPLC chromatograms of a) **(*R*, *R*)-1a, b) (*S*, *S*)-1a** and c) **a mixture of diasteromers-1a** with optimized eluting protocol (CHIRALPAK® IE; *n*-Hexane/DCM/*i*-PrOH 70/20/10; flow rate: 1.0 mL/min; UV detector wavelength 254 nm; oven temperature 40 °C; inject volume 5.0 μL).

a)

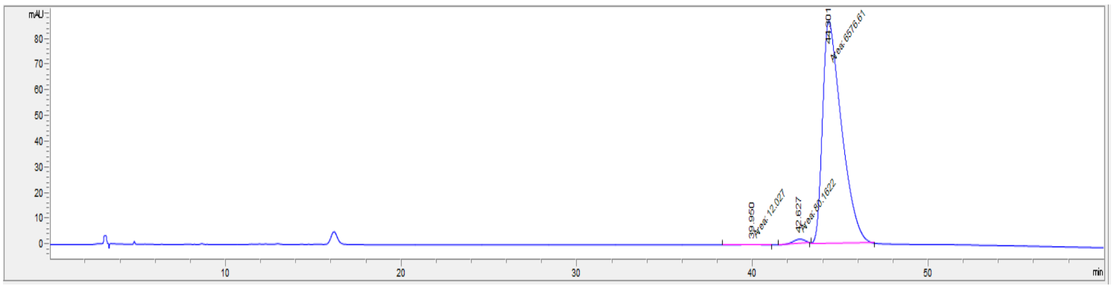

#	Time	Type	Area	Height	Width	Area%
1	39.95	MM	12	1.7E-1	1.1863	0.180
2	42.627	MP	80.2	1.7	0.7775	1.202
3	44.301	MM	6576.6	87.2	1.2565	98.618

b)

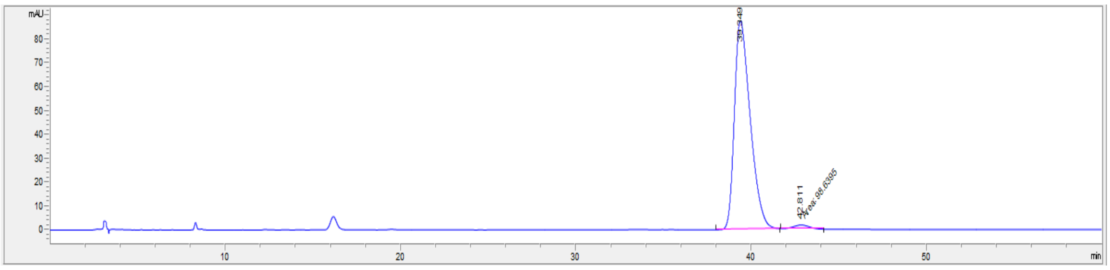

#	Time	Type	Area	Height	Width	Area%
1	39.349	BB	5514	88.1	0.9421	98.243
2	42.811	MP	98.6	1.6	1.0094	1.757

c)

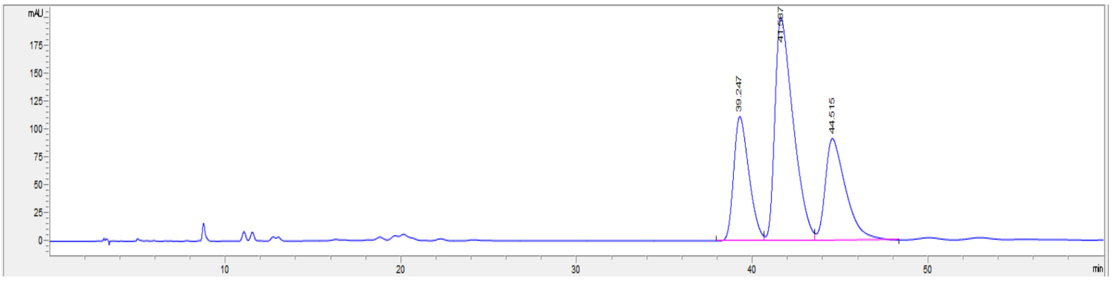

#	Time	Type	Area	Height	Width	Area%
1	39.247	BV	6665.8	110.5	0.9277	23.492
2	41.587	VV	14388.9	198.6	1.0654	50.710
3	44.515	VB	7320.1	90.6	1.1787	25.798

Figure S141: HPLC chromatograms of a) **(*R*, *R*)-1b,** b) **(*S*, *S*)-1b** and c) **a mixture of diasteromers-1b** with optimized eluting protocol (CHIRALPAK® IE; *n*-Hexane/DCM/*i*-PrOH 70/20/10; flow rate: 1.0 mL/min; UV detector wavelength 254 nm; oven temperature 40 °C; inject volume 5.0 μL).

a)

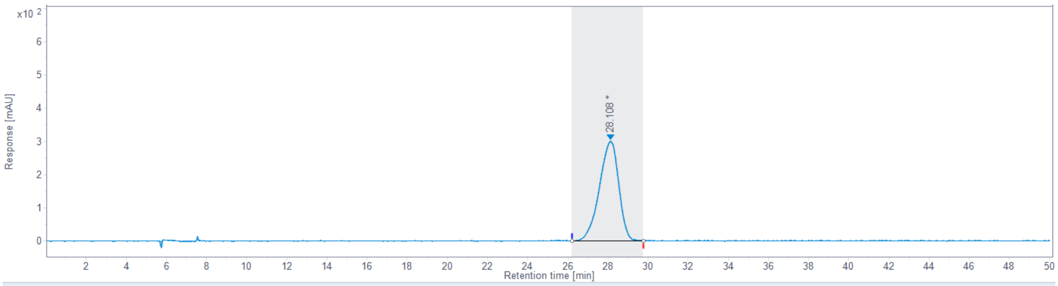

Injection Results

Peaks | Summary

#	Name	Signal description	RT (min)	Area (mAU·s)	Area%	Height (mAU)	Height%	Amount	Concentration	Start time (min)	End time (min)
1		DAD1D,Sig=280,4 Ref=off	28.108	20112.299	100.000	301.705	100.00			26.197	29.785

b)

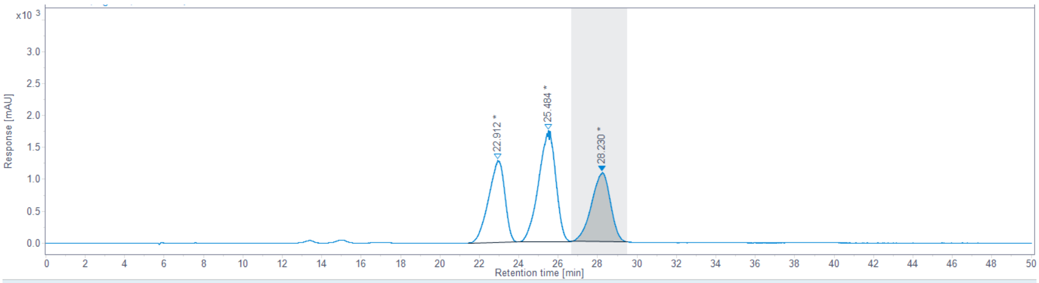

Injection Results

Peaks | Summary

#	Name	Signal description	RT (min)	Area (mAU·s)	Area%	Height (mAU)	Height%	Amount	Concentration	Start time (min)	End time (min)
1		DAD1D,Sig=280,4 Ref=off	22.912	73327.186	29.294	1289.446	31.32			21.441	23.927
2		DAD1D,Sig=280,4 Ref=off	25.484	104861.502	41.893	1743.503	42.35			23.942	26.645
3		DAD1D,Sig=280,4 Ref=off	28.230	72122.042	28.813	1084.403	26.34			26.693	29.530

Figure S142: HPLC chromatograms of a) **(*R*, *R*)-1d**, and b) **a mixture of diasteromers-1d** with optimized eluting protocol (CHIRALPAK® IA; *n*-Hexane/DCM/MeOH 80/20/5; flow rate: 0.5 mL/min; UV detector wavelength 280 nm; inject volume 5.0 µL).

a)

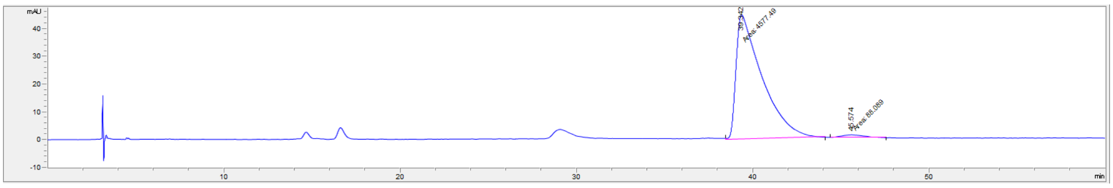

#	Time	Type	Area	Height	Width	Area%
1	39.342	MM	4577.5	44.7	1.7072	98.112
2	45.574	MM	88.1	9.7E-1	1.5079	1.888

b)

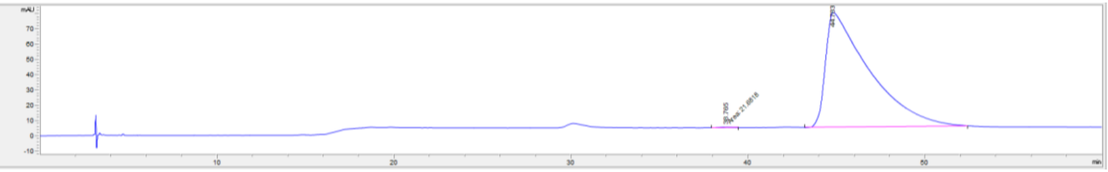

#	Time	Type	Area	Height	Width	Area%
1	38.765	MM	21.7	4.1E-1	0.8834	0.168
2	44.783	BB	12881.6	74.9	2.212	99.832

c)

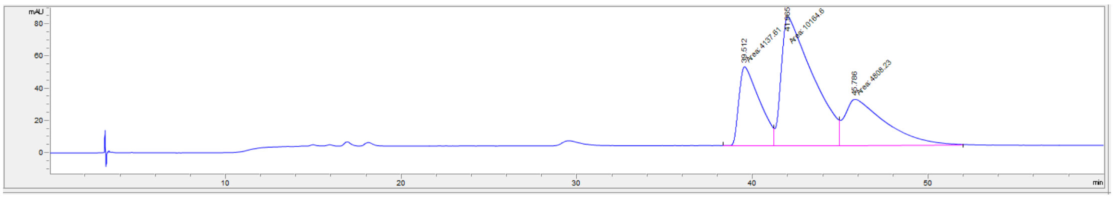

#	Time	Type	Area	Height	Width	Area%
1	39.512	MF	4137.6	49.7	1.3863	21.651
2	41.965	MF	10164.6	81.1	2.0886	53.189
3	45.786	FM	4808.2	29.2	2.7459	25.160

Figure S143: HPLC chromatograms of a) **(*R*, *R*)-1e,** b) **(*S*, *S*)-1e** and c) **a mixture of diasteromers -1e** with optimized eluting protocol (CHIRALPAK® IE; *n*-Hexane/DCM 40/60; flow rate: 1.0 mL/min; UV detector wavelength 60 nm; oven temperature 40 °C; inject volume 5.0 µL).

a)

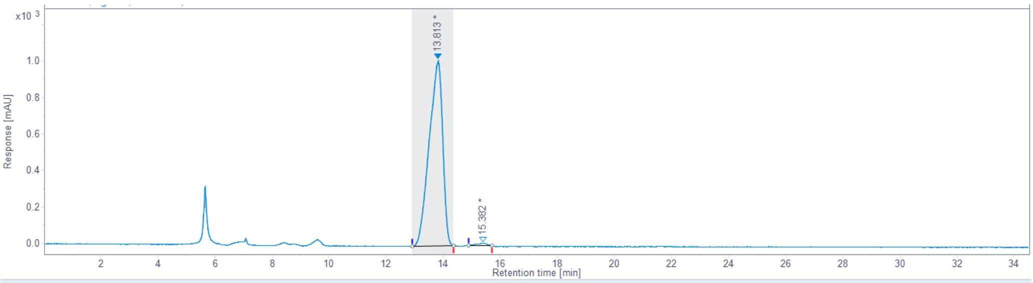

Injection Results

Peaks | Summary

#	Name	Signal description	RT (min)	Area (mAU·s)	Area%	Height (mAU)	Height%	Amount	Concentration	Start time (min)	End time (min)
1		DAD1B,Sig=240,4 Ref=off	13.813	33347.008	99.016	1016.995	98.67			12.899	14.366
2		DAD1B,Sig=240,4 Ref=off	15.382	331.545	0.984	13.688	1.33			14.883	15.709

b)

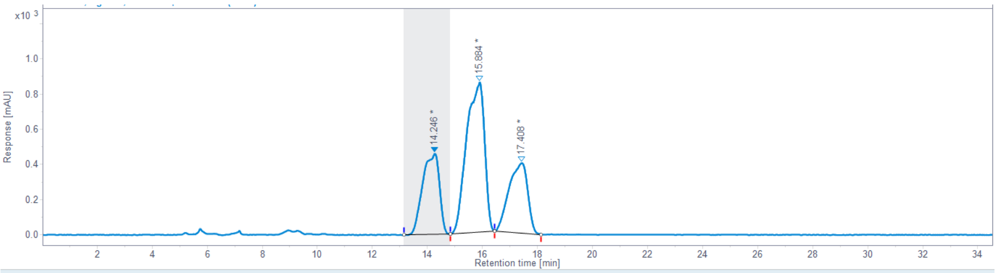

Injection Results

Peaks | Summary

#	Name	Signal description	RT (min)	Area (mAU·s)	Area%	Height (mAU)	Height%	Amount	Concentration	Start time (min)	End time (min)
1		DAD1B,Sig=240,4 Ref=off	14.246	19481.678	25.236	459.164	26.84			13.158	14.839
2		DAD1B,Sig=240,4 Ref=off	15.884	38856.498	50.334	852.381	49.82			14.839	16.430
3		DAD1B,Sig=240,4 Ref=off	17.408	18858.877	24.430	399.302	23.34			16.430	18.136

Figure S144: HPLC chromatograms of a) **(*S*, *S*)-3a**, and b) **a mixture of diasteromers-3a** with optimized eluting protocol (CHIRALPAK® IA; *n*-Hexane/DCM/MeOH 80/20/5; flow rate: 0.5 mL/min; UV detector wavelength 240 nm; inject volume 5.0 μL).

a)

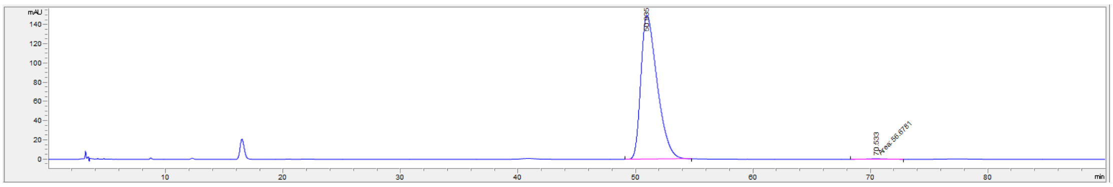

#	Time	Type	Area	Height	Width	Area%
1	50.935	BB	14428.4	149.1	1.4447	99.609
2	70.533	MM	56.7	4.7E-1	2.0156	0.391

b)

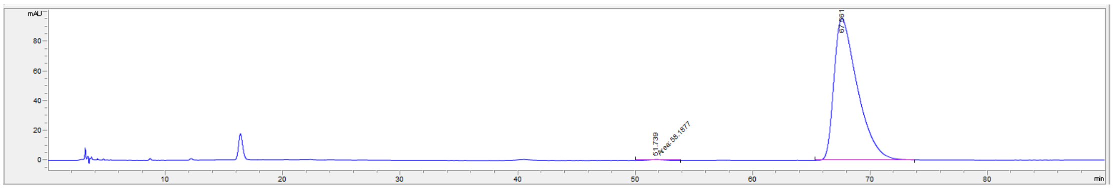

#	Time	Type	Area	Height	Width	Area%
1	51.739	MM	58.2	6.3E-1	1.5336	0.436
2	67.561	BB	13288	96.2	1.9423	99.564

c)

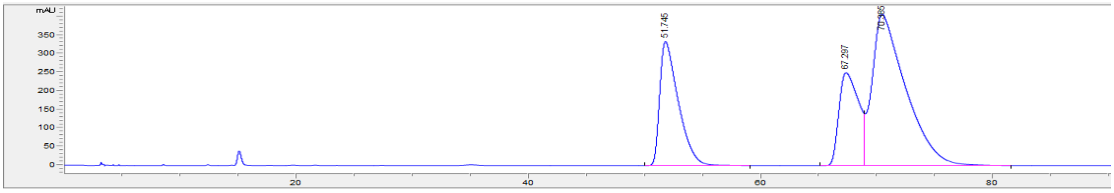

#	Time	Type	Area	Height	Width	Area%
1	51.745	BB	36917.2	333.3	1.6262	24.906
2	67.297	BV	29830.2	249.8	1.7432	20.125
3	70.385	VB	81475.9	407.3	2.7255	54.968

Figure S145: HPLC chromatograms of a) **(*S*, *S*)-3c**, b) **(*R*, *R*)-3c** and c) **a mixture of diasteromers-3c** with optimized eluting protocol (CHIRALPAK® IE; *n*-Hexane/DCM/*i*-PrOH 70/20/10; flow rate: 1.0 mL/min; UV detector wavelength 260 nm; oven temperature 40 °C; inject volume 5.0 µL).

a)

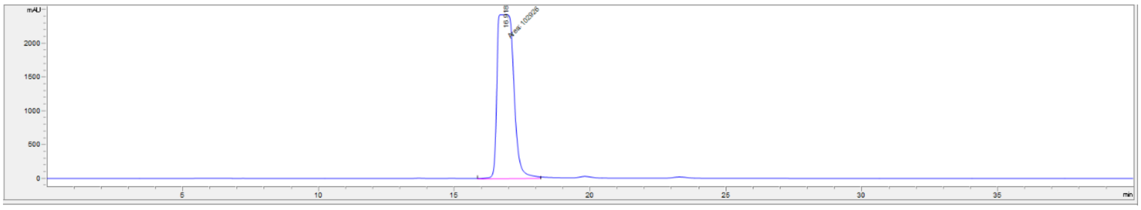

#	Time	Type	Area	Height	Width	Area%
1	16.918	MM	102925.7	2433.5	0.7049	100.000

b)

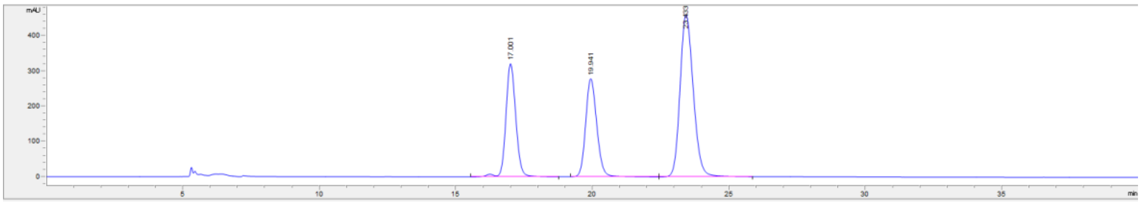

#	Time	Type	Area	Height	Width	Area%
1	17.001	VB R	8099.5	320.8	0.3832	25.720
2	19.941	BB	7879.1	277.9	0.4408	25.020
3	23.433	BB	15512	460.2	0.5233	49.259

c)

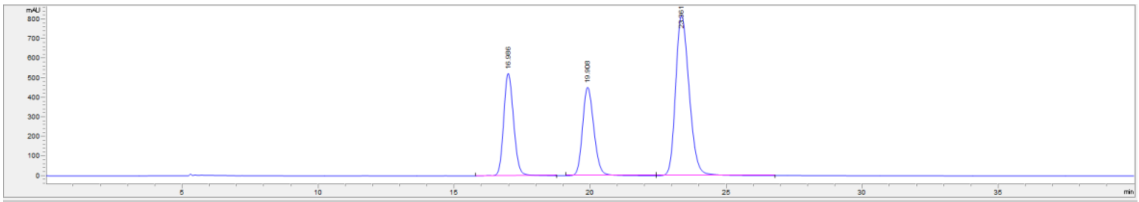

#	Time	Type	Area	Height	Width	Area%
1	16.986	VB R	13442	526.9	0.3948	24.355
2	19.908	BB	13182.1	454.2	0.4506	23.884
3	23.361	BB	28567.1	823.3	0.5388	51.760

Figure S146. HPLC chromatograms of a) (S,S)-**3c**, b) **a mixture of diasteromers-3c** (reaction under DBU and diphenol condition), c) **a mixture of diasteromers-3c** (reaction with sodium diphenolate) with optimized eluting protocol (CHIRALPAK® IA; *n*-Hexane/DCM/*i*-PrOH 75/20/5; flow rate: 0.5 mL/min; UV detector wavelength 260 nm; oven temperature 40 °C; injection volume 5.0 μL).

a)

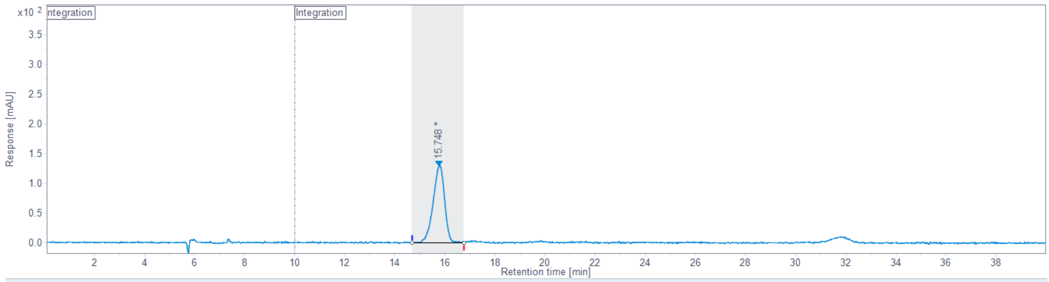

Injection Results

Peaks | Summary

#	Name	Signal description	RT (min)	Area (mAU·s)	Area%	Height (mAU)	Height%	Amount	Concentration	Start time (min)	End time (min)
1		DAD1D,Sig=280,4 Ref=off	15.748	4167.780	100.000	126.946	100.00			14.708	16.771

b)

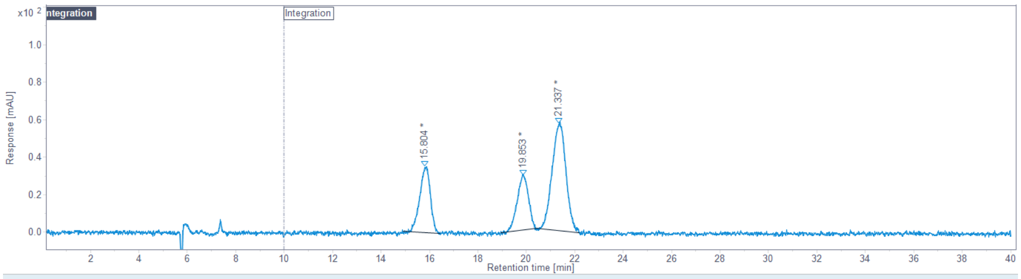

Injection Results

Peaks | Summary

#	Name	Signal description	RT (min)	Area (mAU·s)	Area%	Height (mAU)	Height%	Amount	Concentration	Start time (min)	End time (min)
1		DAD1D,Sig=280,4 Ref=off	15.804	1070.293	24.923	34.778	28.97			14.901	16.428
2		DAD1D,Sig=280,4 Ref=off	19.853	937.808	21.838	28.415	23.67			18.962	20.428
3		DAD1D,Sig=280,4 Ref=off	21.337	2286.329	53.239	56.866	47.36			20.428	22.199

Figure S147: HPLC chromatograms of a) **(*S*, *S*)-3d** and b) **a mixture of diasteromers-3d** with optimized eluting protocol (CHIRALPAK® IA; *n*-Hexane/DCM/MeOH 80/20/5; flow rate: 0.5 mL/min; UV detector wavelength 280 nm; inject volume 5.0 μL).

a)

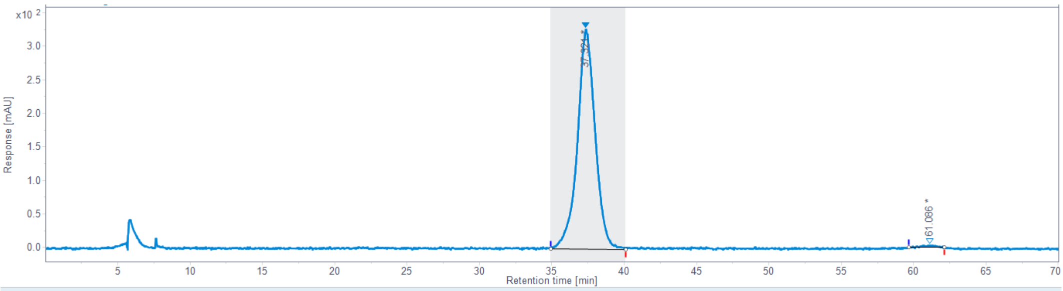

Injection Results

Peaks | Summary

#	Name	Signal description	RT (min)	Area (mAU·s)	Area%	Height (mAU)	Height%	Amount	Concentration	Start time (min)	End time (min)
1		DAD1C,Sig=280,4 Ref=off	37.321	27811.396	99.207	327.760	98.67			34.893	40.077
2		DAD1C,Sig=280,4 Ref=off	61.086	222.365	0.793	4.429	1.33			59.634	62.107

b)

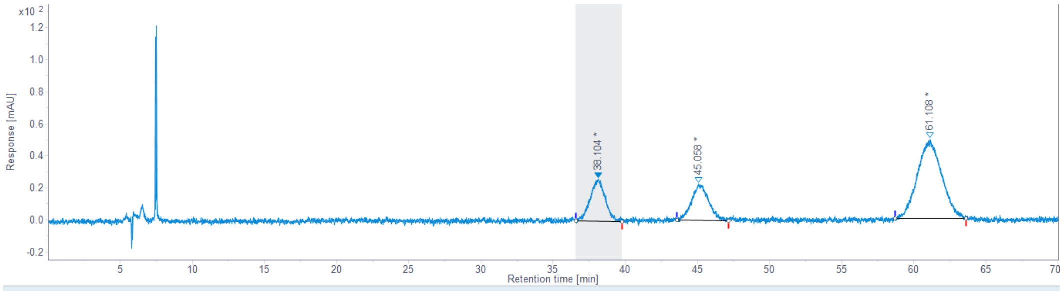

Injection Results

Peaks | Summary

#	Name	Signal description	RT (min)	Area (mAU·s)	Area%	Height (mAU)	Height%	Amount	Concentration	Start time (min)	End time (min)
1		DAD1C,Sig=280,4 Ref=off	38.104	1887.748	20.031	26.190	26.68			36.577	39.769
2		DAD1C,Sig=280,4 Ref=off	45.058	1847.164	19.600	22.673	23.09			43.591	47.147
3		DAD1C,Sig=280,4 Ref=off	61.108	5689.353	60.369	49.317	50.23			58.690	63.636

Figure S148: HPLC chromatograms of a) **(*S, S*)-3f** and b) **a mixture of diasteromers-3f** with optimized eluting protocol (CHIRALPAK® IA; *n*-Hexane/DCM/MeOH 80/20/5; flow rate: 0.5 mL/min; UV detector wavelength 280 nm; inject volume 5.0 µL).

a)

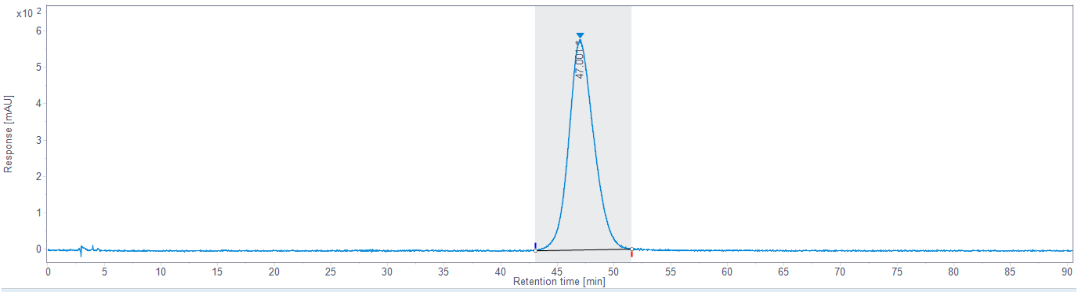

Injection Results

Peaks | Summary

#	Name	Signal description	RT (min)	Area (mAU·s)	Area%	Height (mAU)	Height%	Amount	Concentration	Start time (min)	End time (min)
1		DAD1B,Sig=240,4 Ref=off	47.001	88252.265	100.000	576.368	100.00			43.055	51.574

b)

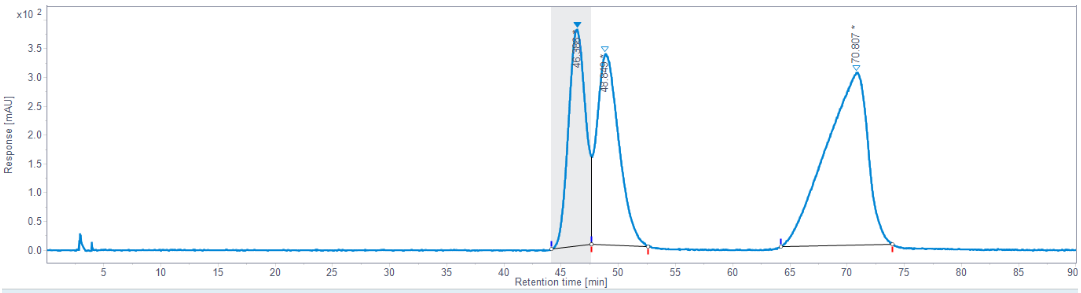

Injection Results

Peaks | Summary

#	Name	Signal description	RT (min)	Area (mAU·s)	Area%	Height (mAU)	Height%	Amount	Concentration	Start time (min)	End time (min)
1		DAD1B,Sig=240,4 Ref=off	46.386	39489.675	24.545	377.163	37.34			44.162	47.637
2		DAD1B,Sig=240,4 Ref=off	48.849	44648.270	27.752	332.346	32.90			47.637	52.579
3		DAD1B,Sig=240,4 Ref=off	70.807	76745.897	47.703	300.521	29.75			64.178	73.977

Figure S149: HPLC chromatograms of a) **(*S*, *S*)-3k** and b) **a mixture of diasteromers-3k** with optimized eluting protocol (CHIRALPAK® IA; *n*-Hexane/DCM/MeOH 80/20/5; flow rate: 0.5 mL/min; UV detector wavelength 240 nm; inject volume 5.0 µL).

a)

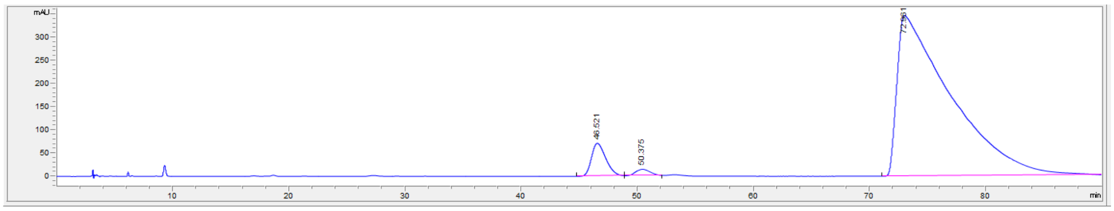

#	Time	Type	Area	Height	Width	Area%
1	46.521	BB	5984	70.3	1.2826	5.060
2	50.375	BB	1147.3	13.5	1.1417	0.970
3	72.961	BBA	111124.9	344.7	4.005	93.970

b)

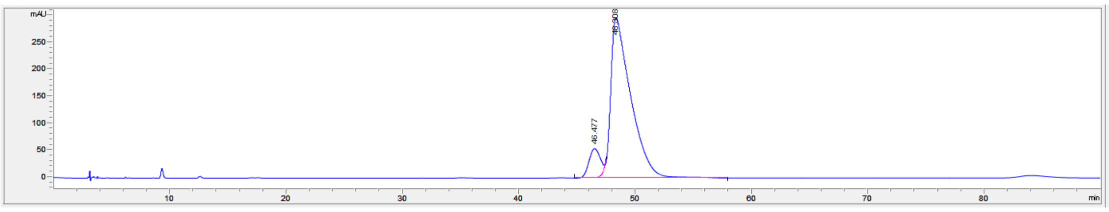

#	Time	Type	Area	Height	Width	Area%
1	46.477	BV E	3720.3	54.4	1.0736	9.438
2	48.308	VB R	35698.5	296.9	1.6469	90.562

c)

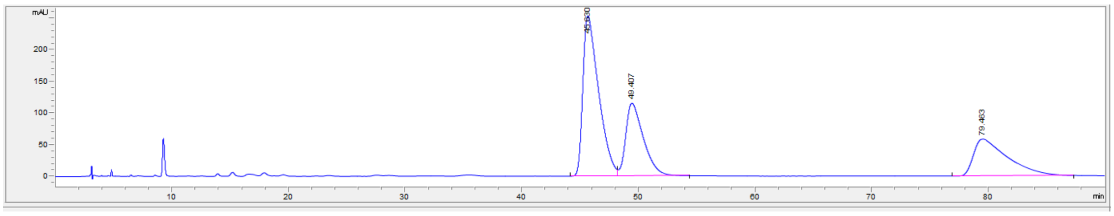

#	Time	Type	Area	Height	Width	Area%
1	45.63	BV	23634.5	252.2	1.3674	49.853
2	49.407	VB	12059.5	114.4	1.5159	25.437
3	79.463	BB	11714.8	58	2.622	24.710

Figure S150: HPLC chromatograms of a) **(*S*, *S*)-3o,** b) **(*R*, *R*)-3o** and c) **a mixture of diasteromers-3o** with optimized eluting protocol (CHIRALPAK® IE; *n*-Hexane/DCM/*i*-PrOH 55/40/5; flow rate: 1.0 mL/min; UV detector wavelength 254 nm; oven temperature 40 °C; inject volume 5.0 µL).

a)

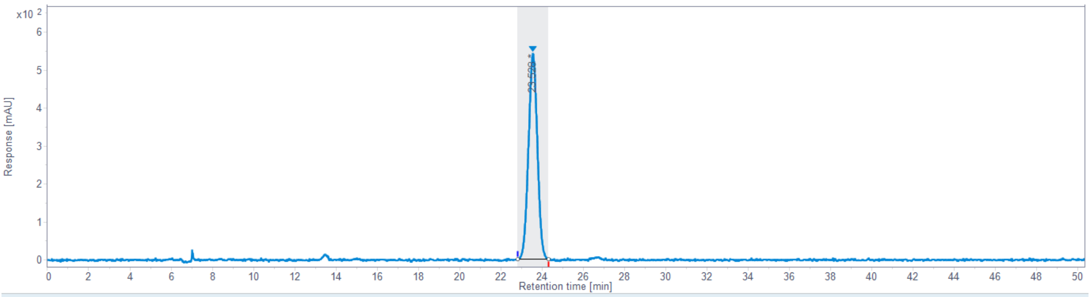

Injection Results

Peaks | Summary

#	Name	Signal description	RT (min)	Area (mAU·s)	Area%	Height (mAU)	Height%	Amount	Concentration	Start time (min)	End time (min)
1		DAD1B,Sig=260,4 Ref=off	23.528	16204.561	100.000	543.576	100.00			22.792	24.290

b)

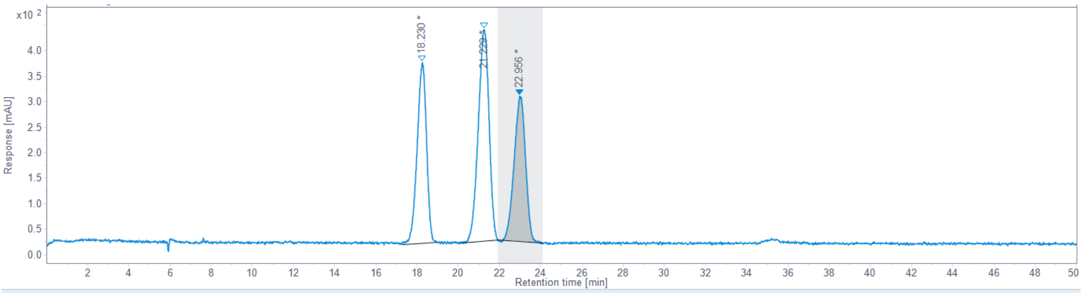

Injection Results

Peaks | Summary

#	Name	Signal description	RT (min)	Area (mAU·s)	Area%	Height (mAU)	Height%	Amount	Concentration	Start time (min)	End time (min)
1		DAD1A,Sig=240,4 Ref=off	18.230	11235.828	30.097	355.061	33.60			17.253	18.933
2		DAD1A,Sig=240,4 Ref=off	21.229	15146.075	40.572	416.228	39.38			20.116	21.910
3		DAD1A,Sig=240,4 Ref=off	22.956	10949.670	29.331	285.561	27.02			21.910	24.086

Figure S151: HPLC chromatograms of a) **(*S*, *S*)-3r** and b) **a mixture of diasteromers-3r** with optimized eluting protocol (CHIRALPAK® IA; *n*-Hexane/DCM/MeOH 80/20/5; flow rate: 0.5 mL/min; UV detector wavelength 240 nm; inject volume 5.0 μL).

a)

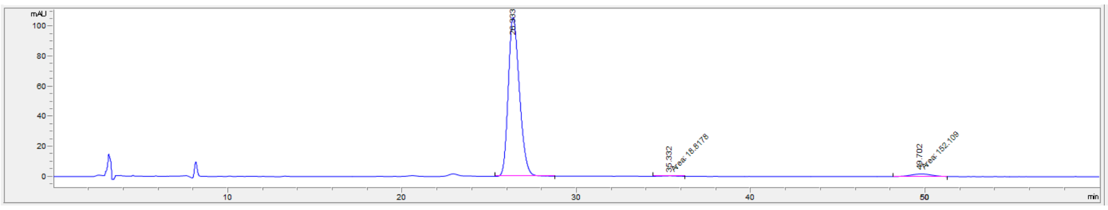

#	Time	Type	Area	Height	Width	Area%
1	26.333	BB	4702.2	105.5	0.6859	96.492
2	35.332	MM	18.8	3.6E-1	0.8596	0.386
3	49.702	MM	152.1	1.8	1.4208	3.121

b)

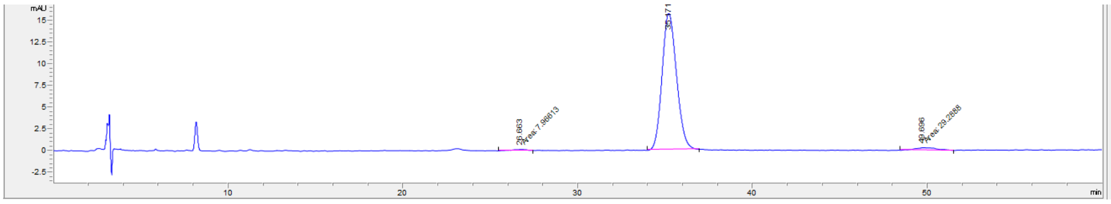

#	Time	Type	Area	Height	Width	Area%
1	26.663	MM	8	1.3E-1	1.0012	0.840
2	35.171	BB	910.6	15.8	0.8594	96.070
3	49.696	MM	29.3	3.3E-1	1.4992	3.090

c)

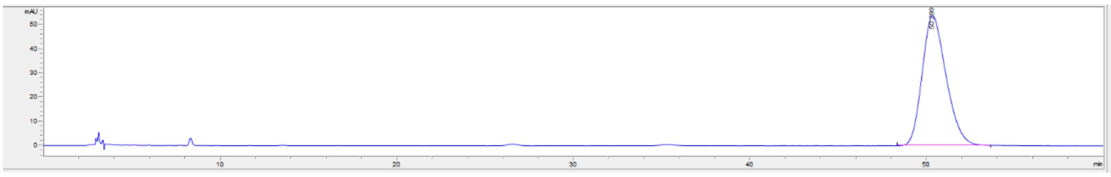

#	Time	Type	Area	Height	Width	Area%
1	50.299	BB	5083.7	54.1	1.3766	100.000

d)

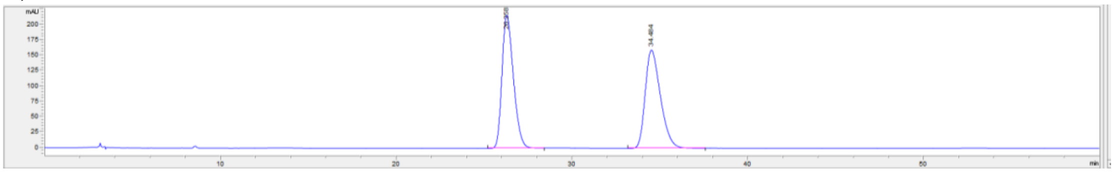

#	Time	Type	Area	Height	Width	Area%
1	26.258	BB	9748.8	216.3	0.6939	50.343
2	34.484	BB	9616.1	159.8	0.9139	49.657

e)

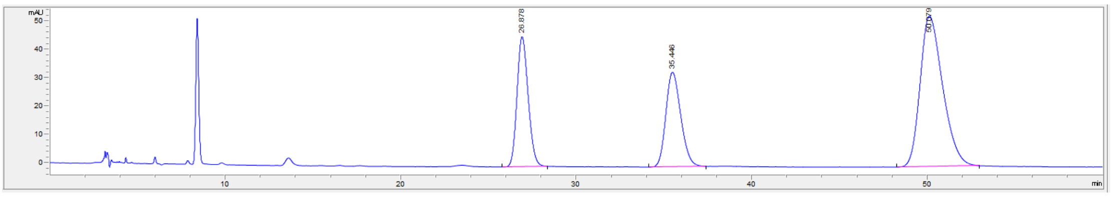

#	Time	Type	Area	Height	Width	Area%
1	26.878	BB	2021.9	45.8	0.6814	23.141
2	35.446	BB	1970.5	33.3	0.9084	22.554
3	50.079	BB	4744.7	53	1.3406	54.305

Figure S152: HPLC chromatograms of a) **(*S*, *S*)-3u,** b) **(*R*, *R*)-3u**, c) **meso-3u** d) **(*S*, *S*) & (*R*, *R*)-3u**, and e) **a mixture of diasteromers-3u** with optimized eluting protocol (CHIRALPAK® IE; *n*-Hexane/DCM/*i*-PrOH 60/30/10; flow rate: 1.0 mL/min; UV detector wavelength 254 nm; oven temperature 40 °C; inject volume 5.0 µL).

a)

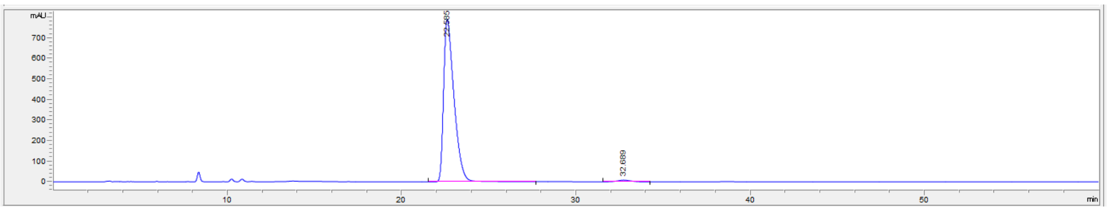

#	Time	Type	Area	Height	Width	Area%
1	22.585	BB	32709.7	790	0.6231	98.674
2	32.689	BB	439.5	8.3	0.7991	1.326

b)

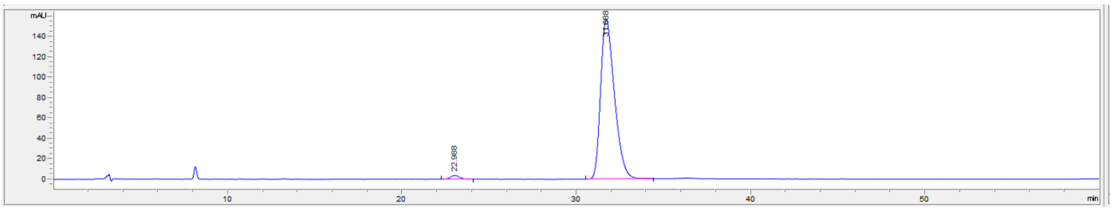

#	Time	Type	Area	Height	Width	Area%
1	22.988	BB	134.4	3.6	0.5557	1.537
2	31.688	BB	8607.1	156.7	0.8429	98.463

c)

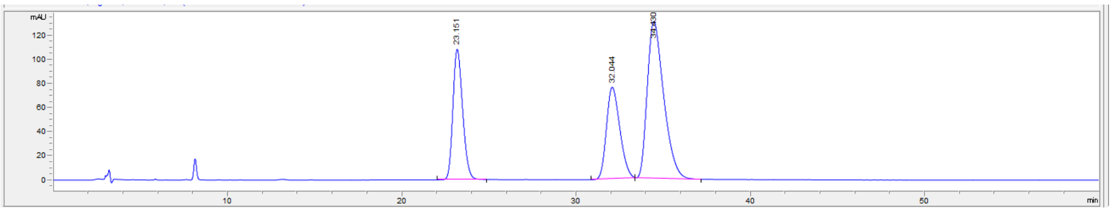

#	Time	Type	Area	Height	Width	Area%
1	23.151	BB	4199.4	108.5	0.5975	25.018
2	32.044	BB	4055.7	76.2	0.8287	24.162
3	34.43	BB	8530.5	130.4	0.9994	50.820

Figure S153: HPLC chromatograms of a) **(*S*, *S*)-3v,** b) **(*R*, *R*)-3v** and c) **a mixture of diasteromers-3v** with optimized eluting protocol (CHIRALPAK® IE; *n*-Hexane/DCM/*i*-PrOH 60/30/10; flow rate: 1.0 mL/min; UV detector wavelength 254 nm; oven temperature 40 °C; inject volume 5.0 μL).

a)

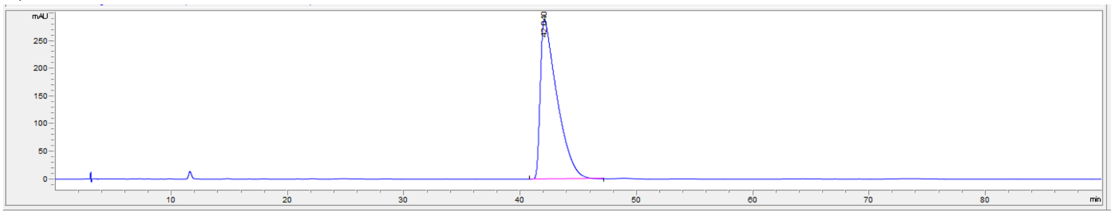

#	Time	Type	Area	Height	Width	Area%
1	42.04	BB	28696.2	287	1.4004	100.000

b)

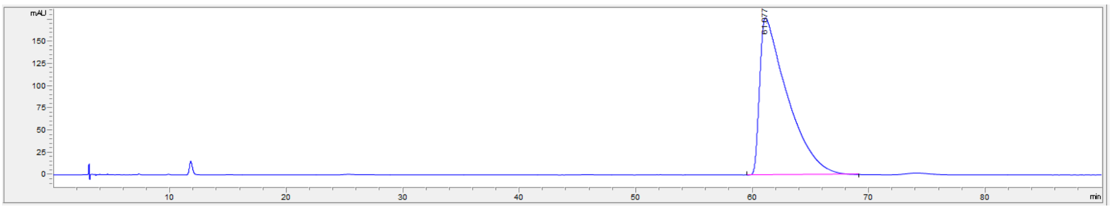

#	Time	Type	Area	Height	Width	Area%
1	61.077	BB	29323.2	177.7	2.2067	100.000

c)

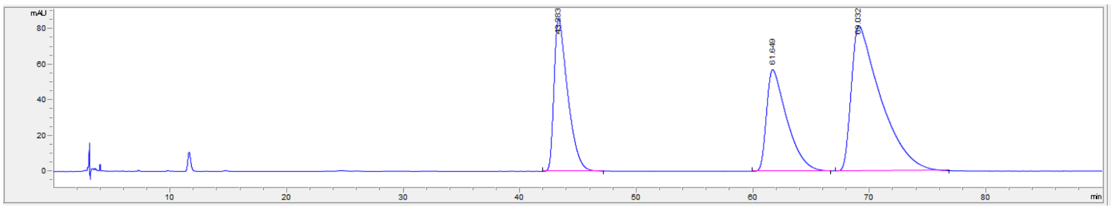

#	Time	Type	Area	Height	Width	Area%
1	43.283	BB	7009.5	86.4	1.1661	24.673
2	61.649	BB	6980.9	57.4	1.7169	24.572
3	69.032	BB	14419.3	81.9	2.3395	50.755

Figure S154: HPLC chromatograms of a) **(*S*, *S*)-3w,** b) **(*R*, *R*)-3w** and c) **a mixture of diasteromers-3w** with optimized eluting protocol (CHIRALPAK® IE; *n*-Hexane/DCM/*i*-PrOH 60/35/5; flow rate: 1.0 mL/min; UV detector wavelength 260 nm; oven temperature 40 °C; inject volume 5.0 µL).

a)

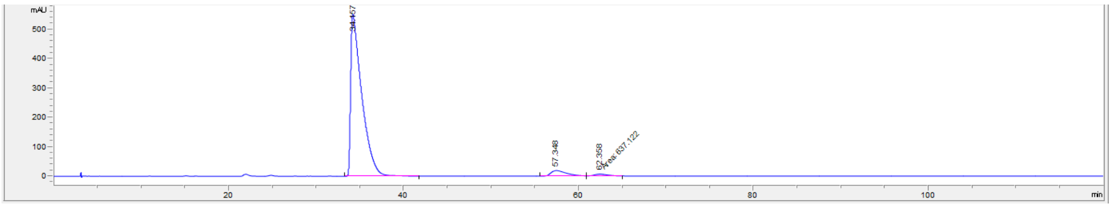

#	Time	Type	Area	Height	Width	Area%
1	34.157	BB	48567.1	552.2	1.2542	94.416
2	57.348	BB	2235.4	18.8	1.5233	4.346
3	62.358	MM	637.1	6.1	1.7341	1.239

b)

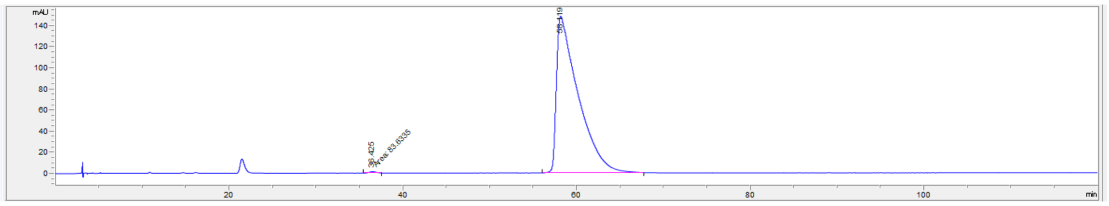

#	Time	Type	Area	Height	Width	Area%
1	36.425	MM	83.6	1.4	1	0.324
2	58.119	BB	25715.1	148.3	2.2826	99.676

c)

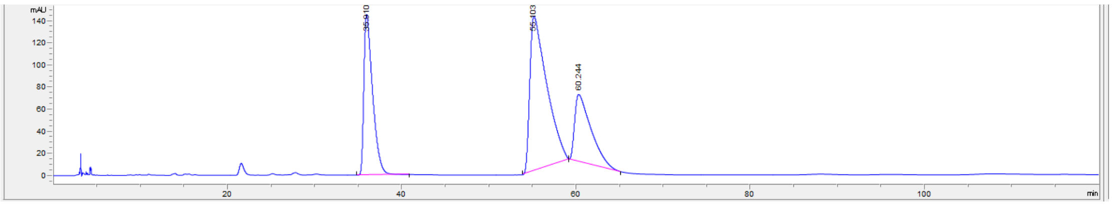

#	Time	Type	Area	Height	Width	Area%
1	35.91	BB	10143.6	145.7	1.0403	27.359
2	55.103	BB	18925.9	140.6	1.8461	51.047
3	60.244	BB	8006	60.9	1.788	21.594

Figure S155: HPLC chromatograms of a) **(*S*, *S*)-3x,** b) **(*R*, *R*)-3x** and c) **Racemica mixture of diasteromers-3x** with optimized eluting protocol (CHIRALPAK® IE; *n*-Hexane/DCM/*i*-PrOH 70/25/5; flow rate: 1.0 mL/min; UV detector wavelength 260 nm; oven temperature 40 °C; inject volume 5.0 μL).

a)

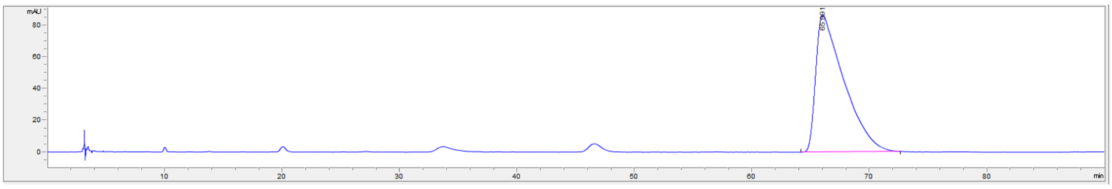

#	Time	Type	Area	Height	Width	Area%
1	65.991	BB	14454.6	86.4	2.2236	100.000

b)

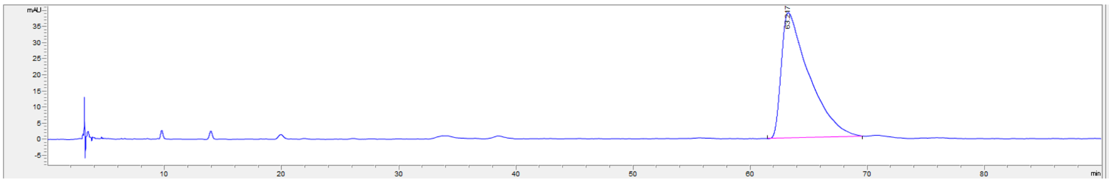

#	Time	Type	Area	Height	Width	Area%
1	63.217	BB	6396.4	38.7	2.1189	100.000

c)

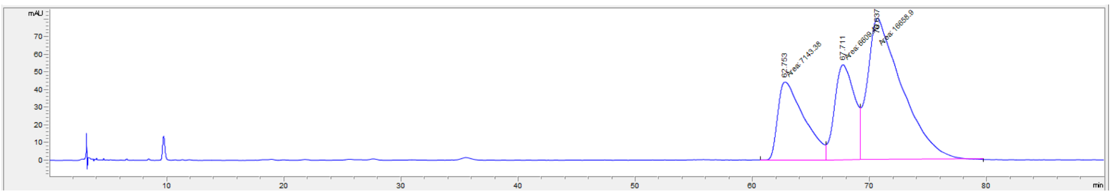

#	Time	Type	Area	Height	Width	Area%
1	62.753	MF	7143.4	44.9	2.6524	23.489
2	67.711	MF	6609.4	54.6	2.0187	21.733
3	70.637	FM	16658.9	81.1	3.4253	54.778

Figure S156: HPLC chromatograms of a) ***(S, S)*-3y,** b) ***(R, R)*-3y** and c) **a mixture of diasteromers-3y** with optimized eluting protocol (CHIRALPAK® IE; *n*-Hexane/DCM/*i*-PrOH 55/40/5; flow rate: 1.0 mL/min; UV detector wavelength 260 nm; oven temperature 40 °C; inject volume 5.0 µL).

a)

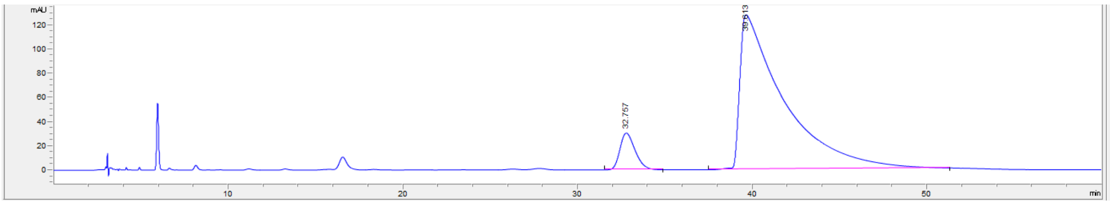

#	Time	Type	Area	Height	Width	Area%
1	32.757	BB	1839	30.6	0.9162	7.554
2	39.613	BB	22506.6	128.6	2.2534	92.446

b)

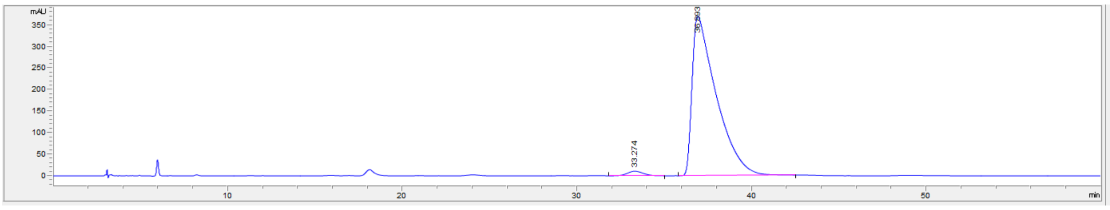

#	Time	Type	Area	Height	Width	Area%
1	33.274	BB	678.4	10.7	0.9183	1.867
2	36.893	BB	35657.9	371.6	1.3095	98.133

c)

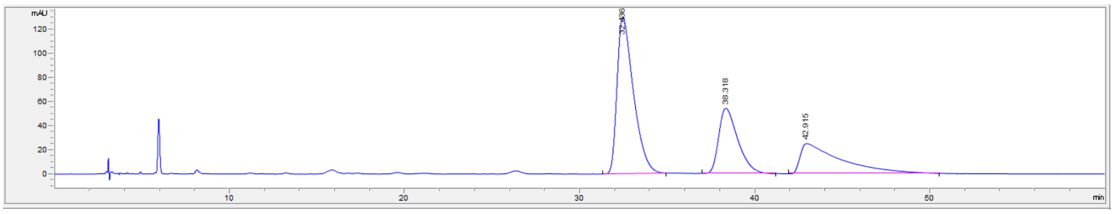

#	Time	Type	Area	Height	Width	Area%
1	32.436	BB	8419.9	129.7	0.9755	51.104
2	38.318	BB	4080.4	53.9	1.1149	24.766
3	42.915	BB	3975.8	24.9	1.9881	24.131

Figure S157: HPLC chromatograms of a) **(*S*, *S*)-3Aa,** b) **(*R*, *R*)-3Aa** and c) **a mixture of diasteromers-3Aa** with optimized eluting protocol (CHIRALPAK® IE; *n*-Hexane/DCM/*i*-PrOH 40/55/5; flow rate: 1.0 mL/min; UV detector wavelength 260 nm; oven temperature 40 °C; inject volume 5.0 µL).

a)

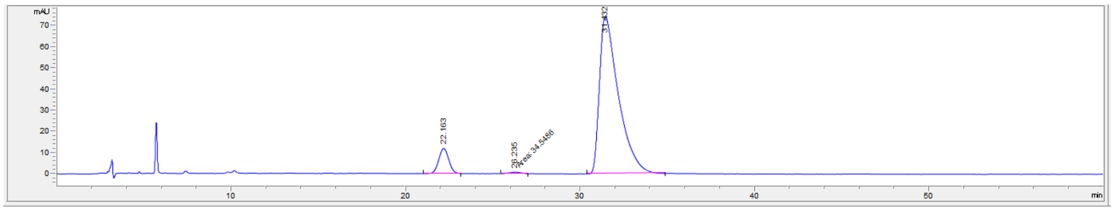

#	Time	Type	Area	Height	Width	Area%
1	22.163	BB	479.7	12	0.618	7.797
2	26.235	MM	34.5	7.4E-1	0.7804	0.562
3	31.432	BB	5638.6	74.8	1.1098	91.641

b)

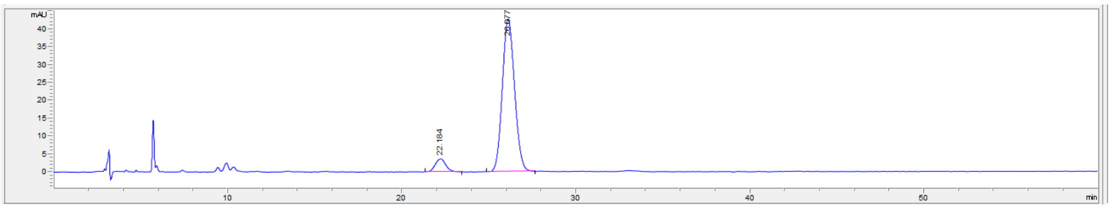

#	Time	Type	Area	Height	Width	Area%
1	22.184	BB	151.8	3.6	0.5994	6.780
2	26.077	BB	2087.2	42.8	0.7552	93.220

c)

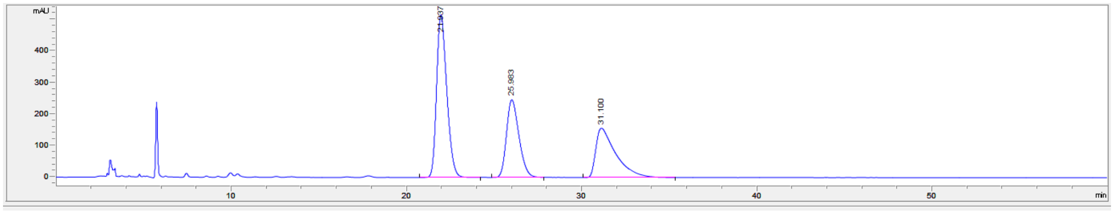

#	Time	Type	Area	Height	Width	Area%
1	21.937	BB	20836.5	515.6	0.6235	45.997
2	25.983	BB	12154.6	246.2	0.7619	26.831
3	31.1	BB	12308.9	156.4	1.0884	27.172

Figure S158: HPLC chromatograms of a) **(*S*, *S*)-Ab,** b) **(*R*, *R*)-3Ab** and c) **a mixture of diasteromers-3Ab** with optimized eluting protocol (CHIRALPAK® IE; *n*-Hexane/DCM/*i*-PrOH 50/40/10; flow rate: 1.0 mL/min; UV detector wavelength 254 nm; oven temperature 40 °C; inject volume 5.0 μL).

a)

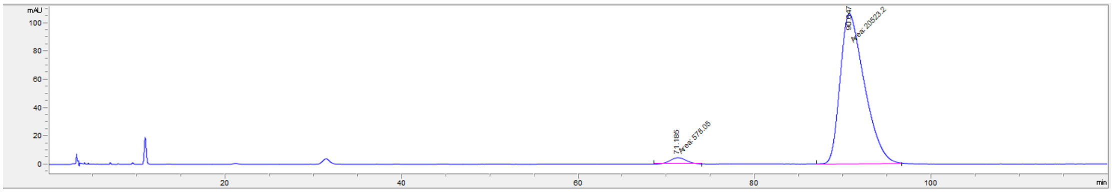

#	Time	Type	Area	Height	Width	Area%
1	71.185	MM	578.1	4.5	2.1625	2.739
2	90.647	MM	20523.2	106.4	3.2134	97.261

b)

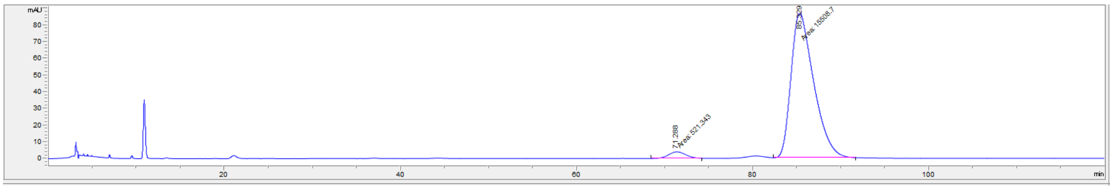

#	Time	Type	Area	Height	Width	Area%
1	71.288	MM	521.3	3.9	2.2297	3.252
2	85.329	MM	15508.7	86.4	2.9924	96.748

c)

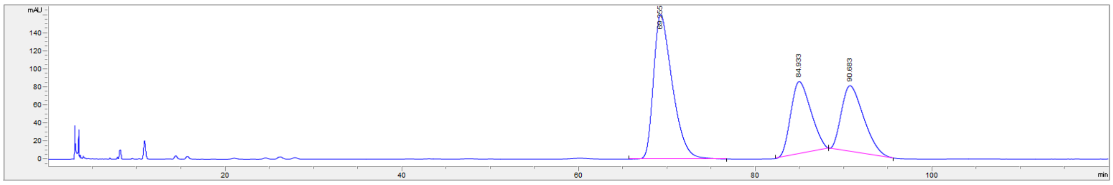

#	Time	Type	Area	Height	Width	Area%
1	69.255	BB	23834.2	161.2	2.1029	48.169
2	84.933	BB	12804.5	80.9	2.1828	25.878
3	90.683	BB	12841.4	73.5	2.3173	25.953

Figure S159: HPLC chromatograms of a) **(*S*, *S*)-Ac,** b) **(*R*, *R*)-3Ac** and c) **a mixture of diasteromers-3Ac** with optimized eluting protocol (CHIRALPAK® IE; *n*-Hexane/DCM/*i*-PrOH 65/25/10; flow rate: 1.0 mL/min; UV detector wavelength 260 nm; oven temperature 40 °C; inject volume 5.0 µL).

a)

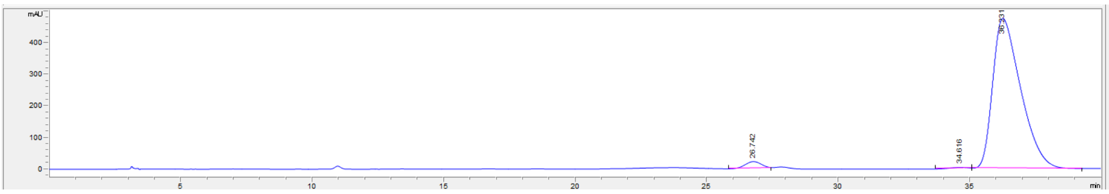

#	Time	Type	Area	Height	Width	Area%
1	26.742	BB	866.8	20.9	0.6586	2.404
2	34.616	BB	125.8	3.1	0.5333	0.349
3	36.231	BB	35062	475.9	1.1095	97.247

b)

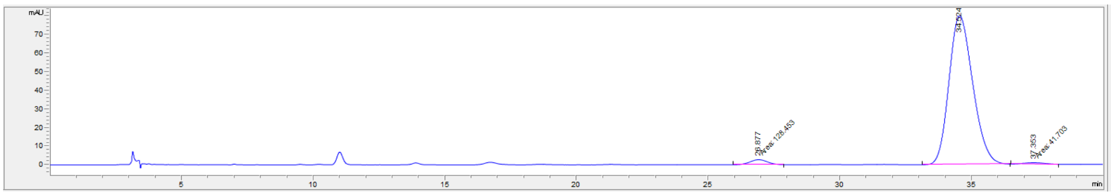

#	Time	Type	Area	Height	Width	Area%
1	26.877	MM	128.5	2.7	0.7833	2.487
2	34.524	BB	4995.7	79.7	0.9634	96.706
3	37.353	MM	41.7	7.5E-1	0.9216	0.807

c)

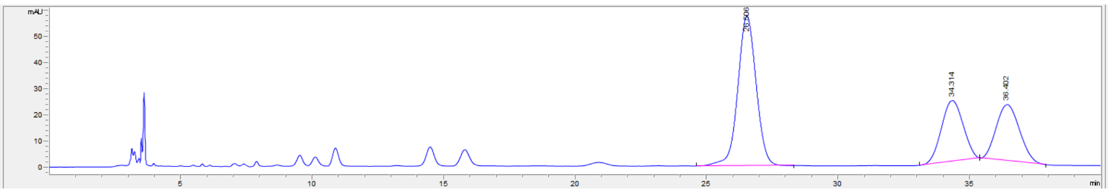

#	Time	Type	Area	Height	Width	Area%
1	26.506	BB	2788.6	57.4	0.7511	51.100
2	34.314	BB	1340.8	23.3	0.8764	24.569
3	36.402	BB	1327.8	21.5	0.951	24.331

Figure S160: HPLC chromatograms of a) **(*S, S*)-3Ad,** b) **(*R, R*)-3Ad** and c) **a mixture of diasteromers-3Ad** with optimized eluting protocol (CHIRALPAK® IE; *n*-Hexane/DCM/*i*-PrOH 65/25/10; flow rate: 1.0 mL/min; UV detector wavelength 260 nm; oven temperature 40 °C; inject volume 5.0 μL).

a)

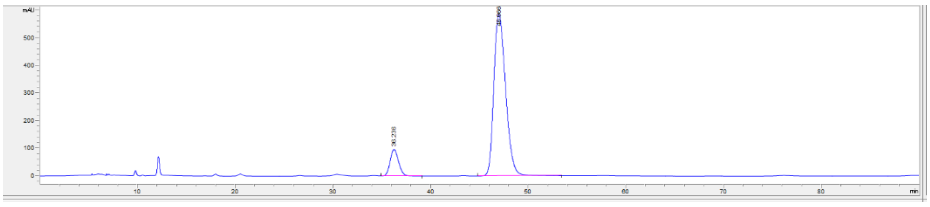

#	Time	Type	Area	Height	Width	Area%
1	36.236	BB	5761.7	95.8	0.9256	10.521
2	46.966	BB	49003	582.8	1.315	89.479

b)

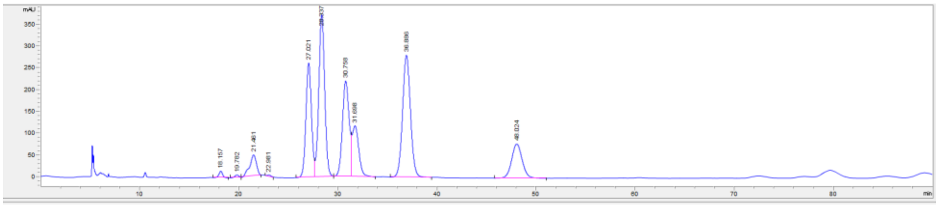

Area	Height	Width	Area%	#	Time	Type
383.2	15	0.3856	0.581	1	18.157	BB
161.4	6	0.4137	0.245	2	19.782	BB
2323	48.5	0.7068	3.521	3	21.461	BB
68.6	2.8	0.3794	0.104	4	22.981	BB
10357.4	262.5	0.6104	15.700	5	27.021	BV
15422.7	372.3	0.6393	23.378	6	28.337	VB
10439.8	219.5	0.7208	15.825	7	30.758	BV
5380.7	116.7	0.6879	8.156	8	31.698	VB
15663.7	281	0.8604	23.743	9	36.886	BB
5770.8	78.7	1.127	8.747	10	48.024	BB

Figure S161: HPLC chromatograms of a) **(*S*, *S*, *S*, *S*)-4a** and b) **a mixture of diasteromers-4a** with optimized eluting protocol (CHIRALPAK® IA; *n*-Hexane/DCM/MeOH 65/30/5; UV detector wavelength 260 nm; flow rate: 0.5 mL/min; inject volume 5.0 µL).

a)

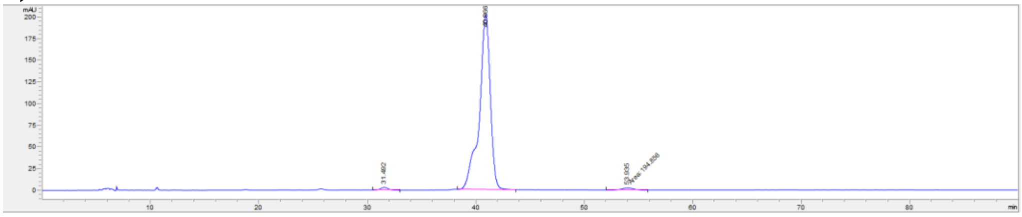

#	Time	Type	Area	Height	Width	Area%
1	31.492	BB	150.7	3.2	0.5742	1.031
2	40.866	BB	14271.1	201.2	1.0496	97.636
3	53.935	MM	194.9	2.4	1.3265	1.333

b)

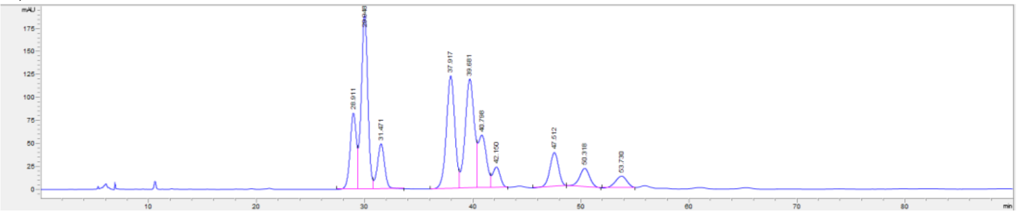

#	Time	Type	Area	Height	Width	Area%
1	28.911	BV	3399.6	83.1	0.6152	9.445
2	29.948	VV	8112.2	189.5	0.6553	22.539
3	31.471	VB	2289.3	49.1	0.7096	6.361
4	37.917	BV	6714.8	122.8	0.8399	18.656
5	39.681	VV	7086.9	118.9	0.9012	19.690
6	40.798	VV	2987.2	57.5	0.7757	8.300
7	42.15	VB	1076.2	22.4	0.7208	2.990
8	47.512	BB	2080.1	37	0.8735	5.779
9	50.318	BB	1307.5	20.1	0.9591	3.633
10	53.73	BB	938.7	12.6	0.9638	2.608

Figure S162: HPLC chromatograms of a) **(*S*, *S*, *S*, *S*)-4b** and b) **a mixture of diasteromers-4b** with optimized eluting protocol (CHIRALPAK® IA; *n*-Hexane/DCM/MeOH 65/30/5; UV detector wavelength 260 nm; flow rate: 0.5 mL/min; inject volume 5.0 µL).

a)

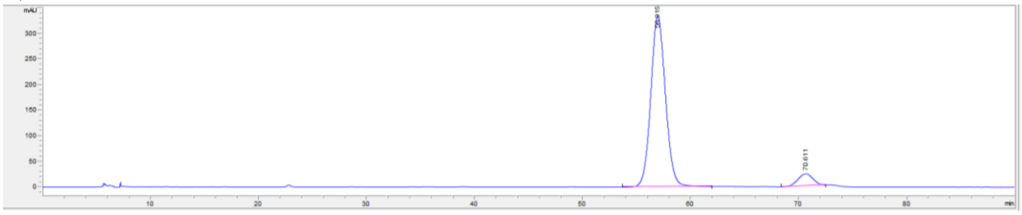

#	Time	Type	Area	Height	Width	Area%
1	56.915	BB	31682.5	335	1.4595	93.221
2	70.611	BB	2304	23.7	1.3498	6.779

b)

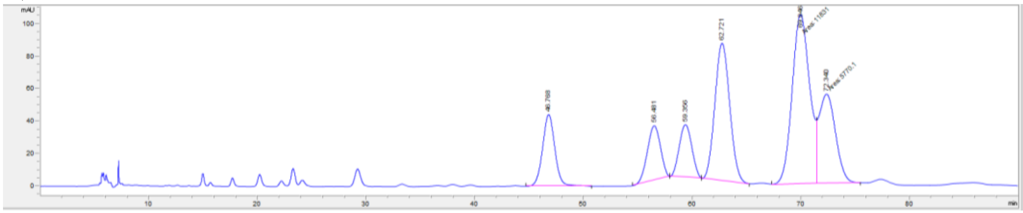

#	Time	Type	Area	Height	Width	Area%
1	46.768	BB	3467.3	44	1.1806	10.038
2	56.481	BB	2828.2	33.6	1.2326	8.188
3	59.356	BB	2611.9	32.4	1.2048	7.561
4	62.721	BB	8033.5	84.5	1.4634	23.257
5	69.946	MF	11831	104.1	1.8937	34.251
6	72.34	FM	5770.1	55	1.7471	16.705

Figure S163: HPLC chromatograms of a) **(*S*, *S*, *S*, *S*)-4c** and b) **a mixture of diasteromers-4c** with optimized eluting protocol (CHIRALPAK® IA; *n*-Hexane/DCM/MeOH 85/15/5; UV detector wavelength 260 nm; flow rate: 0.5 mL/min; inject volume 5.0 µL).

a)

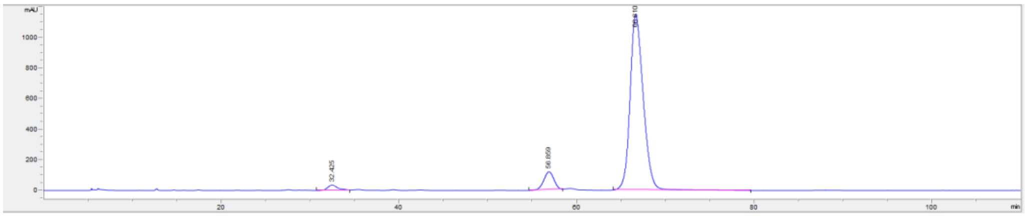

#	Time	Type	Area	Height	Width	Area%
1	32.425	BB	2556.1	33.4	1.0979	1.968
2	56.859	BB	9218	116.7	1.2274	7.098
3	66.61	BB	118102	1142.5	1.556	90.934

b)

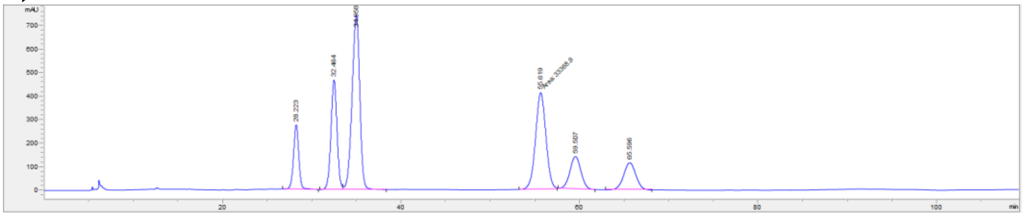

#	Time	Type	Area	Height	Width	Area%
1	28.223	BB	11185	273.7	0.6289	8.364
2	32.464	BV	22024.1	465.8	0.7277	16.469
3	34.958	VB	44502.2	741.4	0.9283	33.277
4	55.619	MM	33368.9	410.4	1.3552	24.952
5	59.507	BB	11672.1	137.2	1.3144	8.728
6	65.596	BB	10980.6	115.4	1.4539	8.211

Figure S164: HPLC chromatograms of a) **(*S*, *S*, *S*, *S*)-4d** and b) **a mixture of diasteromers-4d** with optimized eluting protocol (CHIRALPAK® IA; *n*-Hexane/DCM/MeOH 70/25/5; UV detector wavelength 260 nm; flow rate: 0.5 mL/min; inject volume 5.0 µL).

a)

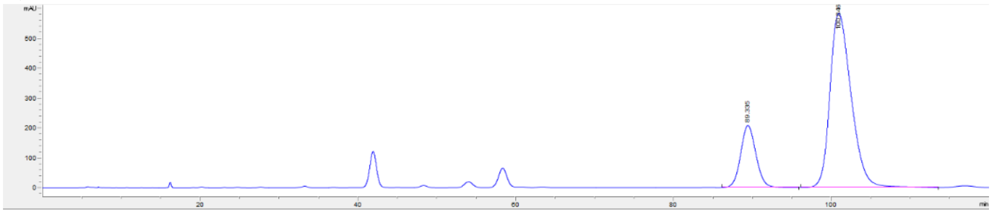

#	Time	Type	Area	Height	Width	Area%
1	89.335	BB	27759.9	207.1	1.9981	20.370
2	100.846	BB	108521.7	580.9	2.8117	79.630

b)

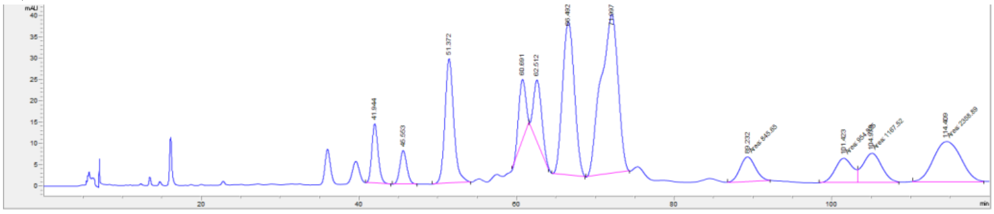

#	Time	Type	Area	Height	Width	Area%
1	41.944	BB	784.8	14	0.8246	3.865
2	45.553	BB	486.6	7.9	0.848	2.396
3	51.372	BB	2291.7	29.4	1.162	11.285
4	60.691	BB	948	14.8	0.9536	4.668
5	62.512	BB	985.2	14.1	0.9507	4.852
6	66.492	BB	3790.9	36.2	1.5165	18.668
7	71.997	BB	5693.1	37.6	1.9867	28.035
8	89.232	MM	845.7	6	2.3539	4.164
9	101.423	MF	954.5	5.9	2.7026	4.700
10	104.979	FM	1167.5	7.1	2.7489	5.749
11	114.409	MM	2358.9	9.6	4.1054	11.616

Figure S165: HPLC chromatograms of a) **(*S*, *S*, *S*, *S*)-4e** and b) **a mixture of diasteromers-4e** with optimized eluting protocol (CHIRALPAK® IA; *n*-Hexane/DCM/MeOH 75/20/5; UV detector wavelength 254 nm; flow rate: 0.5 mL/min; inject volume 5.0 μL)

a)

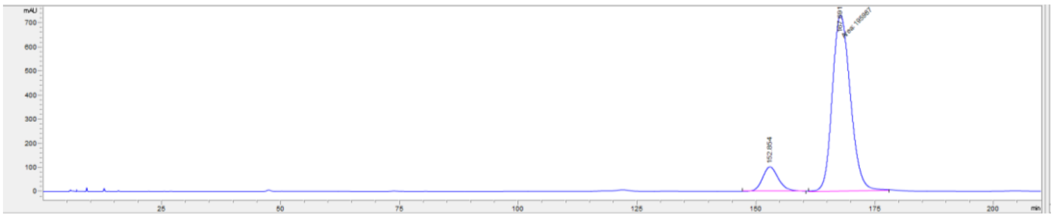

#	Time	Type	Area	Height	Width	Area%
1	152.854	BB	23268.4	101.1	2.7742	10.612
2	167.691	MM	195987	731.2	4.467	89.388

b)

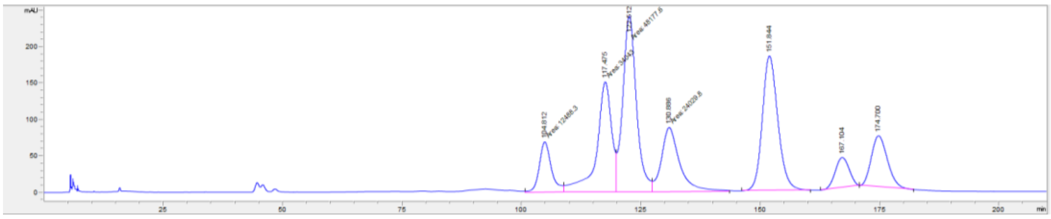

#	Time	Type	Area	Height	Width	Area%
1	104.812	MF	12488.3	69.7	2.9845	6.729
2	117.475	MF	34043	152.1	3.7315	18.342
3	122.512	MF	48177.6	242.3	3.3141	25.958
4	130.886	FM	24029.8	89	4.4984	12.947
5	151.844	BB	41936.1	185.3	3.1669	22.595
6	167.104	BB	8385.8	41.3	2.3873	4.518
7	174.7	BB	16536.3	70.3	2.7623	8.910

Figure S166: HPLC chromatograms of a) **(*S*, *S*, *S*, *S*)-4f** and b) **a mixture of diasteromers-4f** with optimized eluting protocol (CHIRALPAK® IA; *n*-Hexane/DCM/MeOH 75/20/5; UV detector wavelength 280 nm; flow rate: 0.5 mL/min; inject volume 5.0 μL).

a)

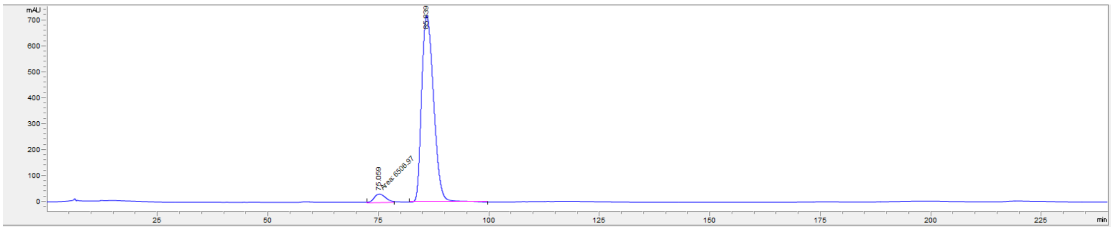

#	Time	Type	Area	Height	Width	Area%
1	75.059	MM	6507	34.3	3.1606	4.574
2	85.839	BB	135744.1	718.1	2.8533	95.426

b)

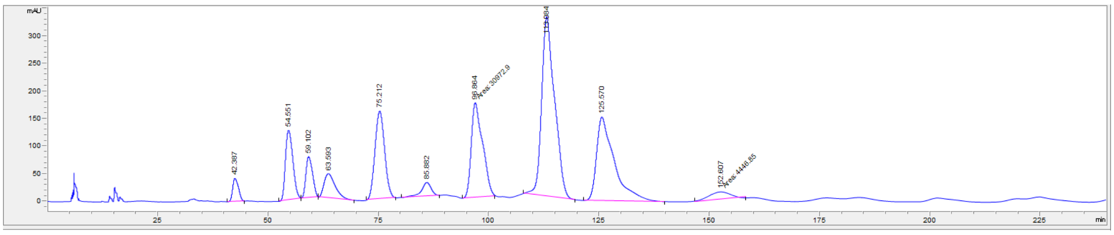

#	Time	Type	Area	Height	Width	Area%
1	42.387	BB	3847.8	42.1	1.2892	1.866
2	54.551	BB	14794.4	126.6	1.779	7.175
3	59.102	BB	7825.4	74.5	1.5192	3.795
4	63.593	BB	7739.2	44.1	2.1292	3.753
5	75.212	BB	24302.9	159.6	2.2069	11.787
6	85.882	BB	3821.8	24.7	1.8183	1.854
7	96.864	MM	30972.9	172.6	2.991	15.022
8	113.084	BB	66245.8	325.8	2.8185	32.129
9	125.57	BB	42190.1	151.7	3.6354	20.462
10	152.607	MM	4446.8	13.3	4.0245	2.157

Figure S167: HPLC chromatograms of a) **(*S*, *S*, *S*, *S*)-4g** and b) **a mixture of diasteromers-4g** with optimized eluting protocol (CHIRALPAK® IA; *n*-Hexane/DCM/MeOH 70/25/5; UV detector wavelength 280 nm; flow rate: 0.5 mL/min; inject volume 5.0 µL).

a)

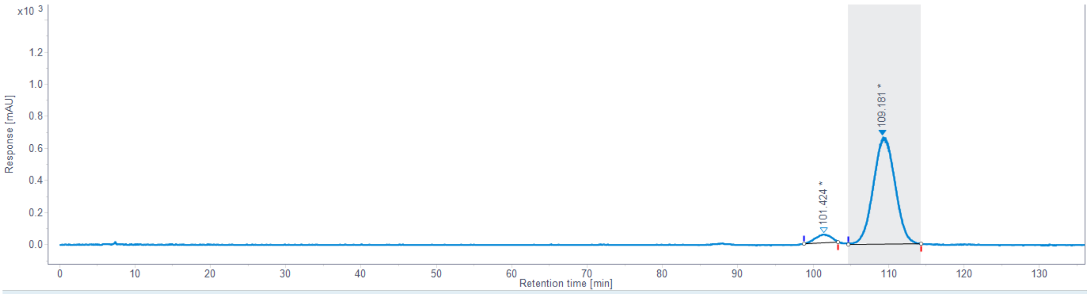

Injection Results

Peaks | Summary

#	Name	Signal description	RT (min)	Area (mAU·s)	Area%	Height (mAU)	Height%	Amount	Concentration	Start time (min)	End time (min)
1		DAD1C,Sig=280,4 Ref=off	101.424	7649.577	5.430	54.066	7.47			98.815	103.307
2		DAD1C,Sig=280,4 Ref=off	109.181	133230.964	94.570	669.866	92.53			104.706	114.273

b)

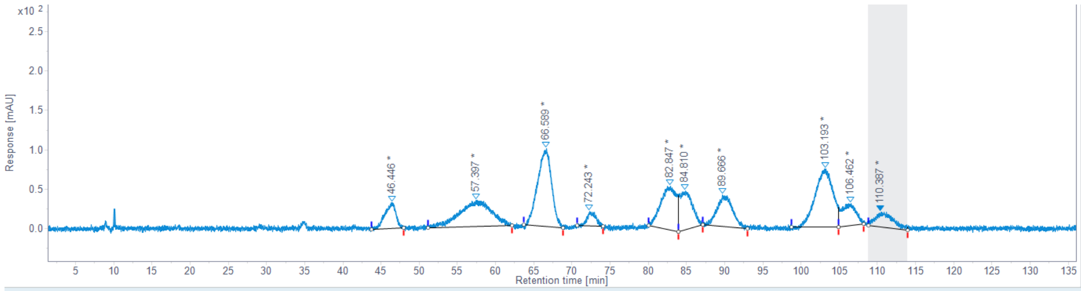

Injection Results

Peaks | Summary

#	Name	Signal description	RT (min)	Area (mAU·s)	Area%	Height (mAU)	Height%	Amount	Concentration	Start time (min)	End time (min)
4		DAD1C,Sig=280,4 Ref=off	72.243	1323.568	2.086	17.864	3.97			70.642	74.029
5		DAD1C,Sig=280,4 Ref=off	82.847	7872.139	12.405	55.749	12.40			80.039	83.934
6		DAD1C,Sig=280,4 Ref=off	84.810	5535.322	8.723	49.037	10.90			83.934	87.140
7		DAD1C,Sig=280,4 Ref=off	89.666	5430.460	8.558	40.160	8.93			87.140	92.980
8		DAD1C,Sig=280,4 Ref=off	103.193	11854.673	18.681	74.387	16.54			98.705	104.888
9		DAD1C,Sig=280,4 Ref=off	106.462	3642.975	5.741	28.572	6.35			104.888	108.153
10		DAD1C,Sig=280,4 Ref=off	110.387	3076.595	4.848	19.201	4.27			108.781	113.934

Figure S168: HPLC chromatograms of a) **(*S*, *S*, *S*, *S*)-4h** and b) **a mixture of diasteromers-4h** with optimized eluting protocol (CHIRALPAK® IA; *n*-Hexane/DCM/MeOH 80/20/5; UV detector wavelength 280 nm; flow rate: 0.5 mL/min; inject volume 5.0 μL).

a)

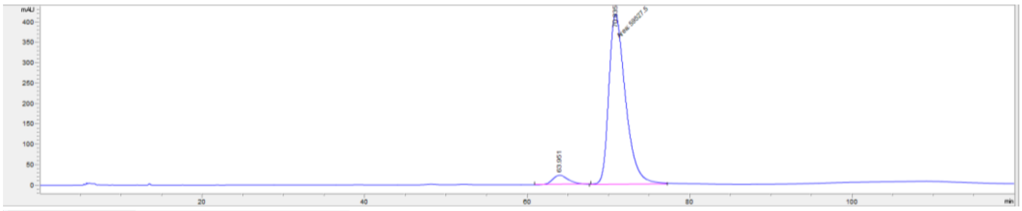

#	Time	Type	Area	Height	Width	Area%
1	63.951	BB	3060.5	22.6	1.6076	4.890
2	70.835	MM	59527.5	416.1	2.3841	95.110

b)

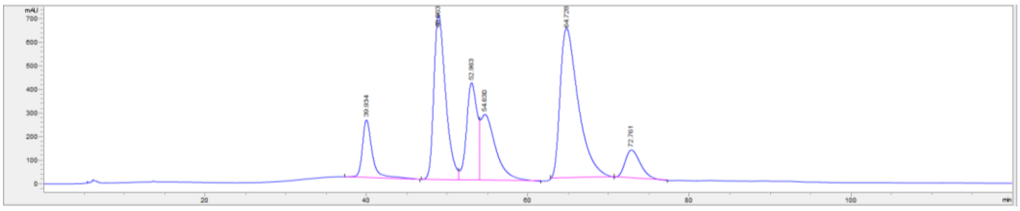

#	Time	Type	Area	Height	Width	Area%
1	39.934	BB	20650.1	243.7	1.2544	7.340
2	48.863	BV	70864.2	697.6	1.5391	25.188
3	52.963	VV	38672.5	412.4	1.4019	13.746
4	54.63	VB	36109.4	279.2	1.8448	12.835
5	64.728	BB	99346.3	634.8	2.1878	35.311
6	72.761	BB	15702.7	118.8	1.9059	5.581

Figure S169: HPLC chromatograms of a) **(*S*, *S*, *S*, *S*)-4i** and b) **a mixture of diasteromers-4i** with optimized eluting protocol (CHIRALPAK® IA; *n*-Hexane/DCM/*i*-PrOH 70/25/5; UV detector wavelength 280 nm; flow rate: 0.5 mL/min; inject volume 5.0 µL).

a)

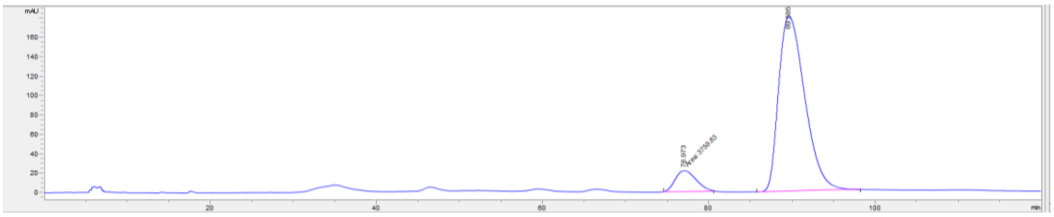

#	Time	Type	Area	Height	Width	Area%
1	76.973	MM	3759.8	21.6	2.8959	8.716
2	89.585	BB	39378.6	179.9	2.9275	91.284

b)

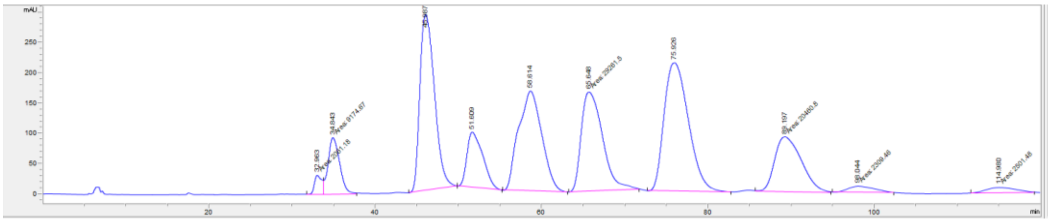

#	Time	Type	Area	Height	Width	Area%
1	32.963	MF	2061.2	32	1.0739	1.093
2	34.843	FM	9174.7	93.7	1.6319	4.865
3	45.987	BB	35502.7	289.5	1.9466	18.824
4	51.609	BB	12126.3	91.1	1.8304	6.430
5	58.614	BB	32151.9	164.8	2.572	17.048
6	65.648	MM	29261.5	163.8	2.9773	15.515
7	75.926	BB	43051.5	211.6	2.4635	22.827
8	89.197	MM	20460.8	90.9	3.7514	10.849
9	98.044	MM	2309.5	10.1	3.8101	1.225
10	114.98	MM	2501.5	9.4	4.4317	1.326

Figure S170: HPLC chromatograms of a) **(*S*, *S*, *S*, *S*)-4j** and b) **a mixture of diasteromers-4j** with optimized eluting protocol (CHIRALPAK® IA; *n*-Hexane/DCM/*i*-PrOH 75/20/5; UV detector wavelength 280 nm; flow rate: 0.5 mL/min; inject volume 5.0 µL).

5. ECD spectra

a)

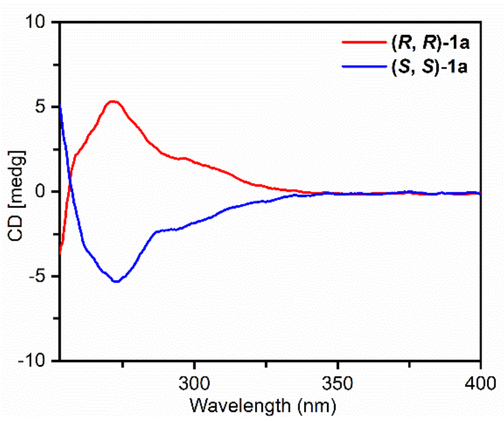

b)

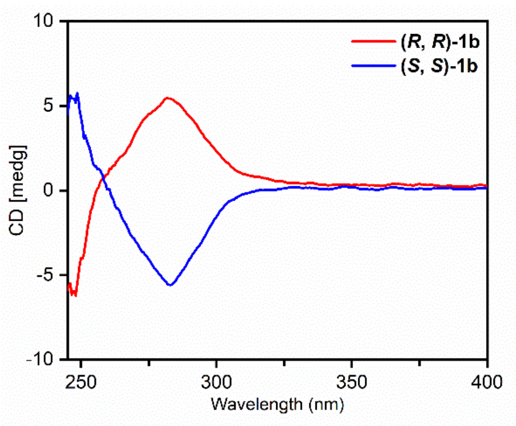

c)

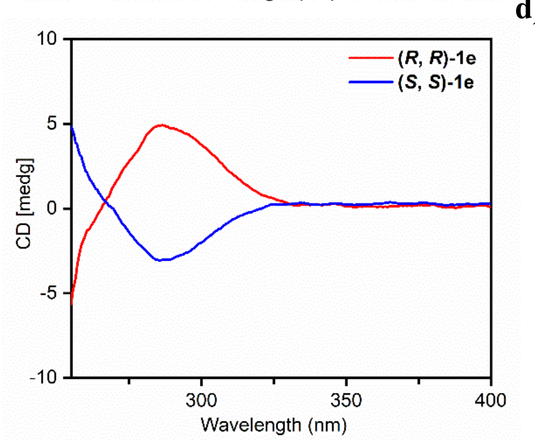

d)

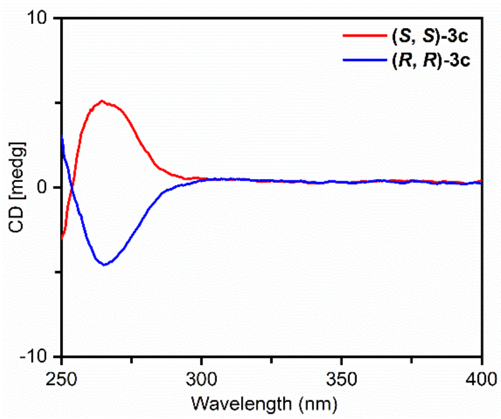

e)

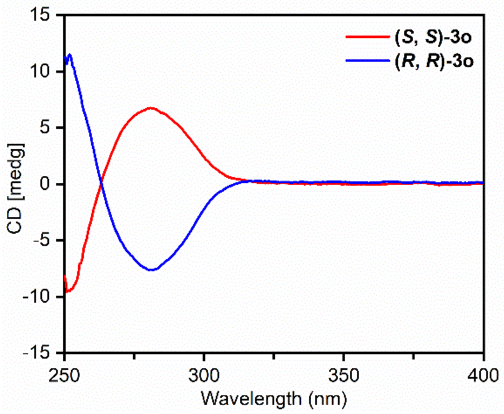

f)

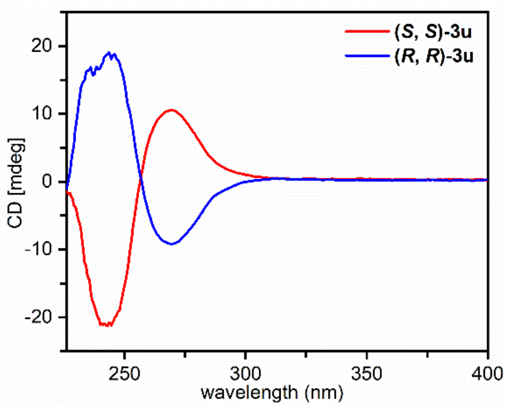

g)

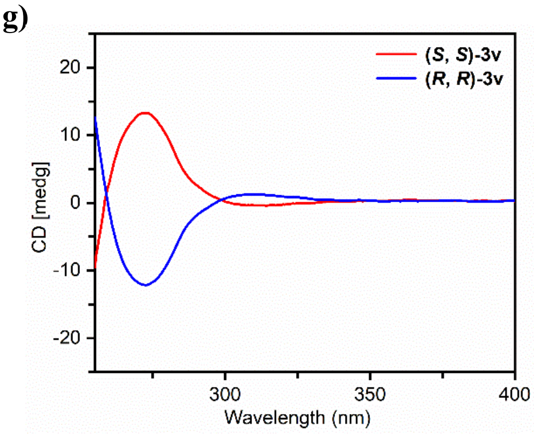

h)

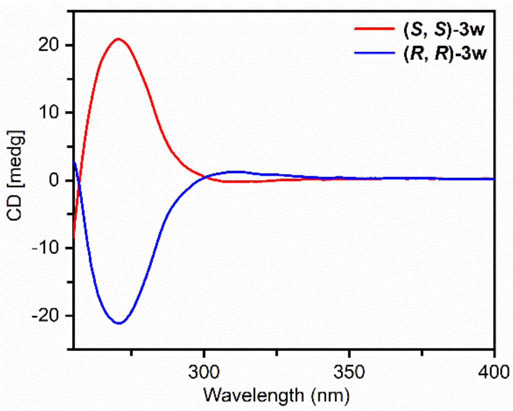

i)

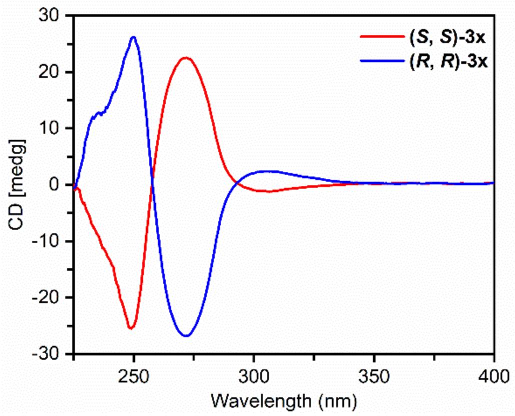

j)

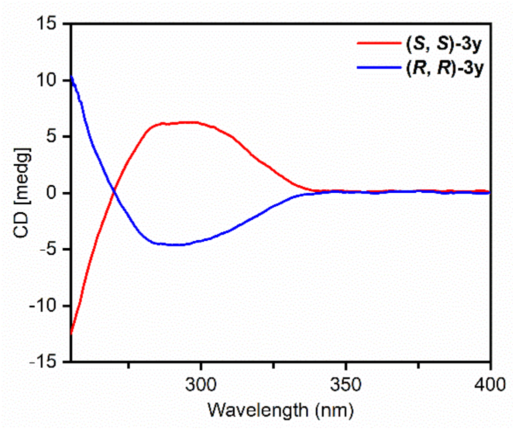

k)

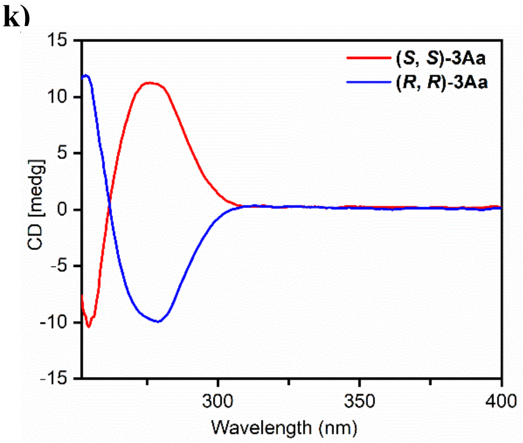

l)

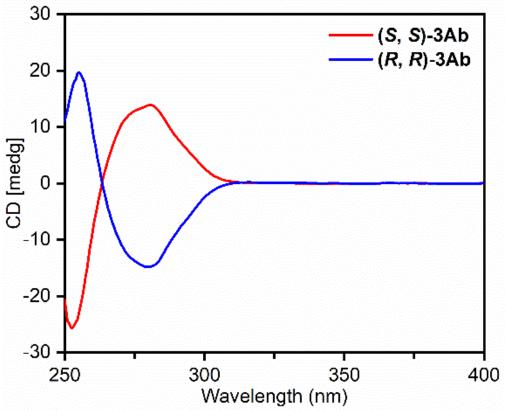

m)

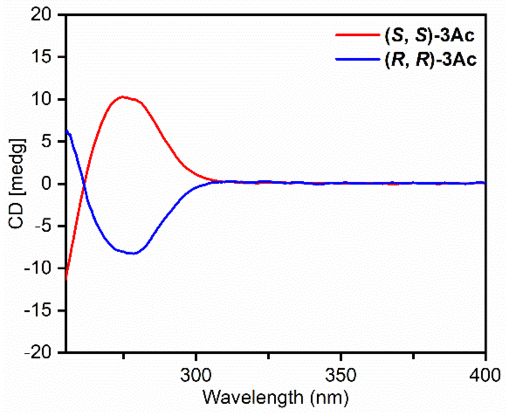

n)

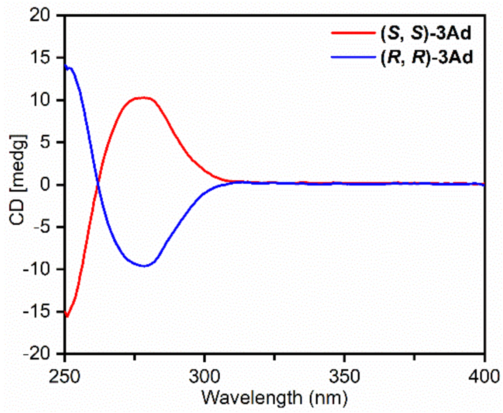

Figure S171. ECD spectra of both enantiomers: Reactants: a) ***(R, R)*-1a** and ***(S, S)*-1a**, b) ***(R, R)*-1b** and ***(S, S)*-1b**, c) ***(R, R)*-1e** and ***(S, S)*-1e**. Macrocycles: d) ***(R, R)*-3c** and ***(S, S)*-3c**, e) ***(R, R)*-3o** and ***(S, S)*-3o**, f) ***(R, R)*-3u** and ***(S, S)*-3u**, g) ***(R, R)*-3v** and ***(S, S)*-3v**, h) ***(R, R)*-3w** and ***(S, S)*-3w**, i) ***(R, R)*-3x** and ***(S, S)*-3x**, j) ***(R, R)*-3y** and ***(S, S)*-3y**, k) ***(R, R)*-3Aa** and ***(S, S)*- 3Aa** , l) ***(R, R)*-3Ab** and ***(S, S)*- 3Ab** , m) ***(R, R)*-3Ac** and ***(S, S)*-3Ac**, n) ***(R, R)*-3Ad** and ***(S, S)*-3Ad**.

6. Chiral macrocycles based on SuPhenEx reaction

6.1 Synthesis of enantiomer di-*p*-nitrophenolate sulfonimidate

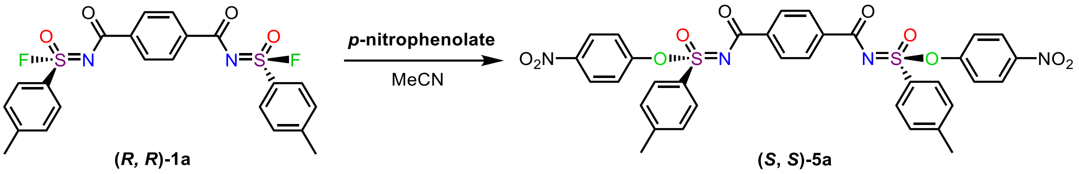

(*S*, *S*)-5a: **(*R*, *R*)-1a** (1.2 g, 2.1 mmol) was dissolved in anhydrous acetonitrile (10 mL) under argon protection. *p*-nitrophenolate (0.7 g, 4.4 mmol, 2.1 equiv) was added to the solution. The reaction mixture was stirred for 2 h at 30 °C. The reaction was quenched by adding water (10 mL), extracted with CH_2Cl_2 (3 × 20 mL), dried with anhydrous $MgSO_4$, concentrated by reduced pressure. The resulting residual was purified by silica gel column chromatography (*n*-hexane/EtOAc = 4:1 to 2:1), and recrystallization with *n*-hexane and ethyl acetate to afford **(*S*, *S*)-5a** as a white solid (1.3 mmol, 0.93 g, 62%, >99% *ee*, *dr* = 82:1) ^{1}H NMR (400 MHz, $CDCl_3$) δ 8.21 (d, *J* = 9.1 Hz, 4H), 8.16 (s, 4H), 7.98 (d, *J* = 8.4 Hz, 4H), 7.44 (d, *J* = 8.1 Hz, 4H), 7.33 (d, *J* = 9.2 Hz, 4H), 2.51 (s, 6H). ^{13}C{1H} NMR (101 MHz, $CDCl_3$) δ 170.8 (s), 153.8 (s), 146.6 (s), 132.8 (s), 130.3 (s), 129.6 (s), 128.1 (s), 125.4 (s), 123.8 (s), 53.4 (s), 21.8 (s). HRMS (ESI) *m/z* $[M + H]^+$ Calcd for $C_{34}H_{27}N_4O_{10}S_2$ 715.1163, found 715.1141.

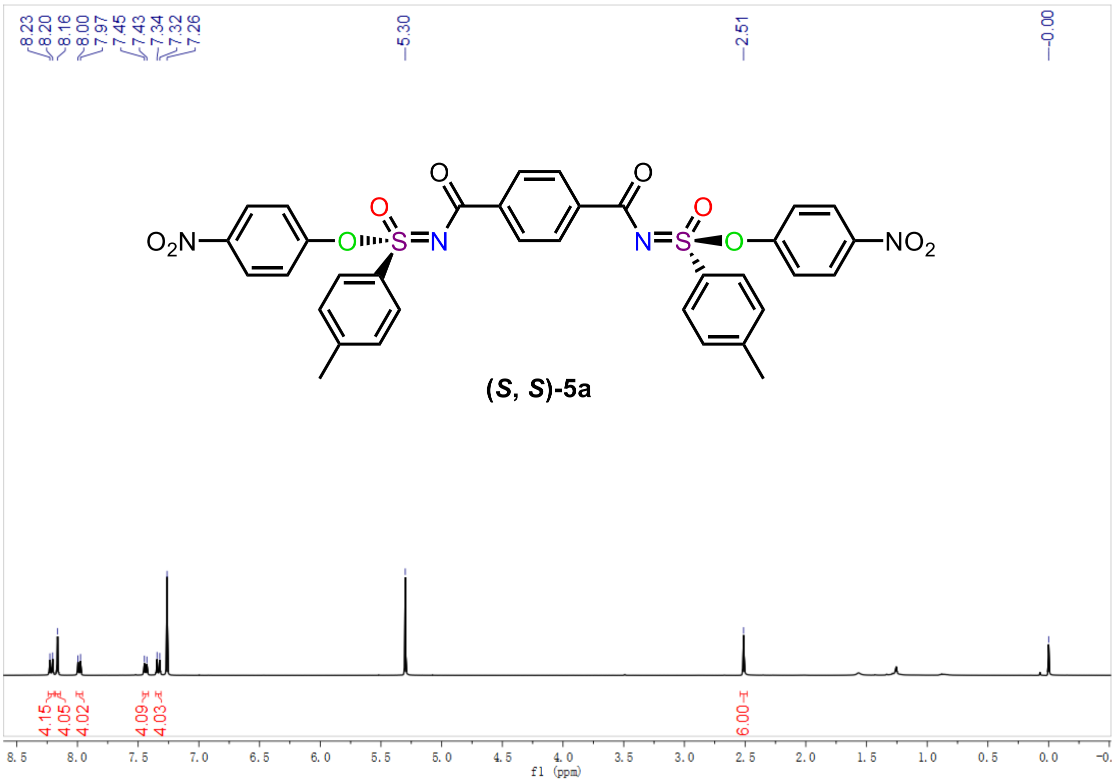

Figure S172. ^{1}H NMR (400 MHz) spectra of compound **(*S*, *S*)-5a** ($CDCl_3$, 298 K).

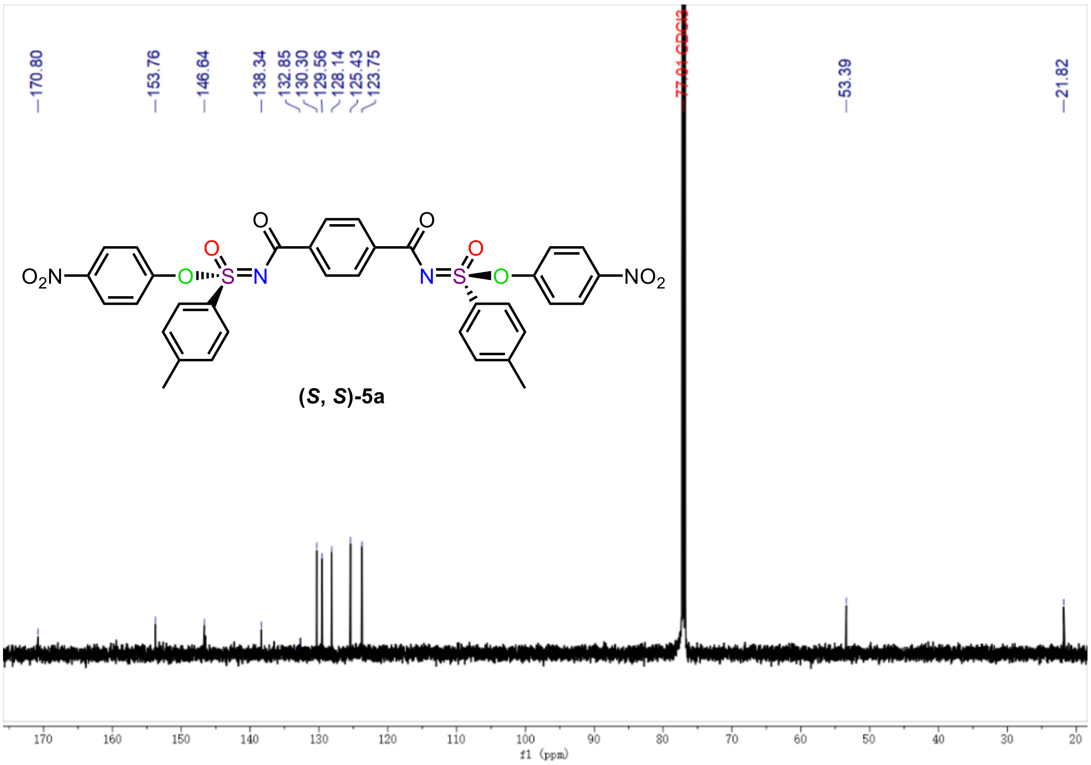

Figure S173. ^{13}C{1H} NMR (101 MHz) spectra of compound **(*S, S*)-5a** ($CDCl_3$, 298 K).

a)

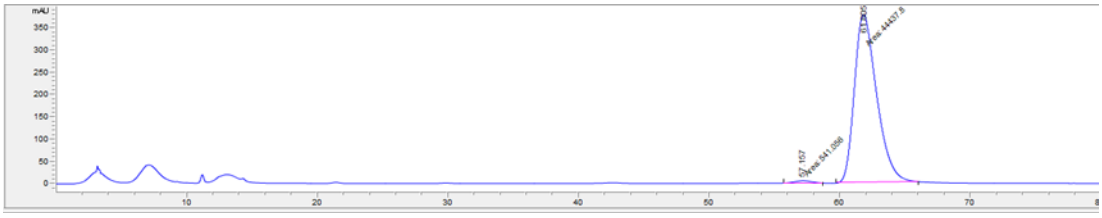

#	Time	Type	Area	Height	Width	Area%
1	57.157	MM	541.1	5.5	1.6296	1.203
2	61.805	MM	44437.8	376.1	1.9691	98.797

b)

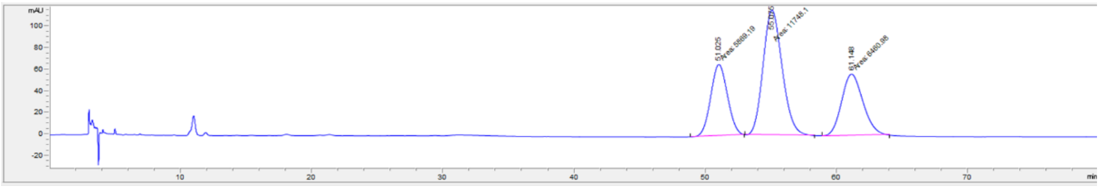

#	Time	Type	Area	Height	Width	Area%
1	51.025	MM	5869.2	66.1	1.4801	24.376
2	55.075	MM	11748.1	115.8	1.6913	48.791
3	61.148	MM	6461	56.9	1.8925	26.833

Figure S174: HPLC chromatograms of a) **(*S, S*)-5a** and b) **Racemic-5a** with optimized eluting protocol (CHIRALPAK® IE; *n*-Hexane/DCM/*i*-PrOH 65/20/15; flow rate: 1.0 mL/min; UV detector wavelength 254 nm; oven temperature 40 °C; inject volume 5.0 µL).

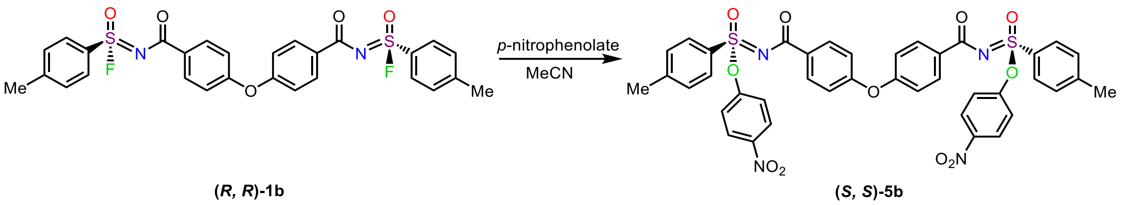

(*S*, *S*)-5b: **(*R*, *R*)-1b** (800 mg, 1.7 mmol) was dissolved in anhydrous acetonitrile (10 mL) under argon protection. *p*-nitrophenolate (576 mg, 3.6 mmol, 2.1 equiv) was added to the solution. The reaction mixture was stirred for 2 h at 30 ºC. The reaction was quenched by adding water (10 mL), extracted with CH_2Cl_2 (3 × 20 mL), dried with anhydrous $MgSO_4$, concentrated by reduced pressure. The resulting residual was purified by silica gel column chromatography (*n*-hexane/EtOAc = 4:1 to 2:1), and recrystallization with *n*-hexane and ethyl acetate to afford **(*S*, *S*)-5b** as a white solid (0.7 mmol, 0.62 g, 45%, 99% *ee*) ^{1}H NMR (400 MHz, $CDCl_3$) δ 8.21 (d, *J* = 9.1 Hz, 4H), 8.14 (d, *J* = 8.8 Hz, 4H), 7.98 (d, *J* = 8.4 Hz, 4H), 7.43 (d, *J* = 8.1 Hz, 4H), 7.33 (d, *J* = 9.2 Hz, 4H), 7.05 (d, *J* = 8.8 Hz, 4H), 2.51 (s, 6H). ^{13}C{1H} NMR (101 MHz, $CDCl_3$) δ 170.7 (s), 160.3 (s), 153.8 (s), 146.6 (s), 146.4 (s), 132.7 (s), 132.1 (s), 130.3 (s), 128.1 (s), 125.4 (s), 123.8 (s), 118.5 (s), 21.9 (s). HRMS (ESI) *m/z* [*M* + H]$^+$ Calcd for $C_{40}H_{31}N_4O_{11}S_2$ 807.1425, found 807.1395.

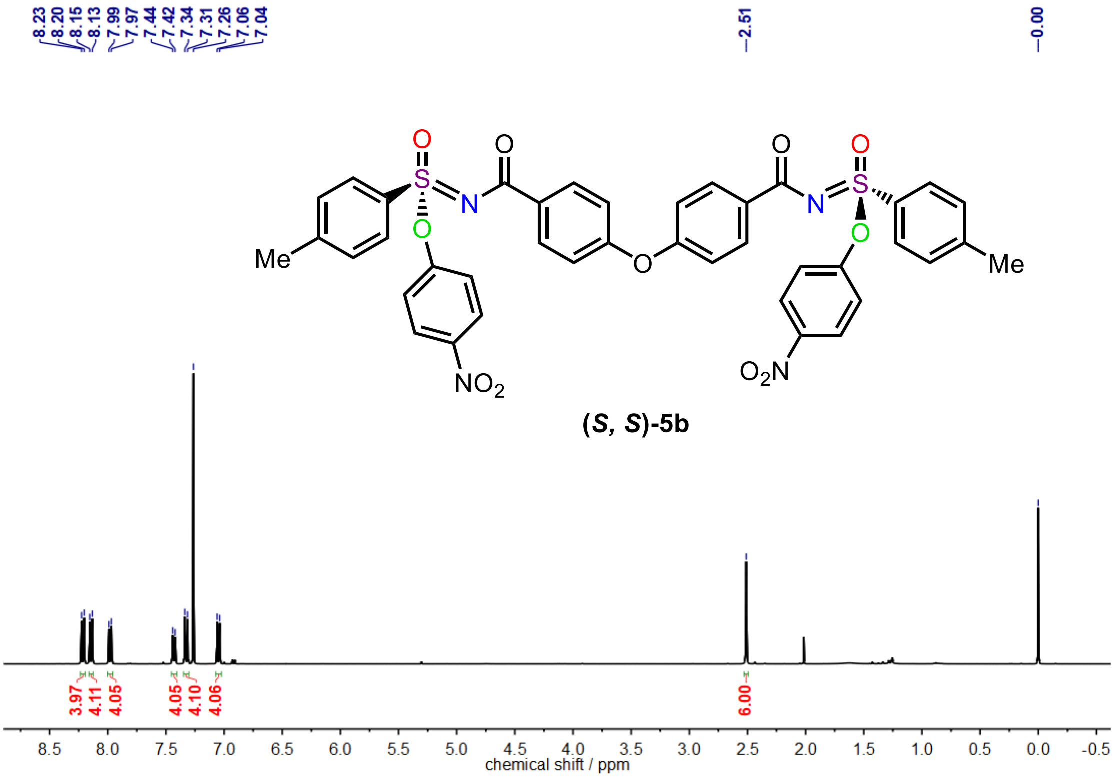

Figure S175. ^{1}H NMR (400 MHz) spectra of compound **(*S*, *S*)-5b** ($CDCl_3$, 298 K).

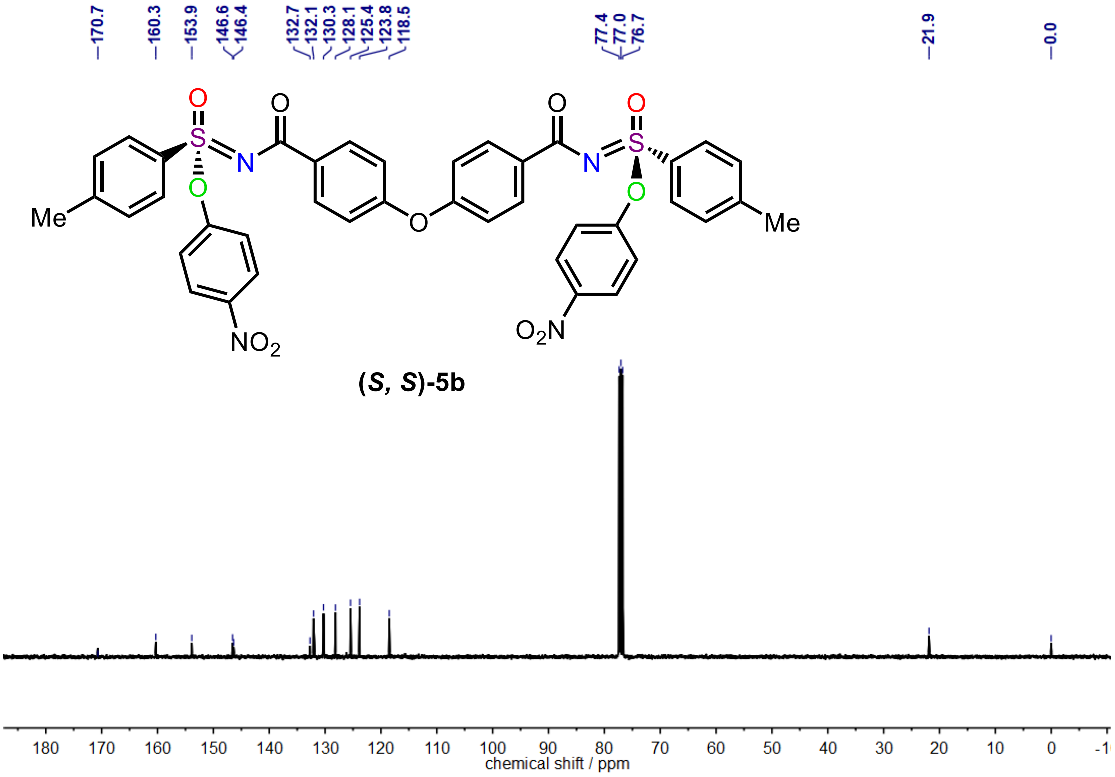

Figure S176. $^{13}C\{1H\}$ NMR (101 MHz) spectra of compound ***(S, S)*-5b** ($CDCl_3$, 298 K).

a)

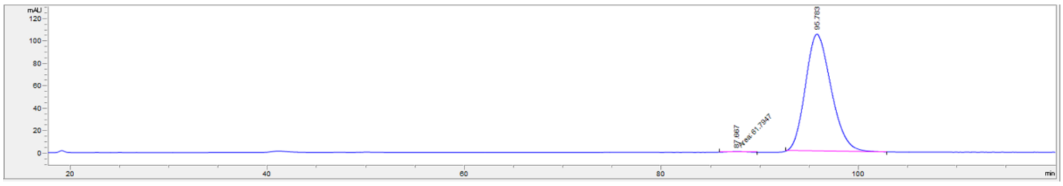

#	Time	Type	Area	Height	Width	Area%
1	87.667	MM	61.8	5.3E-1	1.928	0.323
2	95.783	BB	19052	105	2.3977	99.677

b)

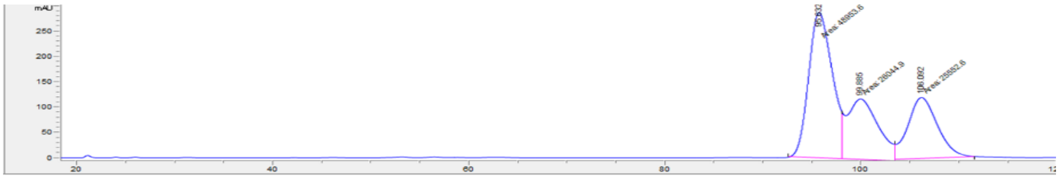

#	Time	Type	Area	Height	Width	Area%
1	95.632	MM	48953.6	288.5	2.8283	48.685
2	99.885	MM	26044.9	119.9	3.6213	25.902
3	106.092	MM	25552.6	121.3	3.5118	25.413

Figure S177: HPLC chromatograms of a) ***(S, S)*-5b** and b) **Racemic-5b** with optimized eluting protocol (CHIRALPAK® IE; *n*-Hexane/DCM/*i*-PrOH 65/20/15; flow rate: 1.0 mL/min; UV detector wavelength 254 nm; oven temperature 40 °C; inject volume 5.0 μL).

6.2 Synthesis of chiral macrocycles by SuPhenEx reaction

General procedure:

To a seal tube (75 mL) with a magnetic stirring bar, was added **(*S*, *S*)-5** (0.25 mmol, 1.0 equiv) and 1.0 equiv of sodium diphenolate (prepared according to a published procedure)[2]. Next 50 mL of anhydrous acetonitrile and 15-crown-5-ether (0.5 mmol, 2.0 equiv) were added into the seal tube. The reaction mixture was allowed to stir for 30 min to 5 h at 50/60 ºC. Then the reaction was quenched with 50 mL water. The solution was extracted with CH_2Cl_2 (3 × 200 mL), dried with anhydrous Na_2SO_4 and concentrated by reduced pressure. The resulting residual was purified by silica gel column chromatography (*n*-hexane/EtOAc = 4:1 to 3:1) to afford **(*R*, *R*)-4** as a white solid.

Table S2. the synthesis of chiral macrocycles by SuPhenEx reaction.

compound	time	Temp (ºC)	Yield (%)	*ee* (%)	*es* (%)
(*R*, *R*)-3c	4.5 h	50	0.11 mmol, 45%	92	96
(*R*, *R*)-3w	3.5 h	50	0.13 mmol, 54%	96	99
(*R*, *R*)-3x	0.5 h	50	0.12 mmol, 50%	98	>99
(*R*, *R*)-3o	5 h	60	0.08 mmol, 55%	96	98

es=enantiospecificity, given by % of (*R*)-stereocenters in **3**/ % of (*S*)-stereocenters in **5**%.

(*R*, *R*)-3c was obtained as a white solid (88 mg, 0.11 mmol, 45%, 96% *es*). ^{1}H NMR (400 MHz, $CDCl_3$) δ 7.95 (d, *J* = 8.4 Hz, 4H), 7.78 (s, 4H), 7.40 (d, *J* = 8.2 Hz, 4H), 7.21 (d, *J* = 3.9 Hz, 1H), 7.10 – 7.07 (m, 6H), 6.98 – 6.95 (m, 4H), 6.89 (t, *J* = 1.9 Hz, 1H), 2.49 (s, 6H), 1.56 (s, 12H).

(*R*, *R*)-3w was obtained as a white solid (74 mg, 0.13 mmol, 54%, 99% *es*). ^{1}H NMR (400 MHz, $CDCl_3$) δ 7.98 (d, *J* = 8.2 Hz, 4H), 7.67 (s, 4H), 7.46 (d, *J* = 8.2 Hz, 4H), 7.14 (d, *J* = 8.6 Hz, 4H), 6.93 (d, *J* = 8.5 Hz, 4H), 2.53 (s, 6H), 1.61 (s, 6H).

(*R*, *R*)-3x was obtained as a white solid (96 mg, 0.12 mmol, 50%, >99% *es*). ^{1}H NMR (400 MHz, $CDCl_3$) δ 7.99 (s, 4H), 7.97 (d, *J* = 8.4 Hz, 4H), 7.48 (d, *J* = 7.9 Hz, 4H), 7.02 (d, *J* = 8.5 Hz, 4H), 6.83 (d, *J* = 9.0 Hz, 4H), 2.55 (s, 6H). ^{19}F NMR (376 MHz, $CDCl_3$) δ -63.80.

(*R*, *R*)-3o was obtained as a white solid (72 mg, 0.08 mmol, 55%, 98% *es*). ^{1}H NMR (400 MHz, CD_2Cl_2) δ 7.90 (d, *J* = 8.4 Hz, 4H), 7.76 (d, *J* = 8.8 Hz, 4H), 7.36 (d, *J* = 8.1 Hz, 4H), 7.23 (s, 1H), 7.16 (d, *J* = 8.8 Hz, 1H), 6.99 (d, *J* = 8.8 Hz, 4H), 6.83 (d, *J* = 8.8 Hz, 4H), 6.72 (t, *J* = 7.7 Hz, 1H), 6.48 (dd, *J* = 7.6, 1.8 Hz, 2H), 2.40 (s, 6H), 1.51 (d, *J* = 15.2 Hz, 12H).

6.2.1 NMR spectra

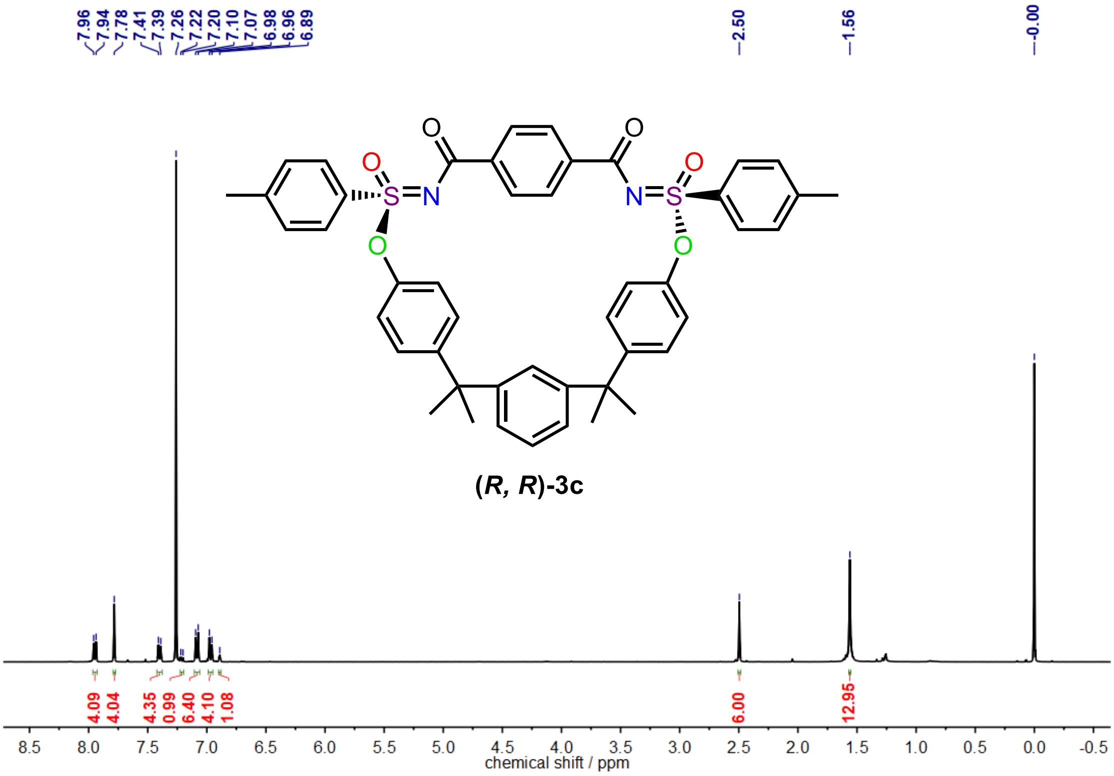

Figure S178. ^{1}H NMR (400 MHz) spectra of compound **(*R*, *R*)-3c** ($CDCl_3$, 298 K).

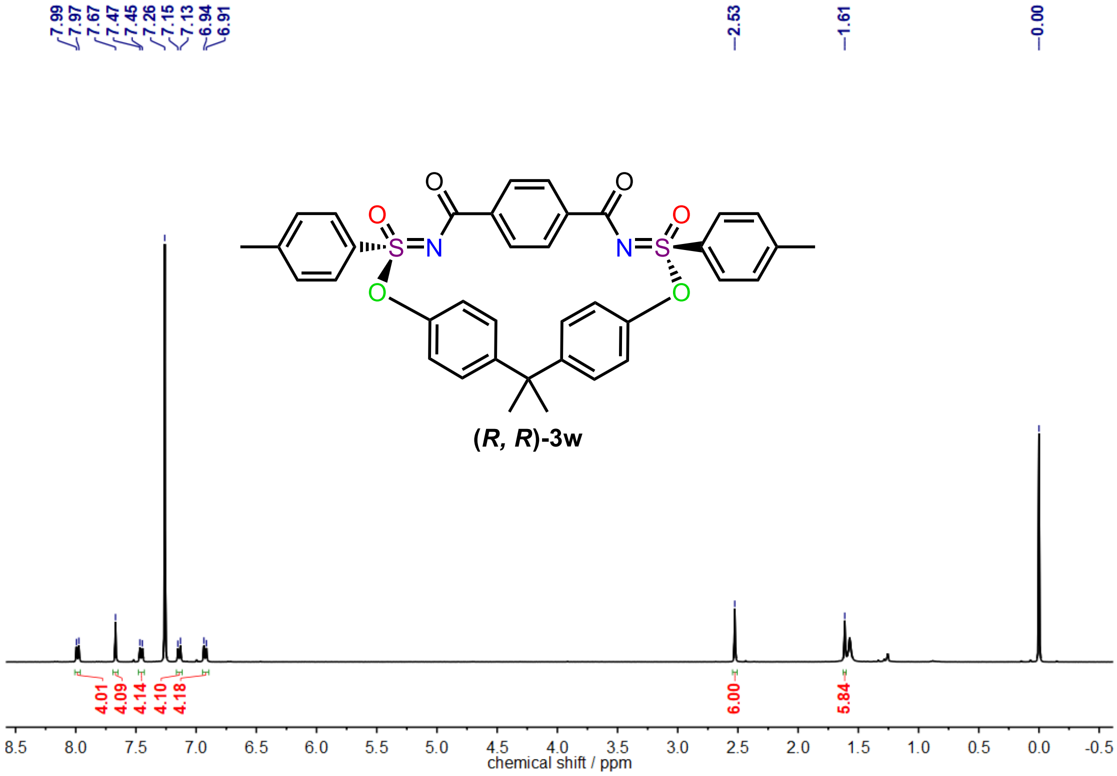

Figure S179. ^{1}H NMR (400 MHz) spectra of compound **(*R*, *R*)-3w** ($CDCl_3$, 298 K).

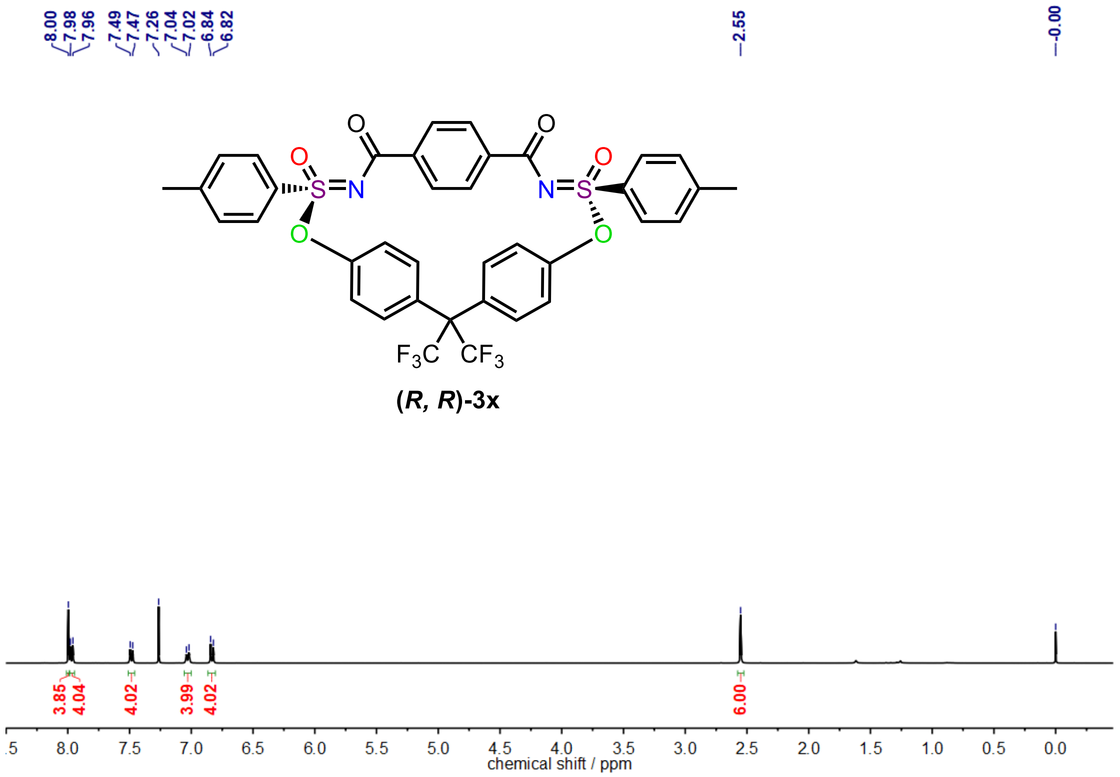

Figure S180. ^{1}H NMR (400 MHz) spectra of compound **(*R*, *R*)-3x** ($CDCl_3$, 298 K).

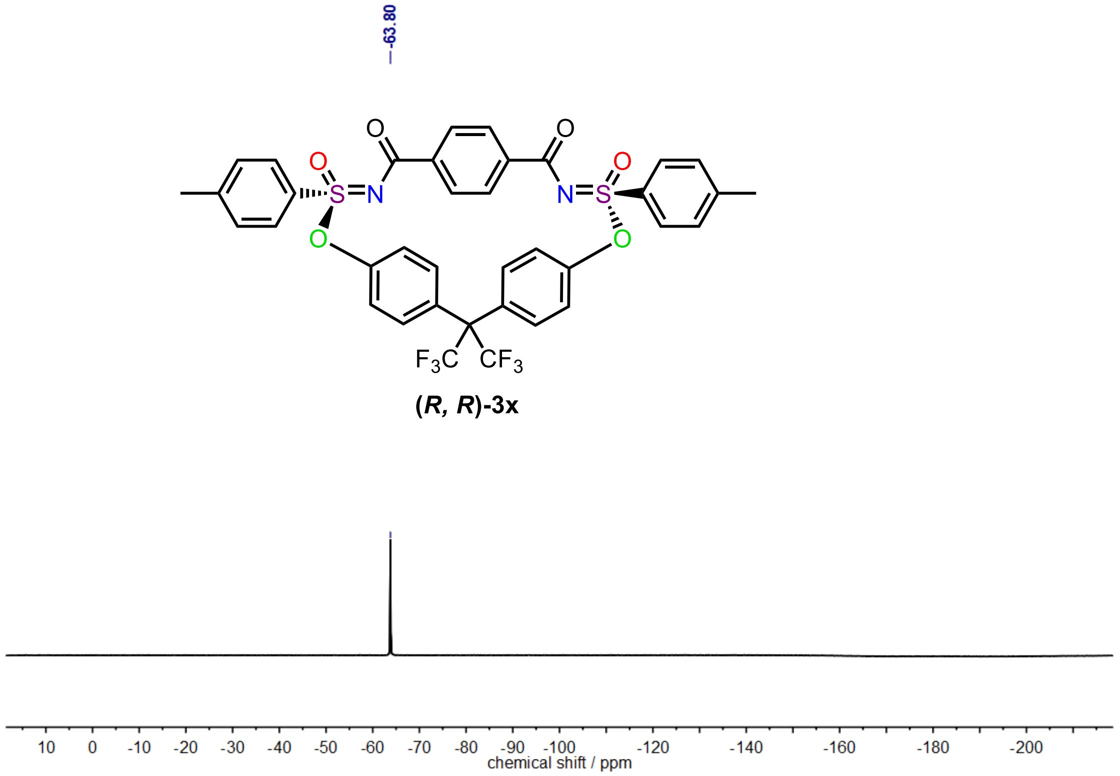

Figure S181. ^{19}F NMR (376 MHz) spectra of compound **(*R*, *R*)-3x** ($CDCl_3$, 298 K).

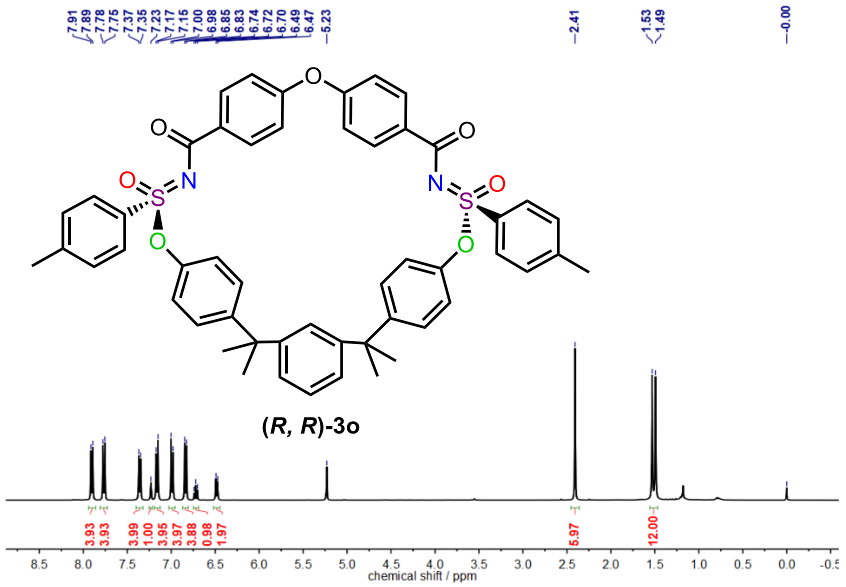

Figure S182. ^{1}H NMR (400 MHz) spectra of compound **(*R*, *R*)-3o** ($CDCl_3$, 298 K).

6.2.2 ECD spectra

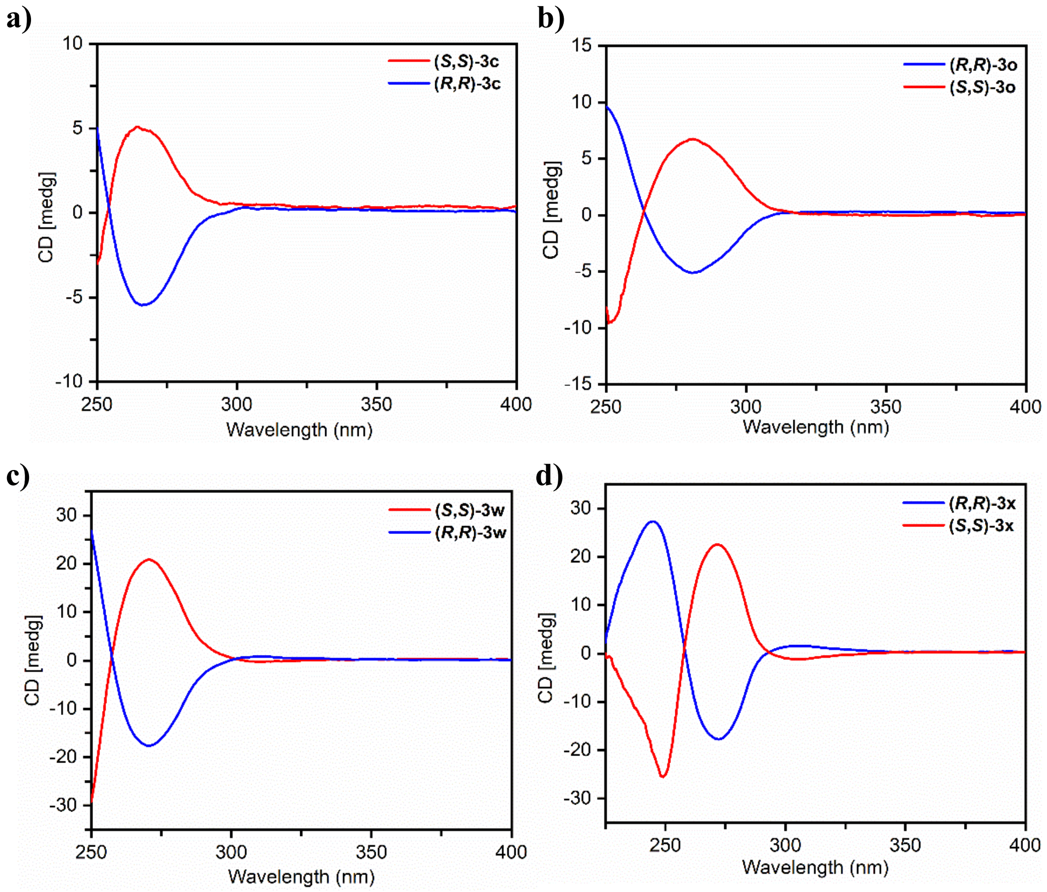

Figure S183. ECD spectra of sets of enantiomers: a) **(*S*, *S*)-3c** and **(*R*, *R*)-3c**, b) **(*S*, *S*)-3o** and **(*R*, *R*)-3o**, c) **(*S*, *S*)-3w** and **(*R*, *R*)-3w**, d) **(*S*, *S*)-3x** and **(*R*, *R*)-3x**. ((*S,S)* configuration from SuFEx reaction and (*R,R)* configuration from SuFEx followed by SuPhenEx reaction).

6.2.3 HPLC spectra

a)

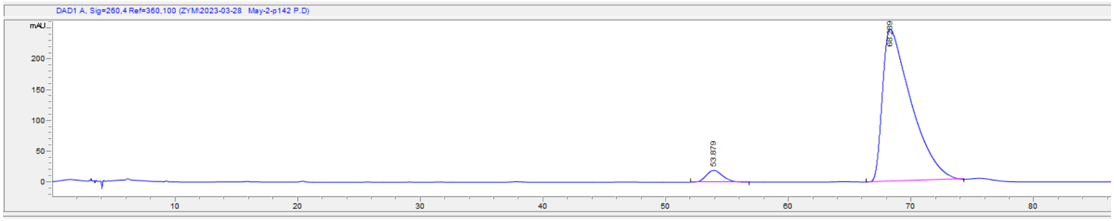

#	Time	Type	Area	Height	Width	Area%
1	53.879	BB	1774.8	19.1	1.2608	4.227
2	68.289	BB	40208.1	247.1	2.2646	95.773

b)

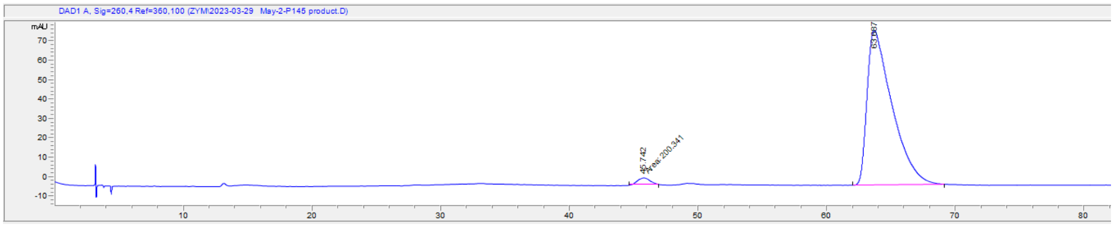

#	Time	Type	Area	Height	Width	Area%
1	45.742	MM	200.3	3.2	1.0427	1.833
2	63.687	BB	10729.4	79.5	1.8531	98.167

c)

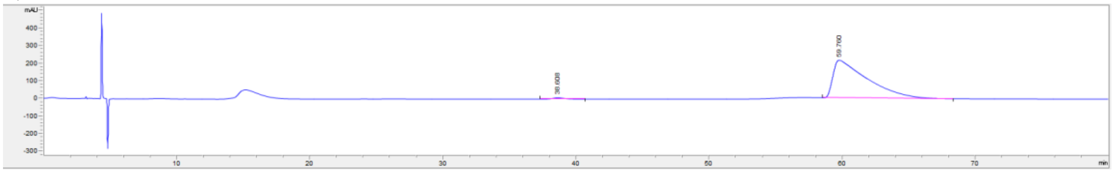

#	Time	Type	Area	Height	Width	Area%
1	38.608	BB	418.4	6.9	0.8494	1.081
2	59.76	BB	38283	213.8	2.3313	98.919

d)

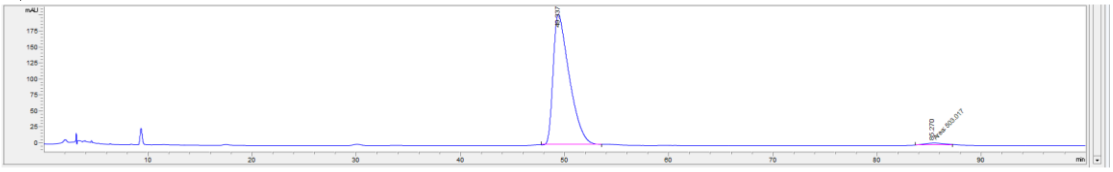

#	Time	Type	Area	Height	Width	Area%
1	49.337	BB	22070.5	205.2	1.5745	97.772
2	85.27	MM	503	3.5	1.6823	2.228

Figure S184: HPLC chromatograms of a) **(*R*, *R*)-3c**, b) **(*R*, *R*)-3w**, c) **(*R*, *R*)-3x**, and d) **(*R*, *R*)-3o** with optimized eluting protocol (CHIRALPAK® IE; *n*-Hexane/DCM/*i*-PrOH 70/20/10 for **(*R*, *R*)-3c**, 60/35/5 for **(*R*, *R*)-3w**, 70/25/5 for **(*R*, *R*)-3x** and 55/40/5 for **(*R*, *R*)-3o** ; flow rate: 1.0 mL/min; UV detector wavelength 260 nm; oven temperature 40 ℃; inject volume 5.0 μL).

7. X-Ray crystallography

Single crystals of **(*R*, *R*)-1a**, **(*R*, *R*)-1b**, **(*R*, *R*)-1e, (*S*, *S*)-3a, meso-3a, (*S*, *S*)-3c, (*R*, *R*)-3c, (*S*, *S*)-3o, meso-3u, (*S*, *S*)-3w, (*R*, *R*)-3w** and **(*S*, *S*)-3y** that were deemed suitable for X-ray diffraction were selected and mounted in inert oil under a cold gas stream, and their X-ray diffraction intensity data were collected on a Rigaku XtaLAB FRX diffractometer equipped with the Hypix6000HE detector, using Cu *Kα* radiation (λ = 1.54184 Å) and Mo *Kα* radiation (λ = 0.71073 Å). The crystal was kept at the temperature listed in **Table S3-S14** during data collection. By the use of Olex2[4], the structure was solved either (i) with the ShelXS[5] structure solution program using Direct Methods, or (ii) with the ShelXT[6] structure solution program using Direct Methods or Intrinsic Phasing, and (iii) refined with the ShelXT refinement package using Least Squares minimization. The hydrogen atoms were set in calculated positions and refined as riding atoms with a common fixed isotropic thermal parameter. Some guest molecules were refined isotropically due to disorder that could not be modeled precisely. Distance restraints were also imposed on some disordered guest hexane molecules. Selected details of the data collection and structural refinement of each compound can be found within **Table S3–S14** and full details are available in the corresponding CIF files.

Note: All crystals were obtained by vapor diffusion of *n*-hexane into an ethyl acetate or chloroform solution of compounds, but the crystals of **(*S*, *S*)-3w** and **(*R*, *R*)-3w** were obtained by slow evaporation of a mixed solution of **(*S*, *S*)-3w** and **(*R*, *R*)-3w** in dichloromethane and methanol, respectively.

Table S3. Crystal data and structure refinement for **(*R*, *R*)-1a**.

Crystallization Solvent	ethyl acetate / *n*-hexane
Empirical formula	$C_{22}H_{18}F_2N_2O_4S_2$
Formula weight	476.50
Temperature / K	160.00(10)
Crystal system	triclinic
Space group	*P*1
a / Å	6.69340(10)
b / Å	7.13570(10)
c / Å	11.2613(2)
α / °	96.495(2)
β / °	99.665(2)
γ / °	90.218(2)
Volume / Å^3	526.683(15)
Z	1
ρ_{calc} / g cm^{-3}	1.502
μ / mm^{-1}	2.751
F (000)	246.0
Crystal size / mm^3	0.2 × 0.05 × 0.05
Radiation	Cu *Kα* (*λ* = 1.54184)
2θ range for data collection / °	8.018 to 152.926
Index ranges	$-8 \le h \le 8$, $-8 \le k \le 8$, $-13 \le l \le 13$
Reflections collected	12835
Independent reflections	3925 [R_{int} = 0.0306, R_{sigma} = 0.0291]
Data/restraints/parameters	3925/3/291
Goodness-of-fit on F^2	1.069
Final *R* indexes [I>=2σ (I)]	R_1 = 0.0329, wR_2 = 0.0881
Final *R* indexes [all data]	R_1 = 0.0341, wR_2 = 0.0894
Largest diff. peak / hole / e Å^{-3}	0.27/-0.39
Flack parameter	0.010(10)
CCDC number	2265337

Table S4. Crystal data and structure refinement for ***(R, R)*-1b**.

Crystallization Solvent	ethyl acetate / *n*-hexane
Empirical formula	$C_{28}H_{22}F_2N_2O_5S_2$
Formula weight	568.59
Temperature / K	159.99(10)
Crystal system	orthorhombic
Space group	$P2_12_12_1$
a / Å	5.4295(4)
b / Å	19.3343(16)
c / Å	24.3639(16)
α / °	90
β / °	90
γ / °	90
Volume / Å^3	2557.6(3)
Z	4
ρ_{calc} / g cm^{-3}	1.477
μ / mm^{-1}	2.398
F (000)	1176.0
Crystal size / mm^3	0.2 × 0.02 × 0.02
Radiation	Cu $K\alpha$ (λ = 1.54184)
2θ range for data collection / °	5.836 to 127.998
Index ranges	$-6 \leq h \leq 5$, $-21 \leq k \leq 22$, $-28 \leq l \leq 27$
Reflections collected	9784
Independent reflections	3982 [R_{int} = 0.0834, R_{sigma} = 0.1083]
Data/restraints/parameters	3982/0/354
Goodness-of-fit on F^2	0.964
Final R indexes [I>=2σ (I)]	R_1 = 0.0484, wR_2 = 0.1034
Final R indexes [all data]	R_1 = 0.0935, wR_2 = 0.1210
Largest diff. peak / hole / e Å^{-3}	0.31/-0.28
Flack parameter	0.08(2)
CCDC number	2265339

Table S5. Crystal data and structure refinement for ***(R, R)*-1e**.

Crystallization Solvent	ethyl acetate / *n*-hexane
Empirical formula	$C_{28}H_{22}F_2N_2O_4S_2$
Formula weight	552.59
Temperature / K	160.00(10)
Crystal system	monoclinic
Space group	$P2_1$
a / Å	5.10460(10)
b / Å	13.6147(2)
c / Å	18.0522(3)
α / °	90
β / °	96.0340(10)
γ / °	90
Volume / Å^3	1247.63(4)
Z	2
ρ_{calc} / g cm^{-3}	1.471
μ / mm^{-1}	2.409
F (000)	572.0
Crystal size / mm^3	0.2 × 0.05 × 0.05
Radiation	Cu $K\alpha$ (λ = 1.54184)
2θ range for data collection / °	4.922 to 152.428
Index ranges	$-5 \leq h \leq 6$, $-16 \leq k \leq 17$, $-22 \leq l \leq 22$
Reflections collected	14466
Independent reflections	4940 [R_{int} = 0.0381, R_{sigma} = 0.0393]
Data/restraints/parameters	4940/1/345
Goodness-of-fit on F^2	1.073
Final R indexes [I>=2σ (I)]	R_1 = 0.0379, wR_2 = 0.1009
Final R indexes [all data]	R_1 = 0.0411, wR_2 = 0.1028
Largest diff. peak / hole / e Å^{-3}	0.24/-0.38
Flack parameter	0.021(10)
CCDC number	2265338

Table S6. Crystal data and structure refinement for ***(S, S)*-3a**.

Crystallization Solvent	ethyl acetate / *n*-hexane
Empirical formula	$C_{34}H_{26}N_2O_6S_4$
Formula weight	686.81
Temperature / K	284.(2)
Crystal system	orthorhombic
Space group	$P2_12_12_1$
a / Å	5.6690(4)
b / Å	13.4413(8)
c / Å	41.656(2)
α / °	90
β / °	90
γ / °	90
Volume / Å^3	3174.1(3)
Z	4
ρ_{calc} / g cm^{-3}	1.437
μ / mm^{-1}	0.349
F (000)	1424.0
Crystal size / mm^3	0.467 × 0.019 × 0.011
Radiation	Mo-$K\alpha$ (λ = 0.71073)
2θ range for data collection / °	3.18 to 50.7
Index ranges	$-5 \leq h \leq 6$, $-15 \leq k \leq 16$, $-50 \leq l \leq 47$
Reflections collected	26956
Independent reflections	5814 [R_{int} = 0.0893, R_{sigma} = 0.1052]
Data/restraints/parameters	5814/0/417
Goodness-of-fit on F^2	0.964
Final R indexes [I>=2σ (I)]	R_1 = 0.0485, wR_2 = 0.0756
Final R indexes [all data]	R_1 = 0.1206, wR_2 = 0.0905
Largest diff. peak / hole / e Å^{-3}	0.18/-0.19
Flack parameter	0.11(6)
CCDC number	2263049

Table S7. Crystal data and structure refinement for **meso-3a**.

Crystallization Solvent	ethyl acetate / *n*-hexane
Empirical formula	$C_{34}H_{26}N_2O_6S_4$
Formula weight	686.81
Temperature / K	284.(2)
Crystal system	triclinic
Space group	$P\bar{1}$
a / Å	6.1342(4)
b / Å	9.1404(6)
c / Å	29.3504(18)
α / °	89.988(2)
β / °	86.177(2)
γ / °	78.928(2)
Volume / Å^3	1611.29(18)
Z	2
ρ_{calc} / g cm^{-3}	1.416
μ / mm^{-1}	0.344
F (000)	712.0
Crystal size / mm^3	0.216 × 0.189 × 0.122
Radiation	Mo-$K\alpha$ (λ = 0.71073)
2θ range for data collection / °	4.18 to 56.56
Index ranges	$-8 \leq h \leq 8$, $-12 \leq k \leq 12$, $-35 \leq l \leq 39$
Reflections collected	42662
Independent reflections	7944 [R_{int} = 0.0626, R_{sigma} = 0.0435]
Data/restraints/parameters	7944/202/544
Goodness-of-fit on F^2	1.035
Final R indexes [I>=2σ (I)]	R_1 = 0.0569, wR_2 = 0.1320
Final R indexes [all data]	R_1 = 0.0995, wR_2 = 0.1548
Largest diff. peak / hole / e Å^{-3}	0.29/-0.32
CCDC number	2263050

Table S8. Crystal data and structure refinement for ***(S, S)*-3c**.

Crystallization Solvent	chloroform / *n*-hexane
Empirical formula	$C_{46}H_{42}N_2O_6S_2$
Formula weight	782.93
Temperature / K	293(2)
Crystal system	orthorhombic
Space group	$P2_12_12_1$
a / Å	12.1690(6)
b / Å	15.9875(10)
c / Å	24.1388(16)
α / °	90
β / °	90
γ / °	90
Volume / Å^3	4696.2(5)
Z	4
ρ_{calc} / g cm^{-3}	1.107
μ / mm^{-1}	1.386
F (000)	1648.0
Crystal size / mm^3	0.02 × 0.02 × 0.02
Radiation	Cu $K\alpha$ (λ = 1.54184)
2θ range for data collection / °	7.324 to 150.024
Index ranges	$-15 \le h \le 9$, $-19 \le k \le 18$, $-30 \le l \le 29$
Reflections collected	27092
Independent reflections	9115 [R_{int} = 0.0930, R_{sigma} = 0.0744]
Data/restraints/parameters	9115/0/511
Goodness-of-fit on F^2	0.975
Final R indexes [I>=2σ (I)]	R_1 = 0.0809, wR_2 = 0.1922
Final R indexes [all data]	R_1 = 0.1206, wR_2 = 0.2205
Largest diff. peak / hole / e Å^{-3}	0.57/-0.25
Flack parameter	0.016(18)
CCDC number	2265497

Table S9. Crystal data and structure refinement for ***(R, R)*-3c**.

Crystallization Solvent	ethyl acetate / *n*-hexane
Empirical formula	$C_{46}H_{42}N_2O_6S_2$
Formula weight	782.93
Temperature / K	160.00(10)
Crystal system	monoclinic
Space group	$P2_1$
a / Å	11.9854(2)
b / Å	19.9068(3)
c / Å	17.0740(2)
α / °	90
β / °	92.494(2)
γ / °	90
Volume / Å^3	4069.84(10)
Z	4
ρ_{calc} / g cm^{-3}	1.278
μ / mm^{-1}	1.599
F (000)	1648.0
Crystal size / mm^3	0.2 × 0.2 × 0.05
Radiation	Cu $K\alpha$ (λ = 1.54184)
2θ range for data collection / °	4.438 to 155.546
Index ranges	$-15 \leq h \leq 15$, $-24 \leq k \leq 25$, $-15 \leq l \leq 21$
Reflections collected	56313
Independent reflections	16404 [R_{int} = 0.0509, R_{sigma} = 0.0422]
Data/restraints/parameters	16404/3/1022
Goodness-of-fit on F^2	1.052
Final R indexes [I>=2σ (I)]	R_1 = 0.1038, wR_2 = 0.2990
Final R indexes [all data]	R_1 = 0.1067, wR_2 = 0.3018
Largest diff. peak / hole / e Å^{-3}	1.92/-0.54
Flack parameter	0.015(6)
CCDC number	2265505

Table S10. Crystal data and structure refinement for **(*S*, *S*)-3o**.

Crystallization Solvent	ethyl acetate / *n*-hexane
Empirical formula	$C_{52}H_{46}N_2O_7S_2$
Formula weight	875.03
Temperature / K	100.00(13)
Crystal system	monoclinic
Space group	$P2_1$
a / Å	6.25350(10)
b / Å	33.9648(6)
c / Å	10.4728(2)
α / °	90
β / °	105.672(2)
γ / °	90
Volume / Å^3	2141.72(7)
Z	2
ρ_{calc} / g cm^{-3}	1.357
μ / mm^{-1}	1.599
F (000)	920.0
Crystal size / mm^3	0.1 × 0.05 × 0.05
Radiation	Cu $K\alpha$ (λ = 1.54184)
2θ range for data collection / °	5.204 to 153.046
Index ranges	$-7 \leq h \leq 7$, $-42 \leq k \leq 42$, $-13 \leq l \leq 13$
Reflections collected	26420
Independent reflections	8537[R_{int} = 0.0433, R_{sigma} = 0.0446]
Data/restraints/parameters	8537/3/596
Goodness-of-fit on F^2	1.052
Final R indexes [I>=2σ (I)]	R_1 = 0.0370, wR_2 = 0.0874
Final R indexes [all data]	R_1 = 0.0414, wR_2 = 0.0894
Largest diff. peak / hole / e Å^{-3}	0.24/-0.40
Flack parameter	0.012 (7)
CCDC number	2265382

Table S11. Crystal data and structure refinement for **meso-3u**.

Crystallization Solvent	dichloromethane / *n*-hexane
Empirical formula	$C_{35}H_{28}N_2O_6S_2$
Formula weight	636.71
Temperature / K	159.99(10)
Crystal system	monoclinic
Space group	$P2_1/c$
a / Å	5.87370(10)
b / Å	31.6045(6)
c / Å	16.2674(2)
α / °	90
β / °	91.8500(10)
γ / °	90
Volume / $Å^3$	3018.23(9)
Z	4
ρ_{calc} / g cm^{-3}	1.401
μ / mm^{-1}	2.024
F (000)	1328.0
Crystal size / mm^3	0.15 × 0.015 × 0.015
Radiation	Cu $K\alpha$ (λ = 1.54184)
2θ range for data collection / °	5.592 to 151.372
Index ranges	$-7 \leq h \leq 5$, $-38 \leq k \leq 39$, $-20 \leq l \leq 20$
Reflections collected	54593
Independent reflections	6140 [R_{int} = 0.0728, R_{sigma} = 0.0336]
Data/restraints/parameters	6140/0/408
Goodness-of-fit on F^2	1.059
Final R indexes [I>=2σ (I)]	R_1 = 0.0410, wR_2 = 0.1158
Final R indexes [all data]	R_1 = 0.0450, wR_2 = 0.1193
Largest diff. peak / hole / e $Å^{-3}$	0.46/-0.53
CCDC number	2265410

Table S12. Crystal data and structure refinement for ***(S, S)*-3w**.

Crystallization Solvent	dichloromethane / methanol
Empirical formula	$C_{74}H_{64}N_4O_{12}S_4$
Formula weight	1329.53
Temperature / K	160.00(10)
Crystal system	orthorhombic
Space group	$P2_12_12_1$
a / Å	13.97000(10)
b / Å	16.0090(2)
c / Å	30.3827(3)
α / °	90
β / °	90
γ / °	90
Volume / Å^3	6794.96(12)
Z	4
ρ_{calc} / g cm^{-3}	1.300
μ / mm^{-1}	1.820
F (000)	2784.0
Crystal size / mm^3	0.04 × 0.03× 0.01
Radiation	Cu $K\alpha$ (λ = 1.54184)
2θ range for data collection / °	5.818 to 153.022
Index ranges	$-11 \leq h \leq 17$, $-20 \leq k \leq 19$, $-38 \leq l \leq 37$
Reflections collected	45773
Independent reflections	13513 [R_{int} = 0.0324, R_{sigma} = 0.0297]
Data/restraints/parameters	13513/0/855
Goodness-of-fit on F^2	1.028
Final R indexes [I>=2σ (I)]	R_1 = 0.0449, wR_2 = 0.1191
Final R indexes [all data]	R_1 = 0.0506, wR_2 = 0.1230
Largest diff. peak / hole / e Å^{-3}	0.63/-0.55
Flack parameter	0.005(5)
CCDC number	2265407

Table S13. Crystal data and structure refinement for ***(R, R)*-3w**.

Crystallization Solvent	dichloromethane / methanol
Empirical formula	$C_{74}H_{64}N_4O_{12}S_4$
Formula weight	1329.53
Temperature / K	159.99(10)
Crystal system	orthorhombic
Space group	$P2_12_12_1$
a / Å	13.9703(3)
b / Å	16.0072(2)
c / Å	30.3518(6)
α / °	90
β / °	90
γ / °	90
Volume / $Å^3$	6787.4(2)
Z	4
ρ_{calc} / g cm^{-3}	1.301
μ / mm^{-1}	1.822
F (000)	2784.0
Crystal size / mm^3	0.05 × 0.03× 0.02
Radiation	Cu $K\alpha$ (λ = 1.54184)
2θ range for data collection / °	5.824 to 152.074
Index ranges	$-17 \leq h \leq 17$, $-12 \leq k \leq 19$, $-38 \leq l \leq 36$
Reflections collected	42850
Independent reflections	13471 [R_{int} = 0.0390, R_{sigma} = 0.0393]
Data/restraints/parameters	13471/0/855
Goodness-of-fit on F^2	1.057
Final R indexes [I>=2σ (I)]	R_1 = 0.0485, wR_2 = 0.1189
Final R indexes [all data]	R_1 = 0.0574, wR_2 = 0.1246
Largest diff. peak / hole / e $Å^{-3}$	0.52/-0.52
Flack parameter	0.011(6)
CCDC number	2265406

Table S14. Crystal data and structure refinement for **(*S, S*)-3y**.

Crystallization Solvent	ethyl acetate / *n*-hexane
Empirical formula	$C_{104}H_{92}N_4O_{12}S_4$
Formula weight	1718.05
Temperature / K	293(2)
Crystal system	triclinic
Space group	*P*1
a / Å	10.81630(10)
b / Å	12.65600(10)
c / Å	17.42540(10)
α / °	99.9470(10)
β / °	106.1830(10)
γ / °	90.0420(10)
Volume / Å^3	2253.41(3)
Z	1
ρ_{calc} / g cm^{-3}	1.266
μ / mm^{-1}	1.492
F (000)	904.0
Crystal size / mm^3	0.1 × 0.05 × 0.05
Radiation	Cu $K\alpha$ (λ = 1.54184)
2θ range for data collection / °	5.368 to 151.458
Index ranges	$-13 \leq h \leq 11$, $-15 \leq k \leq 15$, $-21 \leq l \leq 21$
Reflections collected	80508
Independent reflections	16743[R_{int} = 0.0829, R_{sigma} = 0.0545]
Data/restraints/parameters	16743/3/1129
Goodness-of-fit on F^2	1.087
Final *R* indexes [I>=2σ (I)]	R_1 = 0.0545, wR_2 = 0.1489
Final *R* indexes [all data]	R_1 = 0.0688, wR_2 = 0.1588
Largest diff. peak / hole / e Å^{-3}	0.17/-0.37
Flack parameter	0.041 (13)
CCDC number	2265383

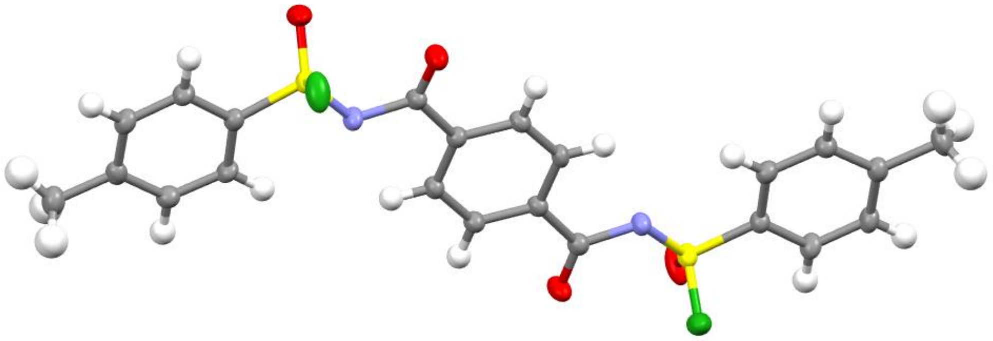

Figure S185. X-ray crystal structure of compound ***(R, R)*-1a** in a thermal ellipsoid style (the ellipsoid contour probability level 50%).

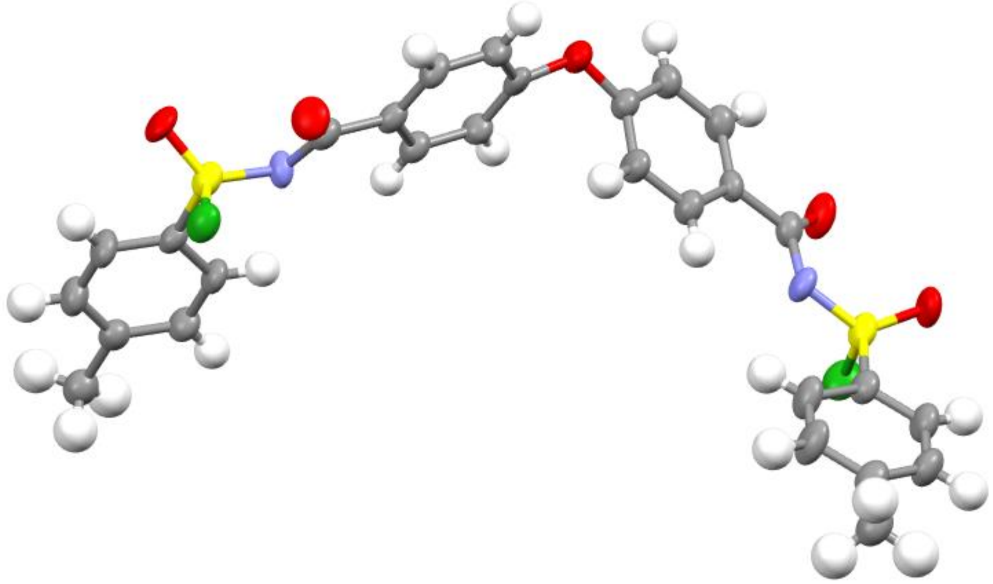

Figure S186. X-ray crystal structure of compound ***(R, R)*-1b** in a thermal ellipsoid style (the ellipsoid contour probability level 50%).

.

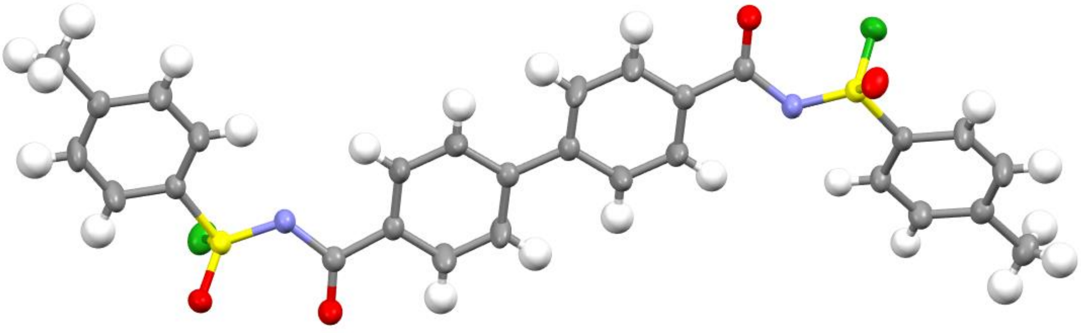

Figure S187. X-ray crystal structure of compound ***(R, R)*-1e** in a thermal ellipsoid style (the ellipsoid contour probability level 50%).

.

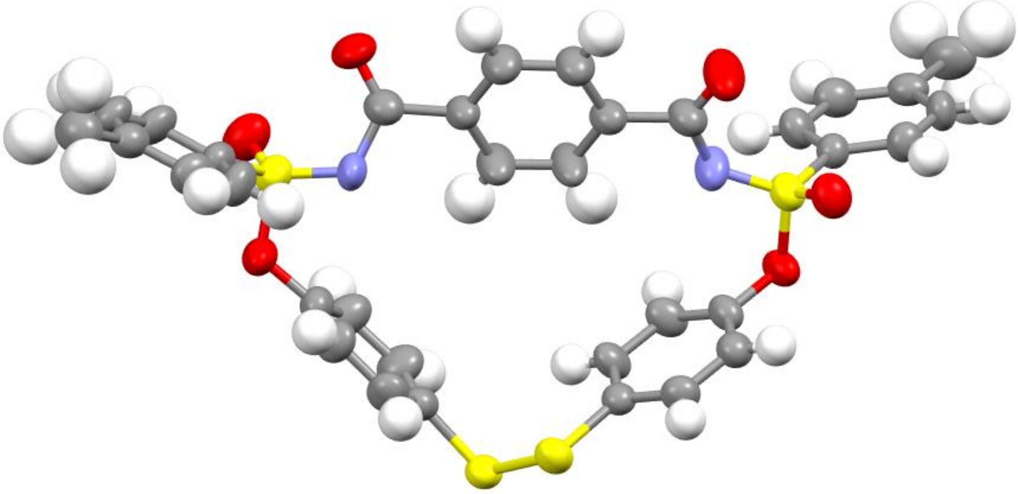

Figure S188. X-ray crystal structure of compound **(*S*, *S*)-3a** in a thermal ellipsoid style (the ellipsoid contour probability level 50%).

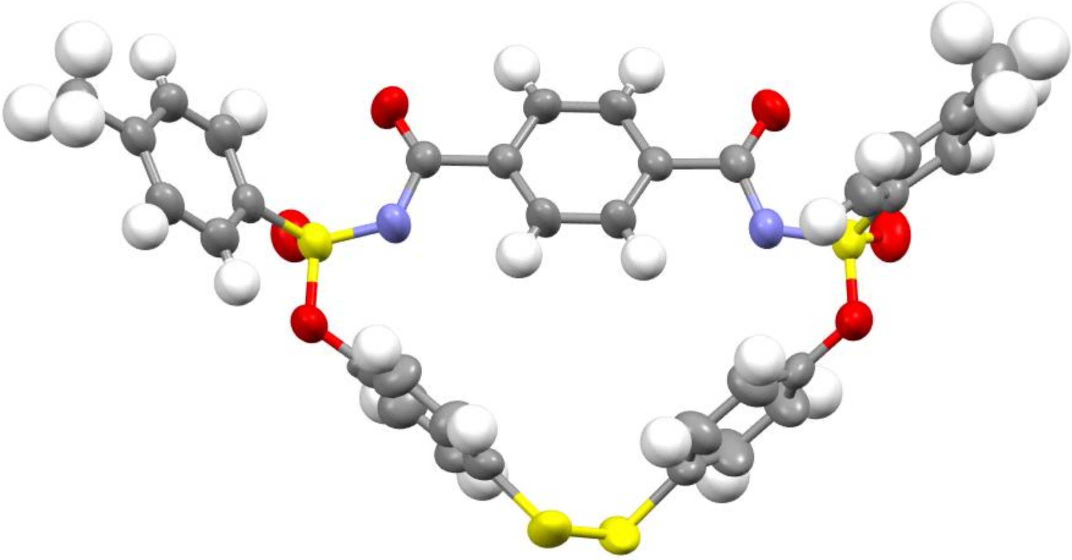

Figure S189. X-ray crystal structure of compound **meso-3a** in a thermal ellipsoid style (the ellipsoid contour probability level 50%).

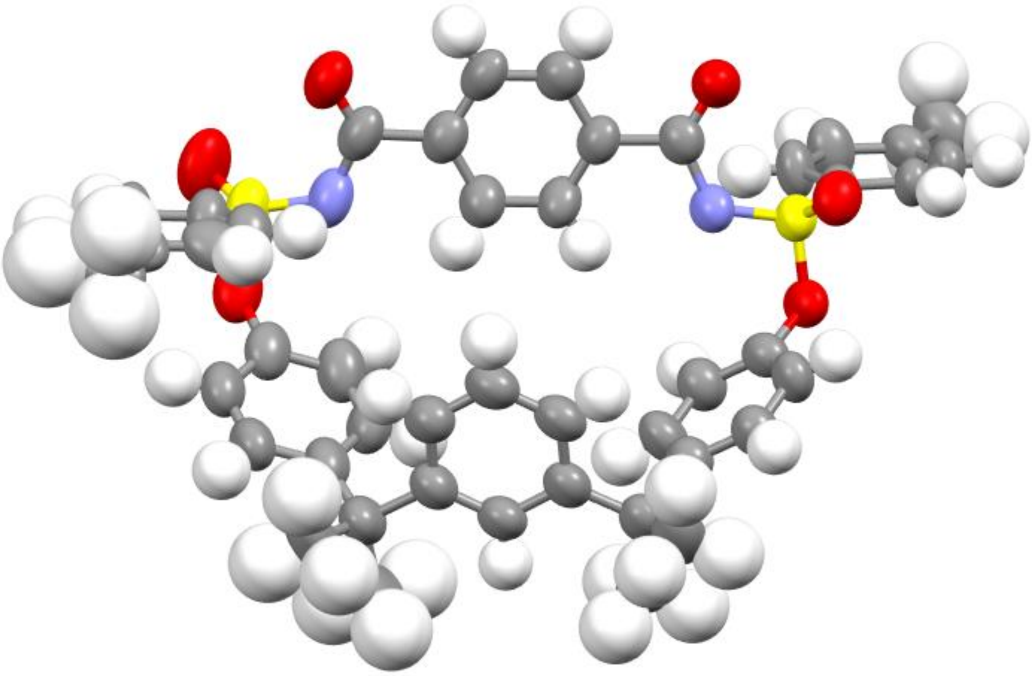

Figure S190. X-ray crystal structure of compound ***(S, S)*-3c** in a thermal ellipsoid style (the ellipsoid contour probability level 50%).

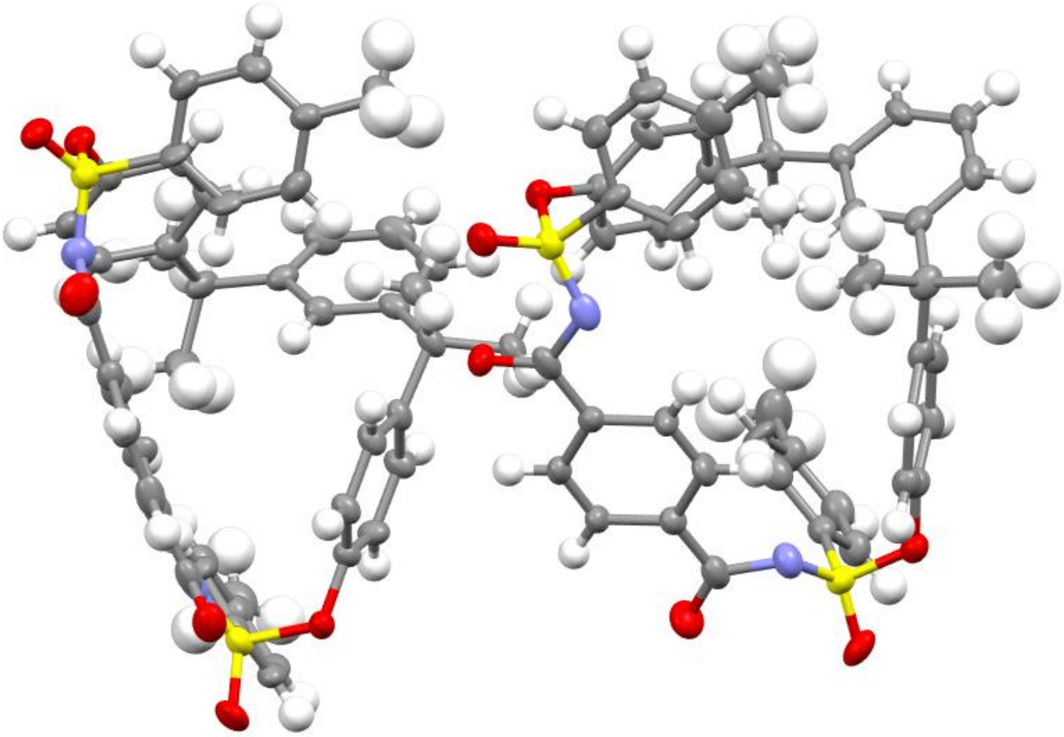

Figure S191. X-ray crystal structure of compound ***(R, R)*-3c** in a thermal ellipsoid style (the ellipsoid contour probability level 50%).

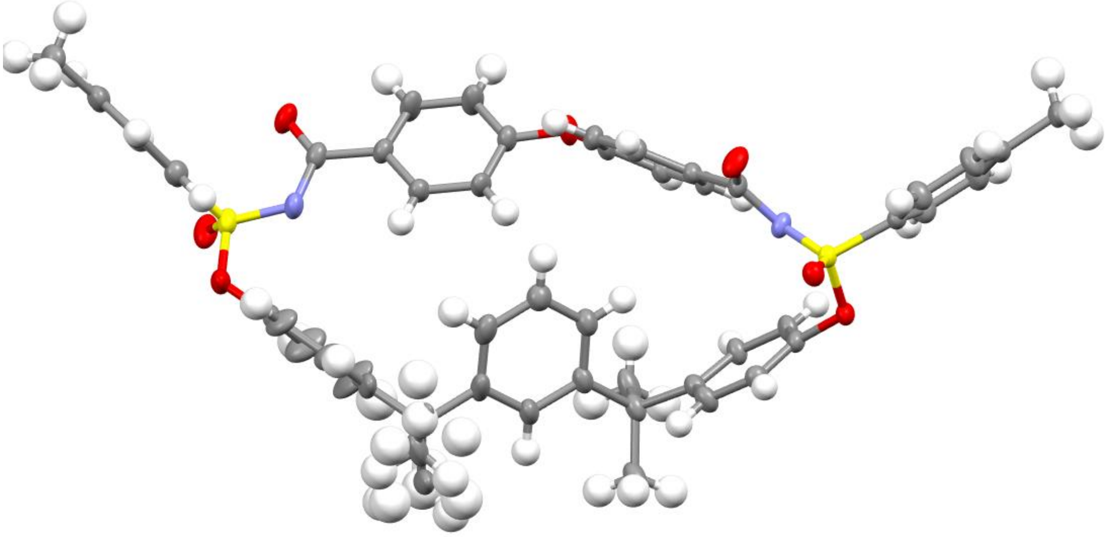

Figure S192. X-ray crystal structure of compound **(*S*, *S*)-3o** in a thermal ellipsoid style (the ellipsoid contour probability level 50%).

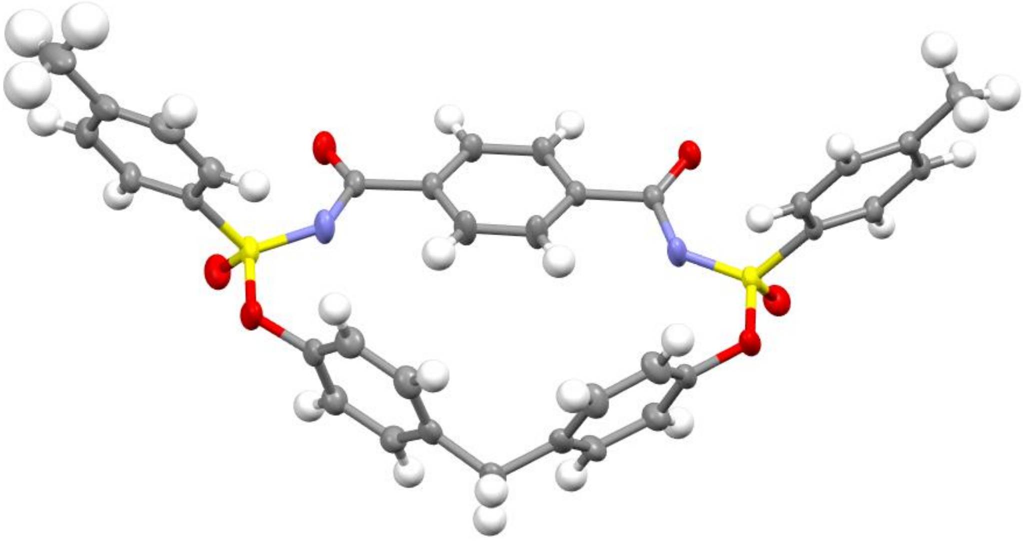

Figure S193. X-ray crystal structure of compound **meso-3u** in a thermal ellipsoid style (the ellipsoid contour probability level 50%).

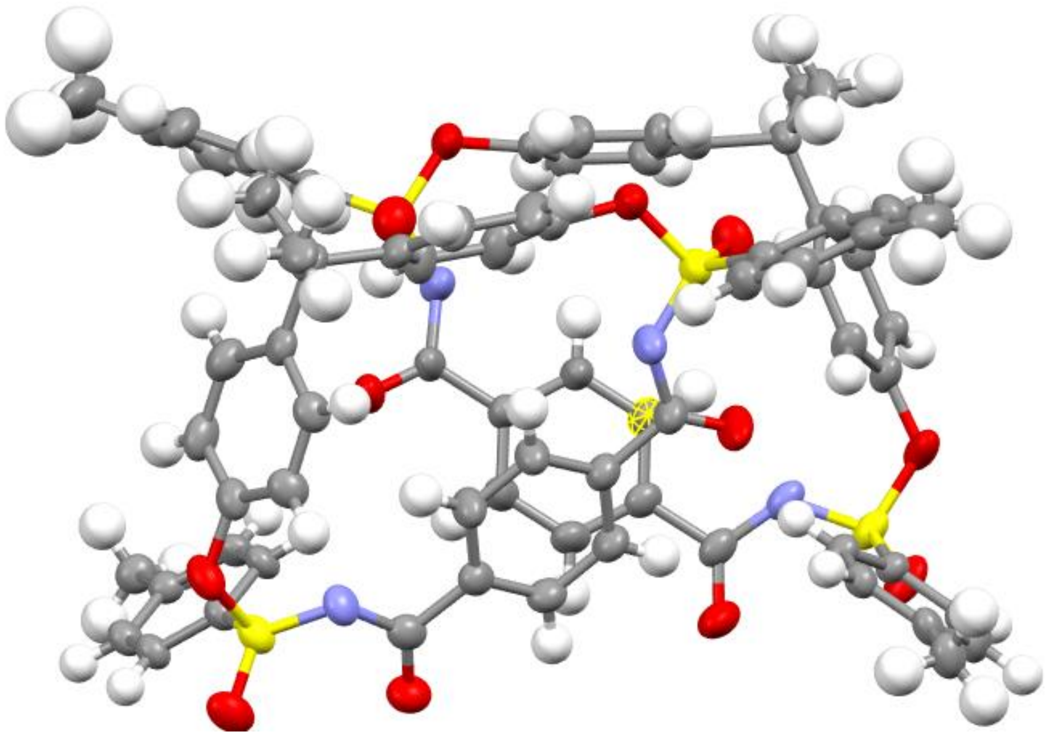

Figure S194. X-ray crystal structure of compound ***(S, S)*-3w** in a thermal ellipsoid style (the ellipsoid contour probability level 50%).

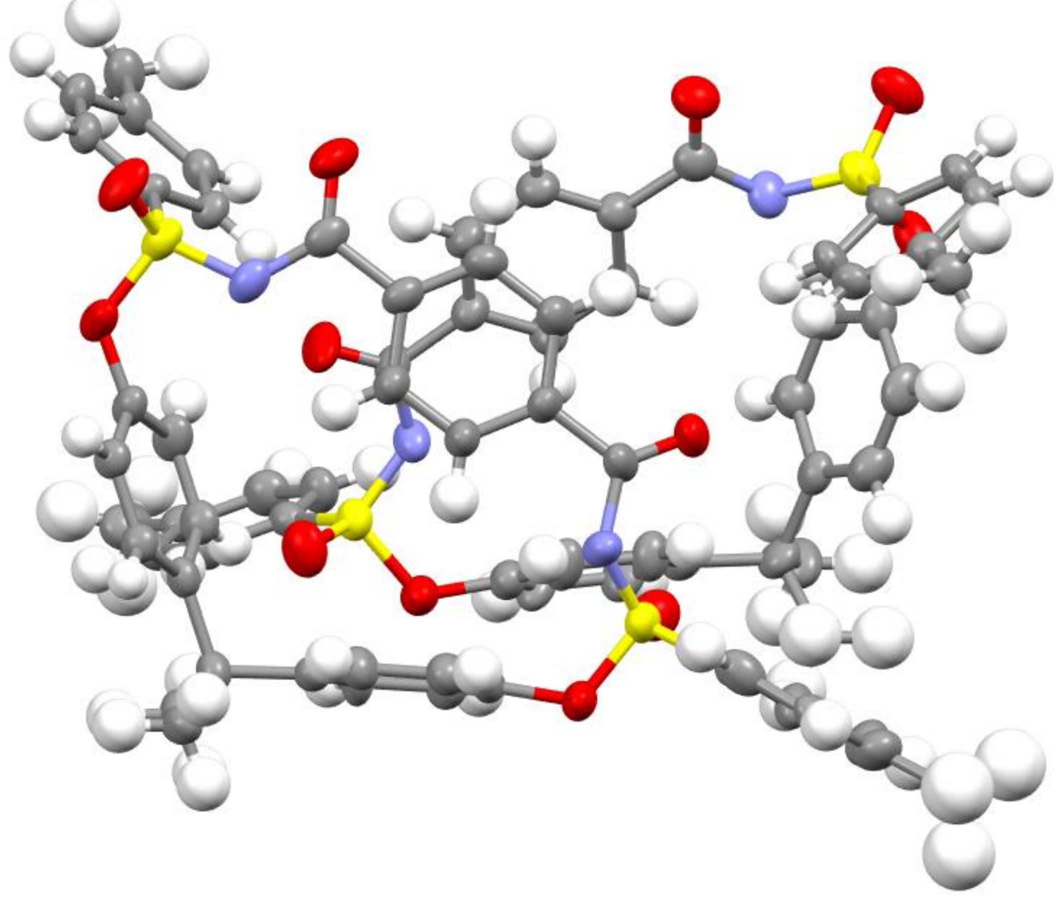

Figure S195. X-ray crystal structure of compound ***(R, R)*-3w** in a thermal ellipsoid style (the ellipsoid contour probability level 50%).

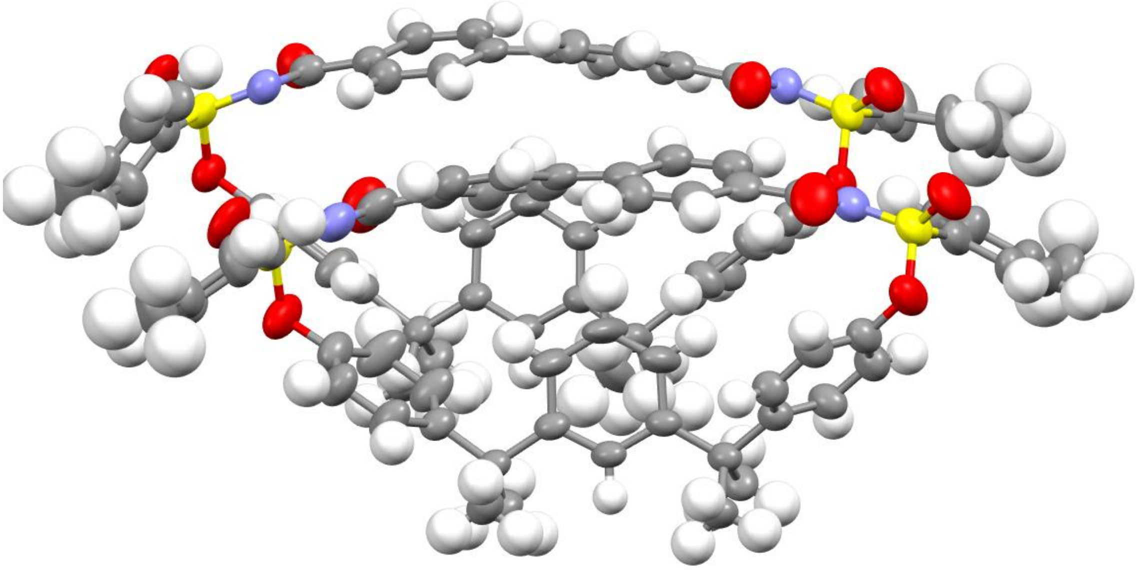

Figure S196. X-ray crystal structure of compound **(*S, S*)-3y** in a thermal ellipsoid style (the ellipsoid contour probability level 50%).

8. References

[1] D-D. Liang, S. P. Pujari, M. Subramaniam, M. Besten, H. Zuilhof. *Angew. Chem. Int. Ed*. **2022**, *61*, e202116158; *Angew. Chem*. **2022**, *134*, e202116158.

[2] P. S. Bhadury, V. Dubey, S. Singh, C. Saxena, 2,2-Bis(3-Allyl-4-Hydroxyphenyl) Hexafluoropropane and Fluorosiloxane as Coating Materials for Nerve Agent Sensors. *J. Fluorine Chem.* **2005**, *126*, 1252−1256.

[3] P. Groves. Diffusion Ordered Spectroscopy (DOSY) as Applied to Polymers. *Polym. Chem.,* **2017**, *8*, 6700−6708.

[4] OLEX2: A Complete Structure Solution, Refinement and Analysis Program. *J. Appl. Crystallogr.* **2009**, *42*, 339−341.

[5] Crystal Structure Refinement with \it SHELXL. *Acta Crystallographica Section C* **2015**, *71*, 3−8.

[6] A Short History of SHELX. *Acta Crystallographica Section A* **2008**, *64*, 112−122.
